# DNA methylation signature in blood mirrors successful weight-loss during lifestyle interventions: the CENTRAL trial

**DOI:** 10.1186/s13073-020-00794-7

**Published:** 2020-11-16

**Authors:** Maria Keller, Anat Yaskolka Meir, Stephan H. Bernhart, Yftach Gepner, Ilan Shelef, Dan Schwarzfuchs, Gal Tsaban, Hila Zelicha, Lydia Hopp, Luise Müller, Kerstin Rohde, Yvonne Böttcher, Peter F. Stadler, Michael Stumvoll, Matthias Blüher, Peter Kovacs, Iris Shai

**Affiliations:** 1 Helmholtz Institute for Metabolic, Obesity and Vascular Research (HI-MAG) of the Helmholtz Center Munich at the University of Leipzig and University Hospital Leipzig, 04103 Leipzig, Germany; 2 Medical Department III – Endocrinology, Nephrology, Rheumatology, University of Leipzig Medical Center, 04103 Leipzig, Germany; 3 IFB Adiposity Diseases, University of Leipzig, Liebigstrasse 19-21, 04103 Leipzig, Germany; 4 Faculty of Health Sciences, Ben-Gurion University of the Negev, P.O.Box 653, 84105 Beer Sheva, Israel; 5 Interdisciplinary Center for Bioinformatics, University of Leipzig, 04107 Leipzig, Germany; 6 Bioinformatics Group, Department of Computer Science, University of Leipzig, 04107 Leipzig, Germany; 7 Transcriptome Bioinformatics, LIFE Research Center for Civilization Diseases, University of Leipzig, 04107 Leipzig, Germany; 8 Department of Epidemiology and Preventive Medicine, School of Public Health, Sackler Faculty of Medicine and Sylvan Adams Sports Institute, Tel Aviv University, 6997801 Ramat Aviv, Israel; 9 Soroka University Medical Center, 84101 Beer-Sheva, Israel; 10 Nuclear Research Center-Negev, 84190 Dimona, Israel; 11 Department of Clinical Molecular Biology, Institute of Clinical Medicine, University of Oslo, 0316 Oslo, Norway; 12 Medical Division, Akershus University Hospital, 1478 Lørenskog, Norway; 13 Competence Center for Scalable Data Services and Solutions Dresden/Leipzig, German Centre for Integrative Biodiversity Research (iDiv), and Leipzig Research Center for Civilization Diseases, University of Leipzig, 04109 Leipzig, Germany; 14 Max Planck Institute for Mathematics in the Sciences, 04103 Leipzig, Germany; 15 Fraunhofer Institute for Cell Therapy and Immunology, 04103 Leipzig, Germany; 16 Department of Theoretical Chemistry, University of Vienna, 1090 Vienna, Austria; 17 Center for RNA in Technology and Health, University of Copenhagen, 1871 Frederiksberg, Denmark; 18 Santa Fe Institute, Santa Fe, NM 87501 USA; 19 Deutsches Zentrum für Diabetesforschung, Helmholtz Zentrum München, Neuherberg, 85764 USA

**Keywords:** Lifestyle intervention, Weight-loss, Epigenetics, DNA methylation, Gene

## Abstract

**Background:**

One of the major challenges in obesity treatment is to explain the high variability in the individual’s response to specific dietary and physical activity interventions. With this study, we tested the hypothesis that specific DNA methylation changes reflect individual responsiveness to lifestyle intervention and may serve as epigenetic predictors for a successful weight-loss.

**Methods:**

We conducted an explorative genome-wide DNA methylation analysis in blood samples from 120 subjects (90% men, mean ± SD age = 49 ± 9 years, body mass-index (BMI) = 30.2 ± 3.3 kg/m^2^) from the 18-month CENTRAL randomized controlled trial who underwent either Mediterranean/low-carbohydrate or low-fat diet with or without physical activity.

**Results:**

Analyses comparing male subjects with the most prominent body weight-loss (responders, mean weight change − 16%) vs. non-responders (+ 2.4%) (*N* = 10 each) revealed significant variation in DNA methylation of several genes including *LRRC27*, *CRISP2*, and *SLFN12* (all adj. *P* < 1 × 10^−5^)*.* Gene ontology analysis indicated that biological processes such as cell adhesion and molecular functions such as calcium ion binding could have an important role in determining the success of interventional therapies in obesity. Epigenome-wide association for relative weight-loss (%) identified 15 CpGs being negatively correlated with weight change after intervention (all combined *P* < 1 × 10^− 4^) including new and also known obesity candidates such as *NUDT3* and *NCOR2*. A baseline DNA methylation score better predicted successful weight-loss [area under the curve (AUC) receiver operating characteristic (ROC) = 0.95–1.0] than predictors such as age and BMI (AUC ROC = 0.56).

**Conclusions:**

Body weight-loss following 18-month lifestyle intervention is associated with specific methylation signatures. Moreover, methylation differences in the identified genes could serve as prognostic biomarkers to predict a successful weight-loss therapy and thus contribute to advances in patient-tailored obesity treatment.

## Background

Obesity represents a major health burden worldwide [1]. Increasing energy expenditure and limiting caloric intake are the major set points to control obesity; however, only restricted long-term success could be reached so far, potentially caused by hormonal, metabolic, and neurochemical adaptations that stabilize weight-loss and may lead to weight regain [2]. The majority of individuals who experience weight-loss will regain it over time [3, 4]. Thus, the effective long-term treatment of obesity would require a systematic assessment and understanding of genetic, epigenetic, and lifestyle factors that potentially affect energy intake, metabolism, and energy expenditure. Therefore, a better understanding of this highly complex interaction is required to explain the high variability in the individual’s response to specific dietary and physical activity (PA) interventions. This would allow to develop more successful preventive and therapeutic strategies ultimately leading to personalized lifestyle treatments in the battle against obesity [5, 6].

Whereas poor adherence to different lifestyle interventions represents a strong factor in response to weight-loss therapies [7], emerging evidence implies that genetic and epigenetic predictors play a role in inter-individual variability of metabolic response [8]. Further, this individual response in weight regain is mainly driven by an unadjusted energy intake after the intervention [9], since after successful weight-loss less caloric intake is required to maintain the achieved weight. Several recent findings directly link obesity development to DNA methylation changes in related target tissues such as adipose tissue (AT) [10, 11], skeletal muscle [12–14], and also in blood [15–18]. DNA methylation marks in whole blood samples have been reported to correlate with target tissue changes [17] and would thereby represent an easy accessible proxy for the future development of personalized treatment strategies and prediction of therapeutical success. However, DNA methylation changes upon long-term behavioral interventions (e.g., specific diets, exercise) are scarcely investigated so far.

In the present study, we conducted a genome-wide DNA methylation analysis in blood samples from 120 subjects who underwent the 18-month randomized controlled trial (RCT) CENTRAL [19]. The CENTRAL trial has been conducted under strict monitoring conditions in the Dimona Nuclear Research Center, Negev, located in a desert in Israel, thus providing an almost homogenous environment and a low drop off rate. In this exploratory study, we tested the hypotheses that (i) metabolic changes mediated by different types of lifestyle intervention including diet and PA (Mediterranean low-carb (MED/LC) vs. low-fat (LF) vs. MED/LC *+* PA vs. LF + PA) correlate with variation in DNA methylation and (ii) that specific DNA methylation signatures reflect individual responsiveness to lifestyle intervention to serve as epigenetic predictors for successful weight-loss.

## Methods

### Study population and design

The CENTRAL RCT was conducted between 2012 and 2014 in an isolated nuclear research center workplace in Israel and primarily aimed to assess changes on visceral fat depots after diet and exercise interventions. The center provides a sophisticated infrastructure including an internal clinic, a cafeteria, and a designated space for lifestyle and PA sessions, thus allowing this well-structured and precisely controlled lifestyle intervention trial. Two hundred seventy-eight of the participants with a mean age of 48 years and a mean body mass index (BMI) of 30.8 kg/m^2^ fulfilled the pre-specified inclusion criteria for the trial. Inclusion criteria for the exploratory analyses were, first, either abdominal obesity (waist circumference (WC) > 102 cm for men and > 88 cm for women) or dyslipidemia (serum triglycerides > 150 mg/dL and high-density-lipoprotein cholesterol (HDL-C) < 40 mg/dL for men and < 50 mg/dL for women); second, the provision of signed and dated informed consent form; and third, the stated willingness to comply with all study procedures and availability for the duration of the study. Exclusion criteria included pregnant or lactating women, subjects with serum creatinine ≥2 mg/dL, with disturbed liver function (≥ 3-fold level of ALT and AST enzymes), active cancer, individuals who had any restrictions regarding physical activity, were highly physical active (> 3 h/week) or were included in other nutritional trials (https://clinicaltrials.gov/ct2/show/NCT01530724) [19, 20].

The study was conducted in accordance with the Declaration of Helsinki, and the protocol for the exploratory analyses was approved by the Medical Ethics Board and Institutional Review Board at Soroka University Medical Center, Be’er Sheva, Israel (0239-11SOR). All participants provided written informed consent before taking part in the study.

**Fig. 1:**
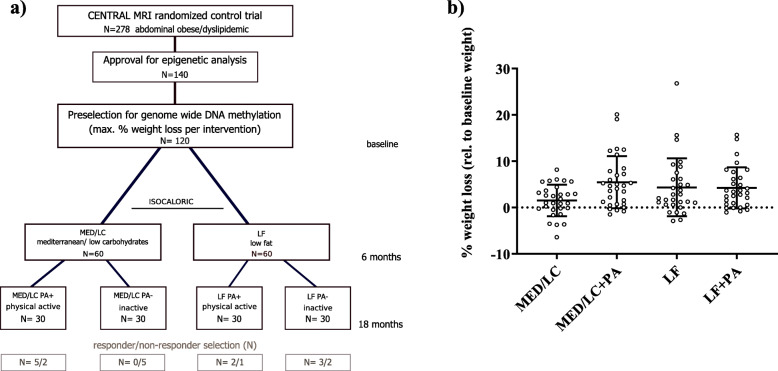
Study design—CENTRAL RCT. **a** shows the study design of the CENTRAL RCT over the three time points: baseline, 6 months, and 18 months; **b** shows the weight-loss at 18 months relative to the baseline weight as mean ± SD (%)

Subjects were randomly assigned to an either LF or MED/LC diet (*N* = 139 each). Both dietary interventions were equal in calories and maintained over the entire study period. After 6 months, each intervention arm was re-randomized to a group with moderate, mostly aerobic (80%) PA (LF + PA; MED/LC + PA) or without PA (LF-PA; MED/LC-PA) for another year of intervention. Details about the study environment, interventions, endpoint measurements, and detailed metabolic phenotyping can be obtained elsewhere [19, 20]. The overall study design is presented in Fig. 1a.

### Sample selection and preparation

Among all CENTRAL participants, a total number of 140 subjects with both baseline and 18 months available blood samples gave additional consent to genetic analysis. Out of this subgroup 30 subjects per intervention group showing the lowest relative weight after 18 months with respect to their initial weight were selected and included in DNA methylation analysis. Details about intervention group-specific phenotypes can be found in Table 1, whereas individual weight-loss (%) is shown in Fig. 1b.

**Table 1:** Study characteristics of the CENTRAL subgroup selected for genome wide DNA methylation analysis

Trait	CENTRAL all (***N*** = 120)	***P*** value	Low carb (***N*** = 30)	Low carb + PA (***N*** = 30)	Low fat (***N*** = 30)	Low fat + PA (***N*** = 30)
Gender (*N*; female/male)	10/110		4/26	0/30	5/25	1/29
Age (years)						
T_0_	49 ± 9.3		47 ± 7.5	50 ± 9.8	50 ± 10.2	48 ± 9.5
Weight (kg)						
T_0_	90.32 ± 11.5		89.98 ± 14.5	90.58 ± 11.6	87.64 ± 10.4	93.07 ± 8.5
T_18_	86.66 ± 11.2		88.50 ± 14.2	85.44 ± 10.8	83.61 ± 9.5	89.11 ± 9.1
Δ_(T18-T0)_	− 3.65 ± 5.2	**< 1 × 10**^**−11**^	− 1.48 ± 3.3*	− 5.14 ± 5.6***	− 4.04 ± 6.6**	− 3.96 ± 4.2***
BMI (kg/m^2^)						
T_0_	30.17 ± 3.3		30.09 ± 4.6	30.13 ± 2.4	30.38 ± 3.3	30.08 ± 2.5
T_18_	28.97 ± 3.4		29.60 ± 4.4	28.45 ± 2.5	29.02 ± 3.4	28.80 ± 2.8
Δ_(T18-T0)_	− 1.20 ± 1.7	**< 1 × 10**^**−11**^	− 0.49 ± 1.1*	− 1.68 ± 1.8***	− 1.36 ± 2.1**	− 1.27 ± 1.4***
Waist circumference (cm)						
T_0_	106.70 ± 8.1		106.56 ± 11.3	108.29 ± 6.7	104.63 ± 7.3	107.56 ± 5.9
T_18_	101.98 ± 8.4		103.49 ± 12.0	100.96 ± 7.0	100.50 ± 6.6	102.92 ± 6.7
Δ_(T18-T0)_	− 4.81 ± 5.6	**< 1 × 10**^**−14**^	− 3.07 ± 4.0***	− 7.3 ± 6.2***	− 3.64 ± 6.3**	− 5.29 ± 5.1***
HbA1c (%)						
T_0_	5.59 ± 0.5		5.66 ± 0.6	5.61 ± 0.4	5.60 ± 0.5	5.49 ± 0.4
T_18_	5.51 ± 0.5		5.62 ± 0.8	5.47 ± 0.4	5.55 ± 0.5	5.39 ± 0.3
Δ_(T18-T0)_	− 0.08 ± 0.3	**0.005**	− 0.04 ± 0.5	− 0.14 ± 0.2***	− 0.06 ± 0.3	− 0.10 ± 0.2**
Insulin (μU/mL)						
T_0_	18.02 ± 11.3		21.06 ± 15.2	16.85 ± 8.15	17.55 ± 12.1	16.56 ± 8.0
T_18_	14.18 ± 7.3		16.66 ± 8.6	13.98 ± 8.1	12.67 ± 6.8	13.50 ± 4.7
Δ_(T18-T0)_	− 3.91 ± 7.8	**< 1 × 10**^**−6**^	− 4.54 ± 10.5*	− 3.40 ± 6.24**	− 4.66 ± 7.8**	− 3.07 ± 6.3*
Visceral adipose tissue area (cm^2^)						
T_0_	176.19 ± 61.3		162.00 ± 63.6	196.5 ± 63.5	160.60 ± 60.8	185.70 ± 51.3
T_18_	128.12 ± 49.8		126.41 ± 47.9	131.33 ± 56.2	121.03 ± 54.3	133.70 ± 41.0
Δ_(T18-T0)_	− 48.19 ± 36.2	**< 1 × 10**^**− 27**^	−35.54 ± 32.7***	− 65.21 ± 38.1***	− 39.73 ± 28.4***	− 52.00 ± 38.6***
Deep subcutaneous adipose tissue area (cm^2^)						
T_0_	210.89 ± 70.0		217.50 ± 79.6	210.40 ± 64.8	201.30 ± 66.9	214.30 ± 70.2
T_18_	144.71 ± 48.7		152.99 ± 60.2	139.50 ± 41.7	143.39 ± 41.6	142.96 ± 50.5
Δ_(T18-T0)_	− 66.66 ± 42.1	**< 1 × 10**^**−33**^	− 64.55 ± 38.6***	− 70.95 ± 45.9***	− 59.59 ± 48.1***	− 71.31 ± 35.9***
Superficial subcutaneous adipose tissue area (cm^2^)						
T_0_	134.89 ± 56.5		146.20 ± 74.7	125.20 ± 47.6	135.90 ± 59.9	132.20 ± 37.9
T_18_	106.76 ± 44.1		121.37 ± 55.8	94.4 ± 31.8	111.29 ± 50.3	99.99 ± 29.53
Δ_(T18-T0)_	− 28.02 ± 23.9	**< 1 × 10**^**−23**^	− 24.85 ± 30.1***	− 30.85 ± 24.5***	− 24.01 ± 22.8***	− 32.23 ± 16.5***

Metabolic traits are shown as mean ± SD values prior intervention (T0), post-intervention (T18), and the intervention specific changes (Δ(T18-T0)). *P* values obtained from paired t-statistics between T0 and T18 are shown for the entire cohort and indicated as: *P < 0.05; **P < 0.01; ***P < 0.001, for the intervention specific changes

Blood samples were taken after an overnight fast at baseline (T0) and at 18 months (T18) after the individuals completed their interventions. Samples were stored at − 80 °C until DNA was extracted following a standard protocol using proteinase K and 0.2% SDS. Samples were integrity controlled using gel-electrophoresis and the concentrations of double-stranded DNA was measured using Quant-iT PicoGreen dsDNA (Invitrogen, ThermoFisher Scientific, Germany) and Quantus (Promega, Germany) technologies.

### Genome-wide DNA methylation

Five hundred nanograms of genomic DNA from each sample was bisulfite converted using EZ DNA Methylation Gold Kit (Zymo Research, Netherlands). Following quality control, amplification, and hybridization on Illumina HumanMethylation850 Bead Chips (Illumina, Inc., San Diego, CA, USA), the Illumina iScan array scanner was used to quantify genome-wide DNA methylation levels at 850 K CpG sites per sample on single-nucleotide resolution (GenomeScan, Leiden, Netherlands).

### Data analysis/statistics

Raw data was first quality controlled using the QC report of the minfi R package [21–23] (Additional file 1). Beta value densities as well as the control probes were within predicted specifications. Probes that did not pass detection *P* value (*P*_detect_ = 0.01) in more than 1% of all 240 samples were excluded from the analysis. Cross-reactive probes [24] as well as probes containing known SNP positions (MASK_snp5_GMAF1p positions from bioconductor’s Illumina EPIC manifest [25]) were per se not excluded from our analysis but are flagged throughout all result and supplementary tables (Additional file 2). Prior to all further analysis steps aimed at identifying differentially methylated regions (DMRs) and specific CpG sites (comparison independent), beta values were computed and quantile normalized using minfi R package ([26], pages 9–10) [21, 22].

#### Cell type composition

As dietary interventions such as western or high-fat diet have been shown to induce systemic inflammation and change the immune cell composition in adipose tissue of mice [27, 28], we analyzed the cell type composition using the Houseman approach [29] adapted to EPIC arrays by Salas et al. [30]. Possible differences in cell-type composition were plotted using ggplot2 and analyzed using Wilcoxon tests in R. As shown in [26] (pages 4–9), none of the cell type population changed significantly after the intervention (comparing T0 vs T18 over all 120 subjects).

Nevertheless, we used the sva R package to correct beta values for cell-type composition in an attempt to reduce noise [31].

#### DNA methylation changes

To identify intervention specific differentially methylated regions (DMRs) between T0 vs. T18 or differences at T18 as a result of the individual interventions, we used the DMR finder metilene [32]. Only DMRs were considered which carried a minimum number of 3 CpGs per DMR with a maximum distance of 1000 nt between the CpGs. Genes from gencode v19 + 1500 nt upstream were intersected using bedtools [33] with the DMRs to annotate the genes. We compared T0 vs. T18, presence or absence of physical activity (PA vs no), low-carb vs low-fat diet, and PA vs no in the two dietary groups.

It has to be noted that methods used to interrogate the data for DMRs can also result in distinctly different findings. Therefore, we performed a single CpG analysis using the dmpFinder function of the minfi package as described in [26] (pages 18–21) [21].

#### Intervention independent changes (responders vs. non-responders)

To investigate differences in DNA methylation levels between the top 10 responders and bottom 10 non-responders (including only men matched for age; Fig. 1a) according to their relative weight-loss after intervention, computed DNA methylation differences at the individual time points (T0; T18) as well as combined data sets (T0 and T18) using metilene were employed to uncover DMRs using the metilene’s two-dimensional Kolmogorov-Smirnov test (2D-KS) under the same criteria mentioned above [32]. Metabolic differences between the groups were calculated in SPSS (V.24) using t-statistics.

#### Predicting methylation marks

To detect individual CpG sites on a genome-wide basis which are associated with the success of weight-loss by a classical lifestyle intervention (independent of the intervention type), Spearman and Pearson correlation analysis were performed individually and combined to take linearity and monotony equally into account and to further reduce potential background noise due to data properties. An epigenome-wide association study (EWAS) for the relative weight loss in % based on the initial body weight (kg) was conducted and plotted using CMplot in R [29]. A receiver operating characteristic (ROC) curve model was used to further test a potential predictive value of a baseline methylation score, computed as mean of all *ß* values from CpG sites. We used 4 methylation-based predictors, two based on CpGs correlating negatively with intervention weight changes with *p* < 0.001 and *p* < 0.0001 and two based on CpGs correlating positively with weight changes: *p* < 0.001 and *p* < 0.0001. We compared these 4 predictors to general intervention predictors such as a linear combination of individuals’ age and BMI (x*age + y*BMI). The analysis was restricted to men’s data sets for all ROC analysis, as there is only a limited number of women and the combination of age and BMI showed different behavior for men’s and women’s data sets. The maximum area under the ROC curve (AUC) was achieved for the coefficients *x* = − 1 for the age and *y* = 2.54 for the BMI in the linear combination ([26], pages 14–17).

#### Statistics

*P* value adjustment was performed using the Benjamini-Hochberg procedure with adj.*P* values < 0.05 considered to be statistically significant. Phenotype correlation analysis at both time points was performed with baseline methylation levels of the identified candidate DMRs and CpGs using again Pearson and Spearman analysis and included the computation of a combined *P* via geometric mean ($\sqrt[n]{\prod \limits_{i=1}^n\chi i}$).

#### ChromHMM prediction

All identified DMRs, as well as the putative EWAS CpGs described above, were aligned to chromatin segments taken from the Epigenomic Roadmap [34] as well as additional cancer cell lines generated as described elsewhere [35] using bedtools [33]. Besides analyzing a background of all cell and tissue types, we focused on AT (adipose tissue-derived mesenchymal stem cells, mesenchymal stem cell-derived adipocyte cultured cells, adipocyte nuclei), intestinal tissue (fetal intestine large, fetal intestines small, small intestines), skeletal muscle (HSMM cell-derived skeletal muscle myotube cells, HSMM skeletal muscle myoblasts cells, skeletal muscle female, skeletal muscle male), and liver tissue.

#### Gene ontology analysis

Probes from the DMRs characterizing methylation differences between responders and non-responders (*P* < 0.05) as well as correlating probes (*P* < 0.05) were taken forward for gene ontology analyses corrected for probe abundance of the EPIC array using R’s missMethyl package [36] and 0.05 as cutoff for the false discovery rate ([26], page 27).

## Results

All subjects included in the DNA methylation analysis lost on average 3.65 ± 5.2 kg (mean ± SD; *P* < 1 × 10^−11^, Table 1 and Fig. 1b) of body weight after 18 months accounting for more than one BMI point. In line with this, the area of visceral AT, deep and superficial subcutaneous AT depots decreased significantly (all *P* < 1 × 10^−20^, Table 1), and obesity-associated metabolic features such as HbA1c and insulin levels clearly improved (all *P* < 0.01, Table 1).

### Specific signatures of DNA methylation between responders and non-responders

**Fig. 2:**
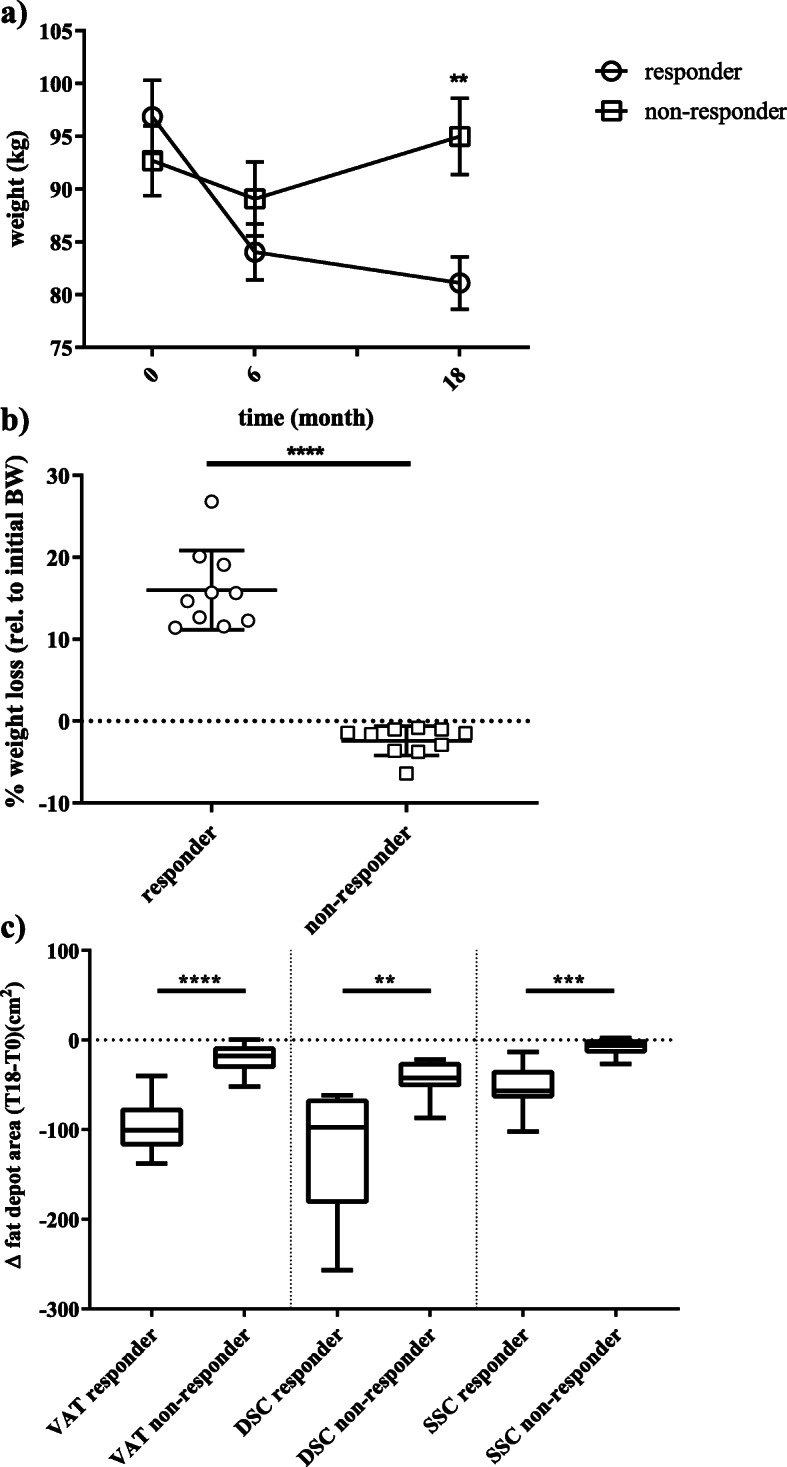
Phenotypic differences between responders and non-responders to a lifestyle intervention. **a** shows the absolute weight of the responders (*N* = 10) and non-responders (N = 10) subgroups over the three time points: baseline (T0), 6 months (T6), and 18 months (T18). Data is shown as mean ± SD; **b** shows the relative (%, rel. to T0) weight-loss at 18 months for both subgroups. Data is shown as scatter dot plots (mean ± SD); **c** shows the absolute changes of fat areas (cm2; VAT-visceral AT, DSC-deep subcutaneous AT, SCC- superficial subcutaneous AT) at 18 months compared to the baseline area as boxplots (line = median) with whiskers representing min and max values; **P < 0.001; ***P < 1 × 10^–3^; ****P < 1 × 10^–4^

First, we conducted analyses to uncover regions potentially discriminating between success and failure of a lifestyle intervention, and we selected 10 male subjects who were referred to as non-responders since they slightly gained weight after intervention and 10 male responders showing the most pronounced weight-loss (Fig. 2a, b). The intervention group distribution of responders and non-responders is provided in Fig. 1a. Both, the top responders and the bottom non-responders (matched with respect to age), lost weight after the first 6 months of diet intervention (Fig. 2a). However, during the following 12 months of intervention, the non-responders regained or even excelled their initial weight whereas the responders lost about 16% of their initial body weight (Fig. 2a, b). Consistently, differences in the area of adipose depots were found between the subgroups of responders and non-responders after 18 months of intervention, with the strongest difference for visceral AT (*P* < 1 × 10^−5^, Fig. 2c).

**Fig. 3:**
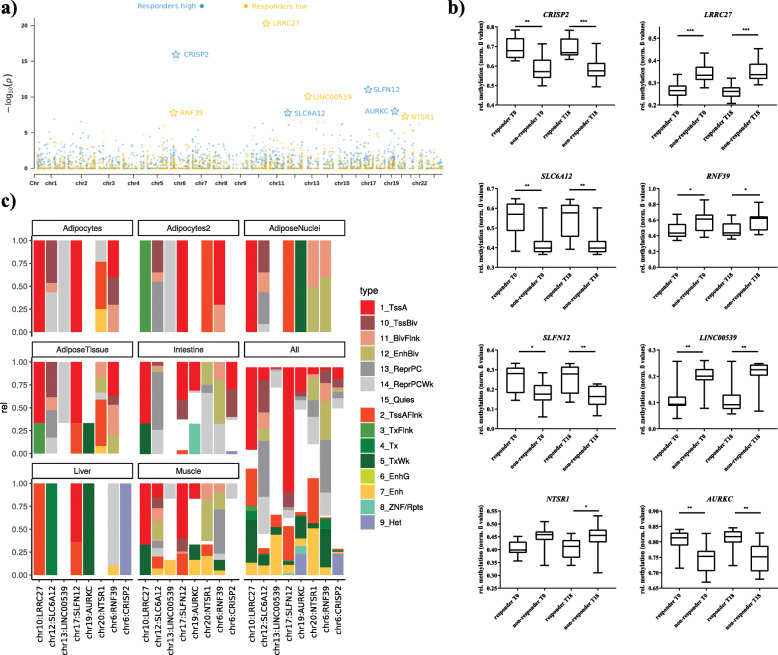
Candidate genes discriminating responders from non-responders. **a** Combined *P* values presented as Manhattan plot for differently methylated DMRs between responders vs. non-responders on a genome-wide scale; blue dots indicate regions were responders showed significantly lower, green dots significantly higher methylation levels compared to non-responders; genes marked with a star remained significant after correction for multiple testing (adj. *P* < 0.05), **b** Methylation levels at the identified genes between responders and non-responders presented as box plots (normalized *ß* values) with whiskers representing min and max values, **P < 0.001; ****P < 1 × 10^−4^. **c** ChromHMM prediction for the significant DMRs (adj. *P* < 0.05). To identify putative target genes in the phenotype relevant target tissues: Intestine (Fetal-Intestine-Large, Fetal-Intestine-Small, Small-Intestines), Adipos-Nuclei (Adipose-Nuclei), AdiposeTissue (Adipose-Nuclei, Adipose-Derived-Mesenchymal-Stem-Cell-Cultured-Cells, Mesenchymal-Stem-Cell-Derived-Adipocyte-Cultured-Cells), Adipocytes (Mesenchymal-Stem-Cell-Derived-Adipocyte-Cultured-Cells), Adipocytes2 (Adipose-Derived-Mesenchymal-Stem-Cell-Cultured-Cells) Liver (Liver), Muscle (HSMM-cell-derived-Skeletal-Muscle-Myotubes-Cells, HSMM-Skeletal-Muscle-Myoblasts-Cells, Skeletal-Muscle-Female, Skeletal-Muscle-Male), and all (all 134 cells from Roadmap Epigenome Gateway). ChromHMM coding for TssA-active TSS, TssBiv-Bivalent (Poised) TSS, BivFlnk-Bivalent Flanking, EnhBiv-Bivalent Enhancer, ReprPC-Polycomb Repressed, ReprPCWk-Weakly Polycomb Repressed, Quies-Quiescent/low, TssAFlnk-Flanking TSS, TxFlnk-Flanking Transcribed, Tx-Transcribed, TxWk-Weakly Transcribed, EnhG- Genetic Enhancer, Enh –Enhancer, ZNF/Rpts-ZNF Genes and Repeats, He-Heterochromatin

Between the two groups, we identified 293 DMRs (2D-KS *P* value< 0.05; comprising 332 genes; 33 DMRs without genes) at baseline, i.e., prior to lifestyle intervention, and 280 DMRs (331 genes; 43 DMRs without genes) after completion of the intervention. However, both before and after intervention, only two DMRs (mapped genes: *CRISP2* and *LRRC27*) remained significant after correction for multiple testing (Additional file 2: Table S1 and S2). Nevertheless, between both time points 150 DMRs corresponding to 168 genes intersected with consistent differences in DNA methylation and were not much affected by weight-loss intervention. Therefore, to minimize the effect of potential outliers by increasing the sample size and so the statistical power, we combined the datasets of both time-points treating the different time-points as biological replicates without any further adjustments for the lack of independence and thereby identified 669 DMRs (759 genes; 100 DMRs without genes) between responders vs. non-responders (Additional file 2: Table S3). After correction for multiple testing 8 DMRs (9 genes) (*P* adjusted < 0.05) remained significant (Table 2, Fig. 3a). Among them, 4 DMRs showed significantly higher (*CRISP2*, *Cysteine Rich Secretory Protein 2*; *SLC6A12*, *Solute Carrier Family 6 Member 12*/*RP11-283I3.2*; *SLFN12*, *Schlafen Family Member 12*; *AURKC*, *Aurora Kinase C*; *deltaM: 0.06–0.13*) and 4 significant lower methylations in responders (*LRRC27*, *Leucine Rich Repeat Containing 27*; *RNF39*, *Ring Finger Protein 39*; *LINC00539*, *Long Intergenic Non-Protein Coding RNA 539*; and *NTSR1*, *Neurotensin Receptor 1*; *deltaM: (− 0.08)-(− 0.11)*) (Fig. 3a/b; Table 2) compared to non-responders. Differences in DNA methylation (normalized *ß* values) for all 8 DMRs are presented in Fig. 3b. Among them, the *SLC6A12 (-RP11-283I3.2)* gene locus revealed the strongest difference in DNA methylation (deltaM: 0.126 = 12.6%; adjusted *P* = 0.008) (Table 2; Fig. 3b) for a DMR at chr12:312736-312753 including 3 CpG sites.

**Table 2:** Genetic regions discriminating responders from non-responders

Location	Adj.***P***	DeltaM	Probes/DMR	2D-KS_***p*** value	SNPprobes	Cross-reactive probes	Genes	Probes/gene
chr10:134150449-134150761	2.4E−15	− 0.082275	8	5E−21	0	0	*LRRC27*	81
chr6:49681176-49681392	6.1E−11	0.101266	8	1.3E−16	0	0	*CRISP2*	14
chr17:33759510-33760309	0.0000054	0.083168	12	1.1E−11	0	0	*SLFN12*	22
chr13:21919004-21919171	0.000038	− 0.090555	3	7.9E−11	0	3	*LINC00539*	39
chr19:57742110-57742424	0.005	0.05827	9	0.000000011	0	0	*AURKC*	16
chr6:30039130-30039802	0.0077	− 0.114807	18	0.000000016	3	0	*RNF39*	83
chr12:311645-313379	0.0082	0.126218	9	0.000000017	1	0	*RP11-283I3.2*, *SLC6A12*	9.59
chr20:61371016-61371809	0.024	− 0.041995	5	0.000000051	3	0	*NTSR1*	38

Differentially methylated DMRs between responders and non-responders to lifestyle intervention; including all datasets at baseline and post-intervention. Top candidate DMRs with adj. *P* ≤ 0.05. DeltaM represents the difference of the mean average methylation rates between responders and non-responder as computed by metilene. The 2D-KS *p* value represents a two-dimensional variety of the Kolmogorov-Smirnov test used by metilene. Probes/DMR represents the number of probes within a DMR. SNPprobes represent probes containing a SNP with a frequency > 0.01. Cross-reactive probes indicate potential off-target probe binding. Genes is a comma-separated list of the genes cis to the DMR, and probes/gene is the total number of probes that are cis to the respective genes. Chromosomal location was annotated to genome assembly GRCh37 (hg19)

Furthermore, among the DMRs which showed significant *P* values in a combined analysis but did not withstand adjustment for multiple testing (*N* = 661), we identified mostly new candidate genes but also confirmed genetic risk loci for BMI (*N* = 256), waist-to-hip ratio (*N* = 154), waist-circumference (*N* = 55), and type 2 diabetes (*N* = 130), such as the *Transcription Factor 7-Like 2* (*TCF7L2*) (Additional file 2: Table S4, risk loci according to the GWAS catalog data accessed 04/2020) [37]. Moreover, we identified 280 genes for SAT and 267 for OVAT which showed differential methylation between the obesity states in a previous work by Keller et al. [10] and were overlapping with genes potentially discriminating between responders and non-responders (Additional file 2: Table S4). Among them, 19 genes in subcutaneous adipose tissue (SAT) and 19 in omental visceral adipose tissue (OVAT) further showed significant transcriptional changes according to differences in metabolic state [10]. GO enrichment analysis unraveled differentially methylated genes between responders and non-responders which annotate to biological processes mainly involved in different types of cell-adhesion (e.g., GO:0007156; homophilic cell adhesion via plasma membrane adhesion molecules; *FDR* = 8.31 × 10^−14^, Additional file 2: Table S5).

### In silico analyses of identified DMRs

Further, we employed a ChromHMM prediction model to functionally annotate the top differentially methylated DMRs to specific tissues most likely relevant for obesity development (e.g., AT derived stem cells) or other metabolically related processes (e.g., skeletal muscle or liver). Data shows *RNF39* and *SLFN12* to be located in an active TSS for AT derived mesenchymal stem cells. While for the other DMRs this seems to be ubiquitous among most tissues, for *RNF39* it is limited to AT (Fig. 3c).

### DNA methylation changes due to specific weight-loss interventions

Based on the findings from responder vs. non-responder analyses, we investigated whether different lifestyle intervention (dietary MED/LC vs LF) and PA had a recognizable impact on CpG methylation in human blood in an expanded analysis including all samples from all interventions together (*N* = 120; T0 vs T18; Additional file 2: Table S6). Thereby we identified 1146 CpG mapping to 1459 genes (84 CpGs with no gene) with a significant methylation change (*q* value, < 0.05). Interestingly, the individual interventions (LF + PA; MED/LC + PA; LF-PA; MED/LC-PA) did not show significant changes on CpG (all *q* value > 0.05) or DMR (all adj. *P* > 0.05) specific DNA methylation levels (Additional file 2: Tables S6–7). Of note, we identified two DMRs on chromosome 1 (2D-KS *P* ≤ 0.015, methylation change between 3.1 and 4.2%) when comparing the different intervention groups between pre- and post-intervention states (see legend to Additional file 2: Table S7). Obviously, these DMRs were also observed in baseline intergroup comparisons. Both DMRs (chr1:564503-565170; chr1:567311-567358) are overlapping and cis to the micro RNA 6723 (miR-6723) and different pseudogenes such as *MTND1P23* and *MTND1P28* (*Mitochondrially Encoded NADH Ubiquinone Oxidoreductase Core Subunit 1 Pseudogene 23/28*) (Additional file 2: Table S7). They further contain the top two differently methylated CpGs when comparing T0 vs. T18 independent from the type of intervention (Additional file 2: Table S6). It has to be acknowledged though that the 2 DMRs include cross-reactive probes and therefore, although the observed differences are most likely bona fide methylation differences, this has to been seen with caution since other processes cannot be fully excluded.

### Epigenome-wide association study uncovers DNA methylation patterns associated with body weight-loss

We tested whether baseline DNA methylation marks correlate with a successful weight-loss by a combined diet and exercise intervention via conducting an epigenome-wide association for the relative difference in body weight after 18 months of the intervention (+/−, relative to initial body weight) on a single base resolution. Thereby, 47 CpG sites corresponding to 41 genes (10 sites with no gene mapping) showed a significant correlation (all combined *P* < 1 × 10^−4^; Table 3). Among them, 15 CpGs correlated negatively and 32 positively with weight change after intervention. The strongest effect, among the CpGs in cis to annotated genes, was found for a CpG site (chr9:128330232) in close proximity to the *MAPK Associated Protein 1* (MAPKAP1) gene locus (*P* = 2.14 × 10^−6^; *r* = 0.42) followed by the *Histocompatibility Minor 13*gene (HM13; *P* = 3.65 × 10^−6^; *r* = 0.41) and an uncharacterized protein (*KIAA0513*; *P* = 9.60 × 10^−6^; *r* = − 0.39). A complete list of CpG loci (*N* = 7776; combined *P* ≤ 0.01) with a correlation of the methylation status prior to intervention and individual’s relative weight-change is given in Additional file 2: Table S8. Among the 7776 CpG sites potentially predicting weight-loss at baseline (combined *P* < 0.01), 506 sites are annotated to genes that are also discriminating between responders and non-responders including 5 genes out of the top loci (*RNF39*, *SLFN12*, *NTSR1*, *LRRC27*, and *LINC00539*).

**Table 3:** Top EWAS hits for weight-change

Location	Combined_***p*** value	Combined_correl	SNPprobes	Croscorr_probes	Genes	Probes/gene
chr9:128330232-128330233	2.13894E−06	0.415656804	0	0	*RP11-12A16.3*, *MAPKAP1*	4.87
chr20:48681452-48681453	2.89151E−06	0.410278561	0	0	*.*	
chr20:30102074-30102075	3.64554E−06	0.40785049	0	0	*HM13*	51
chr8:47108510-47108511	7.25868E−06	0.396511089	0	0	*.*	
chr16:85061578-85061579	9.60171E−06	− 0.390429782	0	0	*KIAA0513*	85
chr4:2860776-2860777	1.03553E−05	0.390219534	0	0	*ADD1*	60
chr1:10676746-10676747	1.10438E−05	0.38897137	1	0	*RN7SL614P*, *PEX14*	6.97
chr21:43995264-43995265	1.53041E-05	0.381354803	0	0	*SLC37A1*	92
chr1:155224863-155224864	1.65133E−05	0.382167212	0	0	*FAM189B*	22
chr14:24611228-24611229	1.66638E−05	0.381131462	0	0	*EMC9*	20
chr6:34312254-34312255	1.85412E−05	− 0.379666826	0	0	*NUDT3*, *RPS10-NUDT3*	40.55
chr3:39766166-39766167	2.68388E−05	− 0.369598821	1	0	*.*	
chr16:32599433-32599434	2.95334E−05	0.36329876	1	1	*.*	
chr14:23832577-23832578	3.0238E−05	− 0.370665738	0	0	*EFS*	19
chr2:43285304-43285305	3.60853E−05	− 0.367590736	0	0	*.*	
chr5:74614787-74614788	3.80965E−05	0.365526726	0	0	*.*	
chr14:19109264-19109265	3.95428E−05	0.365892373	1	1	*RP11-754I20.1*	6
chr10:35027638-35027639	3.97516E−05	0.36532732	0	0	*PARD3*	211
chr6:30009242-30009243	4.04387E−05	0.364775077	0	1	*ZNRD1-AS1*	223
chr1:151941392-151941393	4.22465E−05	0.363467421	1	0	*.*	
chr7:30741951-30741952	4.41082E−05	0.362463879	0	0	*INMT*	39
chr20:58637969-58637970	4.4336E−05	0.362478499	0	0	*C20orf197*	16
chr8:25937278-25937279	4.62614E−05	0.362791545	0	0	*.*	
chr18:76148676-76148677	4.88724E−05	0.361851294	1	0	*.*	
chr12:96710026-96710027	4.89494E−05	0.360939508	0	1	*CDK17*	44
chr20:30947335-30947336	5.34863E−05	− 0.358758835	1	0	*ASXL1*	36
chr11:93884806-93884807	5.40302E−05	0.359319982	0	0	*PANX1*	38
chr12:69982015-69982016	5.77386E−05	0.35850731	0	0	*CCT2*	27
chr12:56837403-56837404	5.92256E−05	0.357960025	1	1	*TIMELESS*	21
chr22:31488064-31488065	5.93091E−05	0.357833796	0	0	*SMTN*	59
chr4:48508755-48508756	5.95427E−05	− 0.357497447	0	0	*FRYL*	74
chr4:186092824-186092825	6.24725E−05	−0.354276473	0	0	*KIAA1430*	40
chr2:232456125-232456126	6.63359E−05	0.354055051	0	0	*C2orf57*	7
chr19:51497512-51497513	6.73546E−05	0.355569746	0	0	*CTB-147C22.9*	59
chr2:202507599-202507600	7.31631E−05	0.35259332	0	0	*TMEM237*	27
chr16:66914802-66914803	8.20107E−05	− 0.351288405	0	0	*PDP2*	27
chr13:114201583-114201584	8.30031E−05	0.350644678	0	0	*TMCO3*	108
chr7:1694822-1694823	8.61102E−05	0.350667814	0	0	*.*	
chr1:84763932-84763933	8.75277E−05	− 0.35001248	0	0	*SAMD13*	37
chr5:86563580-86563581	8.79946E-05	− 0.346433913	0	0	*RASA1*	46
chr11:1220228-1220229	8.93413E−05	− 0.349897083	0	0	*MUC5AC*	49
chr8:117864506-117864507	9.16977E−05	− 0.349138937	0	0	*RAD21*	39
chr5:52929435-52929436	9.18704E−05	0.348561794	0	0	*NDUFS4*	31
chr12:124808992-124808993	9.7238E−05	− 0.348213686	1	0	*NCOR2*	334
chr6:100483904-100483905	9.74908E−05	0.348082518	0	0	*MCHR2-AS1*	23
chr1:110753895-110753896	9.93168E−05	0.343588975	0	0	*KCNC4-AS1*, *KCNC4*	19.47
chr11:117054853-117054854	9.94225E−05	− 0.347463234	0	0	*SIDT2*	34

The table presents the top CpG sites (*P* ≤ 1 × 10^−4^) from combined Spearman and Pearson correlation analysis of genome-wide DNA methylation at baseline with relative changes (+/−) in body weight after intervention. Combined *P* value represents geometric mean of Spearman and Pearson correlation *P* values. SNPprobes represent probes containing a SNP with a frequency > 0.01. Cross-reactive probes indicate potential off-target probe binding. Genes is a comma-separated list of the genes cis to the CpG. Chromosomal location was annotated to genome assembly GRCh37 (hg19)

**Fig. 4:**
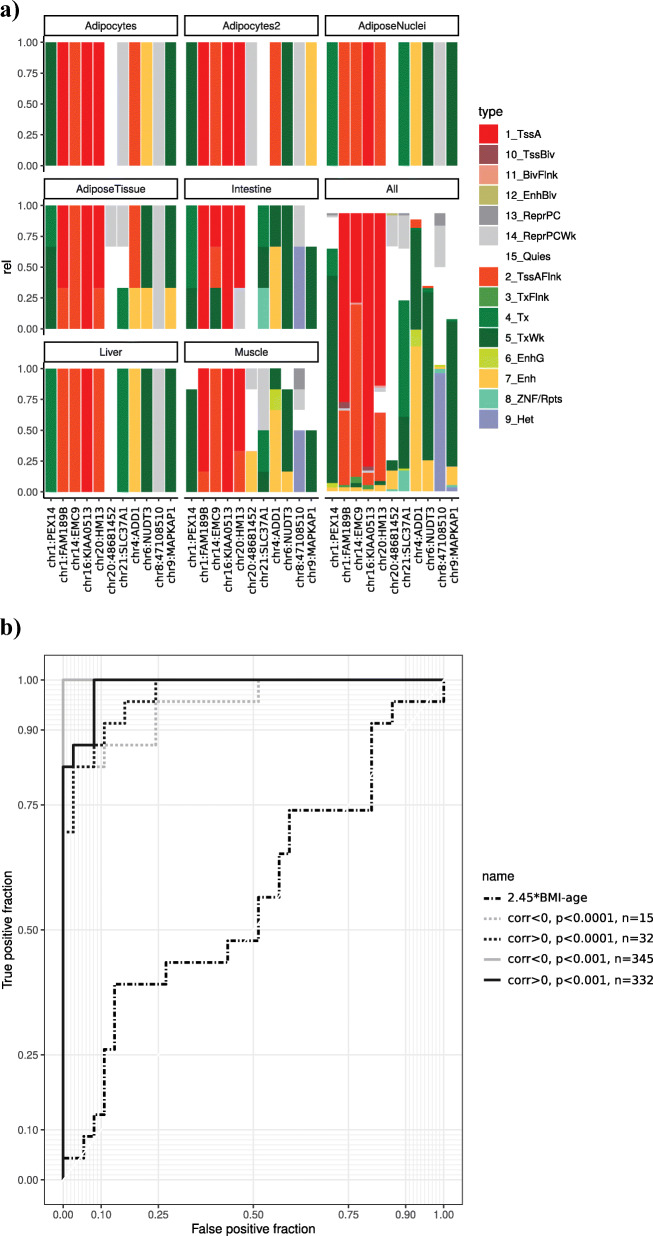
EWAS candidate genes for weight-loss prediction. **a** ChromHMM prediction for all CpG sites which are significantly associated with weight changes during lifestyle intervention (*P* < 1 × 10^–4^).To identify putative target genes in the phenotype relevant target tissues: Intestine (Fetal-Intestine-Large, Fetal-Intestine-Small, Small-Intestines), Adipos-Nuclei (Adipose-Nuclei), Adipose Tissue (Adipose-Nuclei, Adipose-Derived-Mesenchymal-Stem-Cell-Cultured-Cells, Mesenchymal-Stem-Cell-Derived-Adipocyte-Cultured-Cells), Adipocytes (Mesenchymal-Stem-Cell-Derived-Adipocyte-Cultured-Cells), Adipocytes2 (Adipose-Derived-Mesenchymal-Stem-Cell-Cultured-Cells), Liver (Liver), Muscle (HSMM-cell-derived-Skeletal-Muscle-Myotubes-Cells, HSMM-Skeletal-Muscle-Myoblasts-Cells, Skeletal-Muscle-Female, Skeletal-Muscle-Male), and all (all 134 cells from Roadmap Epigenome Gateway). ChromHMM coding for TssA-active TSS, TssBiv-Bivalent (Poised) TSS, BivFlnk-Bivalent Flanking, EnhBiv-Bivalent Enhancer, ReprPC-Polycomb Repressed, ReprPCWk-Weakly Polycomb Repressed, Quies-Quiescent/low, TssAFlnk-Flanking TSS, TxFlnk-Flanking Transcribed, Tx-Transcribed, TxWk-Weakly Transcribed, EnhG- Genetic Enhancer, Enh –Enhancer, ZNF/Rpts-ZNF Genes and Repeats, He-Heterochromatin. **b** Receiver operating characteristic (ROC) curve for successful weight-loss under lifestyle intervention (for all males)

ChromHMM analysis for the top annotated CpGs showed that among the potentially weight-change predicting CpG marks 4 loci are consistent functionally annotated to an active TSS for AT, adipocytes, and adipose nuclei (Fig. 4a) including the top-ranked CpGs for a negative (*KIAA0513*) and positive weight-change (*Histocompatibility Minor 13-HM13*).

### Baseline DNA methylation could predict a successful treatment response

Next, we analyzed whether baseline CpG methylation could also be used to predict intervention success. To do so, we used the receiver operating characteristic (ROC) curve model to potentially predict successful weight-loss. We observed a clear additive value of a baseline DNA methylation score (generated from *ß* values of all CpG sites showing correlation at baseline with weight change) (AUC = 0.95–1.0) compared to known predictors such as baseline age and BMI (AUC = 0.564, Fig. 4b, [26] pages 14–17). However, as we do not have sufficient data to split our data into a training and a test set, this has to be considered with caution and further validation in an independent data set is clearly warranted.

### Correlations between clinical phenotypes and baseline DNA methylation

We further performed correlation analysis for DNA methylation prior to intervention with different parameters of fat distribution (deep subcutaneous (DSC), superficial subcutaneous (SSC) and visceral AT (VAT) area, waist-circumference), glucose homeostasis (plasma-glucose levels, HOMA index), serum adiponectin and leptin levels, BMI and C-reactive protein (CRP) serum levels for all 47 (*P* < 1 × 10^− 4^) identified candidate individual CpG sites coming from baseline EWAS (Additional file 2: Table S9) and 72 CpG from candidate DMRs (*P* adj. < 0.05) (Additional file 2: Table S10). As proof of principle, for the EWAS CpG, we identified mostly correlation to changes in BMI, deep subcutaneous-, superficial subcutaneous-, visceral- adipose tissue, and waist-circumference (Additional file 2: Table S9). Although none of the correlations remained significant after correction for multiple testing, DMR specific correlation analysis revealed a genetic region (672 bp) on chromosome 6 spanning from intron 3 to exon 4 of the *Ring Finger Protein 39* (*RNF39*) which showed positive correlations with HOMA index (all *P* < 0.05, at both time points) and fasting plasma glucose levels (all *P <* 0.03, at both time points) (Additional file 2: Table S10) for 17 overlapping CpGs. In line with this, 16 of those CpG sites annotated to *RNF39* were negatively correlated with adiponectin serum levels and positively with BMI post-intervention (Additional file 2: Table S10). These correlations may warrant further investigation because of their known biological role. Exemplarily, for this previously identified DMR at the *RNF39* locus, which already showed a lower methylation in responders, the ChromHMM predicted underlying active TSS and a bivalent flanking region in adipose tissue-derived stem cells as well as a bivalent flanking and enhancer region for adipose nuclei (Fig. 3c).

Another potentially functional relevant DMR might be the one annotating for the *Long Intergenic Non-Protein Coding RNA 539* (*LINC00539*) which is positively correlated with the change of the deep subcutaneous adipose tissue area as well as the HOMA index change after intervention (all *P* < 0.05, Additional file 2: Table S10). However, this result has to be seen with caution since all three probes among this DMR could be affected by a cross-hybridization to the intergenic region chr6:5831672-5831936.

## Discussion

In the present study, we conducted a genome-wide DNA methylation analysis in blood samples from 120 subjects who underwent the 18-month CENTRAL randomized controlled trial. We demonstrate that the success of lifestyle interventions aimed at reducing weight and improving metabolic health by different dietary (either MED/LC or LF) and exercise (PA+/−) strategies are strongly reflected by specific DNA methylation signature in human blood.

By comparing the genome-wide DNA methylation profiles of 10 top responders and 10 non-responders, we identified 9 DMRs corresponding to 10 individual genes which remained significant even after correction for multiple testing. We further demonstrated that specific DNA methylation patterns prior to intervention are associated with a successful therapy outcome and could thereby along with classical predictors such as age and BMI [7] potentially be used in the future to further specify individuals’ response to lifestyle treatment. We are aware that our prediction analyses were strongly biased by pre-selection of the corresponding sites; thus, the strength of the AUC was not surprising. Also, a validation of our prediction model in vivo would be highly desirable from a statistical point of view, as we lack an independent test set. Unfortunately, given the uniqueness of our cohort and dataset, it is currently not possible. Yet, our data appear robust, even though we could not replicate previously reported findings by Moleres et al. [38], who followed a similar approach in human blood and found 5 CpG sites being differentially methylated between responders and non-responders of a multidisciplinary weight-loss intervention. It has to be acknowledged though that the published data by Moleres et al. was performed in males and females and did not withstand correction for multiple testing, therefore, require further replication and validation [38]. Moreover, whereas 27 k Illumina arrays have been used by Moleres et al., we included 850 k arrays in our study to reach higher genomic coverage. Also, the type of intervention differs between the studies, as only a 10 weeks intervention trial has been conducted by Moleres et al. [38]. Another study performed by Bouchard et al. [39] in human SAT samples, identified 35 CpG sites before a 6-month caloric restriction and 3 CpG sites afterwards being differently methylated between responders and non-responders which correspond to 22 genes. Three out of the 22 genes also light up to be differentially methylated in blood between responders and non-responders of our CENTRAL trial, e.g., the *PRDM8* gene locus (*PR/SET Domain 8*) showing directionally consistent differential methylation. This lack of overlap could be driven by the fact that Bouchard et al. detected individual CpG sites to be differentially methylated between women and our study highlights larger regions being differentially methylated in men. The small overlap indicates that at least for some sites blood DNA methylation might represent a surrogate parameter for changes in obesity relevant target tissues [39]. Furthermore, Bollepalli and colleagues, who studied short-and long-term mRNA expression and DNA methylation changes in SAT using a similar 1-year lifestyle intervention in 19 obese subjects, identified a very similar separation between responders and non-responders after 5 months of intervention. In line with this, our group of responders and non-responders starts to separate in their weight-loss behavior at 6 months of intervention [40]. Additionally, the group identified and replicated mRNA expression changes of *FAM129A* (*Family With Sequence Similarity 129 Member A*) locus correlating with CpG methylation changes after long-term intervention. Since the study design is comparable with our cohort, we could confirm CpG methylation changes (*P* < 1 × 10^−4^, Additional file 2: Table S6, T0 vs T18) in human blood among the LC+/−PA intervention group [40]. Along this line, we proved the overlap with previously published data analyzing methylation differences in human SAT and OVAT between subjects with and without obesity [10]. Although we could not directly compare these gene sets due to the different nature of the studies, we identified 280 genes for SAT and 267 for OVAT which showed differential methylation between the obesity states and were also among our four list of genes potentially discriminating between responders and non-responders (Additional file 2: Table S4). Further, 19 of those genes in SAT and 19 in OVAT also showed a significant mRNA expression change in the data from Keller et al. [10]. This included the *SORBS2* locus manifesting higher methylation and lower expression in OVAT of subjects with obesity, in agreement with our results showing a significantly lower methylation in responders compared to non-responders (data not shown). Furthermore, we have recently shown that an in vitro hyper-methylation of the *SORBS2* promoter which overlaps with the here identified DMR leads to a reduced mRNA expression [10].

Among the identified candidate genes *RNF39* seems to be one of the most promising regions. Besides the clear differences in methylation between responders and non-responders of a lifestyle therapy, the DMR is further associated with parameters of glucose metabolism in our study. This is reasonable since *RNF39* is located in close proximity to the *HLA-J* (*Major Histocompatibility Complex, Class I, J*) locus on chromosome 6p22, a pseudogene of *HLA-A* (*Major Histocompatibility Complex, Class I, A*), and genetic variants in this region have been shown to be associated with insulin resistance in childhood obesity [41] as well as non-obstructive coronary artery disease in women [42]. Furthermore, our results go in line with data from Meeks et al. showing that a DMR of 13 CpG sites in close proximity to *RNF39* is associated with obesity in human blood samples among 547 Ghanaians subjects [43]. The DMR described by Meeks et al. is exactly located within our identified DMR [43].

As recently elaborated by Aronica et al. [44], epigenome-wide association studies of weight are rather rare compared to candidate gene approaches. In particular, changes in body weight following diet and/or exercise-driven interventions are sparsely considered in epigenetic studies. Even the few studies available so far are strongly limited by short intervention times, small sample sizes, or, e.g., restricted to women only [44–47]. Further, it has to be acknowledged that many of the prior studies published on similar subjects did not analyze immune cell subtypes; thus, it cannot be excluded that some of the differences they found are a function of immune cell differences. Therefore, inconsistencies in reported findings might be expected making a comparison of the different studies quite problematic. It is of note, however, that according to the Houseman method in our study, none of the cell type populations/cell compositions changed significantly after the intervention. In general, it is noteworthy that our data is in line with other studies on DNA methylation changes which overall reported only small changes (≤ 5%) on genome-wide DNA methylation when comparing pre- and post-interventional DNA methylation status [44].

We are aware of several limitations of our investigation. First, given the specific nature of the intervention workplace, the number of women in our study is strongly limited. Further, tissue-specificity of the epigenome is one of the major concerns in epigenetic epidemiology. Whole blood is the most frequently used biological material in genetic and epigenetic studies, since for the majority of studies, it is often the only source available. Since we were lacking a cohort to validate our identified candidate genes in relevant target tissues such as adipose tissue, we used previously published data [10] to indirectly check the potential role of these genes in the pathophysiology of obesity. We have to note that blood and adipose datasets were completely independent (i.e., not the same individuals), and therefore, any conclusions drawn in terms of validation of the original observations are treated with caution. Nevertheless, the observed overlaps with previously published data in AT [10, 39] may indicate that blood methylation marks indeed may have the potential in reflecting changes in corresponding target tissues. We have to acknowledge, though, that similar results in methylation studies of adipose and blood may also simply reflect the leukocyte differential invasion and activation in these organs. Regrettably, we did not have access to the respective biological material allowing to prove whether the subtypes of immune cells that are reflected in the results from the EWAS conducted in blood happened to be the subtype infiltrating the adipose tissue in different obesity subtypes. Moreover, we have to acknowledge that although cell type composition was not significantly changed by the individual lifestyle interventions in our study, this may have been driven by the limited statistical power due to our relatively small sample size.

However, these findings are of relevance, since they further support similar studies [17] suggesting that assessment of DNA methylation in blood samples might be a powerful tool to identify variation in DNA methylation related to obesity and/or particularly to body weight change. Despite the huge potential of whole blood as a source of biomaterial, its cellular heterogeneity is a big challenge in epigenetic analyses. The variation in DNA methylation patterns caused by the different cell type compositions may represent a strong confounding factor, which has to be accounted for. In our study, we used a statistical approach to infer cellular distribution from epigenomic data. Finally, an important concern for any association study is to clarify the causative chains behind the recorded statistical relationships. Given the lack of additional data such as genome-wide genotypes (e.g., SNPs), we were not able to address the causality by employing Mendelian randomization or similar mediation analyses. Nevertheless, our findings are sufficiently robust and informative for identified genes to be considered as prognostic biomarkers, e.g., DNA methylation *RNF39* might contribute to the prediction of a successful weight-loss therapy.

## Conclusion

In conclusion, our findings suggest that biological processes such as cell adhesion or molecular functions such as calcium ion binding could have an important role in determining the success of interventional therapies in obesity. Moreover, methylation differences in the identified genes could serve as prognostic biomarkers to predict a successful weight-loss therapy and thus contribute to advances in patient-tailored obesity treatment.

## Supplementary Information

**Additional file 1.** Detailed quality report of the Illumina EPIC 850 K raw data.

**Additional file 2: Supplementary Tables S1-S10**. **Table S1**. Full list of discriminating DMRs (responder vs. non-responder) - T0. **Table S2**. Full list of discriminating DMRs (responder vs. non-responder) - T18. **Table S3**. Full list of discriminating DMRs (responder vs. non-responder) – from combined datasets T0 and T18. **Table S4**. Candidate genes from GWAS and methylation differences in adipose tissue. **Table S5**. Gene ontology enrichment analysis. **Table S6**. Full list of intervention specific CpGs. **Table S7**. Full list of intervention specific DMRs. **Table S8**. Full list of EWAS CpGs for weight-change. **Table S9**. Phenotype associations for CpGs from baseline EWAS. **Table S10**. Phenotype associations for CpGs from DMRs (responder vs. non-responder).

## Data Availability

All data generated or analyzed during this study are included in this published article and its Additional files. Raw data is available in the ArrayExpress [23] repository, https://www.ebi.ac.uk/arrayexpress/experiments/E-MTAB-8956. The complete data processing code can be obtained from github https://github.com/Bierinformatik/CENTRALEPIC [26].
