# Supplementary material for: DNA methylation signature in blood mirrors successful weight-loss during lifestyle interventions: the CENTRAL trial
Source: Genome Med. 2020 Nov 16;12:97. doi: 10.1186/s13073-020-00794-7 (PMC7670623; doi:10.1186/s13073-020-00794-7)
Supplement: Supplementary file 1 — Additional file 1. Detailed quality report of the Illumina EPIC 850 K raw data. [file 13073_2020_794_MOESM1_ESM.pdf]

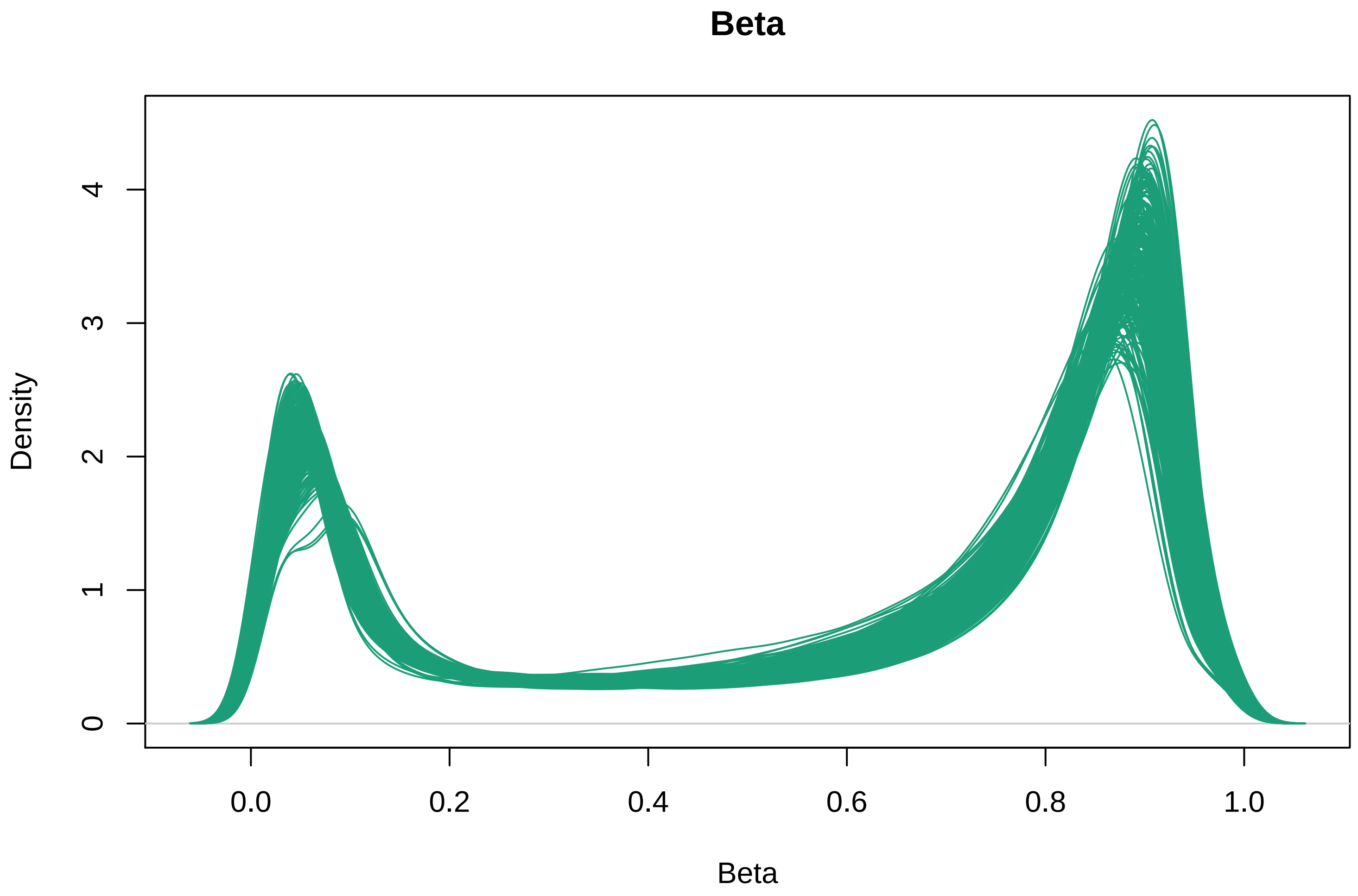

Beta
Density
0
1
2
3
4
0.0
0.2
0.4
0.6
0.8
1.0
Beta

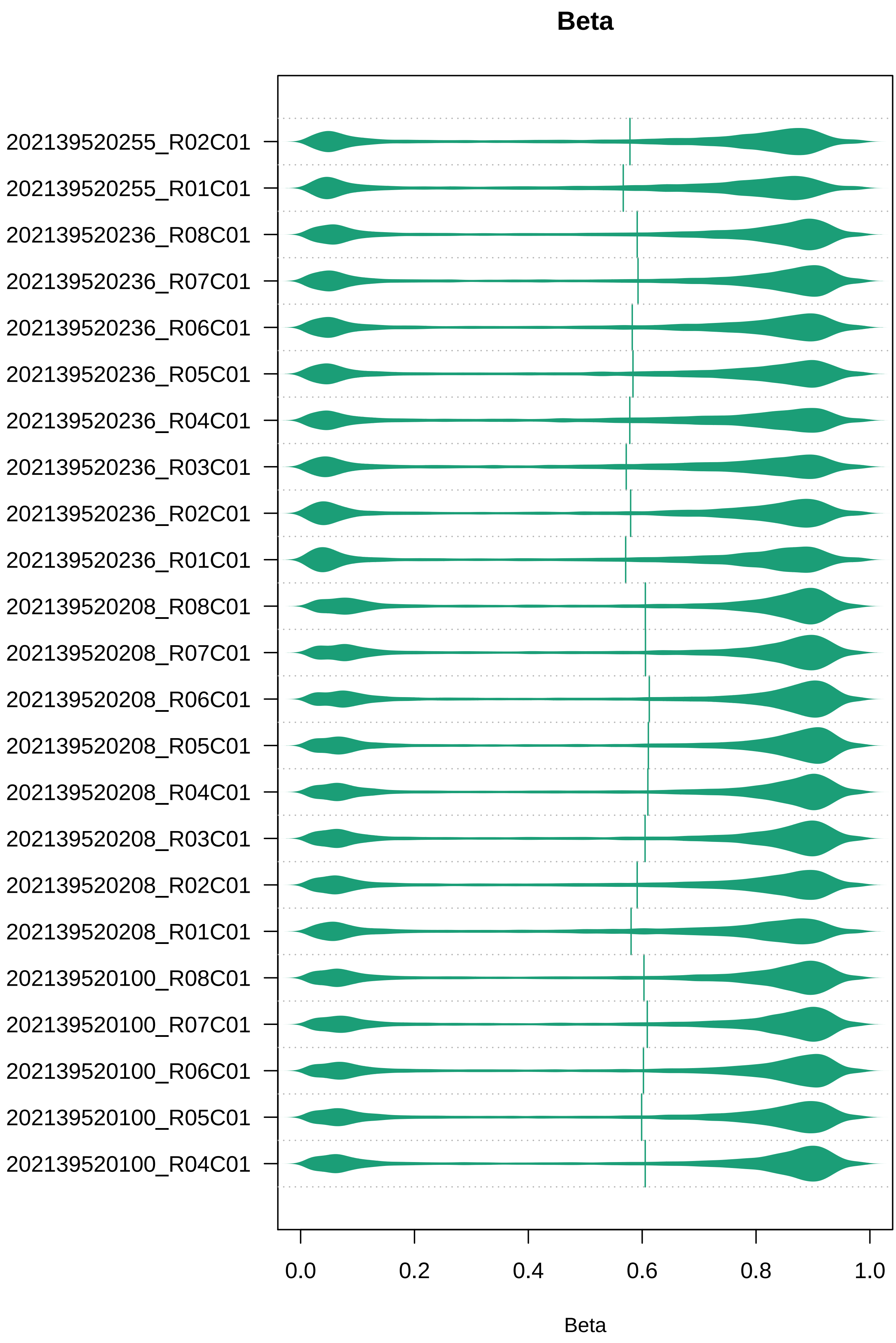
Beta
202139520255_R02C01
202139520255_R01C01
202139520236_R08C01
202139520236_R07C01
202139520236_R06C01
202139520236_R05C01
202139520236_R04C01
202139520236_R03C01
202139520236_R02C01
202139520236_R01C01
202139520208_R08C01
202139520208_R07C01
202139520208_R06C01
202139520208_R05C01
202139520208_R04C01
202139520208_R03C01
202139520208_R02C01
202139520208_R01C01
202139520100_R08C01
202139520100_R07C01
202139520100_R06C01
202139520100_R05C01
202139520100_R04C01
0.0
0.2
0.4
0.6
0.8
1.0
Beta

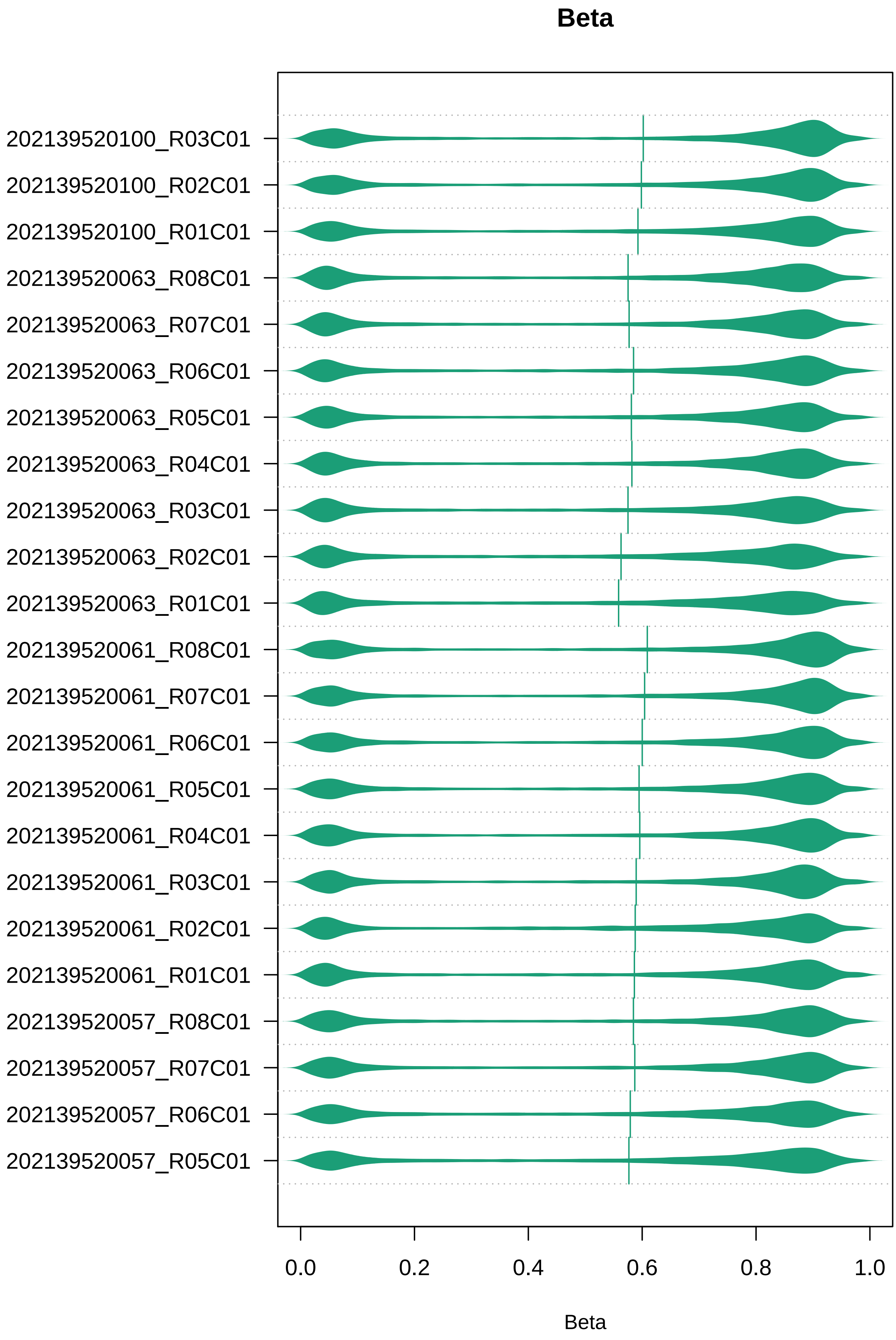

Beta
202139520100_R03C01
202139520100_R02C01
202139520100_R01C01
202139520063_R08C01
202139520063_R07C01
202139520063_R06C01
202139520063_R05C01
202139520063_R04C01
202139520063_R03C01
202139520063_R02C01
202139520063_R01C01
202139520061_R08C01
202139520061_R07C01
202139520061_R06C01
202139520061_R05C01
202139520061_R04C01
202139520061_R03C01
202139520061_R02C01
202139520061_R01C01
202139520057_R08C01
202139520057_R07C01
202139520057_R06C01
202139520057_R05C01
0.0
0.2
0.4
0.6
0.8
1.0
Beta

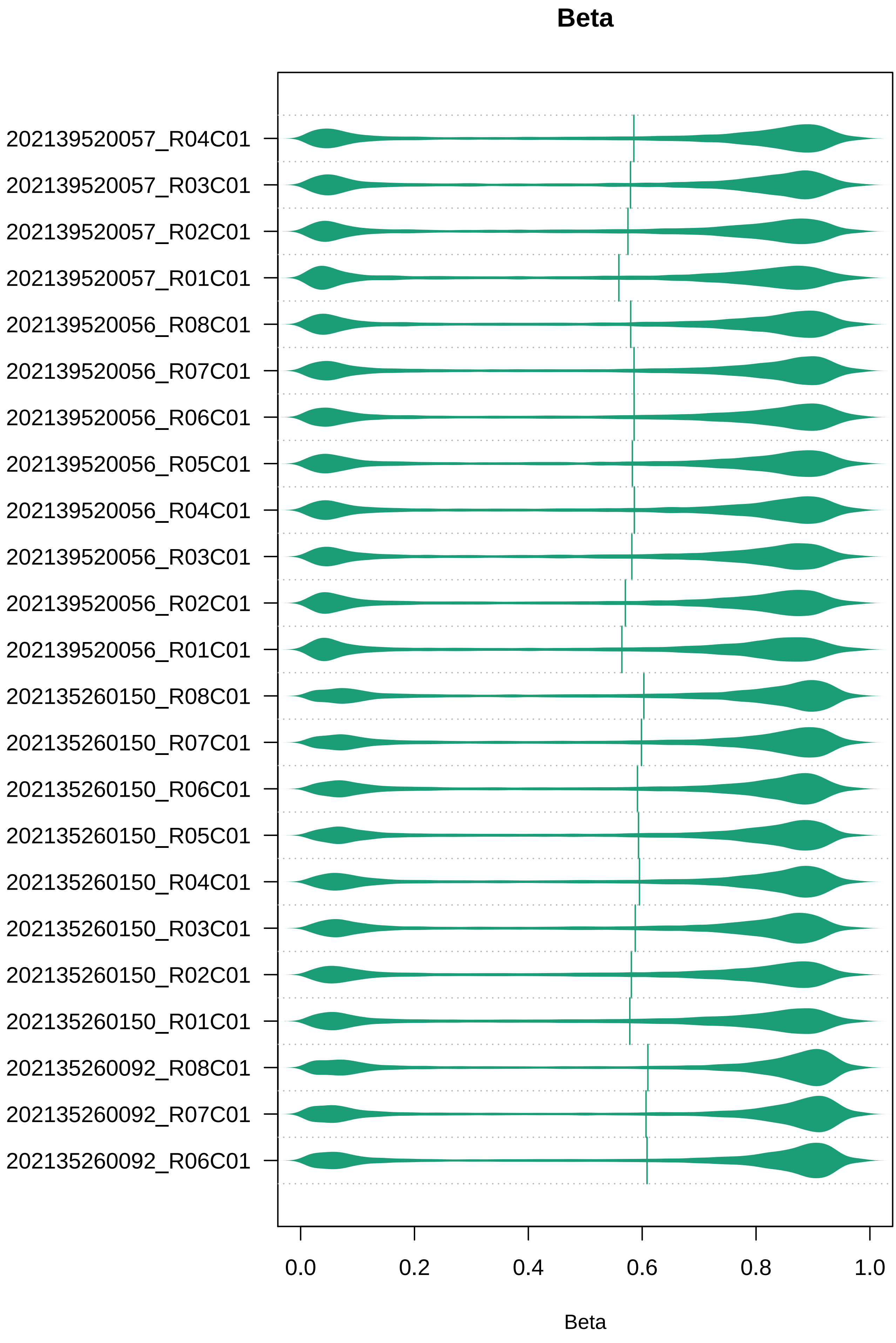
Beta
202139520057_R04C01
202139520057_R03C01
202139520057_R02C01
202139520057_R01C01
202139520056_R08C01
202139520056_R07C01
202139520056_R06C01
202139520056_R05C01
202139520056_R04C01
202139520056_R03C01
202139520056_R02C01
202139520056_R01C01
202135260150_R08C01
202135260150_R07C01
202135260150_R06C01
202135260150_R05C01
202135260150_R04C01
202135260150_R03C01
202135260150_R02C01
202135260150_R01C01
202135260092_R08C01
202135260092_R07C01
202135260092_R06C01
0.0
0.2
0.4
0.6
0.8
1.0
Beta

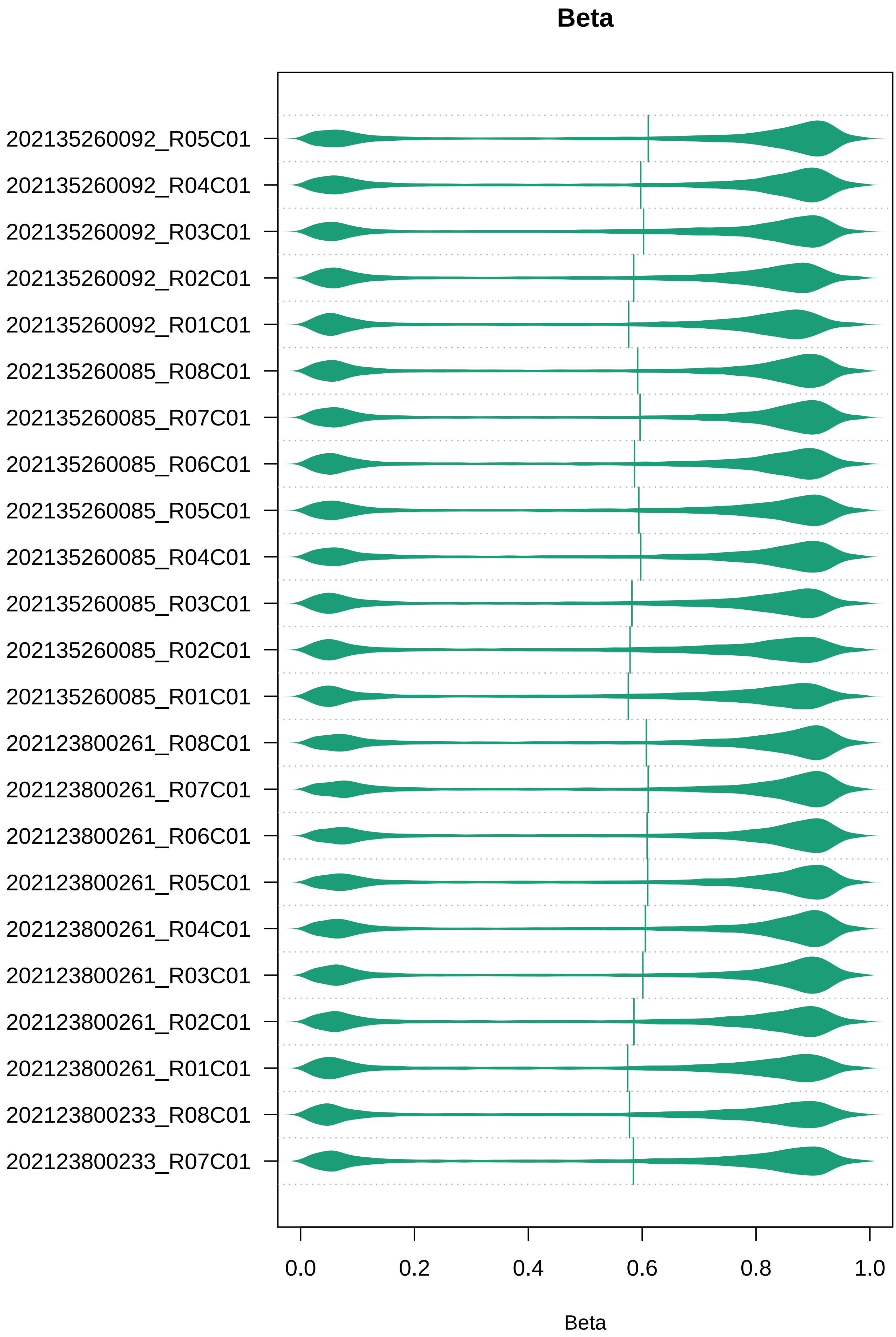
Beta
202135260092_R05C01
202135260092_R04C01
202135260092_R03C01
202135260092_R02C01
202135260092_R01C01
202135260085_R08C01
202135260085_R07C01
202135260085_R06C01
202135260085_R05C01
202135260085_R04C01
202135260085_R03C01
202135260085_R02C01
202135260085_R01C01
202123800261_R08C01
202123800261_R07C01
202123800261_R06C01
202123800261_R05C01
202123800261_R04C01
202123800261_R03C01
202123800261_R02C01
202123800261_R01C01
202123800233_R08C01
202123800233_R07C01
0.0
0.2
0.4
0.6
0.8
1.0
Beta

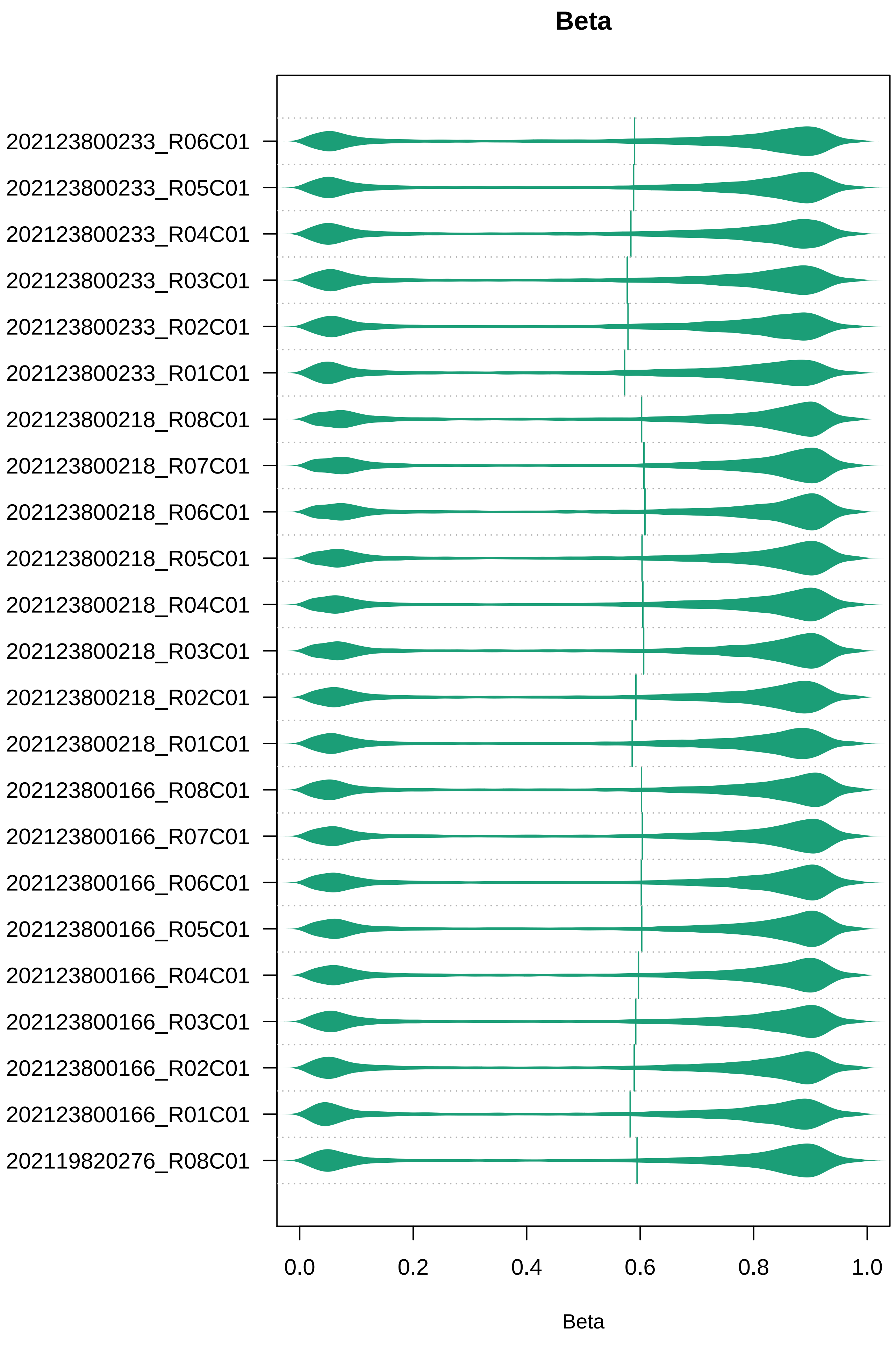
Beta
202123800233_R06C01
202123800233_R05C01
202123800233_R04C01
202123800233_R03C01
202123800233_R02C01
202123800233_R01C01
202123800218_R08C01
202123800218_R07C01
202123800218_R06C01
202123800218_R05C01
202123800218_R04C01
202123800218_R03C01
202123800218_R02C01
202123800218_R01C01
202123800166_R08C01
202123800166_R07C01
202123800166_R06C01
202123800166_R05C01
202123800166_R04C01
202123800166_R03C01
202123800166_R02C01
202123800166_R01C01
202119820276_R08C01
0.0
0.2
0.4
0.6
0.8
1.0
Beta

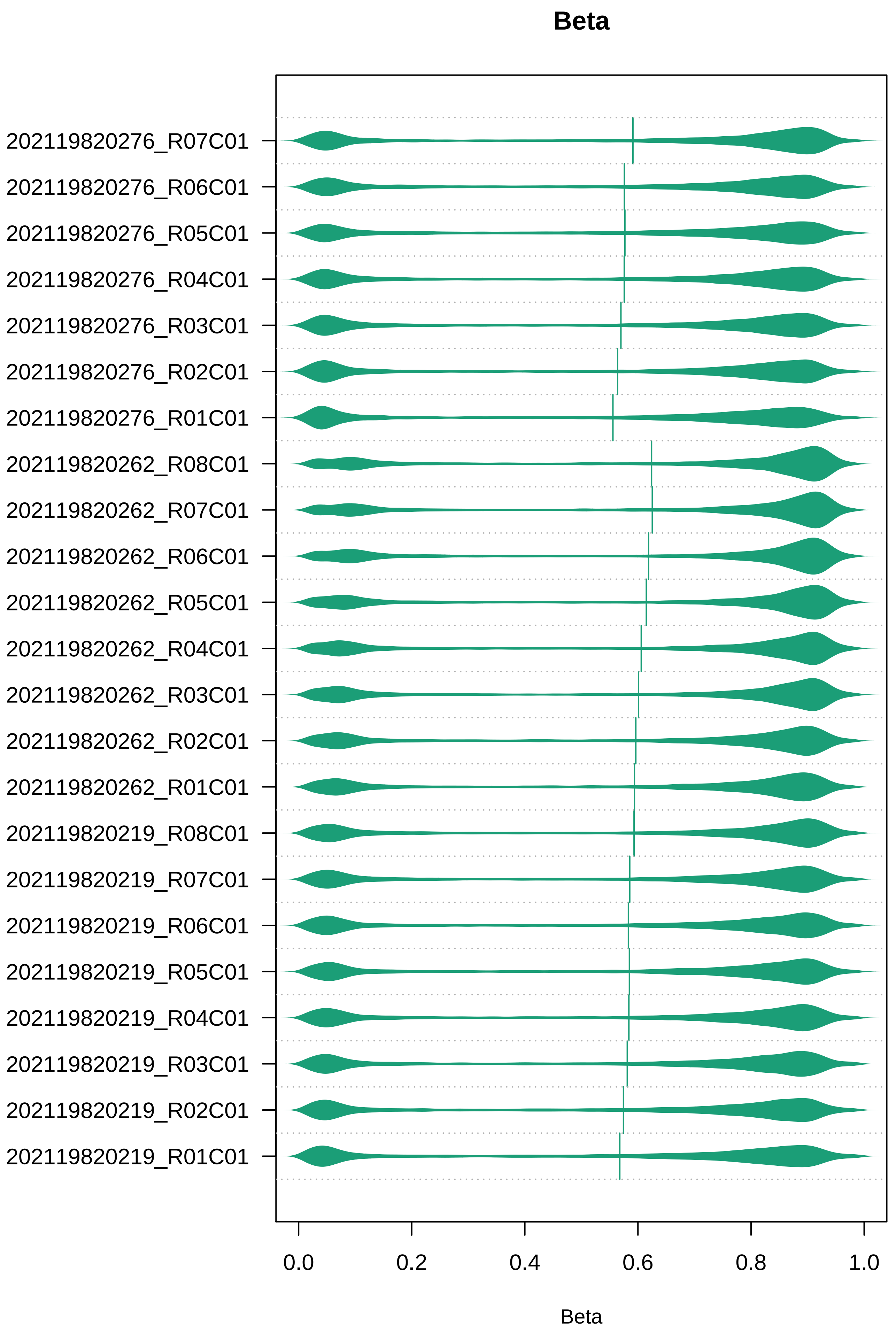

Beta
202119820276_R07C01
202119820276_R06C01
202119820276_R05C01
202119820276_R04C01
202119820276_R03C01
202119820276_R02C01
202119820276_R01C01
202119820262_R08C01
202119820262_R07C01
202119820262_R06C01
202119820262_R05C01
202119820262_R04C01
202119820262_R03C01
202119820262_R02C01
202119820262_R01C01
202119820219_R08C01
202119820219_R07C01
202119820219_R06C01
202119820219_R05C01
202119820219_R04C01
202119820219_R03C01
202119820219_R02C01
202119820219_R01C01
0.0
0.2
0.4
0.6
0.8
1.0
Beta

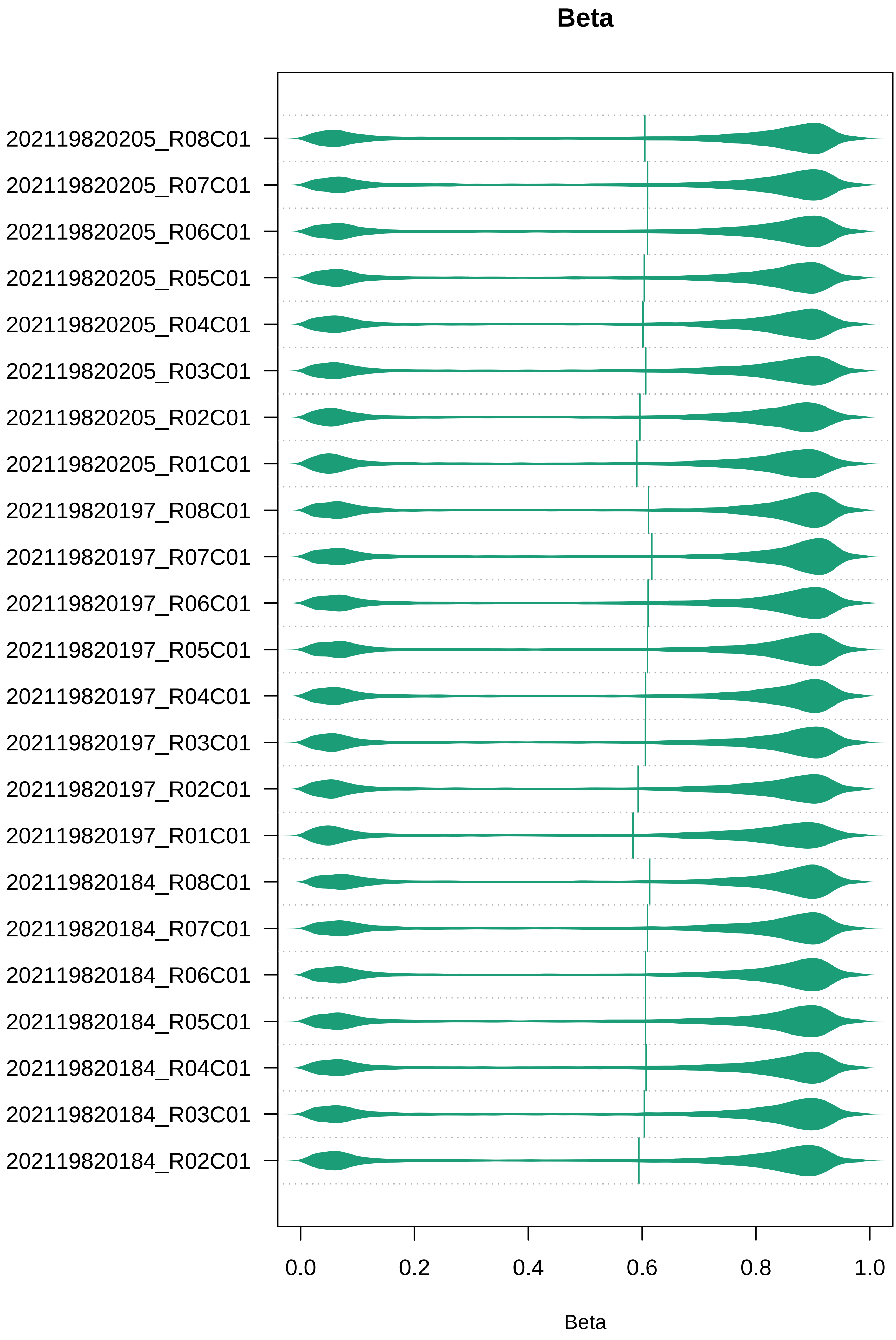

Beta
202119820205_R08C01
202119820205_R07C01
202119820205_R06C01
202119820205_R05C01
202119820205_R04C01
202119820205_R03C01
202119820205_R02C01
202119820205_R01C01
202119820197_R08C01
202119820197_R07C01
202119820197_R06C01
202119820197_R05C01
202119820197_R04C01
202119820197_R03C01
202119820197_R02C01
202119820197_R01C01
202119820184_R08C01
202119820184_R07C01
202119820184_R06C01
202119820184_R05C01
202119820184_R04C01
202119820184_R03C01
202119820184_R02C01
0.0
0.2
0.4
0.6
0.8
1.0
Beta

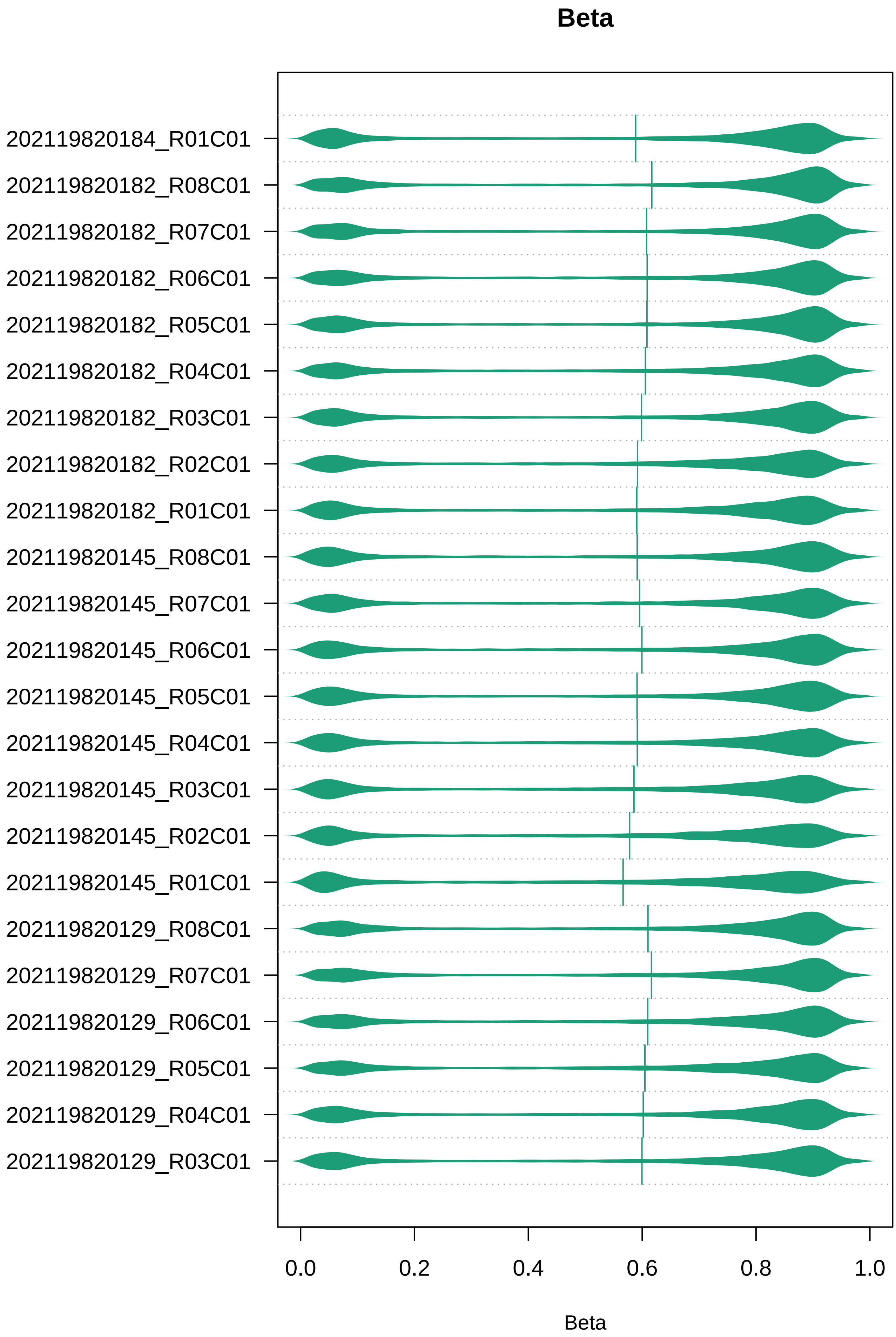

Beta
202119820184_R01C01
202119820182_R08C01
202119820182_R07C01
202119820182_R06C01
202119820182_R05C01
202119820182_R04C01
202119820182_R03C01
202119820182_R02C01
202119820182_R01C01
202119820145_R08C01
202119820145_R07C01
202119820145_R06C01
202119820145_R05C01
202119820145_R04C01
202119820145_R03C01
202119820145_R02C01
202119820145_R01C01
202119820129_R08C01
202119820129_R07C01
202119820129_R06C01
202119820129_R05C01
202119820129_R04C01
202119820129_R03C01
0.0
0.2
0.4
0.6
0.8
1.0
Beta

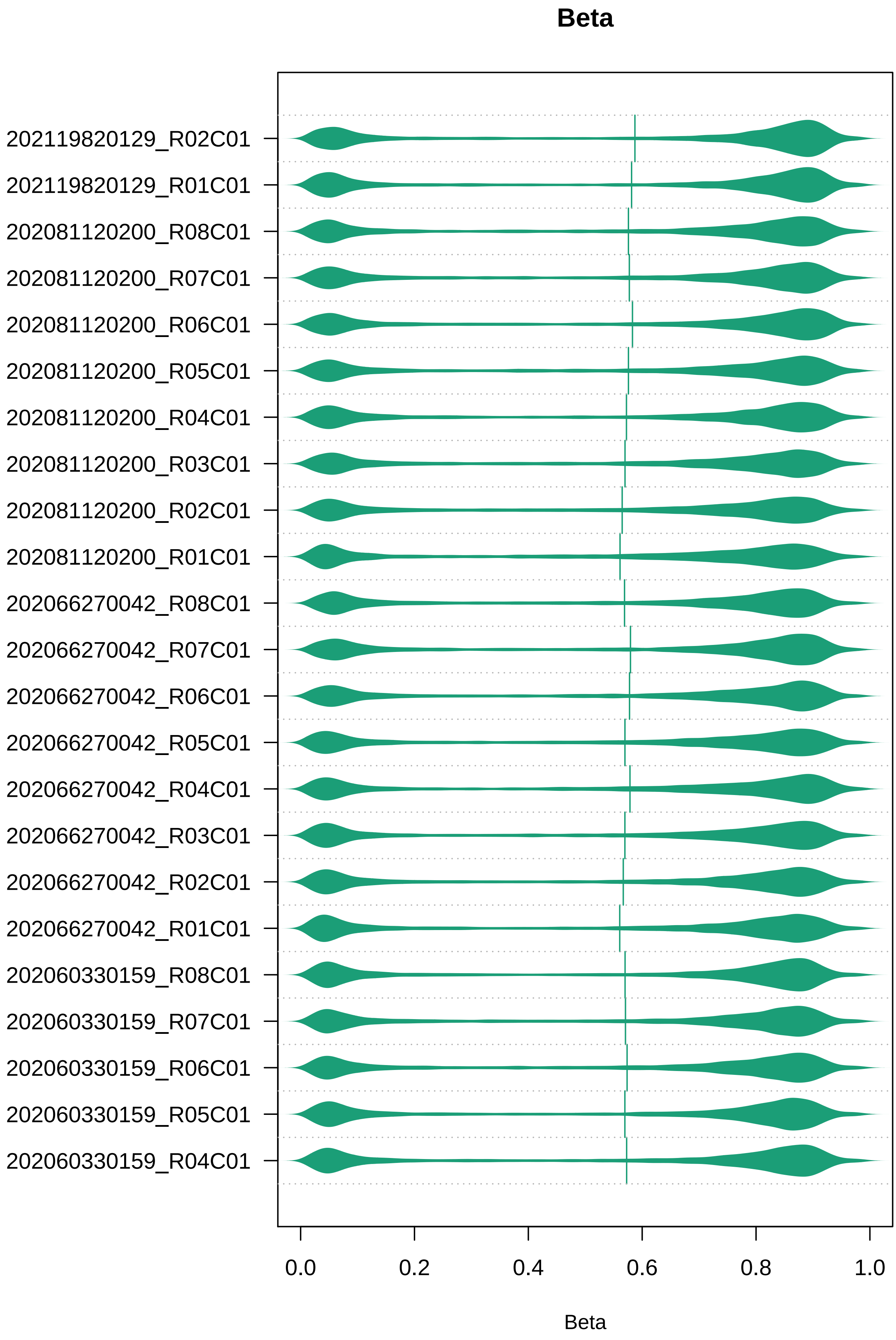

Beta
202119820129_R02C01
202119820129_R01C01
202081120200_R08C01
202081120200_R07C01
202081120200_R06C01
202081120200_R05C01
202081120200_R04C01
202081120200_R03C01
202081120200_R02C01
202081120200_R01C01
202066270042_R08C01
202066270042_R07C01
202066270042_R06C01
202066270042_R05C01
202066270042_R04C01
202066270042_R03C01
202066270042_R02C01
202066270042_R01C01
202060330159_R08C01
202060330159_R07C01
202060330159_R06C01
202060330159_R05C01
202060330159_R04C01
0.0
0.2
0.4
0.6
0.8
1.0
Beta

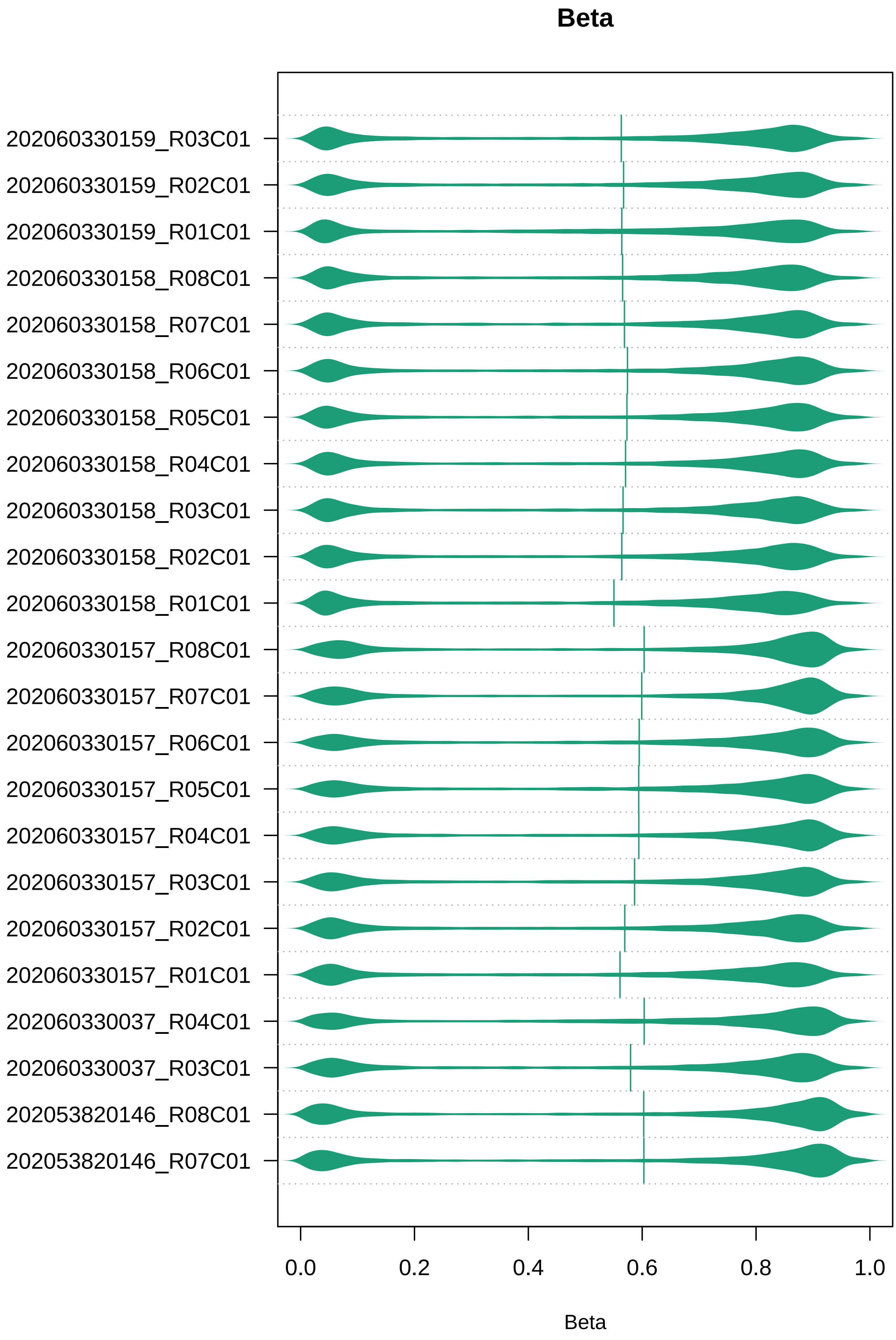

Beta
202060330159_R03C01
202060330159_R02C01
202060330159_R01C01
202060330158_R08C01
202060330158_R07C01
202060330158_R06C01
202060330158_R05C01
202060330158_R04C01
202060330158_R03C01
202060330158_R02C01
202060330158_R01C01
202060330157_R08C01
202060330157_R07C01
202060330157_R06C01
202060330157_R05C01
202060330157_R04C01
202060330157_R03C01
202060330157_R02C01
202060330157_R01C01
202060330037_R04C01
202060330037_R03C01
202053820146_R08C01
202053820146_R07C01
0.0
0.2
0.4
0.6
0.8
1.0
Beta

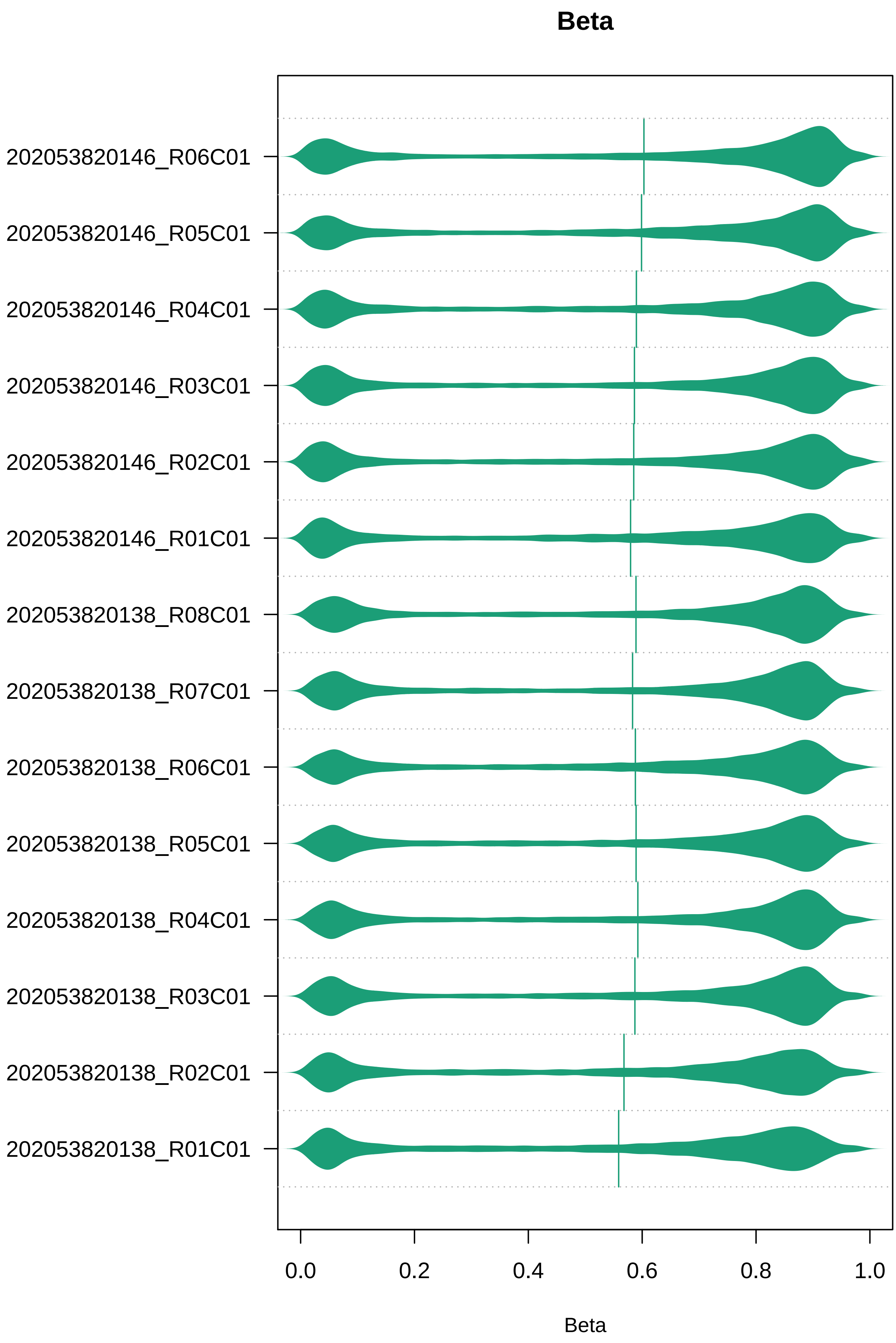

Beta
202053820146_R06C01
202053820146_R05C01
202053820146_R04C01
202053820146_R03C01
202053820146_R02C01
202053820146_R01C01
202053820138_R08C01
202053820138_R07C01
202053820138_R06C01
202053820138_R05C01
202053820138_R04C01
202053820138_R03C01
202053820138_R02C01
202053820138_R01C01
0.0
0.2
0.4
0.6
0.8
1.0
Beta

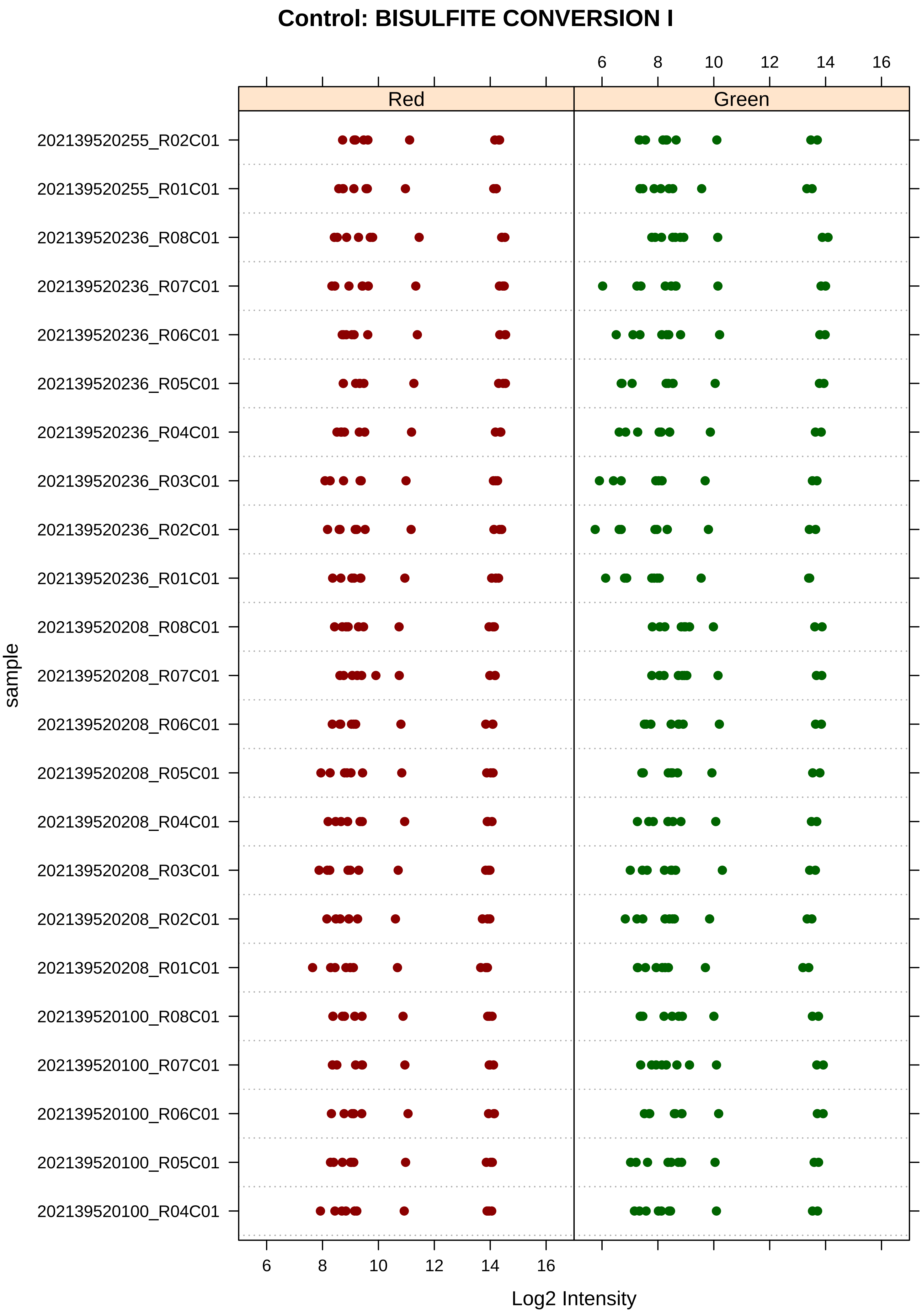

Control: BISULFITE CONVERSION I
Red
Green
sample
Log2 Intensity
202139520255_R02C01
202139520255_R01C01
202139520236_R08C01
202139520236_R07C01
202139520236_R06C01
202139520236_R05C01
202139520236_R04C01
202139520236_R03C01
202139520236_R02C01
202139520236_R01C01
202139520208_R08C01
202139520208_R07C01
202139520208_R06C01
202139520208_R05C01
202139520208_R04C01
202139520208_R03C01
202139520208_R02C01
202139520208_R01C01
202139520100_R08C01
202139520100_R07C01
202139520100_R06C01
202139520100_R05C01
202139520100_R04C01
6
8
10
12
14
16

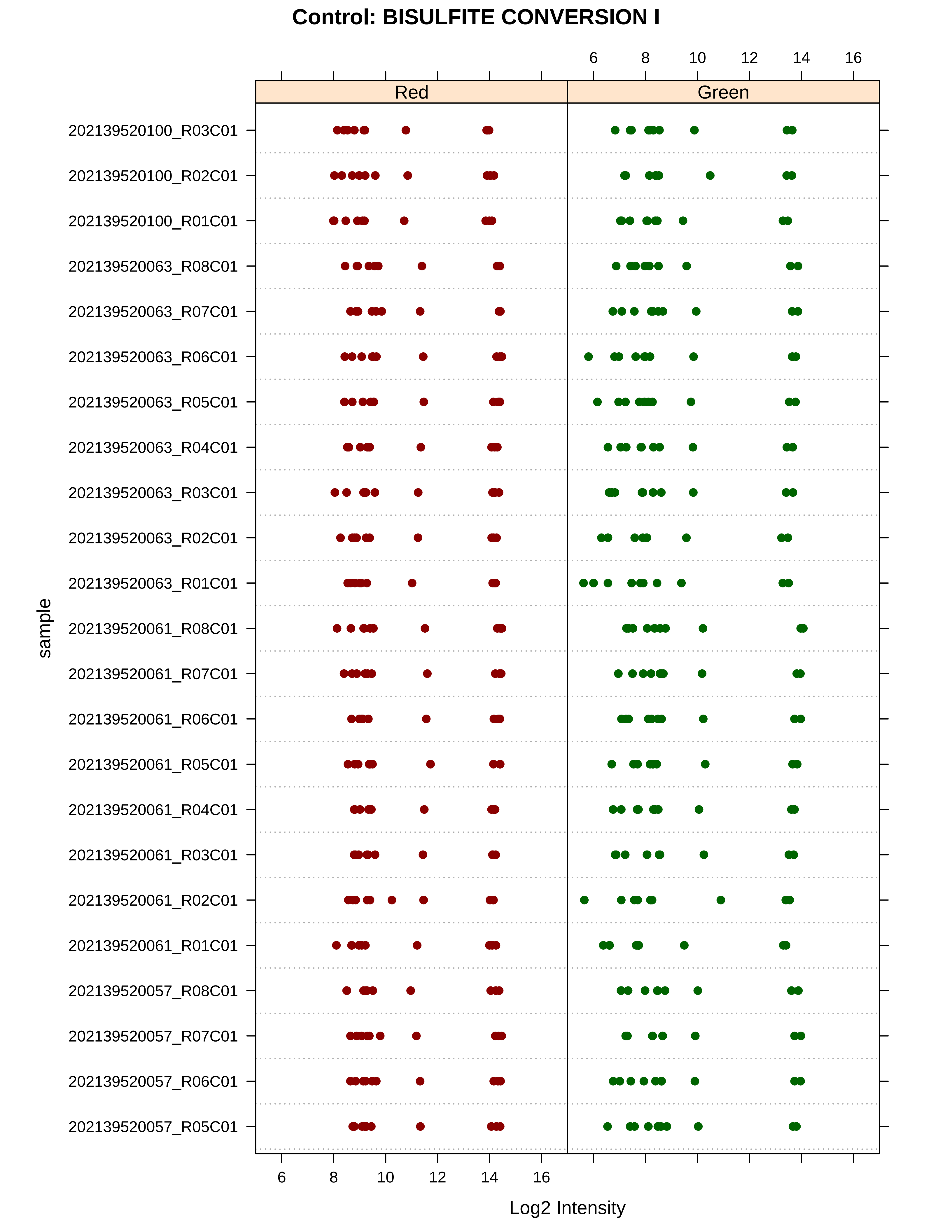

Control: BISULFITE CONVERSION I
Red
Green
sample
Log2 Intensity
202139520100_R03C01
202139520100_R02C01
202139520100_R01C01
202139520063_R08C01
202139520063_R07C01
202139520063_R06C01
202139520063_R05C01
202139520063_R04C01
202139520063_R03C01
202139520063_R02C01
202139520063_R01C01
202139520061_R08C01
202139520061_R07C01
202139520061_R06C01
202139520061_R05C01
202139520061_R04C01
202139520061_R03C01
202139520061_R02C01
202139520061_R01C01
202139520057_R08C01
202139520057_R07C01
202139520057_R06C01
202139520057_R05C01
6
8
10
12
14
16

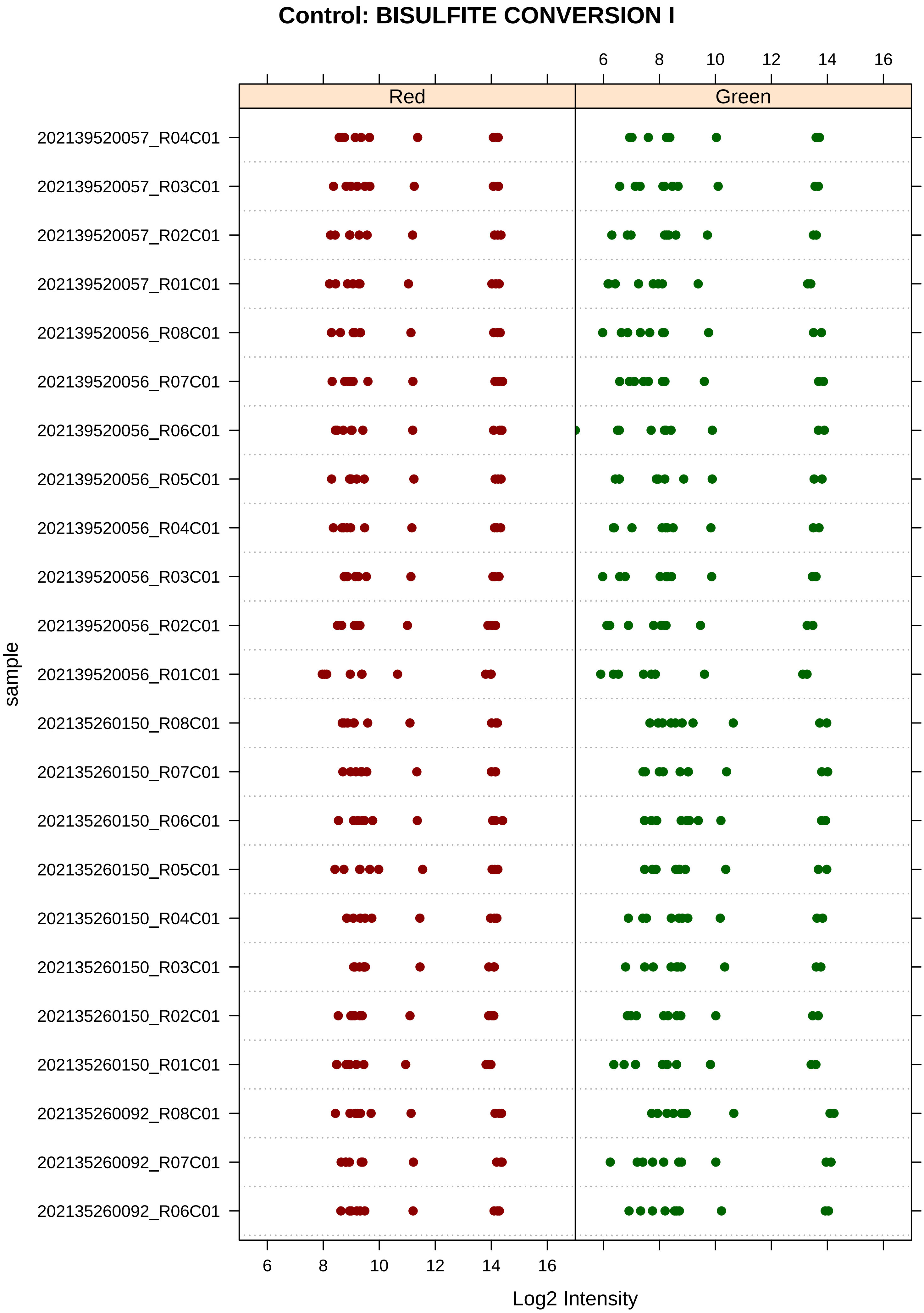
Control: BISULFITE CONVERSION I
Red
Green
sample
Log2 Intensity
202139520057_R04C01
202139520057_R03C01
202139520057_R02C01
202139520057_R01C01
202139520056_R08C01
202139520056_R07C01
202139520056_R06C01
202139520056_R05C01
202139520056_R04C01
202139520056_R03C01
202139520056_R02C01
202139520056_R01C01
202135260150_R08C01
202135260150_R07C01
202135260150_R06C01
202135260150_R05C01
202135260150_R04C01
202135260150_R03C01
202135260150_R02C01
202135260150_R01C01
202135260092_R08C01
202135260092_R07C01
202135260092_R06C01
6
8
10
12
14
16

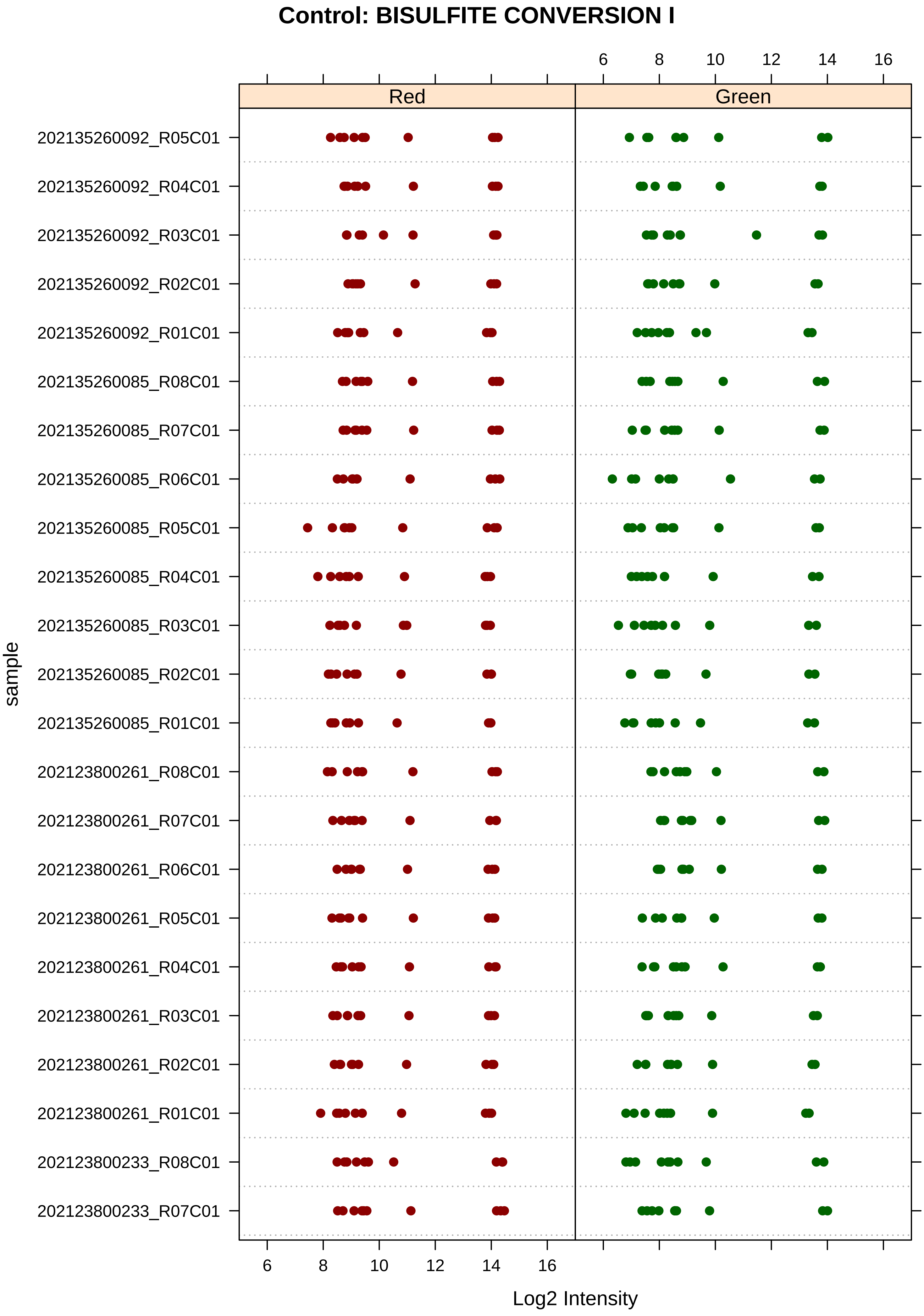
Control: BISULFITE CONVERSION I
Red
Green
sample
Log2 Intensity
202135260092_R05C01
202135260092_R04C01
202135260092_R03C01
202135260092_R02C01
202135260092_R01C01
202135260085_R08C01
202135260085_R07C01
202135260085_R06C01
202135260085_R05C01
202135260085_R04C01
202135260085_R03C01
202135260085_R02C01
202135260085_R01C01
202123800261_R08C01
202123800261_R07C01
202123800261_R06C01
202123800261_R05C01
202123800261_R04C01
202123800261_R03C01
202123800261_R02C01
202123800261_R01C01
202123800233_R08C01
202123800233_R07C01
6
8
10
12
14
16

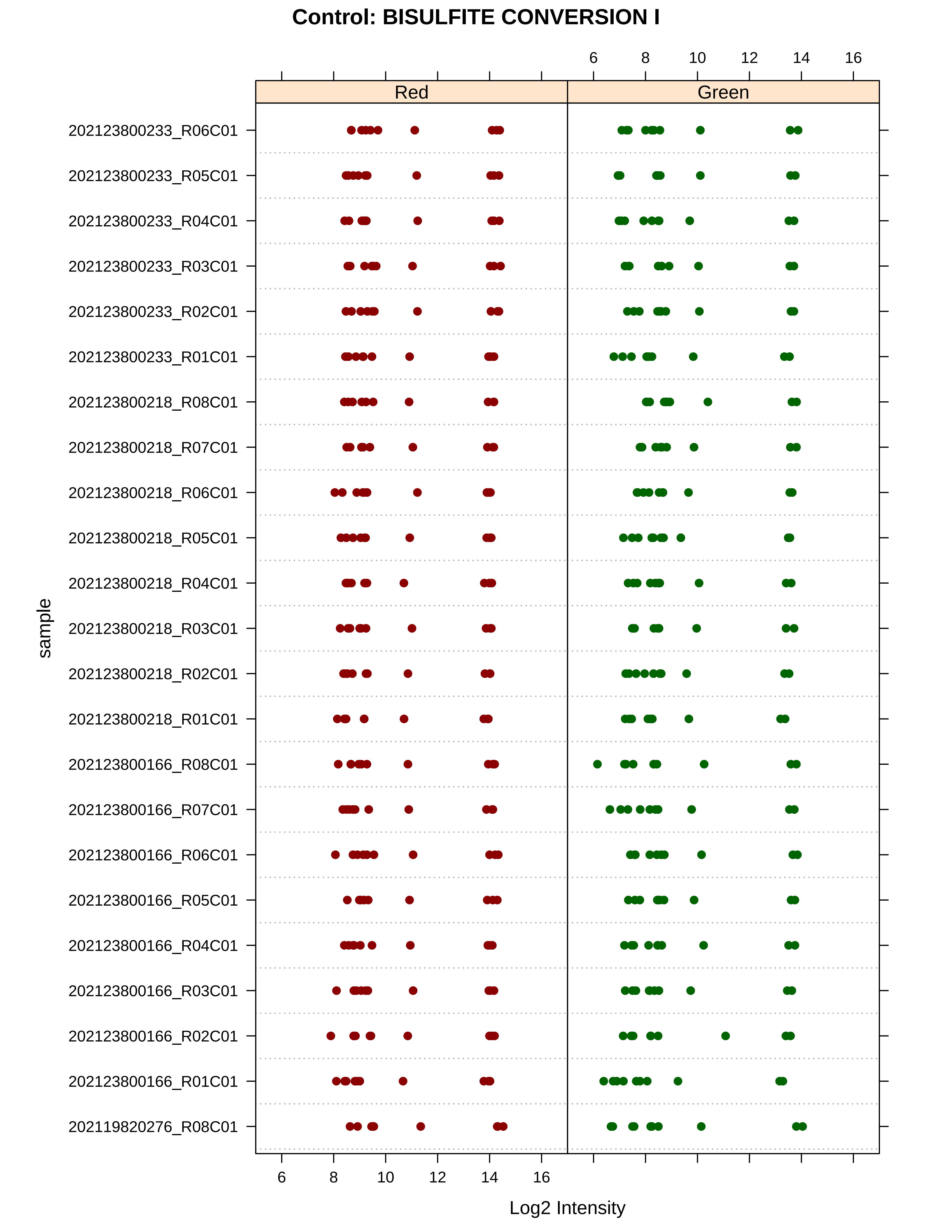
Control: BISULFITE CONVERSION I
Red
Green
sample
Log2 Intensity
6
8
10
12
14
16
202123800233_R06C01
202123800233_R05C01
202123800233_R04C01
202123800233_R03C01
202123800233_R02C01
202123800233_R01C01
202123800218_R08C01
202123800218_R07C01
202123800218_R06C01
202123800218_R05C01
202123800218_R04C01
202123800218_R03C01
202123800218_R02C01
202123800218_R01C01
202123800166_R08C01
202123800166_R07C01
202123800166_R06C01
202123800166_R05C01
202123800166_R04C01
202123800166_R03C01
202123800166_R02C01
202123800166_R01C01
202119820276_R08C01

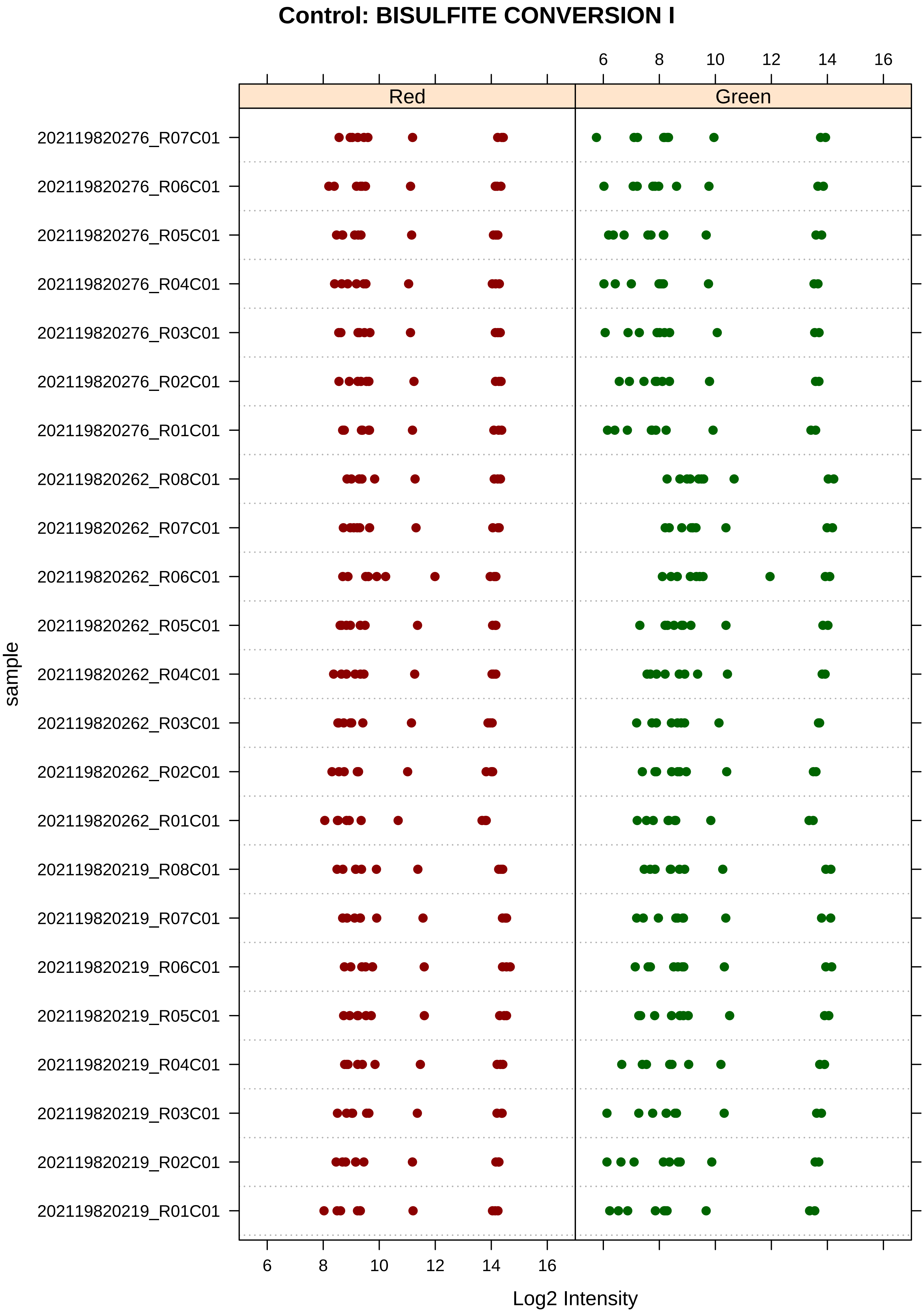
Control: BISULFITE CONVERSION I
Red
Green
sample
Log2 Intensity
202119820276_R07C01
202119820276_R06C01
202119820276_R05C01
202119820276_R04C01
202119820276_R03C01
202119820276_R02C01
202119820276_R01C01
202119820262_R08C01
202119820262_R07C01
202119820262_R06C01
202119820262_R05C01
202119820262_R04C01
202119820262_R03C01
202119820262_R02C01
202119820262_R01C01
202119820219_R08C01
202119820219_R07C01
202119820219_R06C01
202119820219_R05C01
202119820219_R04C01
202119820219_R03C01
202119820219_R02C01
202119820219_R01C01
6
8
10
12
14
16

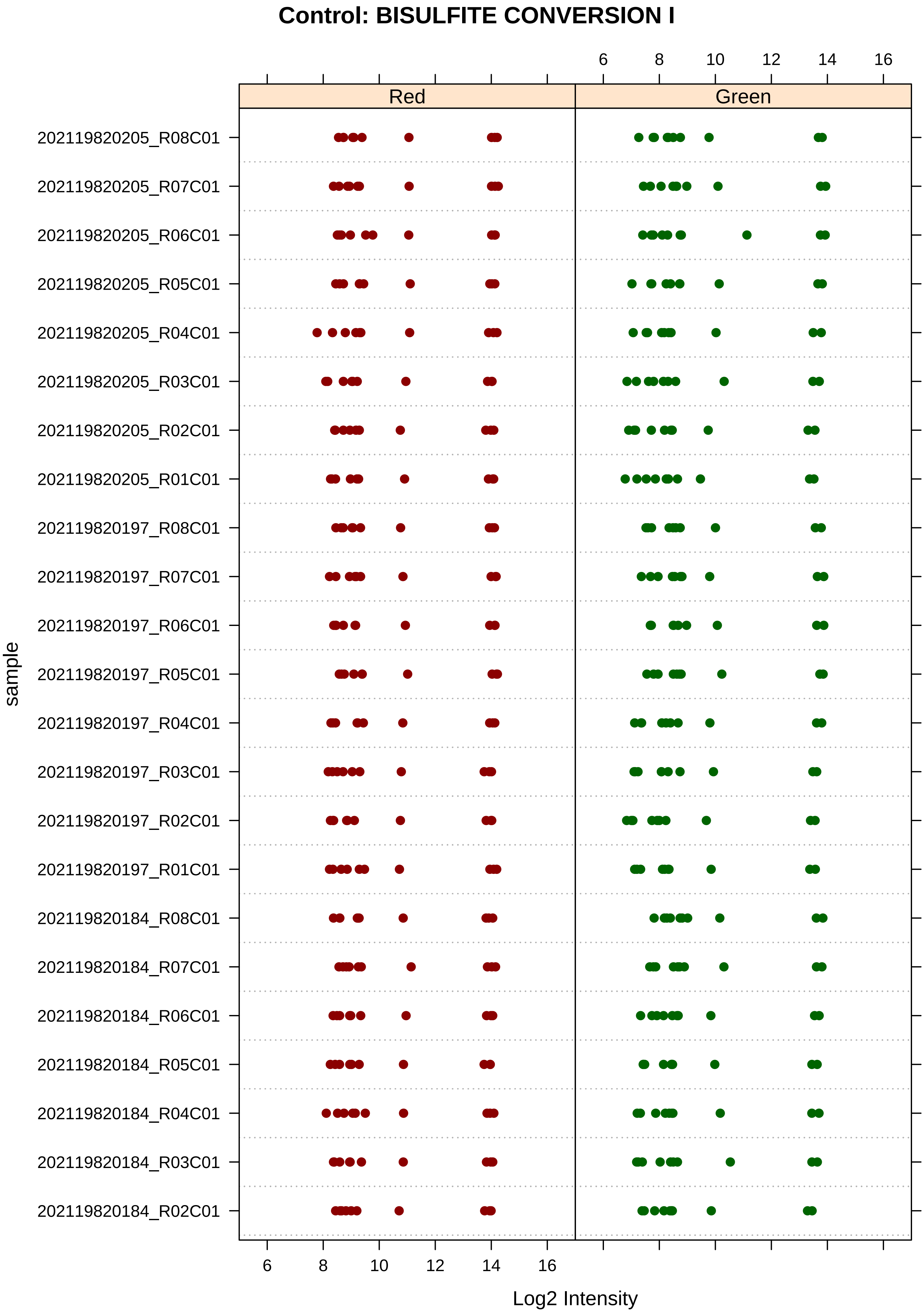

Control: BISULFITE CONVERSION I
Red
Green
202119820205_R08C01
202119820205_R07C01
202119820205_R06C01
202119820205_R05C01
202119820205_R04C01
202119820205_R03C01
202119820205_R02C01
202119820205_R01C01
202119820197_R08C01
202119820197_R07C01
202119820197_R06C01
202119820197_R05C01
202119820197_R04C01
202119820197_R03C01
202119820197_R02C01
202119820197_R01C01
202119820184_R08C01
202119820184_R07C01
202119820184_R06C01
202119820184_R05C01
202119820184_R04C01
202119820184_R03C01
202119820184_R02C01
sample
6
8
10
12
14
16
Log2 Intensity

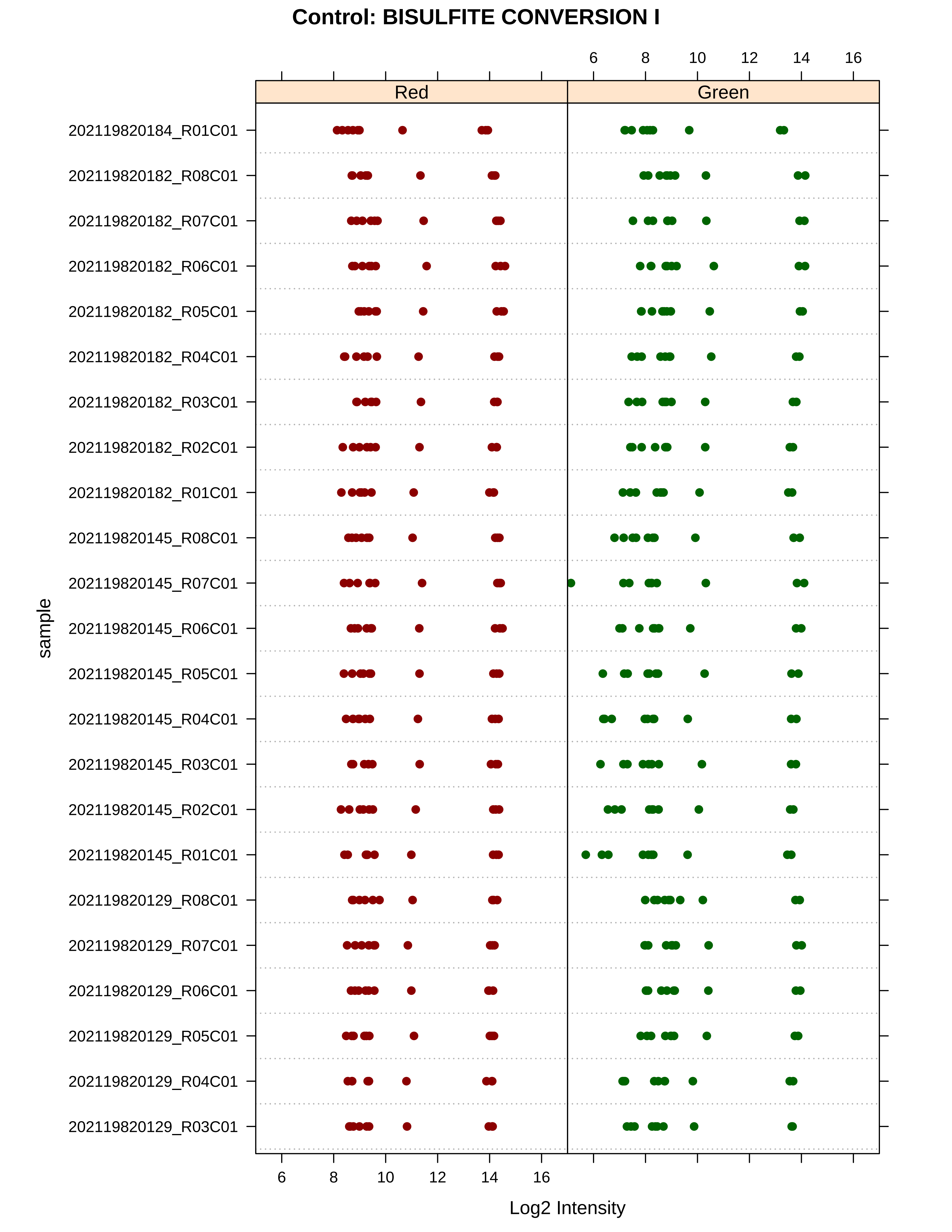
Control: BISULFITE CONVERSION I
Red
Green
sample
Log2 Intensity
6
8
10
12
14
16
202119820184_R01C01
202119820182_R08C01
202119820182_R07C01
202119820182_R06C01
202119820182_R05C01
202119820182_R04C01
202119820182_R03C01
202119820182_R02C01
202119820182_R01C01
202119820145_R08C01
202119820145_R07C01
202119820145_R06C01
202119820145_R05C01
202119820145_R04C01
202119820145_R03C01
202119820145_R02C01
202119820145_R01C01
202119820129_R08C01
202119820129_R07C01
202119820129_R06C01
202119820129_R05C01
202119820129_R04C01
202119820129_R03C01

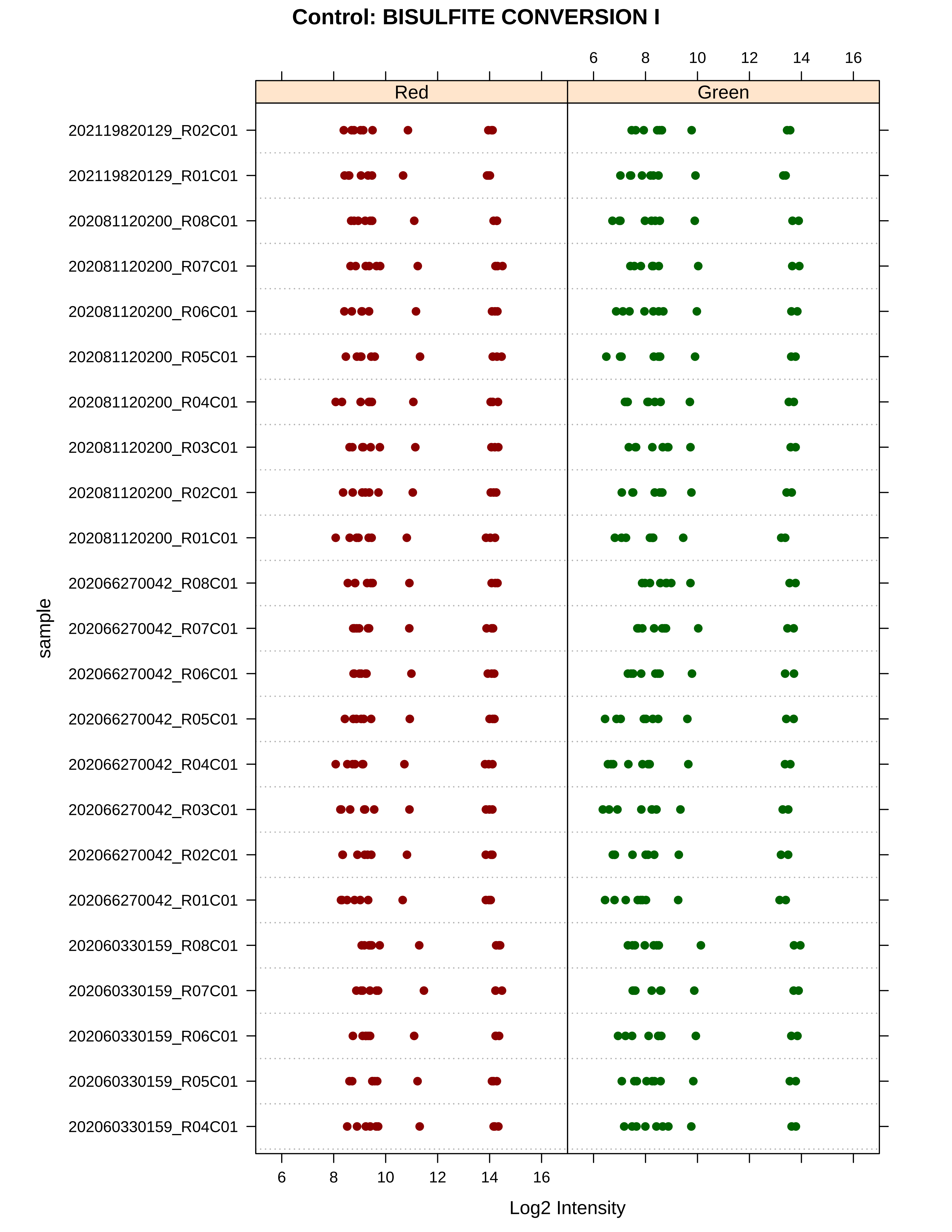

Control: BISULFITE CONVERSION I
Red
Green
sample
202119820129_R02C01
202119820129_R01C01
202081120200_R08C01
202081120200_R07C01
202081120200_R06C01
202081120200_R05C01
202081120200_R04C01
202081120200_R03C01
202081120200_R02C01
202081120200_R01C01
202066270042_R08C01
202066270042_R07C01
202066270042_R06C01
202066270042_R05C01
202066270042_R04C01
202066270042_R03C01
202066270042_R02C01
202066270042_R01C01
202060330159_R08C01
202060330159_R07C01
202060330159_R06C01
202060330159_R05C01
202060330159_R04C01
6 8 10 12 14 16
Log2 Intensity

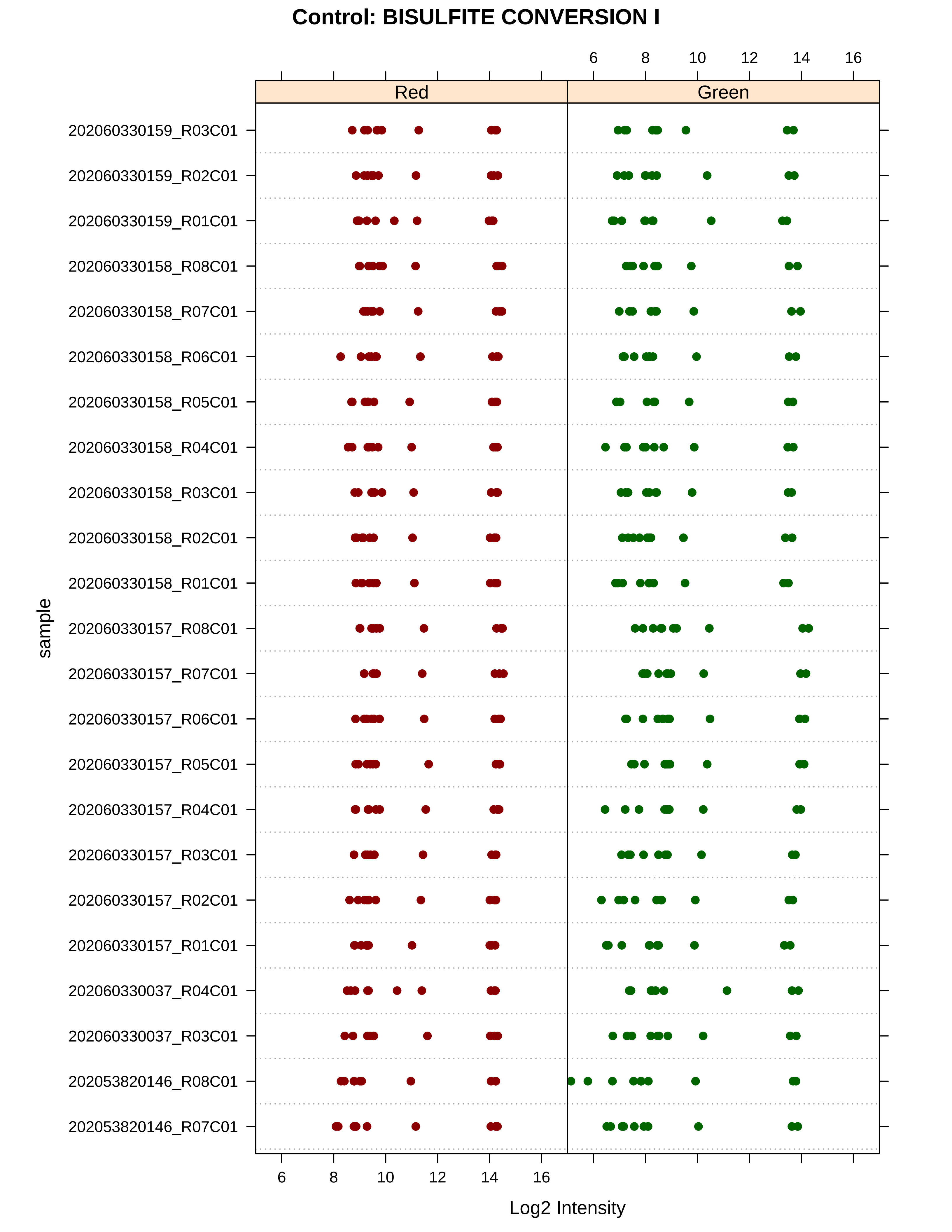

Control: BISULFITE CONVERSION I
Red
Green
sample
Log2 Intensity
202060330159_R03C01
202060330159_R02C01
202060330159_R01C01
202060330158_R08C01
202060330158_R07C01
202060330158_R06C01
202060330158_R05C01
202060330158_R04C01
202060330158_R03C01
202060330158_R02C01
202060330158_R01C01
202060330157_R08C01
202060330157_R07C01
202060330157_R06C01
202060330157_R05C01
202060330157_R04C01
202060330157_R03C01
202060330157_R02C01
202060330157_R01C01
202060330037_R04C01
202060330037_R03C01
202053820146_R08C01
202053820146_R07C01
6
8
10
12
14
16

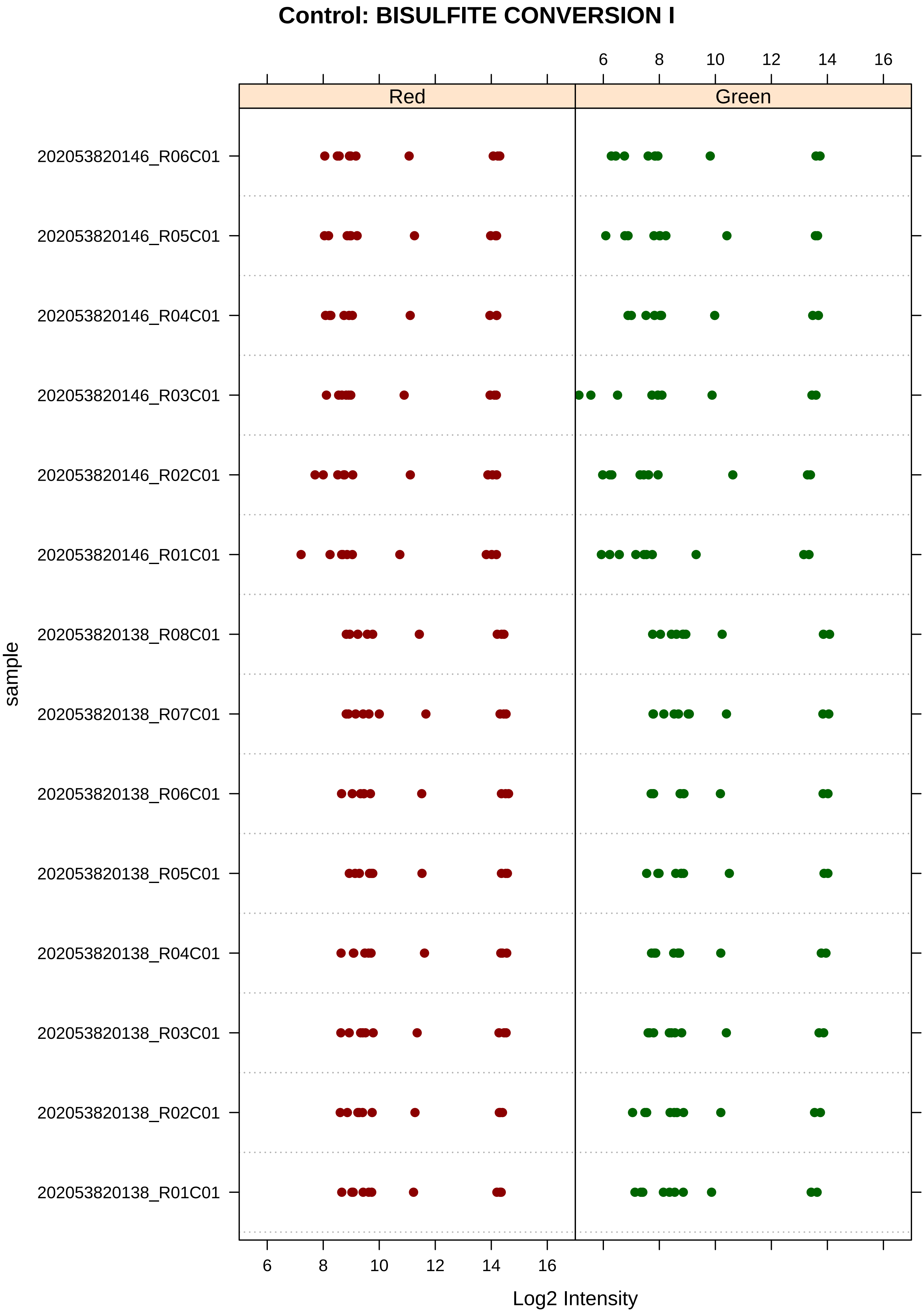

Control: BISULFITE CONVERSION I
Red
Green
202053820146_R06C01
202053820146_R05C01
202053820146_R04C01
202053820146_R03C01
202053820146_R02C01
202053820146_R01C01
202053820138_R08C01
202053820138_R07C01
202053820138_R06C01
202053820138_R05C01
202053820138_R04C01
202053820138_R03C01
202053820138_R02C01
202053820138_R01C01
sample
6
8
10
12
14
16
Log2 Intensity

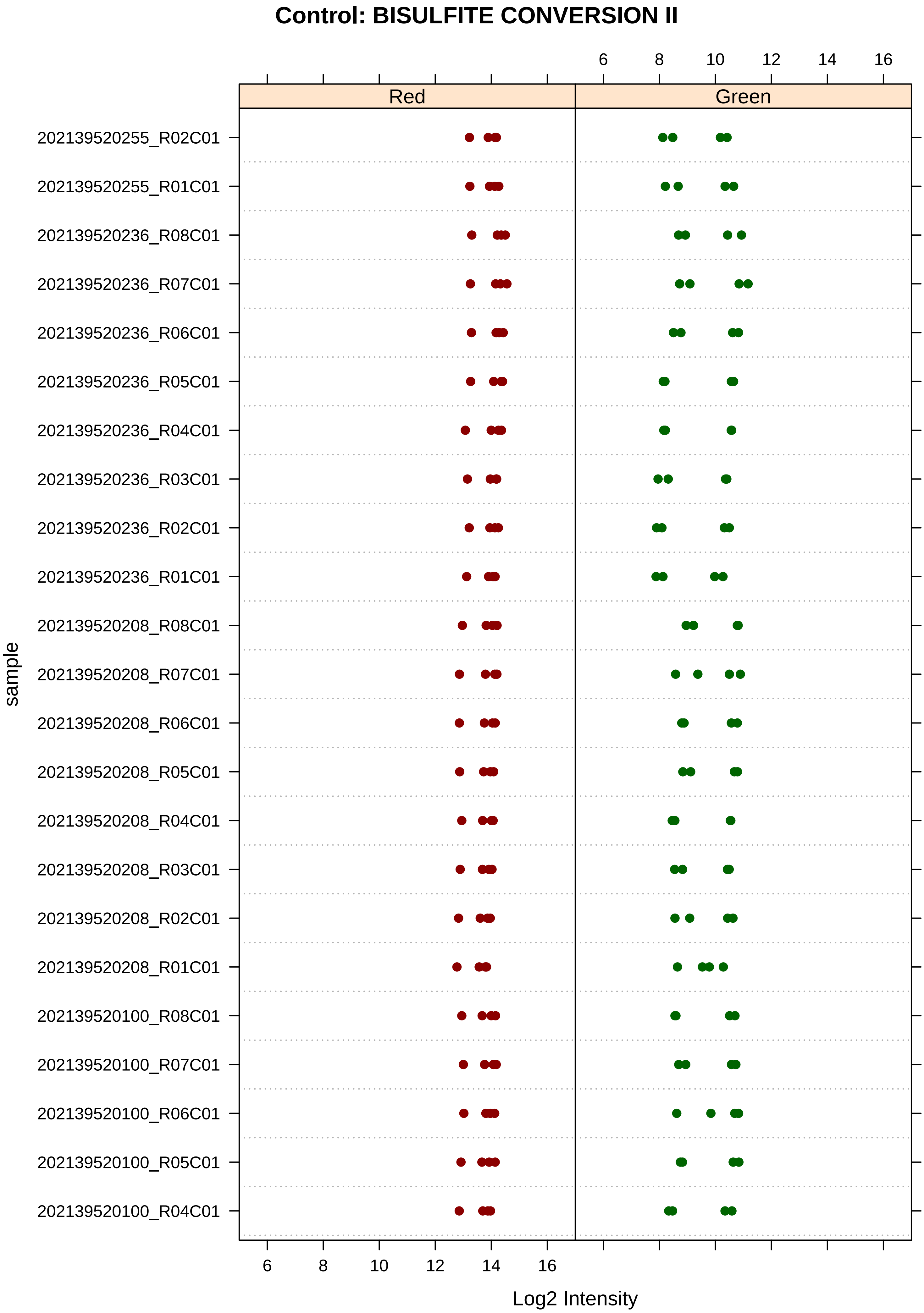
Control: BISULFITE CONVERSION II
Red
Green
sample
Log2 Intensity
202139520255_R02C01
202139520255_R01C01
202139520236_R08C01
202139520236_R07C01
202139520236_R06C01
202139520236_R05C01
202139520236_R04C01
202139520236_R03C01
202139520236_R02C01
202139520236_R01C01
202139520208_R08C01
202139520208_R07C01
202139520208_R06C01
202139520208_R05C01
202139520208_R04C01
202139520208_R03C01
202139520208_R02C01
202139520208_R01C01
202139520100_R08C01
202139520100_R07C01
202139520100_R06C01
202139520100_R05C01
202139520100_R04C01
6
8
10
12
14
16

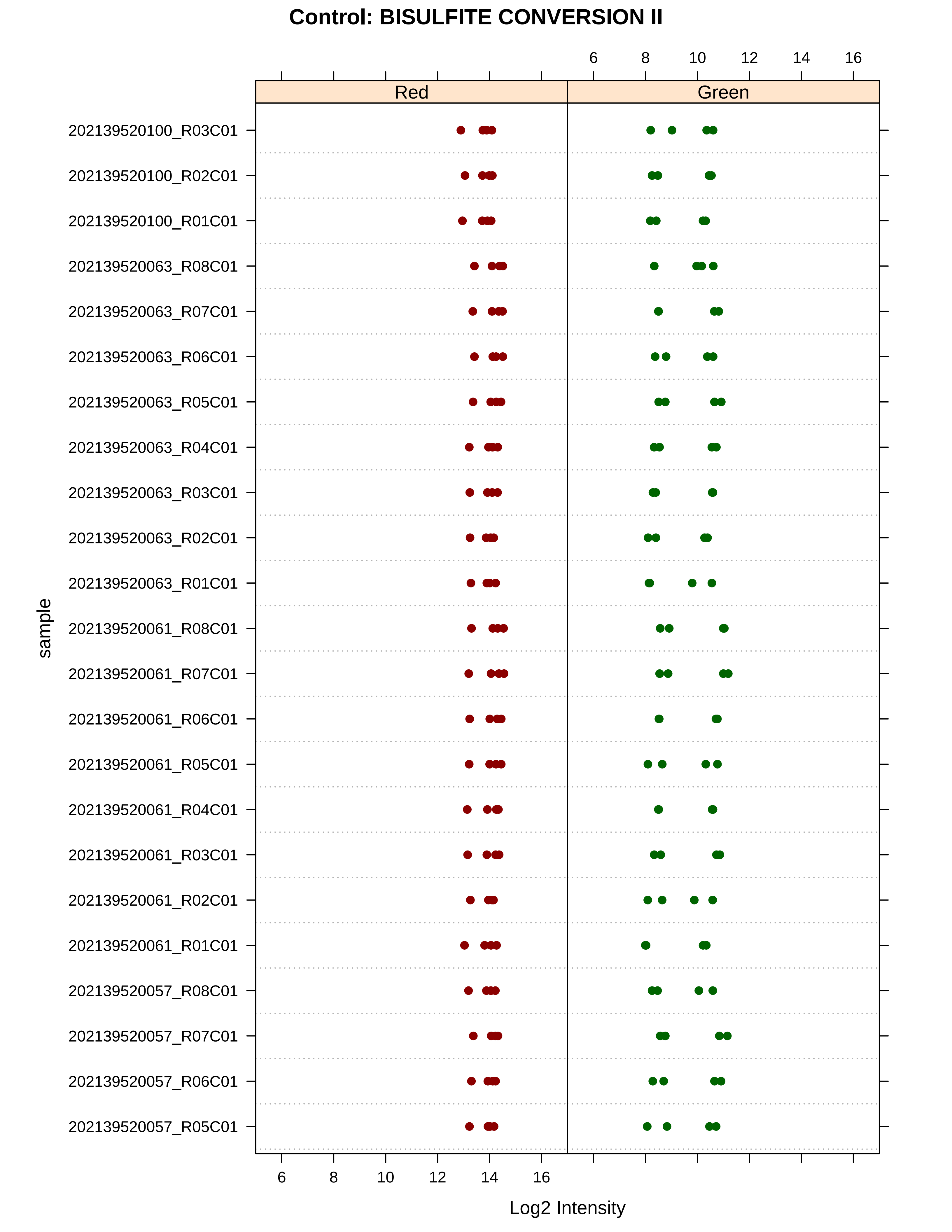
Control: BISULFITE CONVERSION II
Red
Green
sample
Log2 Intensity
202139520100_R03C01
202139520100_R02C01
202139520100_R01C01
202139520063_R08C01
202139520063_R07C01
202139520063_R06C01
202139520063_R05C01
202139520063_R04C01
202139520063_R03C01
202139520063_R02C01
202139520063_R01C01
202139520061_R08C01
202139520061_R07C01
202139520061_R06C01
202139520061_R05C01
202139520061_R04C01
202139520061_R03C01
202139520061_R02C01
202139520061_R01C01
202139520057_R08C01
202139520057_R07C01
202139520057_R06C01
202139520057_R05C01
6
8
10
12
14
16

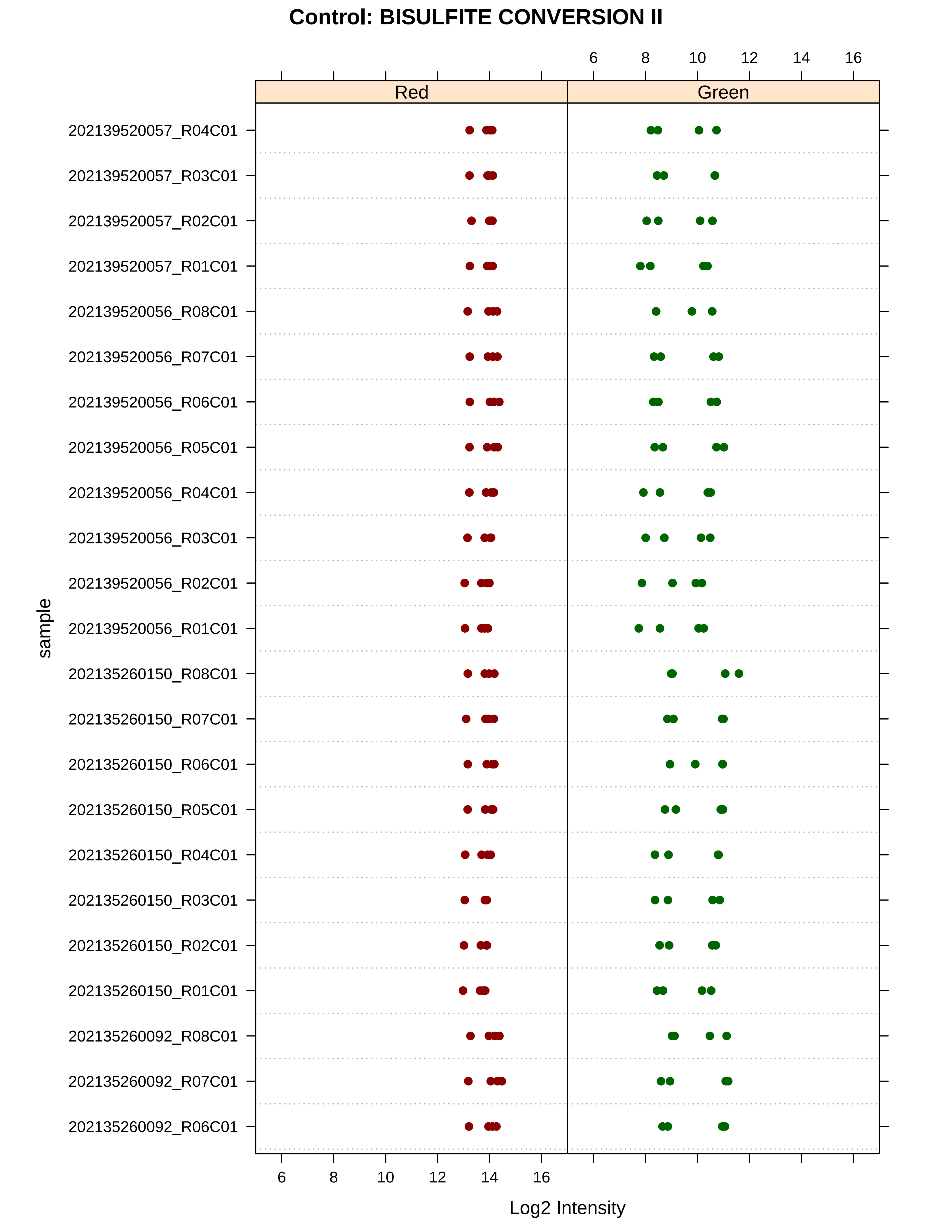

Control: BISULFITE CONVERSION II
Red
Green
sample
Log2 Intensity
202139520057_R04C01
202139520057_R03C01
202139520057_R02C01
202139520057_R01C01
202139520056_R08C01
202139520056_R07C01
202139520056_R06C01
202139520056_R05C01
202139520056_R04C01
202139520056_R03C01
202139520056_R02C01
202139520056_R01C01
202135260150_R08C01
202135260150_R07C01
202135260150_R06C01
202135260150_R05C01
202135260150_R04C01
202135260150_R03C01
202135260150_R02C01
202135260150_R01C01
202135260092_R08C01
202135260092_R07C01
202135260092_R06C01
6
8
10
12
14
16

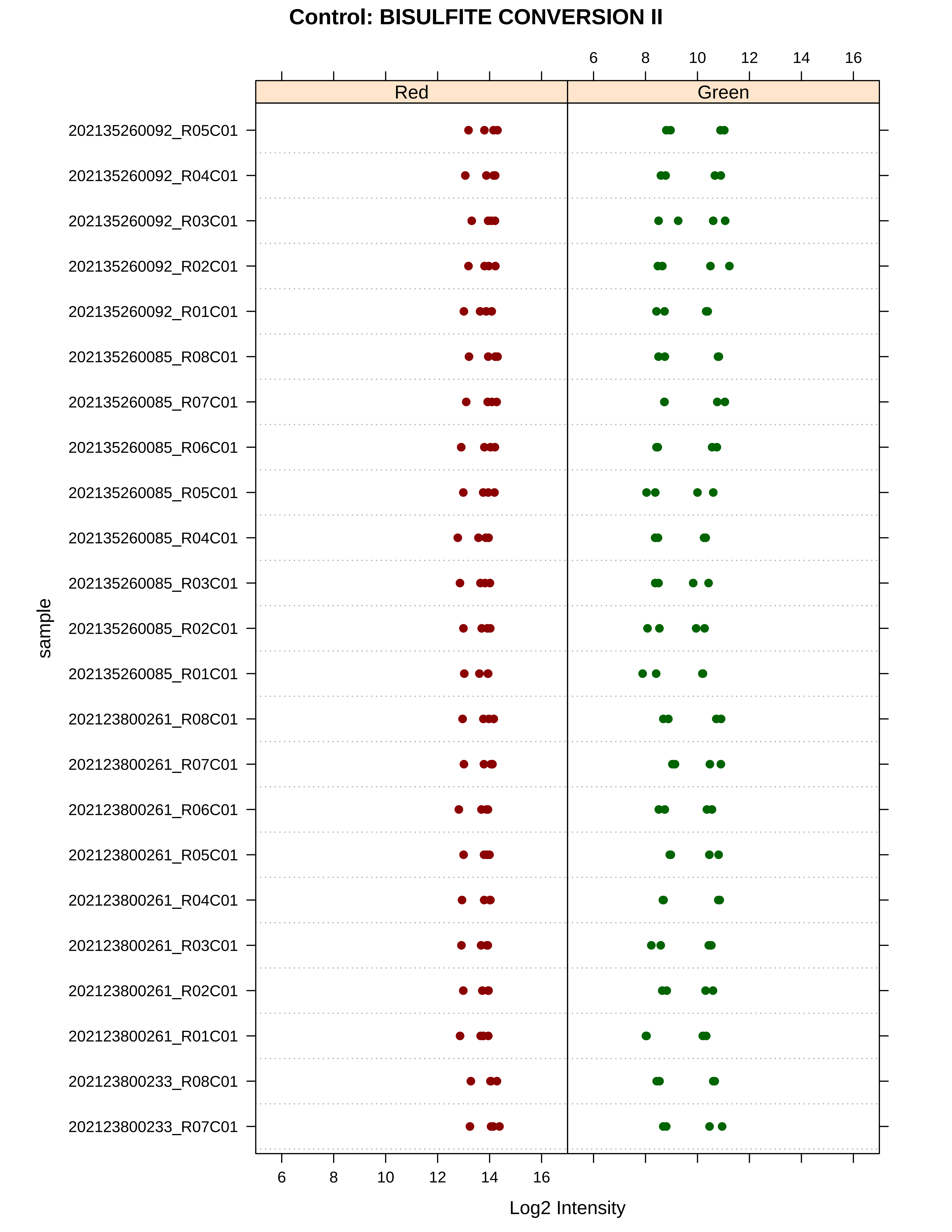

Control: BISULFITE CONVERSION II
Red
Green
sample
Log2 Intensity
6
8
10
12
14
16
202135260092_R05C01
202135260092_R04C01
202135260092_R03C01
202135260092_R02C01
202135260092_R01C01
202135260085_R08C01
202135260085_R07C01
202135260085_R06C01
202135260085_R05C01
202135260085_R04C01
202135260085_R03C01
202135260085_R02C01
202135260085_R01C01
202123800261_R08C01
202123800261_R07C01
202123800261_R06C01
202123800261_R05C01
202123800261_R04C01
202123800261_R03C01
202123800261_R02C01
202123800261_R01C01
202123800233_R08C01
202123800233_R07C01

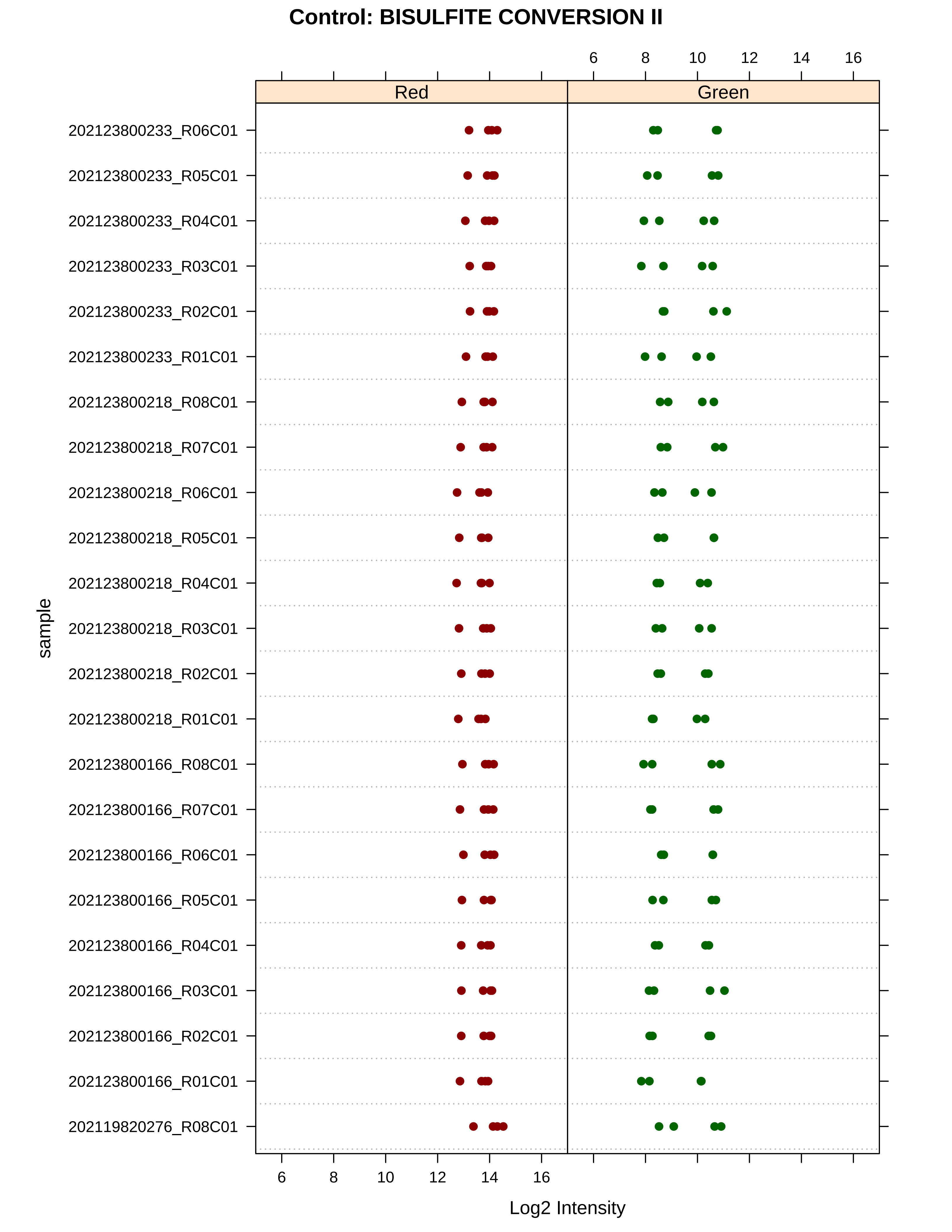

Control: BISULFITE CONVERSION II
Red
Green
sample
Log2 Intensity
202123800233_R06C01
202123800233_R05C01
202123800233_R04C01
202123800233_R03C01
202123800233_R02C01
202123800233_R01C01
202123800218_R08C01
202123800218_R07C01
202123800218_R06C01
202123800218_R05C01
202123800218_R04C01
202123800218_R03C01
202123800218_R02C01
202123800218_R01C01
202123800166_R08C01
202123800166_R07C01
202123800166_R06C01
202123800166_R05C01
202123800166_R04C01
202123800166_R03C01
202123800166_R02C01
202123800166_R01C01
202119820276_R08C01
6
8
10
12
14
16

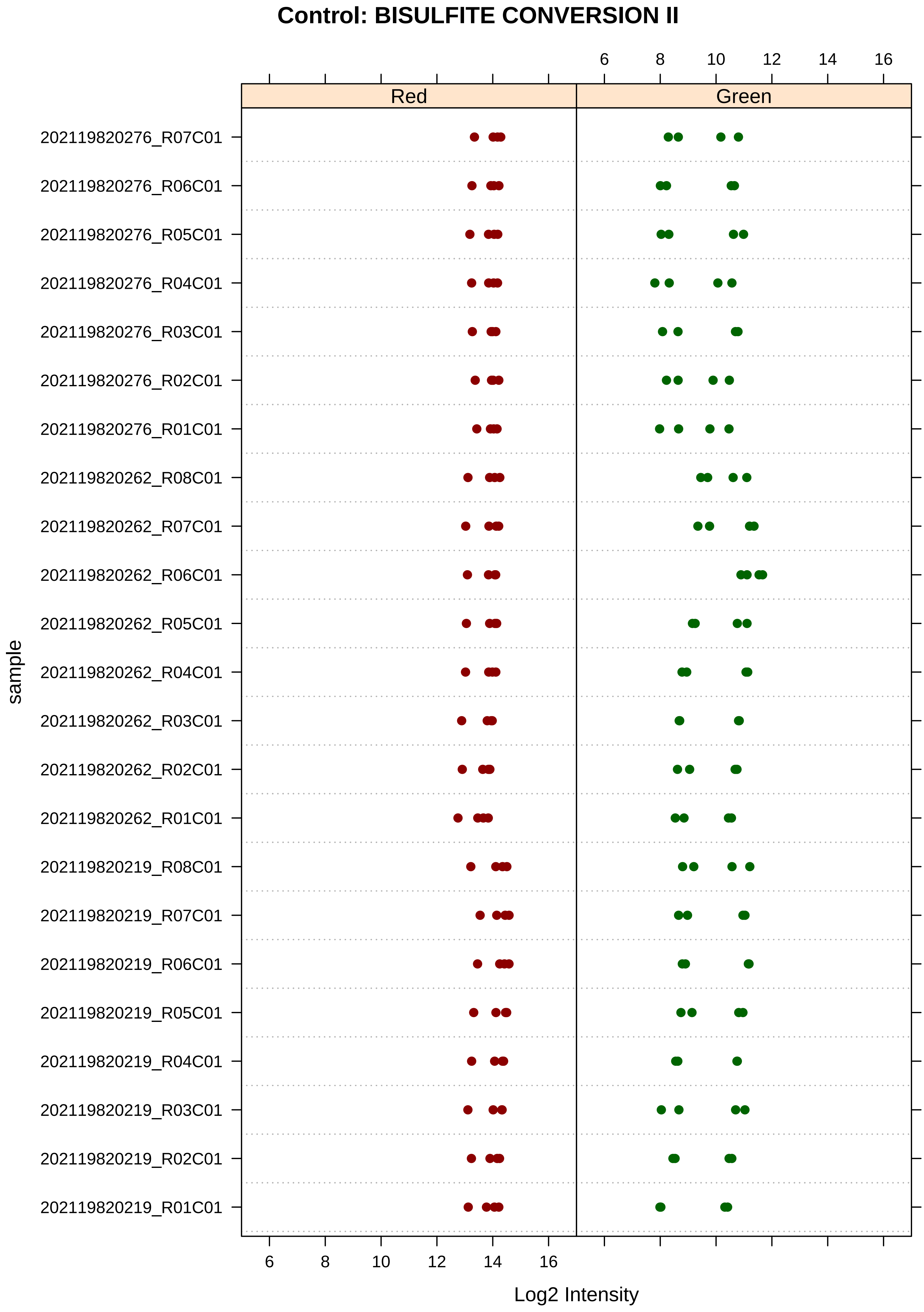

Control: BISULFITE CONVERSION II
Red
Green
sample
Log2 Intensity
6
8
10
12
14
16
202119820276_R07C01
202119820276_R06C01
202119820276_R05C01
202119820276_R04C01
202119820276_R03C01
202119820276_R02C01
202119820276_R01C01
202119820262_R08C01
202119820262_R07C01
202119820262_R06C01
202119820262_R05C01
202119820262_R04C01
202119820262_R03C01
202119820262_R02C01
202119820262_R01C01
202119820219_R08C01
202119820219_R07C01
202119820219_R06C01
202119820219_R05C01
202119820219_R04C01
202119820219_R03C01
202119820219_R02C01
202119820219_R01C01

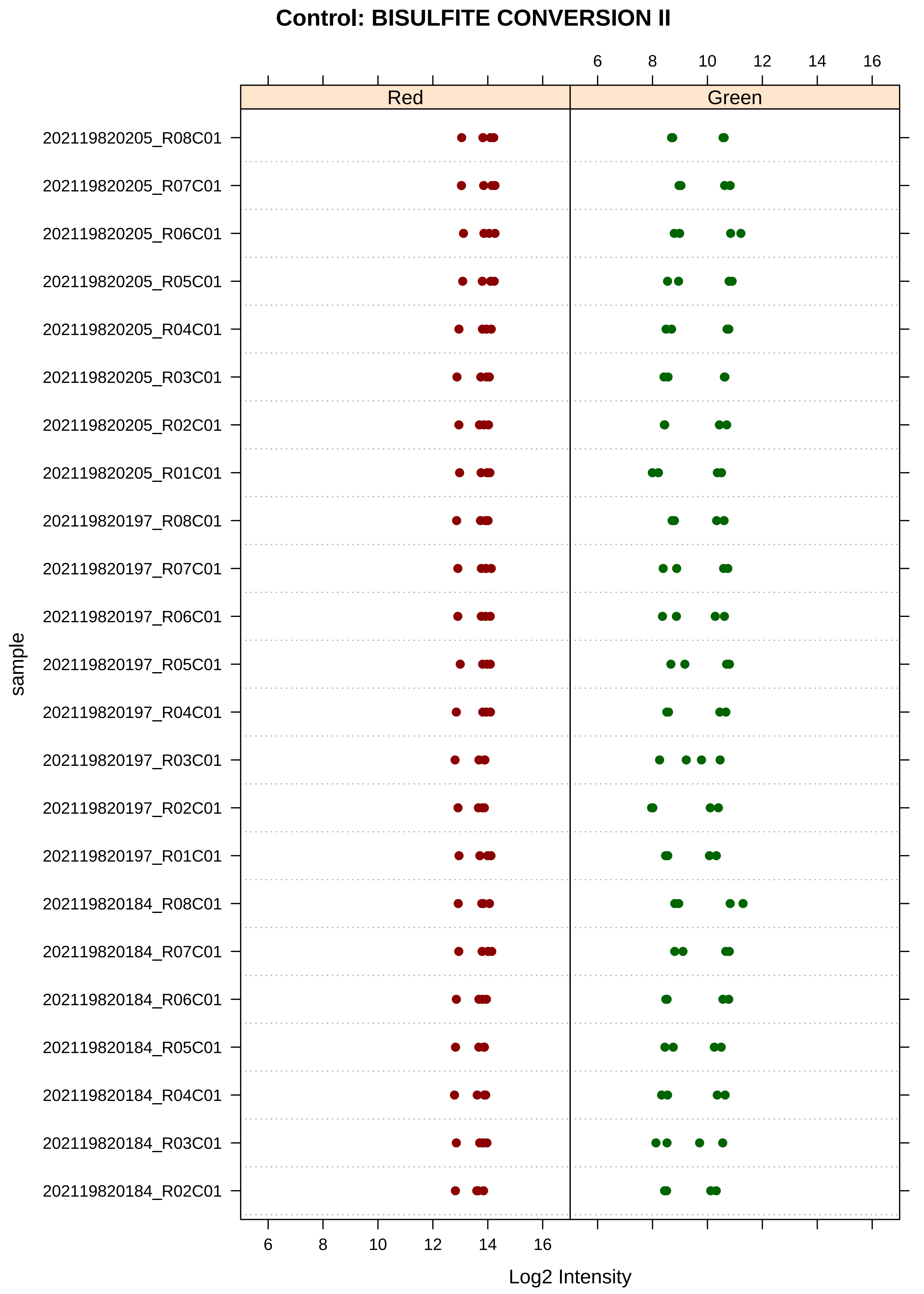

Control: BISULFITE CONVERSION II
Red
Green
sample
Log2 Intensity
202119820205_R08C01
202119820205_R07C01
202119820205_R06C01
202119820205_R05C01
202119820205_R04C01
202119820205_R03C01
202119820205_R02C01
202119820205_R01C01
202119820197_R08C01
202119820197_R07C01
202119820197_R06C01
202119820197_R05C01
202119820197_R04C01
202119820197_R03C01
202119820197_R02C01
202119820197_R01C01
202119820184_R08C01
202119820184_R07C01
202119820184_R06C01
202119820184_R05C01
202119820184_R04C01
202119820184_R03C01
202119820184_R02C01
6
8
10
12
14
16

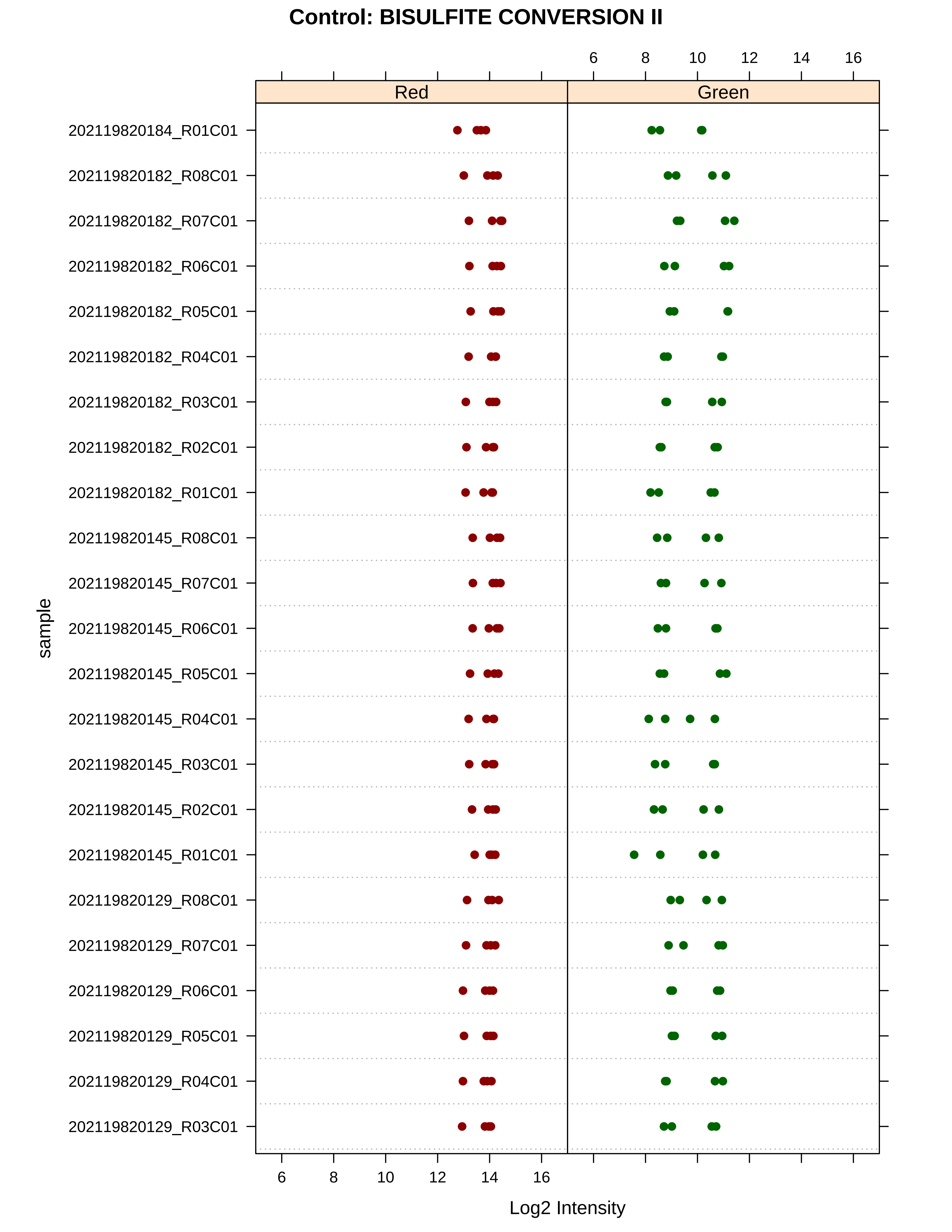
Control: BISULFITE CONVERSION II
Red
Green
sample
Log2 Intensity
202119820184_R01C01
202119820182_R08C01
202119820182_R07C01
202119820182_R06C01
202119820182_R05C01
202119820182_R04C01
202119820182_R03C01
202119820182_R02C01
202119820182_R01C01
202119820145_R08C01
202119820145_R07C01
202119820145_R06C01
202119820145_R05C01
202119820145_R04C01
202119820145_R03C01
202119820145_R02C01
202119820145_R01C01
202119820129_R08C01
202119820129_R07C01
202119820129_R06C01
202119820129_R05C01
202119820129_R04C01
202119820129_R03C01
6
8
10
12
14
16

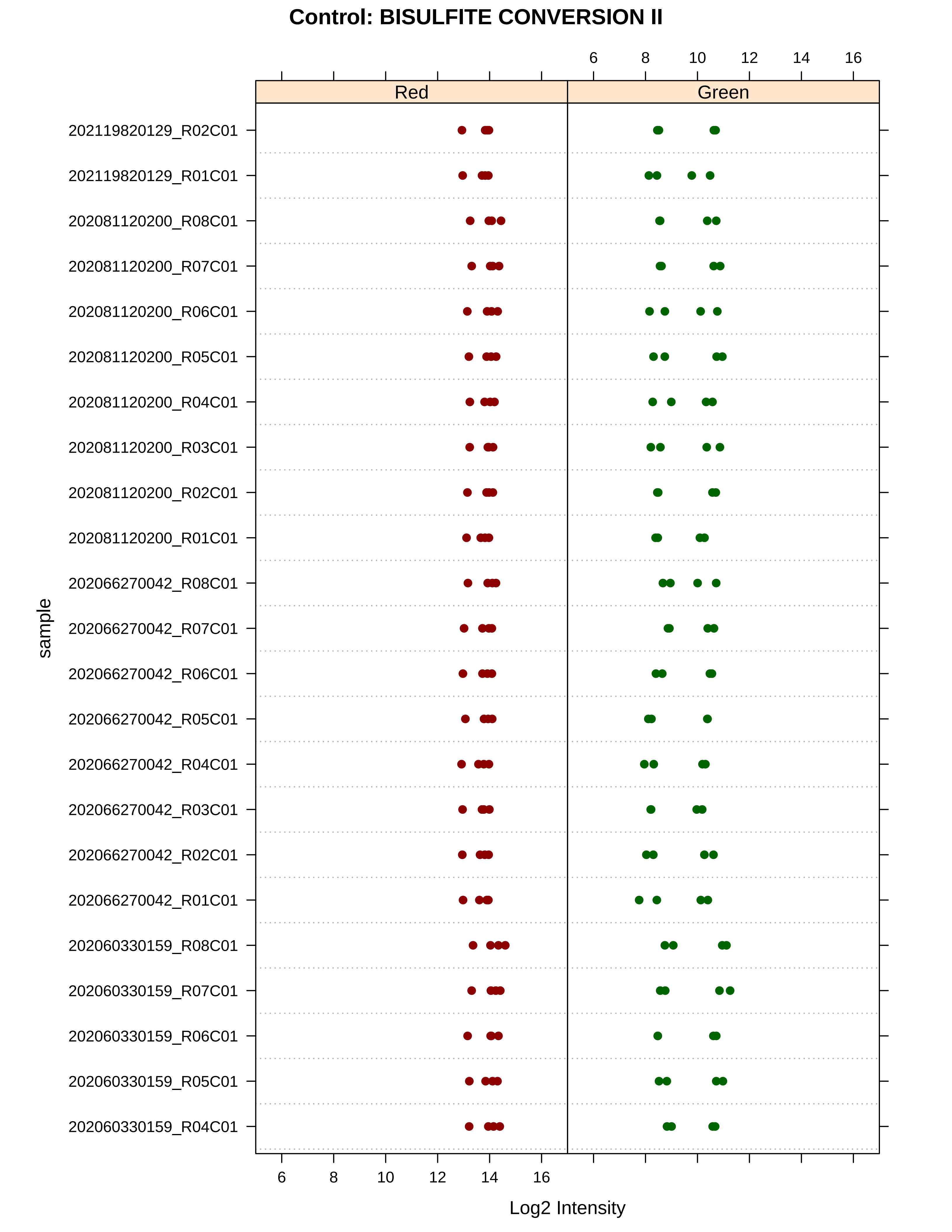

Control: BISULFITE CONVERSION II
Red
Green
202119820129_R02C01
202119820129_R01C01
202081120200_R08C01
202081120200_R07C01
202081120200_R06C01
202081120200_R05C01
202081120200_R04C01
202081120200_R03C01
202081120200_R02C01
202081120200_R01C01
202066270042_R08C01
202066270042_R07C01
202066270042_R06C01
202066270042_R05C01
202066270042_R04C01
202066270042_R03C01
202066270042_R02C01
202066270042_R01C01
202060330159_R08C01
202060330159_R07C01
202060330159_R06C01
202060330159_R05C01
202060330159_R04C01
sample
6
8
10
12
14
16
Log2 Intensity

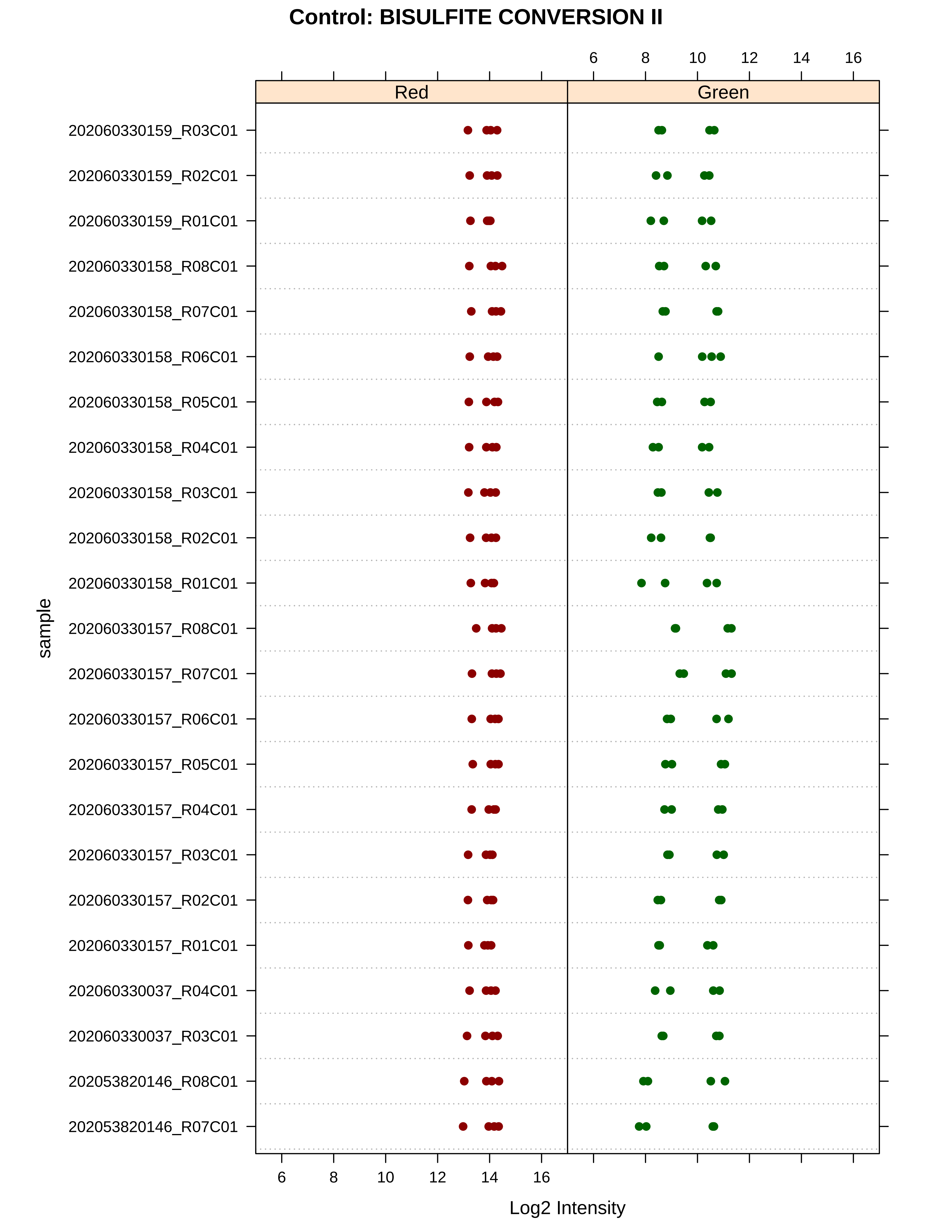
Control: BISULFITE CONVERSION II
Red
Green
sample
Log2 Intensity
202060330159_R03C01
202060330159_R02C01
202060330159_R01C01
202060330158_R08C01
202060330158_R07C01
202060330158_R06C01
202060330158_R05C01
202060330158_R04C01
202060330158_R03C01
202060330158_R02C01
202060330158_R01C01
202060330157_R08C01
202060330157_R07C01
202060330157_R06C01
202060330157_R05C01
202060330157_R04C01
202060330157_R03C01
202060330157_R02C01
202060330157_R01C01
202060330037_R04C01
202060330037_R03C01
202053820146_R08C01
202053820146_R07C01
6 8 10 12 14 16

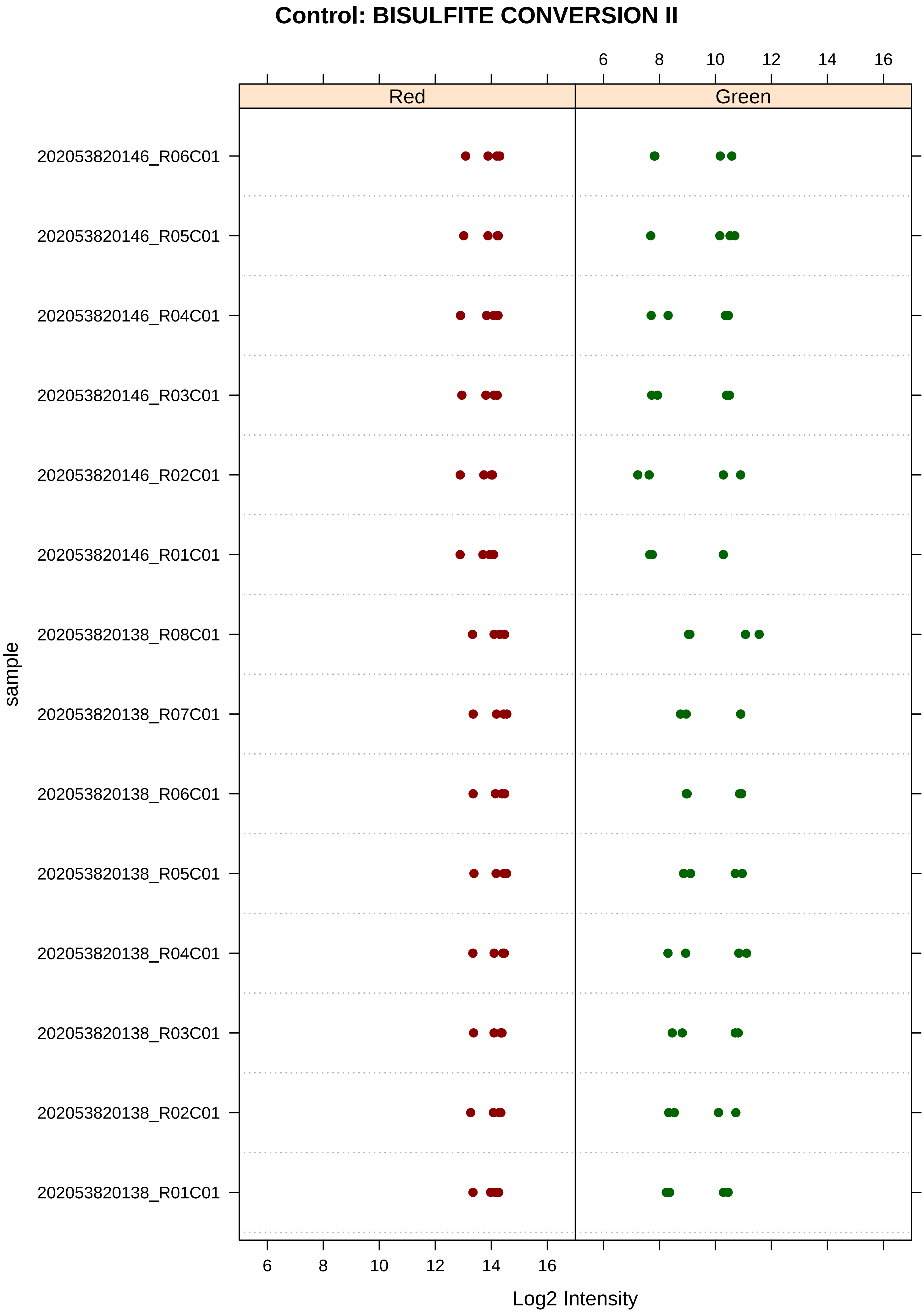
Control: BISULFITE CONVERSION II
Red
Green
sample
202053820146_R06C01
202053820146_R05C01
202053820146_R04C01
202053820146_R03C01
202053820146_R02C01
202053820146_R01C01
202053820138_R08C01
202053820138_R07C01
202053820138_R06C01
202053820138_R05C01
202053820138_R04C01
202053820138_R03C01
202053820138_R02C01
202053820138_R01C01
6
8
10
12
14
16
Log2 Intensity

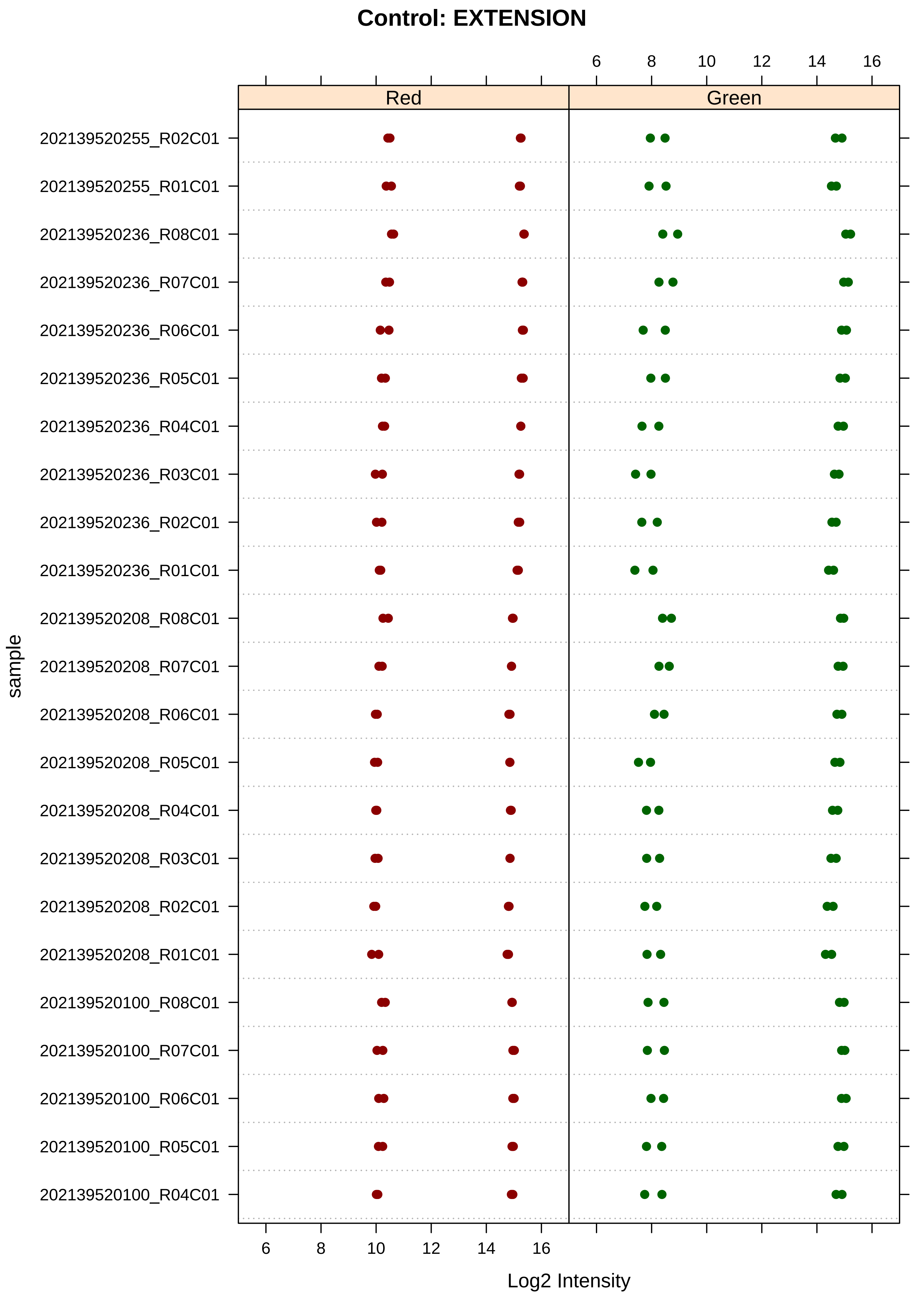
Control: EXTENSION
Red
Green
sample
202139520255_R02C01
202139520255_R01C01
202139520236_R08C01
202139520236_R07C01
202139520236_R06C01
202139520236_R05C01
202139520236_R04C01
202139520236_R03C01
202139520236_R02C01
202139520236_R01C01
202139520208_R08C01
202139520208_R07C01
202139520208_R06C01
202139520208_R05C01
202139520208_R04C01
202139520208_R03C01
202139520208_R02C01
202139520208_R01C01
202139520100_R08C01
202139520100_R07C01
202139520100_R06C01
202139520100_R05C01
202139520100_R04C01
6
8
10
12
14
16
Log2 Intensity

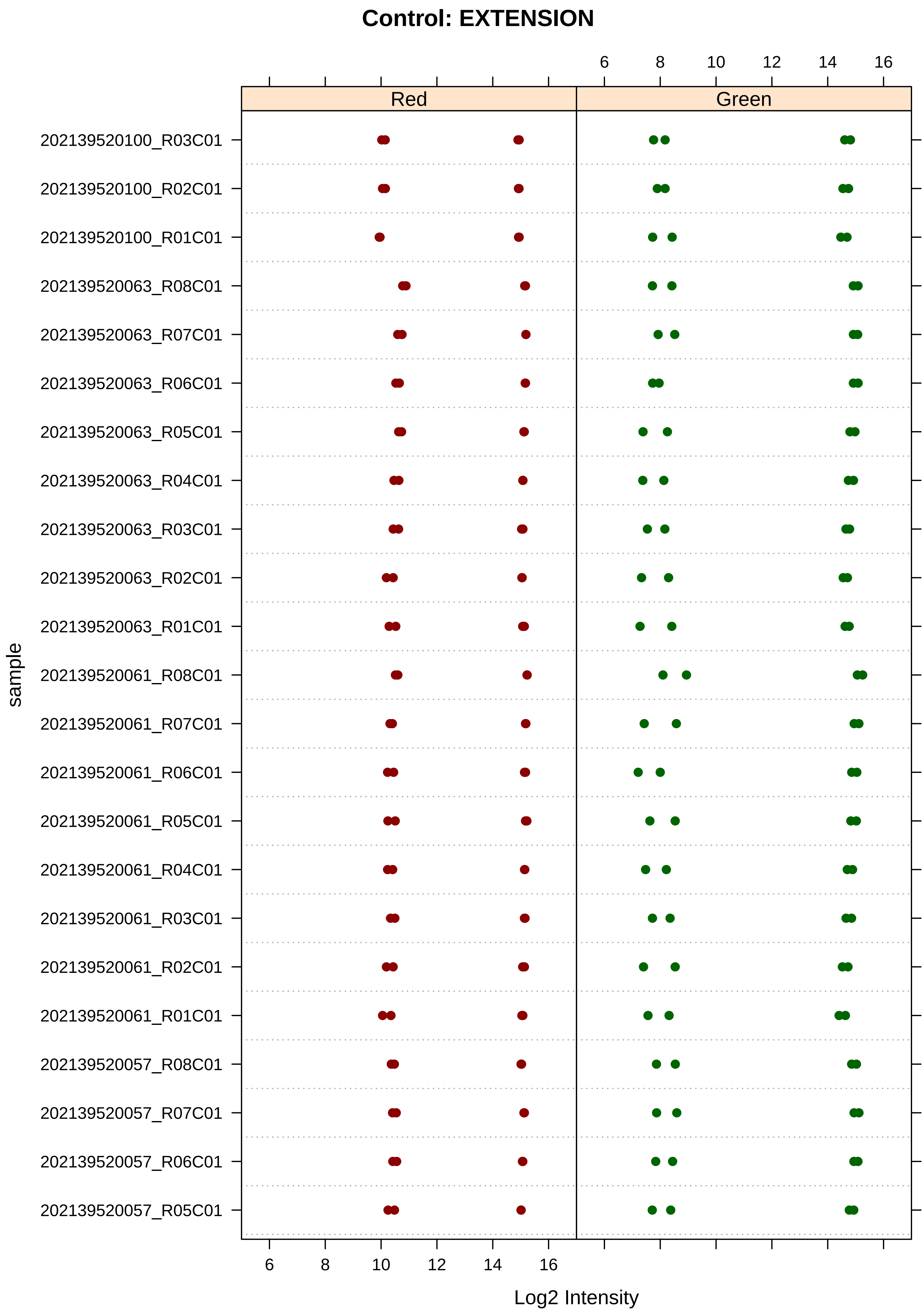
Control: EXTENSION
Red
Green
sample
Log2 Intensity
202139520100_R03C01
202139520100_R02C01
202139520100_R01C01
202139520063_R08C01
202139520063_R07C01
202139520063_R06C01
202139520063_R05C01
202139520063_R04C01
202139520063_R03C01
202139520063_R02C01
202139520063_R01C01
202139520061_R08C01
202139520061_R07C01
202139520061_R06C01
202139520061_R05C01
202139520061_R04C01
202139520061_R03C01
202139520061_R02C01
202139520061_R01C01
202139520057_R08C01
202139520057_R07C01
202139520057_R06C01
202139520057_R05C01
6
8
10
12
14
16

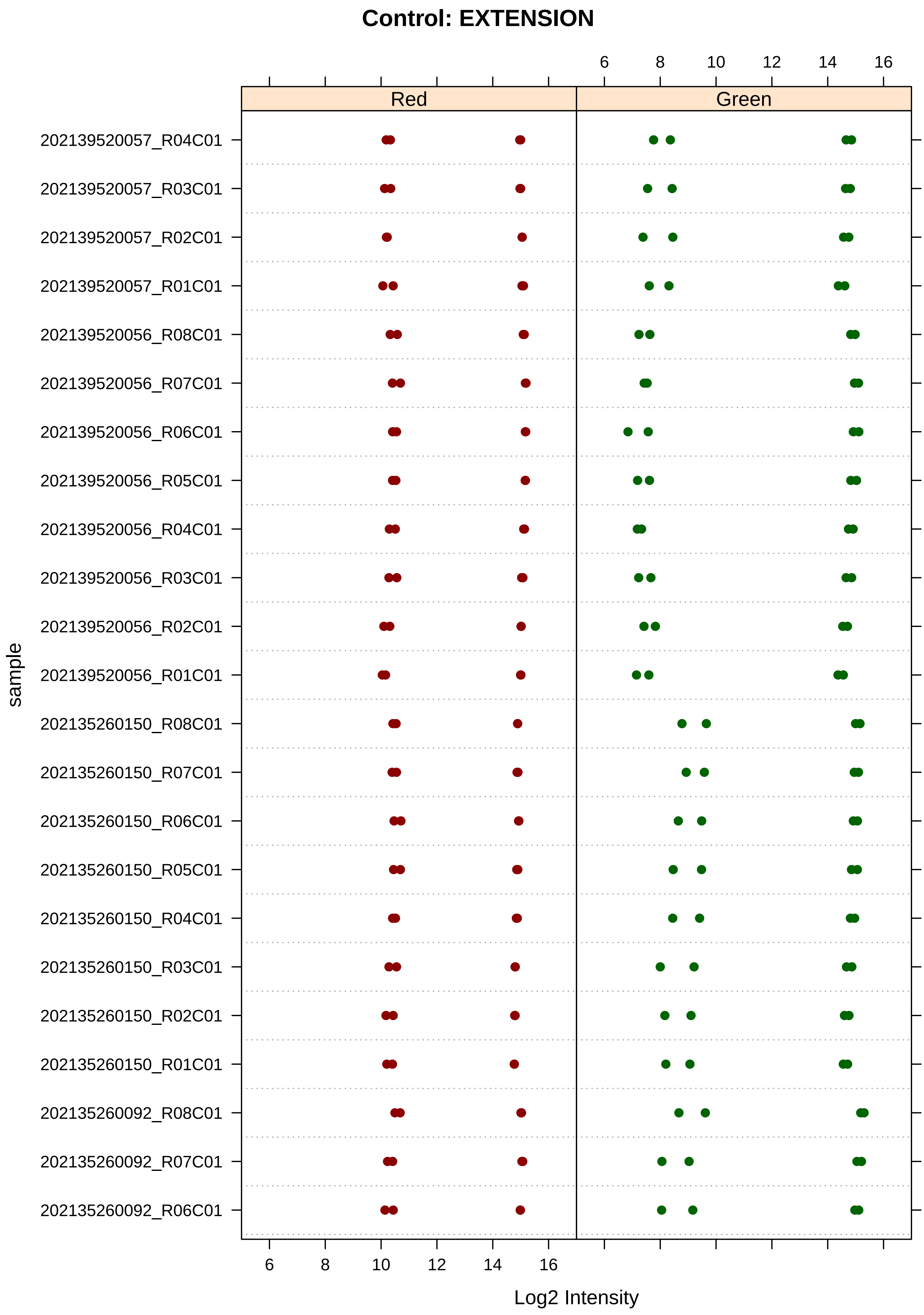
Control: EXTENSION
Red
Green
sample
Log2 Intensity
6
8
10
12
14
16
202139520057_R04C01
202139520057_R03C01
202139520057_R02C01
202139520057_R01C01
202139520056_R08C01
202139520056_R07C01
202139520056_R06C01
202139520056_R05C01
202139520056_R04C01
202139520056_R03C01
202139520056_R02C01
202139520056_R01C01
202135260150_R08C01
202135260150_R07C01
202135260150_R06C01
202135260150_R05C01
202135260150_R04C01
202135260150_R03C01
202135260150_R02C01
202135260150_R01C01
202135260092_R08C01
202135260092_R07C01
202135260092_R06C01

Control: EXTENSION

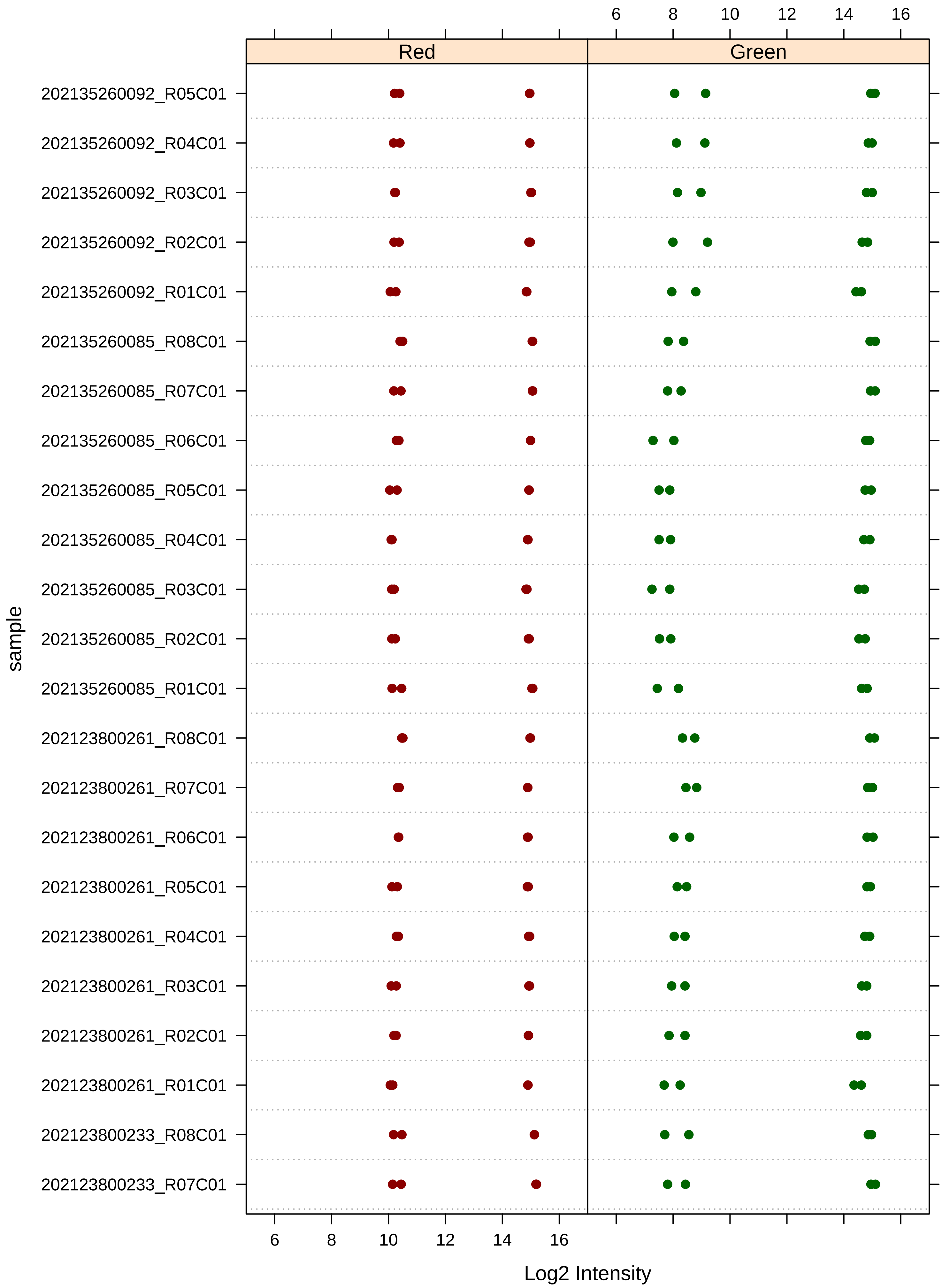

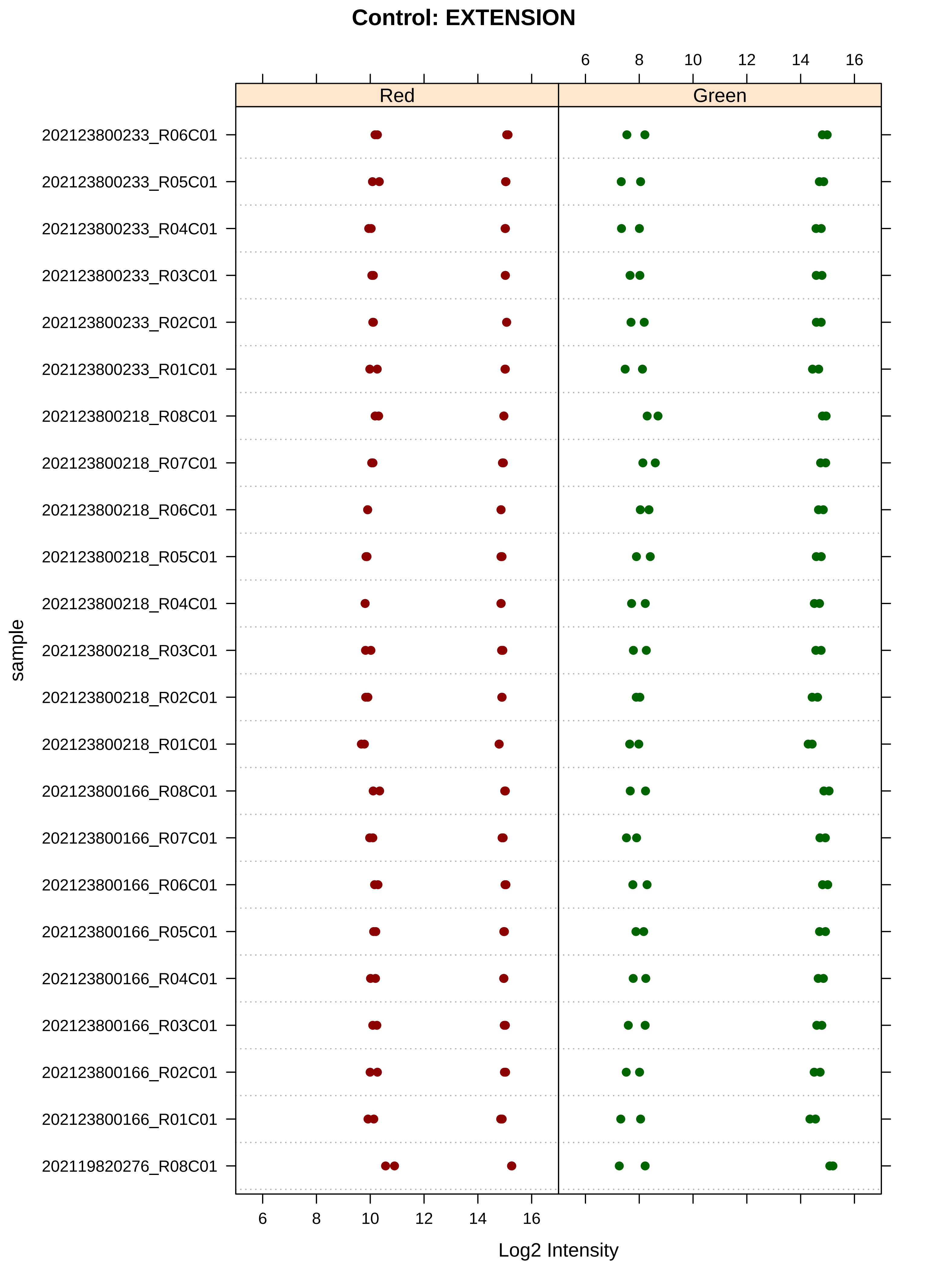
Control: EXTENSION
Red
Green
sample
Log2 Intensity
202123800233_R06C01
202123800233_R05C01
202123800233_R04C01
202123800233_R03C01
202123800233_R02C01
202123800233_R01C01
202123800218_R08C01
202123800218_R07C01
202123800218_R06C01
202123800218_R05C01
202123800218_R04C01
202123800218_R03C01
202123800218_R02C01
202123800218_R01C01
202123800166_R08C01
202123800166_R07C01
202123800166_R06C01
202123800166_R05C01
202123800166_R04C01
202123800166_R03C01
202123800166_R02C01
202123800166_R01C01
202119820276_R08C01
6
8
10
12
14
16

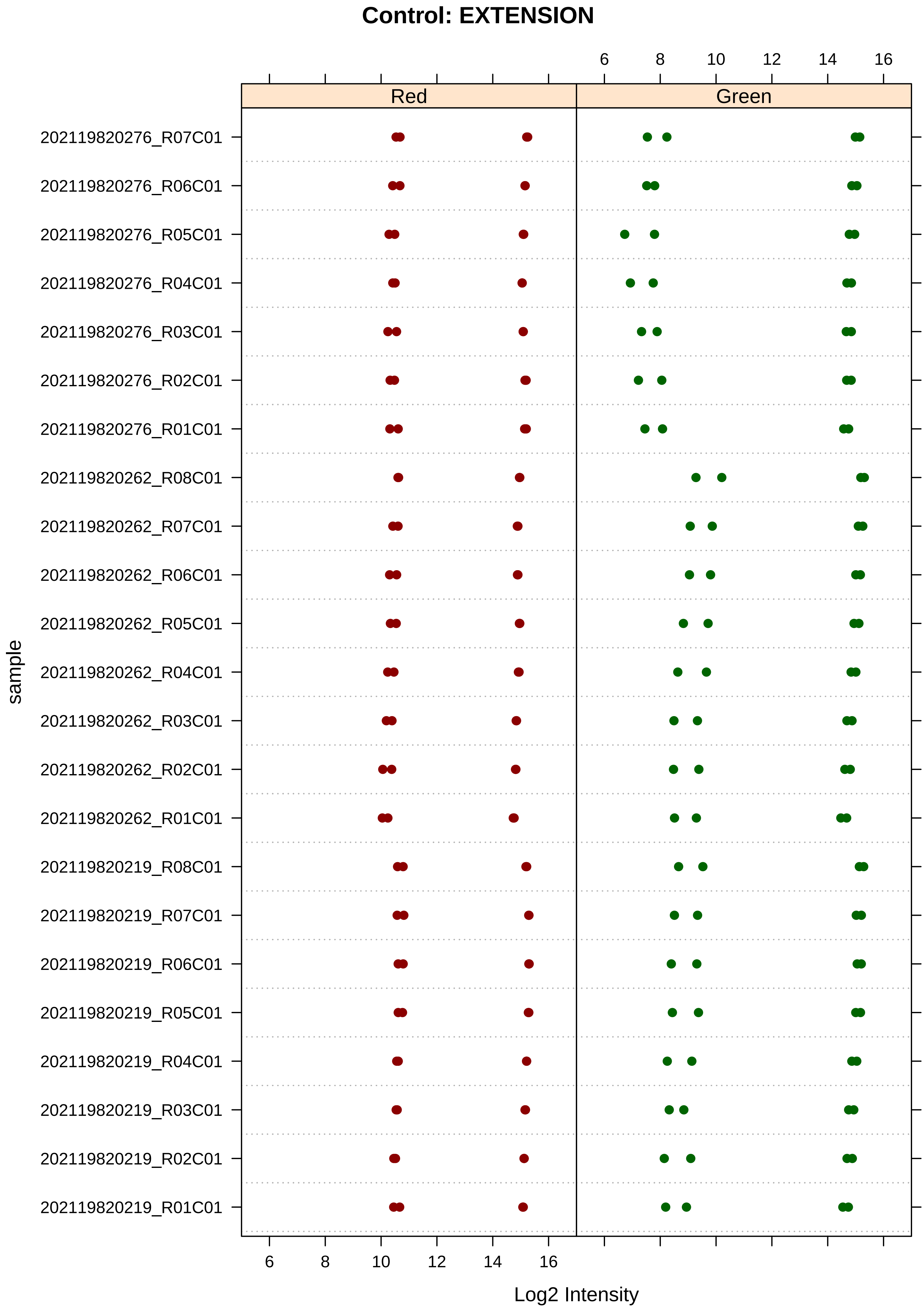
Control: EXTENSION
Red
Green
sample
202119820276_R07C01
202119820276_R06C01
202119820276_R05C01
202119820276_R04C01
202119820276_R03C01
202119820276_R02C01
202119820276_R01C01
202119820262_R08C01
202119820262_R07C01
202119820262_R06C01
202119820262_R05C01
202119820262_R04C01
202119820262_R03C01
202119820262_R02C01
202119820262_R01C01
202119820219_R08C01
202119820219_R07C01
202119820219_R06C01
202119820219_R05C01
202119820219_R04C01
202119820219_R03C01
202119820219_R02C01
202119820219_R01C01
6
8
10
12
14
16
Log2 Intensity

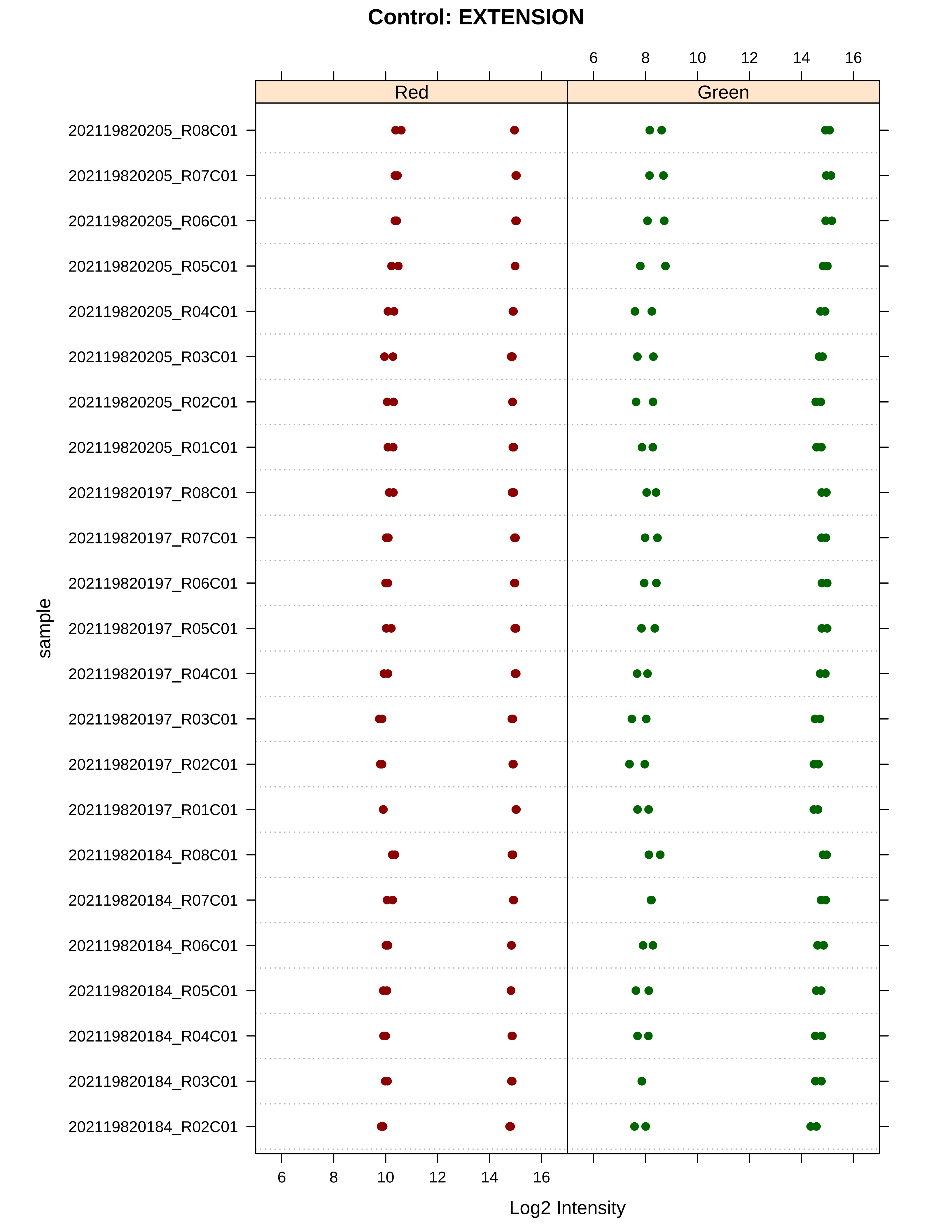
Control: EXTENSION
Red
Green
sample
202119820205_R08C01
202119820205_R07C01
202119820205_R06C01
202119820205_R05C01
202119820205_R04C01
202119820205_R03C01
202119820205_R02C01
202119820205_R01C01
202119820197_R08C01
202119820197_R07C01
202119820197_R06C01
202119820197_R05C01
202119820197_R04C01
202119820197_R03C01
202119820197_R02C01
202119820197_R01C01
202119820184_R08C01
202119820184_R07C01
202119820184_R06C01
202119820184_R05C01
202119820184_R04C01
202119820184_R03C01
202119820184_R02C01
6
8
10
12
14
16
Log2 Intensity

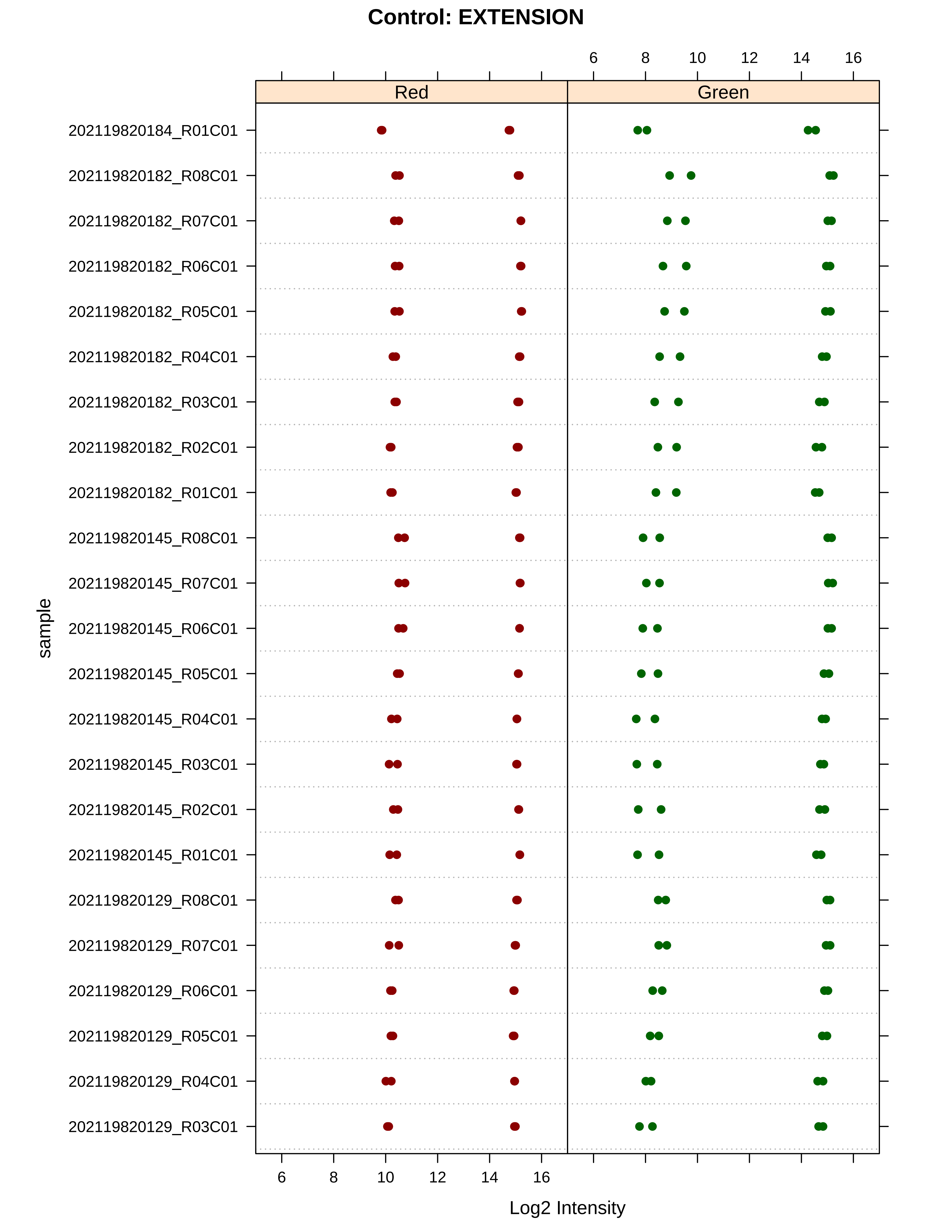
Control: EXTENSION
Red
Green
sample
202119820184_R01C01
202119820182_R08C01
202119820182_R07C01
202119820182_R06C01
202119820182_R05C01
202119820182_R04C01
202119820182_R03C01
202119820182_R02C01
202119820182_R01C01
202119820145_R08C01
202119820145_R07C01
202119820145_R06C01
202119820145_R05C01
202119820145_R04C01
202119820145_R03C01
202119820145_R02C01
202119820145_R01C01
202119820129_R08C01
202119820129_R07C01
202119820129_R06C01
202119820129_R05C01
202119820129_R04C01
202119820129_R03C01
6
8
10
12
14
16
Log2 Intensity

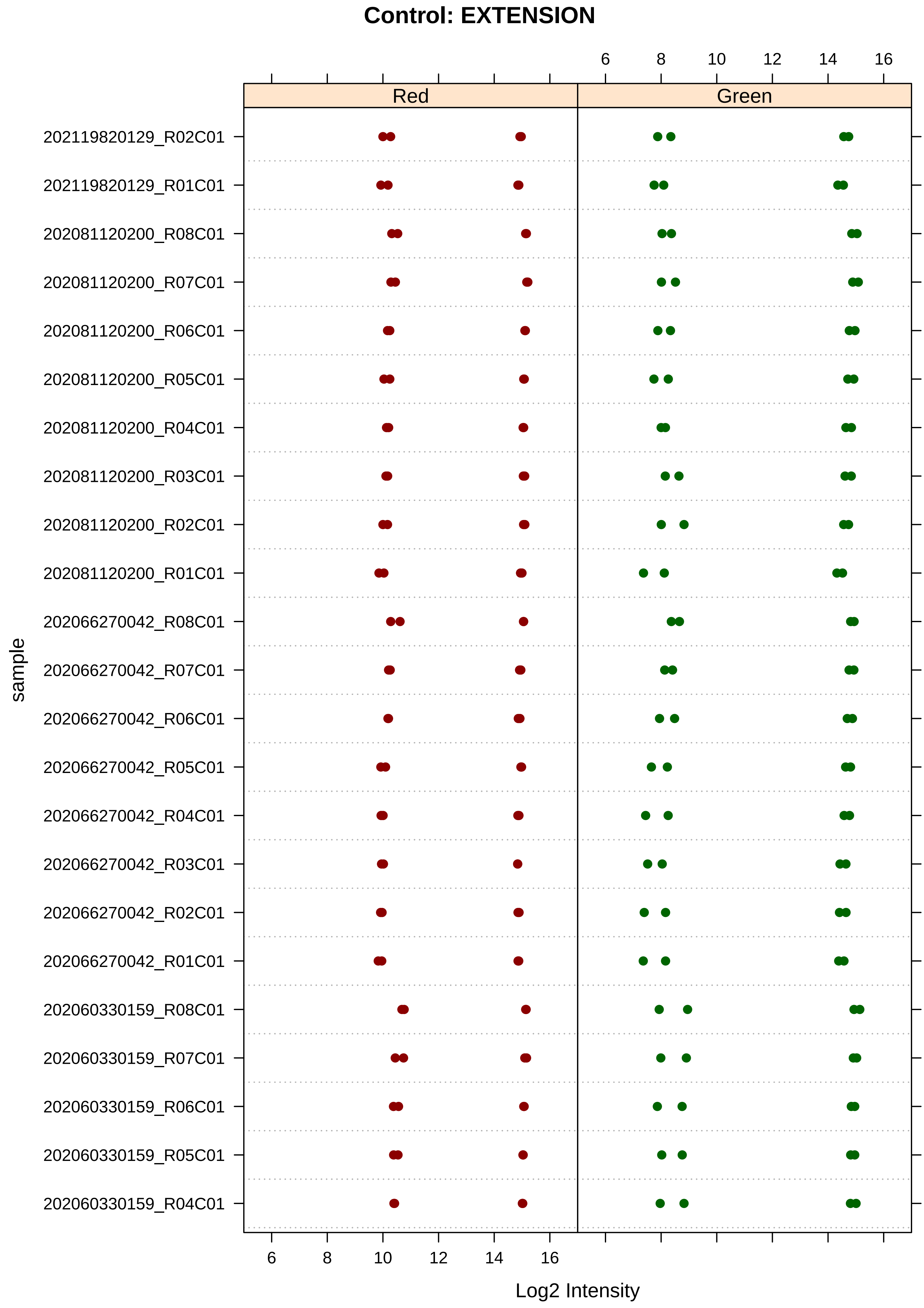

Control: EXTENSION
Red
Green
sample
Log2 Intensity
6
8
10
12
14
16
202119820129_R02C01
202119820129_R01C01
202081120200_R08C01
202081120200_R07C01
202081120200_R06C01
202081120200_R05C01
202081120200_R04C01
202081120200_R03C01
202081120200_R02C01
202081120200_R01C01
202066270042_R08C01
202066270042_R07C01
202066270042_R06C01
202066270042_R05C01
202066270042_R04C01
202066270042_R03C01
202066270042_R02C01
202066270042_R01C01
202060330159_R08C01
202060330159_R07C01
202060330159_R06C01
202060330159_R05C01
202060330159_R04C01

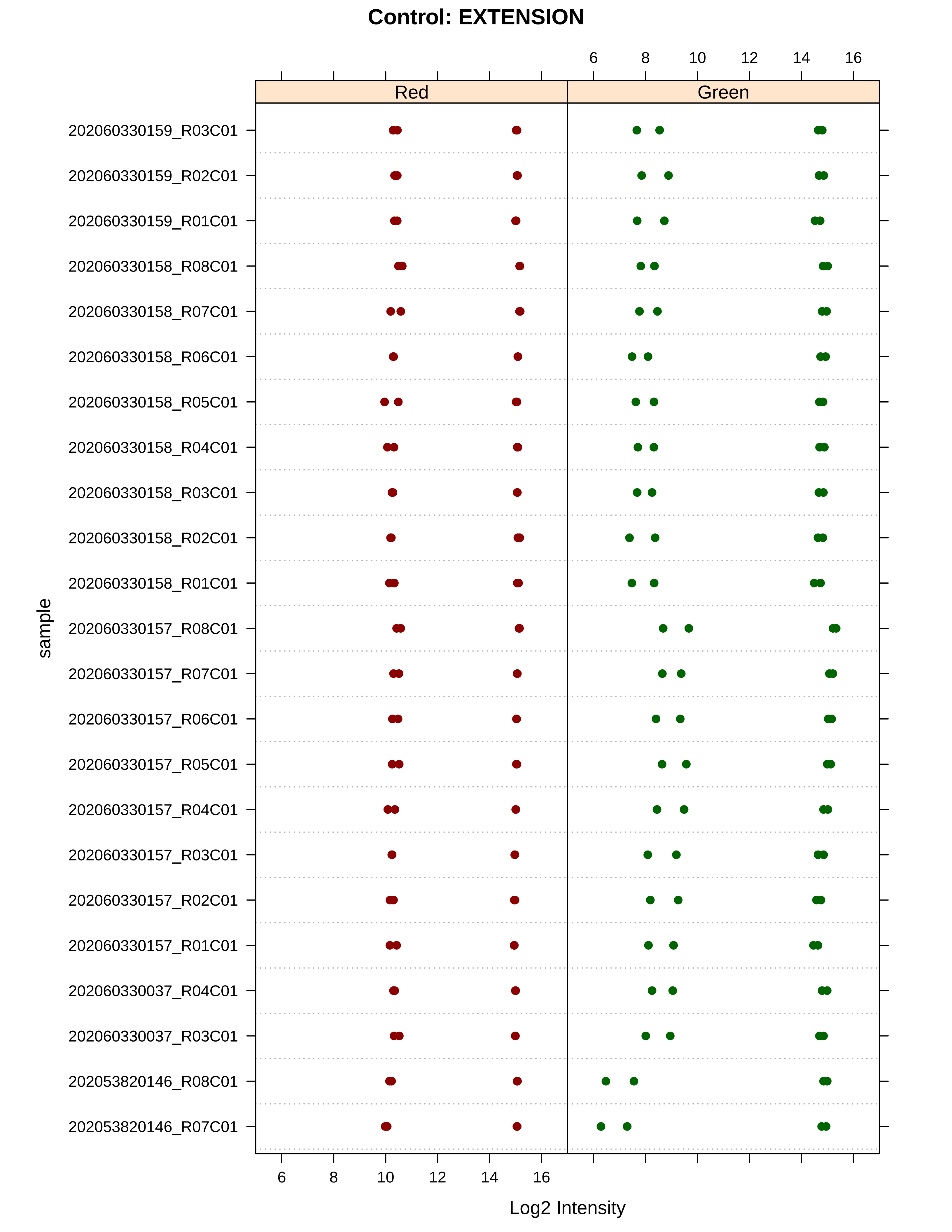

Control: EXTENSION
Red
Green
sample
Log2 Intensity
202060330159_R03C01
202060330159_R02C01
202060330159_R01C01
202060330158_R08C01
202060330158_R07C01
202060330158_R06C01
202060330158_R05C01
202060330158_R04C01
202060330158_R03C01
202060330158_R02C01
202060330158_R01C01
202060330157_R08C01
202060330157_R07C01
202060330157_R06C01
202060330157_R05C01
202060330157_R04C01
202060330157_R03C01
202060330157_R02C01
202060330157_R01C01
202060330037_R04C01
202060330037_R03C01
202053820146_R08C01
202053820146_R07C01
6
8
10
12
14
16

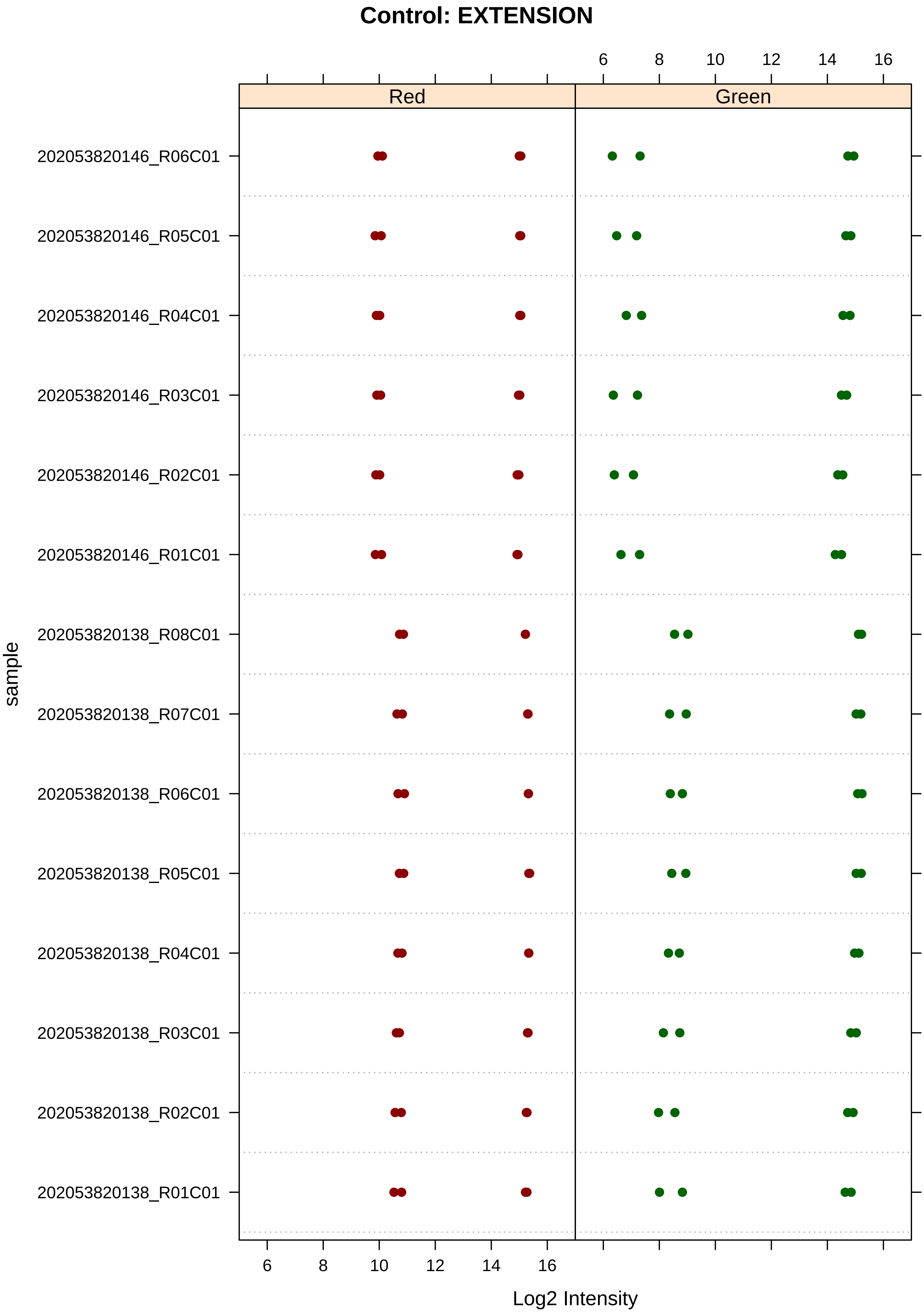
Control: EXTENSION
Red
Green
sample
202053820146_R06C01
202053820146_R05C01
202053820146_R04C01
202053820146_R03C01
202053820146_R02C01
202053820146_R01C01
202053820138_R08C01
202053820138_R07C01
202053820138_R06C01
202053820138_R05C01
202053820138_R04C01
202053820138_R03C01
202053820138_R02C01
202053820138_R01C01
6
8
10
12
14
16
Log2 Intensity

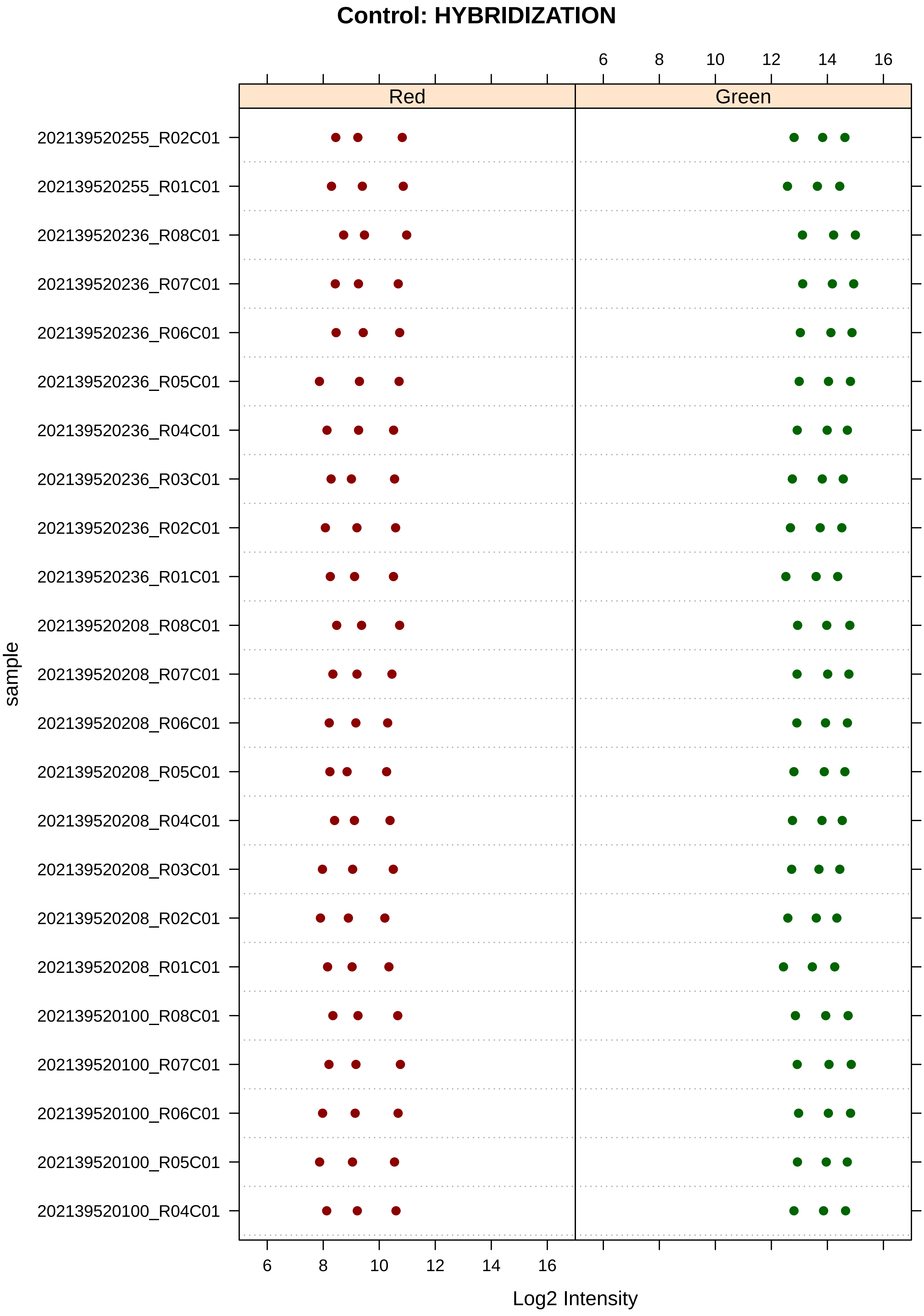
Control: HYBRIDIZATION
Red
Green
sample
Log2 Intensity
6
8
10
12
14
16
202139520255_R02C01
202139520255_R01C01
202139520236_R08C01
202139520236_R07C01
202139520236_R06C01
202139520236_R05C01
202139520236_R04C01
202139520236_R03C01
202139520236_R02C01
202139520236_R01C01
202139520208_R08C01
202139520208_R07C01
202139520208_R06C01
202139520208_R05C01
202139520208_R04C01
202139520208_R03C01
202139520208_R02C01
202139520208_R01C01
202139520100_R08C01
202139520100_R07C01
202139520100_R06C01
202139520100_R05C01
202139520100_R04C01

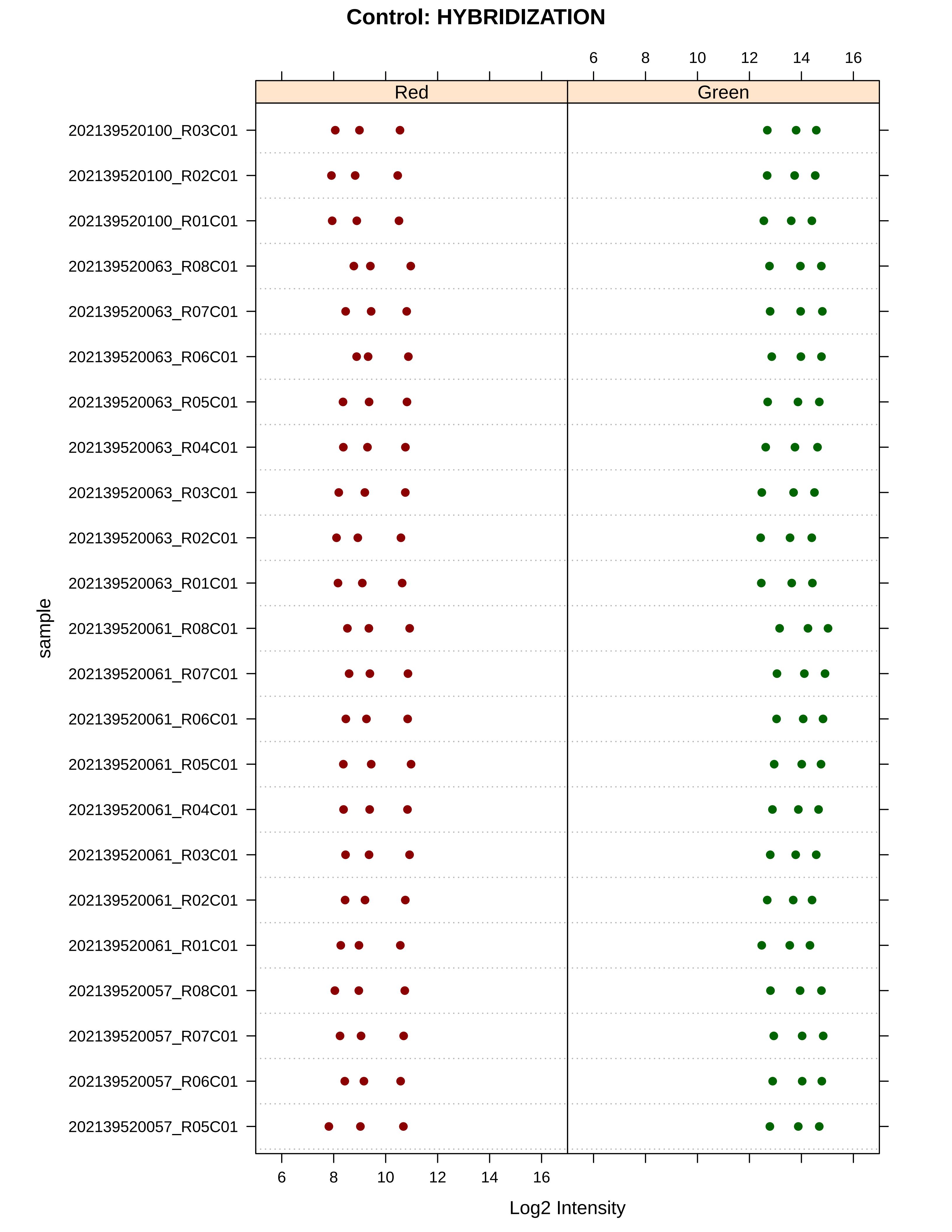
Control: HYBRIDIZATION
Red
Green
sample
Log2 Intensity
202139520100_R03C01
202139520100_R02C01
202139520100_R01C01
202139520063_R08C01
202139520063_R07C01
202139520063_R06C01
202139520063_R05C01
202139520063_R04C01
202139520063_R03C01
202139520063_R02C01
202139520063_R01C01
202139520061_R08C01
202139520061_R07C01
202139520061_R06C01
202139520061_R05C01
202139520061_R04C01
202139520061_R03C01
202139520061_R02C01
202139520061_R01C01
202139520057_R08C01
202139520057_R07C01
202139520057_R06C01
202139520057_R05C01
6
8
10
12
14
16

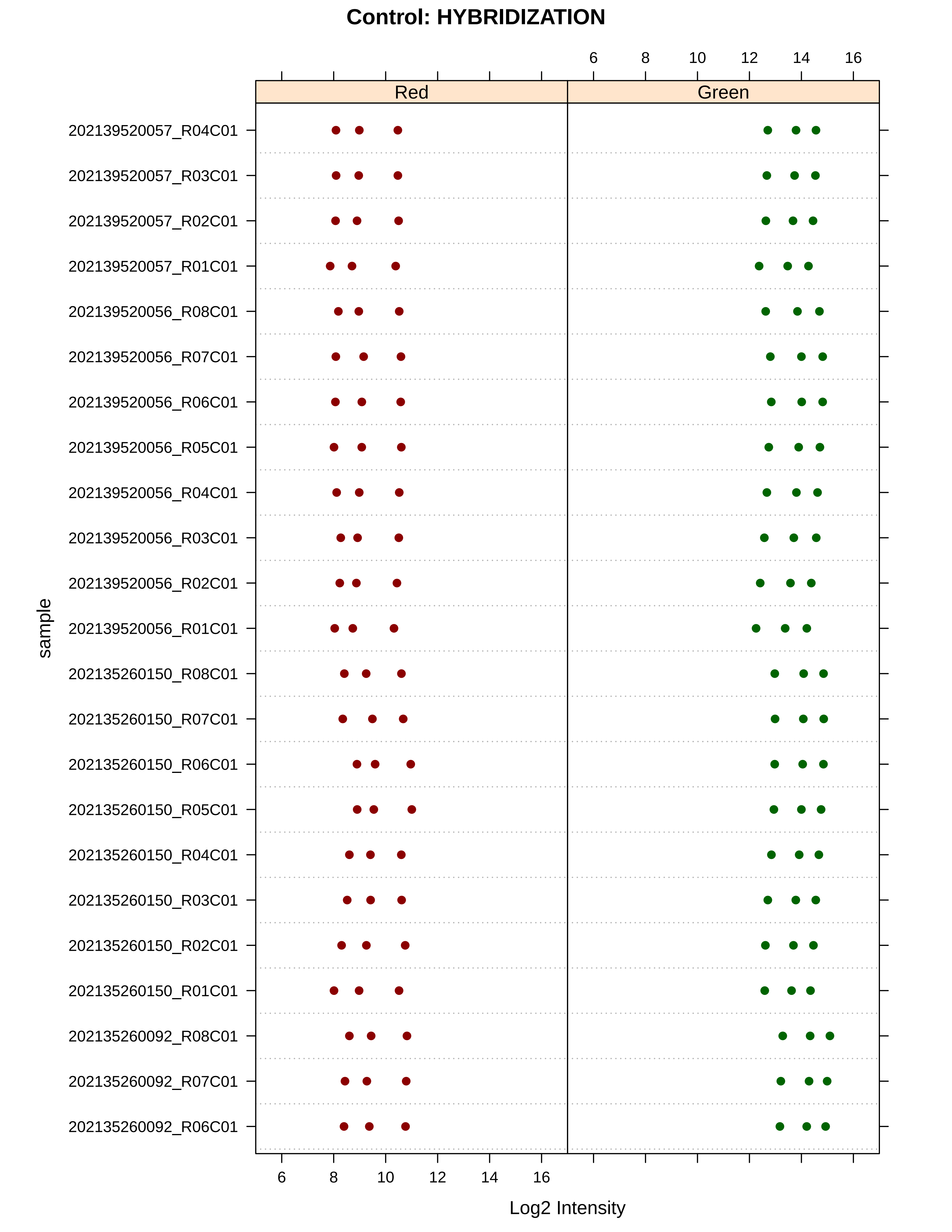

Control: HYBRIDIZATION
Red
Green
202139520057_R04C01
202139520057_R03C01
202139520057_R02C01
202139520057_R01C01
202139520056_R08C01
202139520056_R07C01
202139520056_R06C01
202139520056_R05C01
202139520056_R04C01
202139520056_R03C01
202139520056_R02C01
202139520056_R01C01
202135260150_R08C01
202135260150_R07C01
202135260150_R06C01
202135260150_R05C01
202135260150_R04C01
202135260150_R03C01
202135260150_R02C01
202135260150_R01C01
202135260092_R08C01
202135260092_R07C01
202135260092_R06C01
sample
6
8
10
12
14
16
Log2 Intensity

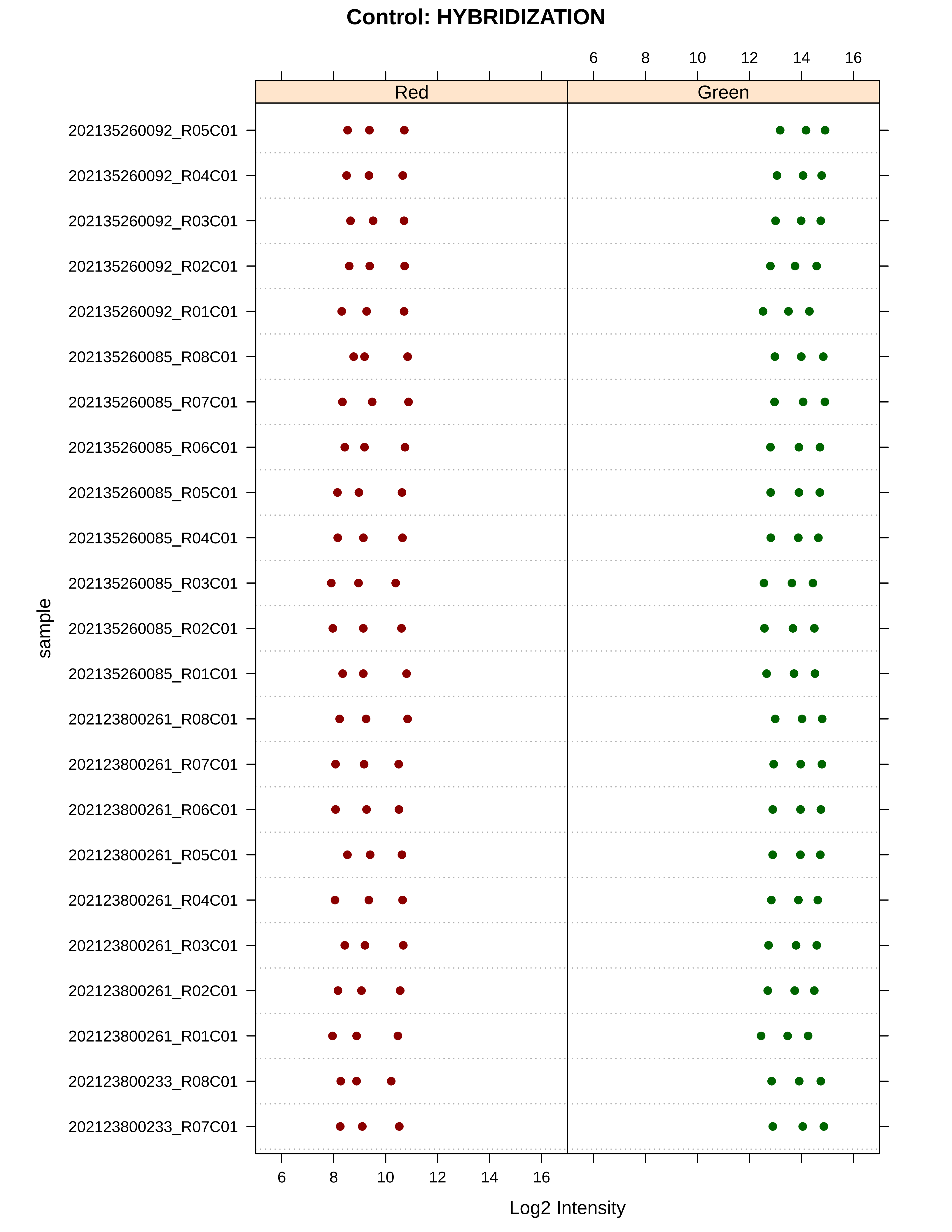

Control: HYBRIDIZATION
Red
Green
sample
Log2 Intensity
6
8
10
12
14
16
202135260092_R05C01
202135260092_R04C01
202135260092_R03C01
202135260092_R02C01
202135260092_R01C01
202135260085_R08C01
202135260085_R07C01
202135260085_R06C01
202135260085_R05C01
202135260085_R04C01
202135260085_R03C01
202135260085_R02C01
202135260085_R01C01
202123800261_R08C01
202123800261_R07C01
202123800261_R06C01
202123800261_R05C01
202123800261_R04C01
202123800261_R03C01
202123800261_R02C01
202123800261_R01C01
202123800233_R08C01
202123800233_R07C01

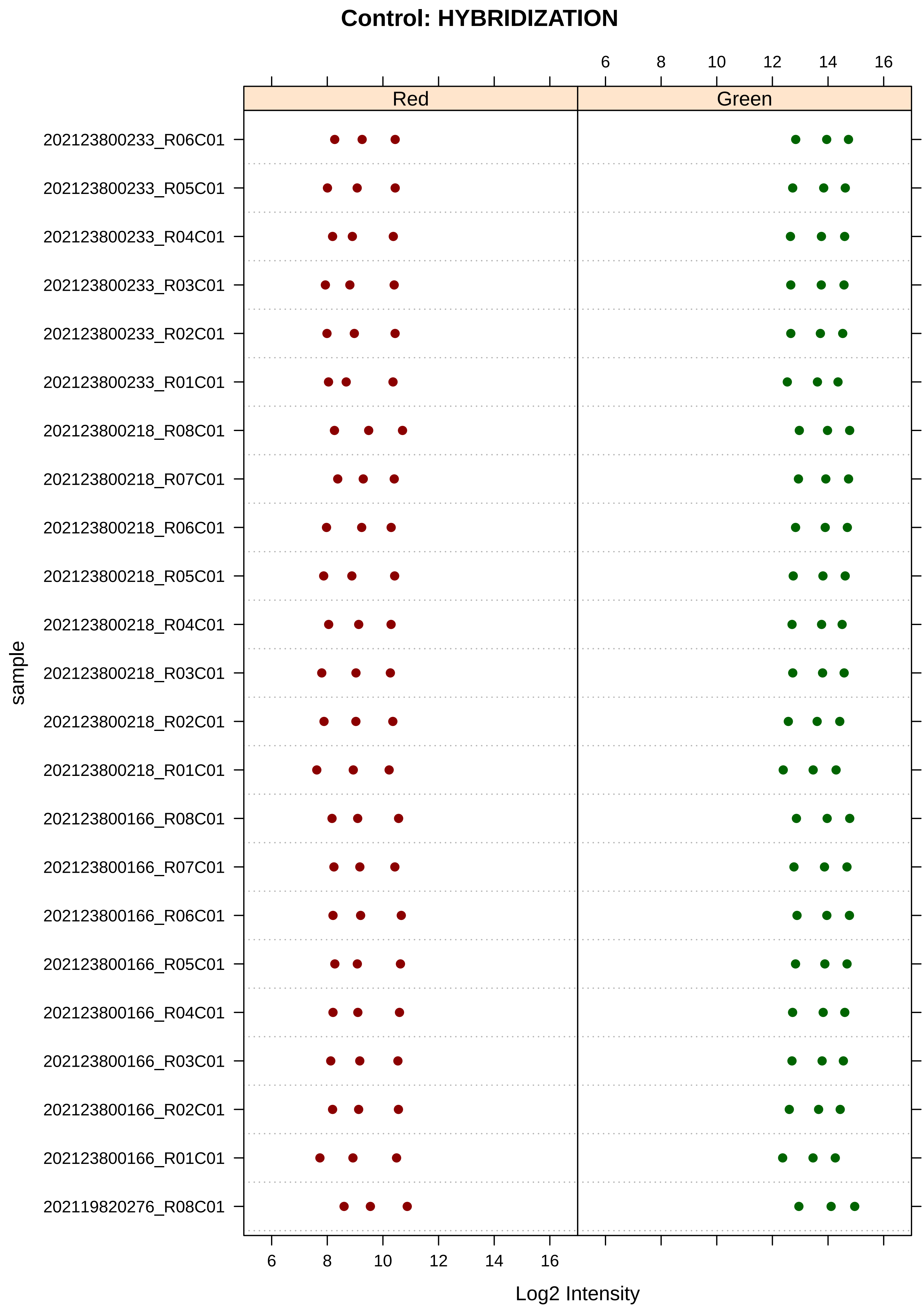
Control: HYBRIDIZATION
Red
Green
sample
Log2 Intensity
6
8
10
12
14
16
202123800233_R06C01
202123800233_R05C01
202123800233_R04C01
202123800233_R03C01
202123800233_R02C01
202123800233_R01C01
202123800218_R08C01
202123800218_R07C01
202123800218_R06C01
202123800218_R05C01
202123800218_R04C01
202123800218_R03C01
202123800218_R02C01
202123800218_R01C01
202123800166_R08C01
202123800166_R07C01
202123800166_R06C01
202123800166_R05C01
202123800166_R04C01
202123800166_R03C01
202123800166_R02C01
202123800166_R01C01
202119820276_R08C01

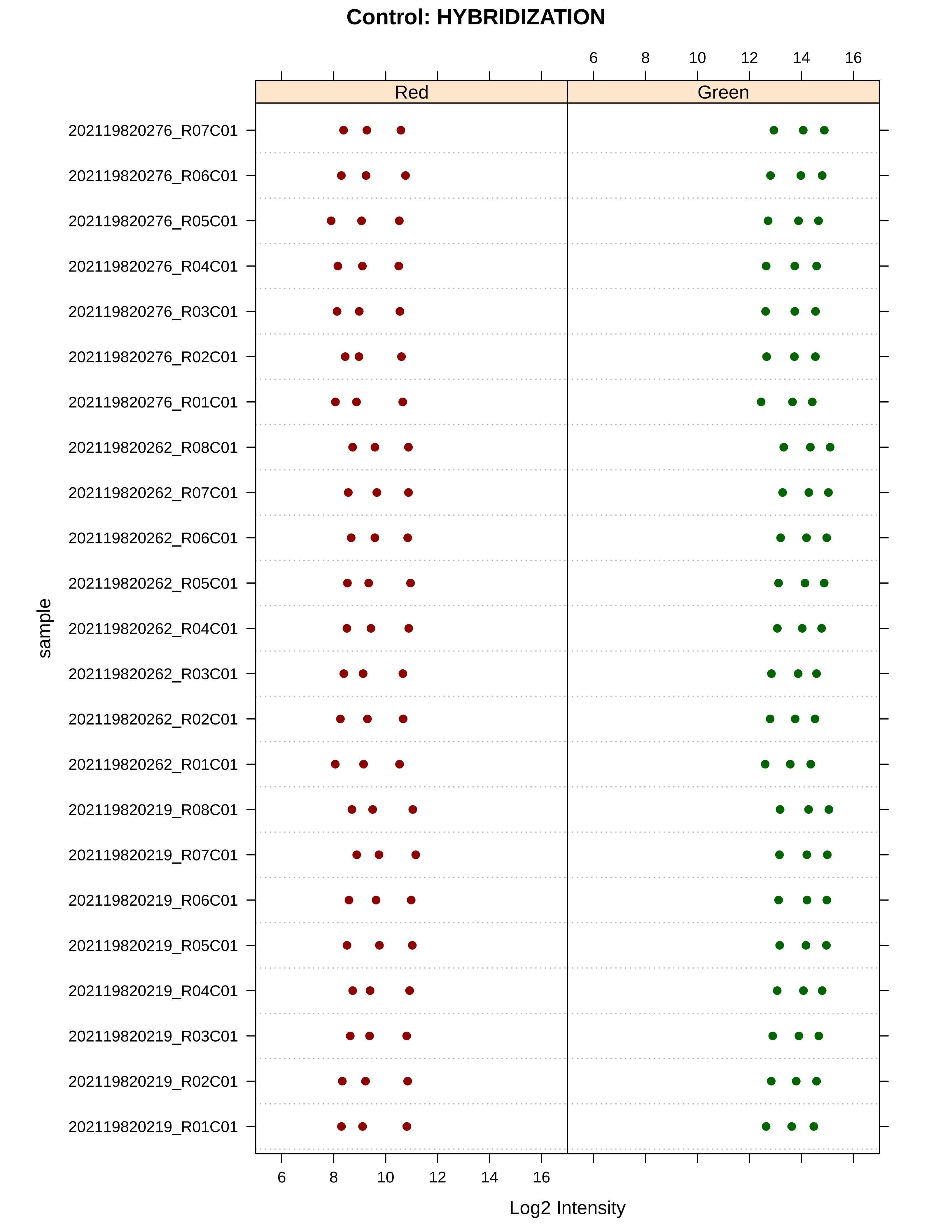

Control: HYBRIDIZATION
Red
Green
sample
Log2 Intensity
6
8
10
12
14
16
202119820276_R07C01
202119820276_R06C01
202119820276_R05C01
202119820276_R04C01
202119820276_R03C01
202119820276_R02C01
202119820276_R01C01
202119820262_R08C01
202119820262_R07C01
202119820262_R06C01
202119820262_R05C01
202119820262_R04C01
202119820262_R03C01
202119820262_R02C01
202119820262_R01C01
202119820219_R08C01
202119820219_R07C01
202119820219_R06C01
202119820219_R05C01
202119820219_R04C01
202119820219_R03C01
202119820219_R02C01
202119820219_R01C01

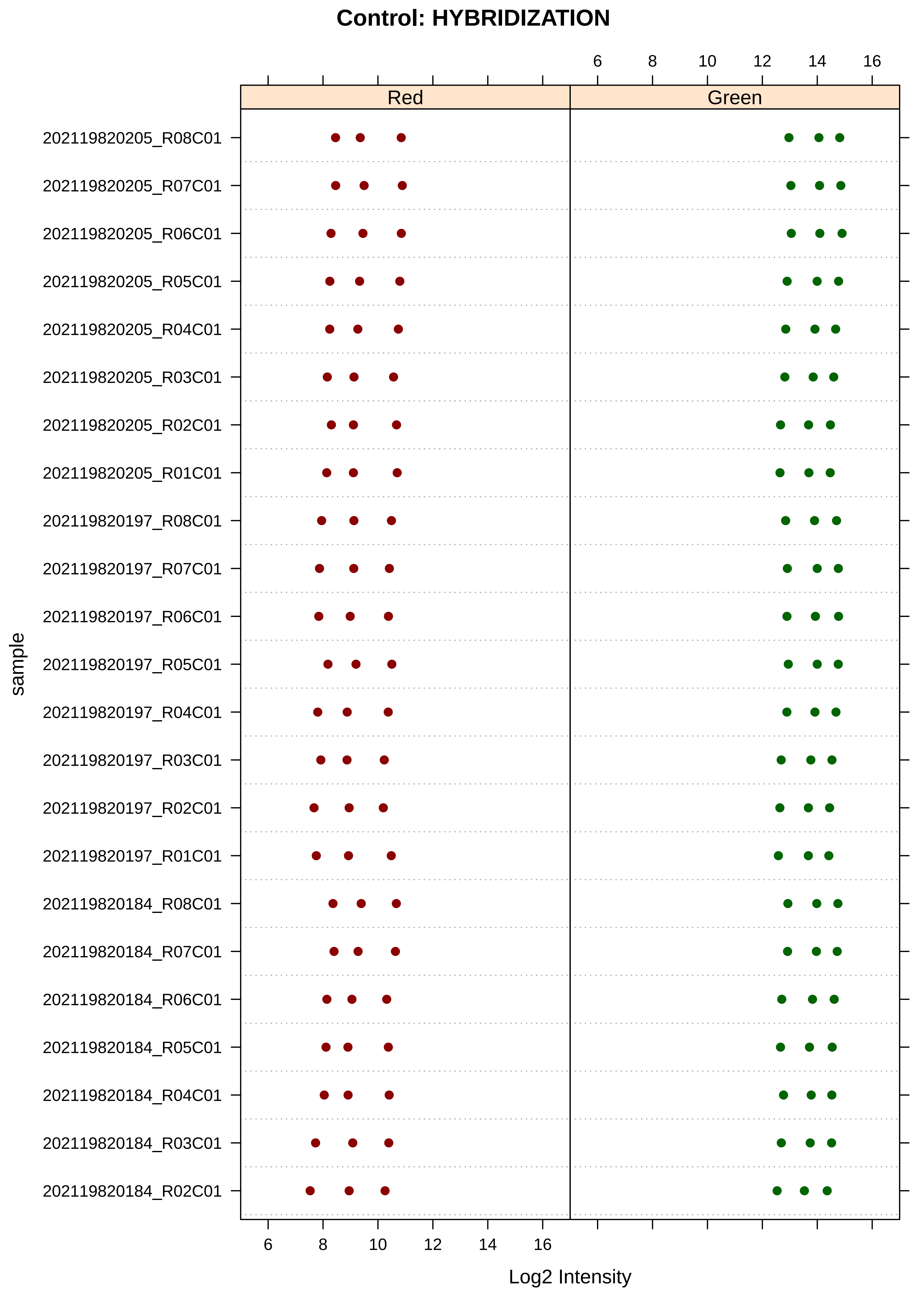

Control: HYBRIDIZATION
Red
Green
sample
Log2 Intensity
6
8
10
12
14
16
202119820205_R08C01
202119820205_R07C01
202119820205_R06C01
202119820205_R05C01
202119820205_R04C01
202119820205_R03C01
202119820205_R02C01
202119820205_R01C01
202119820197_R08C01
202119820197_R07C01
202119820197_R06C01
202119820197_R05C01
202119820197_R04C01
202119820197_R03C01
202119820197_R02C01
202119820197_R01C01
202119820184_R08C01
202119820184_R07C01
202119820184_R06C01
202119820184_R05C01
202119820184_R04C01
202119820184_R03C01
202119820184_R02C01

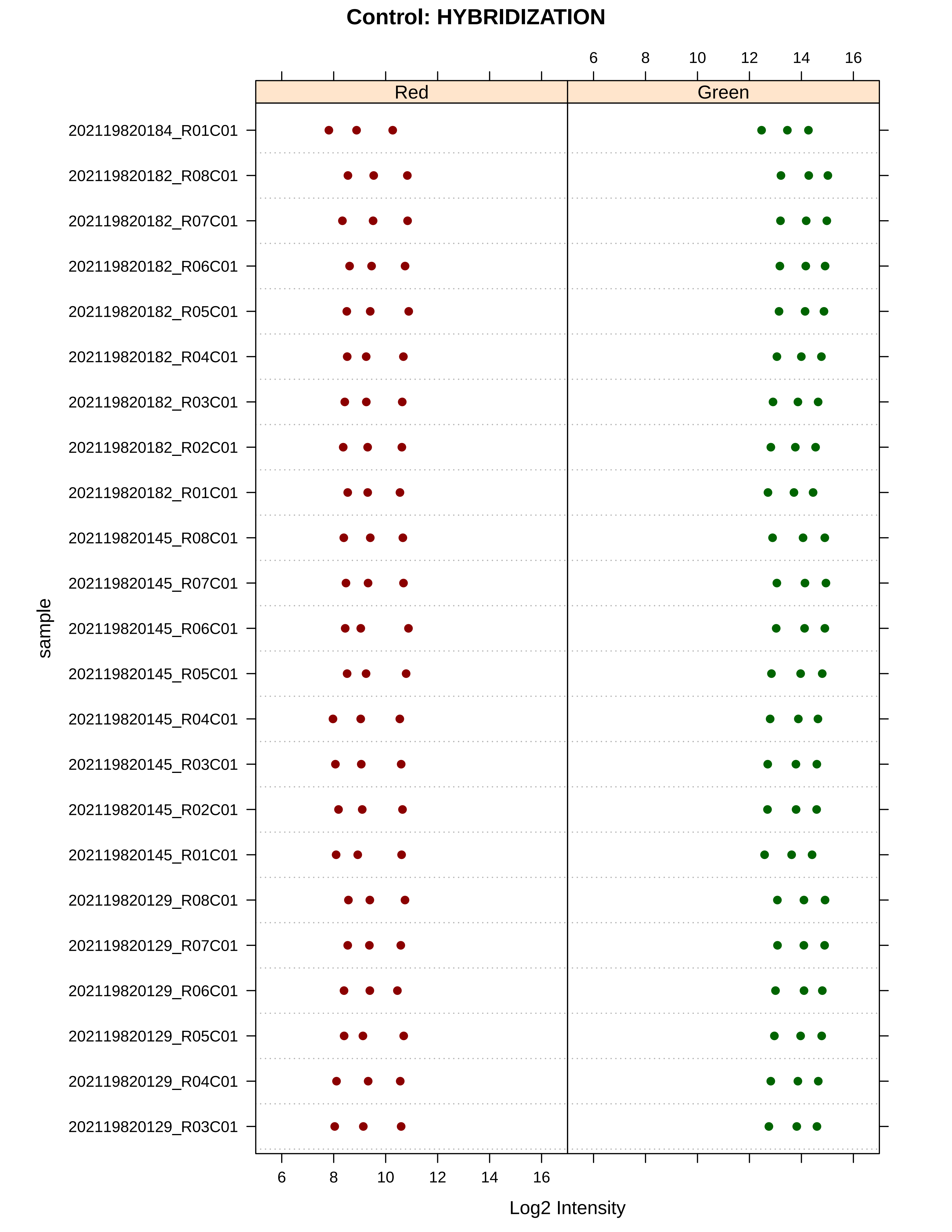
Control: HYBRIDIZATION
Red
Green
sample
Log2 Intensity
6
8
10
12
14
16
202119820184_R01C01
202119820182_R08C01
202119820182_R07C01
202119820182_R06C01
202119820182_R05C01
202119820182_R04C01
202119820182_R03C01
202119820182_R02C01
202119820182_R01C01
202119820145_R08C01
202119820145_R07C01
202119820145_R06C01
202119820145_R05C01
202119820145_R04C01
202119820145_R03C01
202119820145_R02C01
202119820145_R01C01
202119820129_R08C01
202119820129_R07C01
202119820129_R06C01
202119820129_R05C01
202119820129_R04C01
202119820129_R03C01

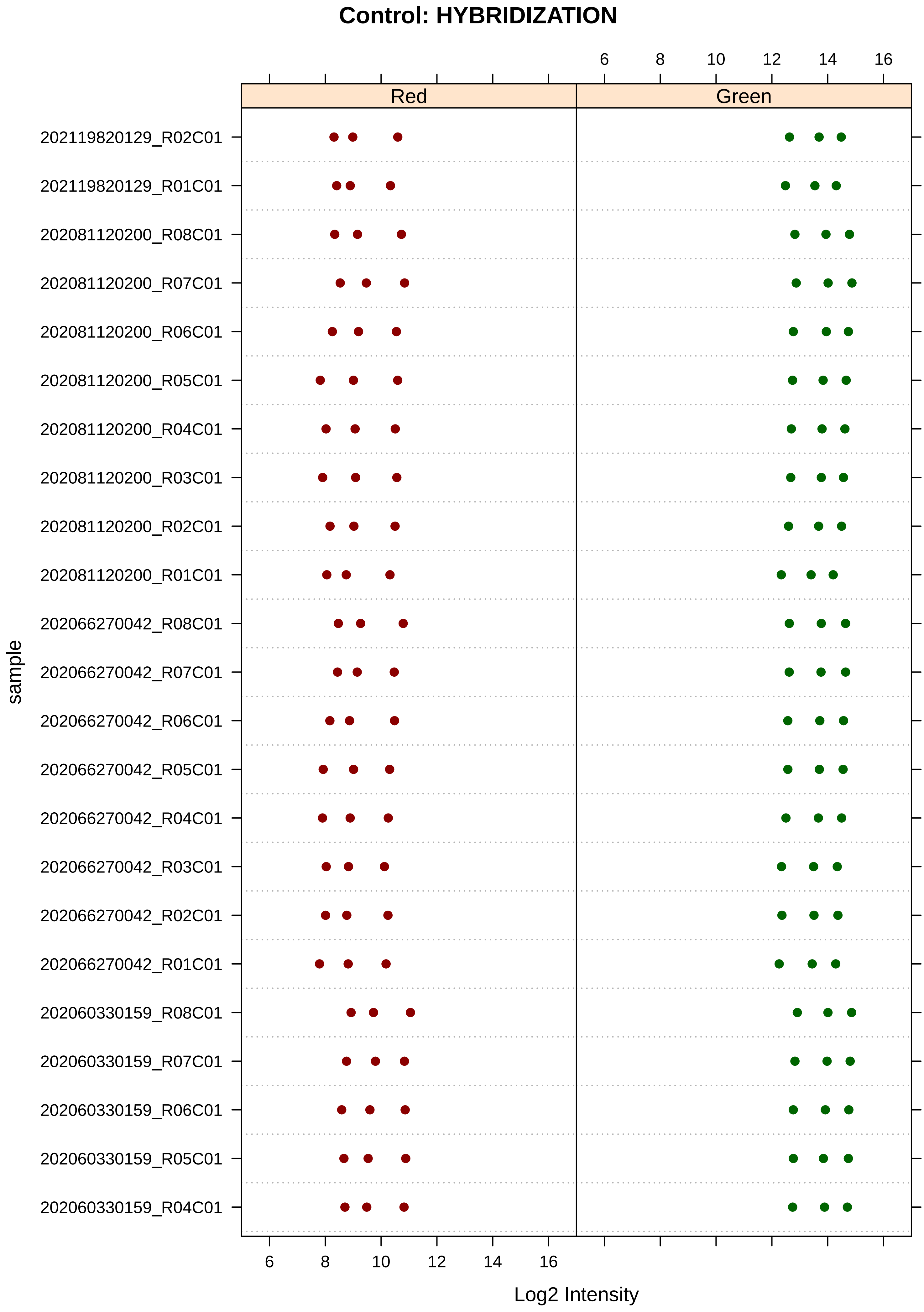
Control: HYBRIDIZATION
Red
Green
sample
Log2 Intensity
6
8
10
12
14
16
202119820129_R02C01
202119820129_R01C01
202081120200_R08C01
202081120200_R07C01
202081120200_R06C01
202081120200_R05C01
202081120200_R04C01
202081120200_R03C01
202081120200_R02C01
202081120200_R01C01
202066270042_R08C01
202066270042_R07C01
202066270042_R06C01
202066270042_R05C01
202066270042_R04C01
202066270042_R03C01
202066270042_R02C01
202066270042_R01C01
202060330159_R08C01
202060330159_R07C01
202060330159_R06C01
202060330159_R05C01
202060330159_R04C01

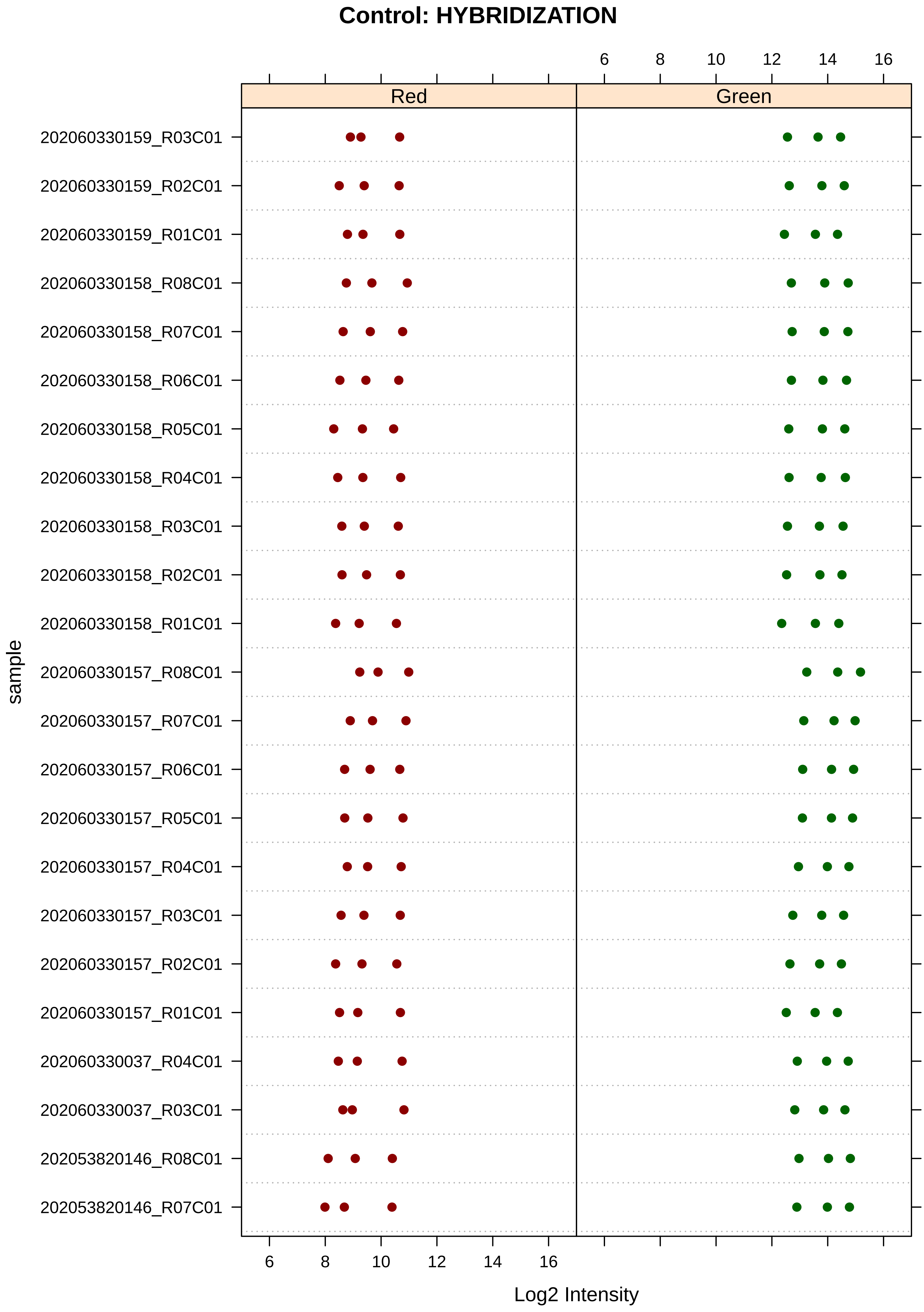

Control: HYBRIDIZATION
Red
Green
sample
Log2 Intensity
202060330159_R03C01
202060330159_R02C01
202060330159_R01C01
202060330158_R08C01
202060330158_R07C01
202060330158_R06C01
202060330158_R05C01
202060330158_R04C01
202060330158_R03C01
202060330158_R02C01
202060330158_R01C01
202060330157_R08C01
202060330157_R07C01
202060330157_R06C01
202060330157_R05C01
202060330157_R04C01
202060330157_R03C01
202060330157_R02C01
202060330157_R01C01
202060330037_R04C01
202060330037_R03C01
202053820146_R08C01
202053820146_R07C01
6
8
10
12
14
16

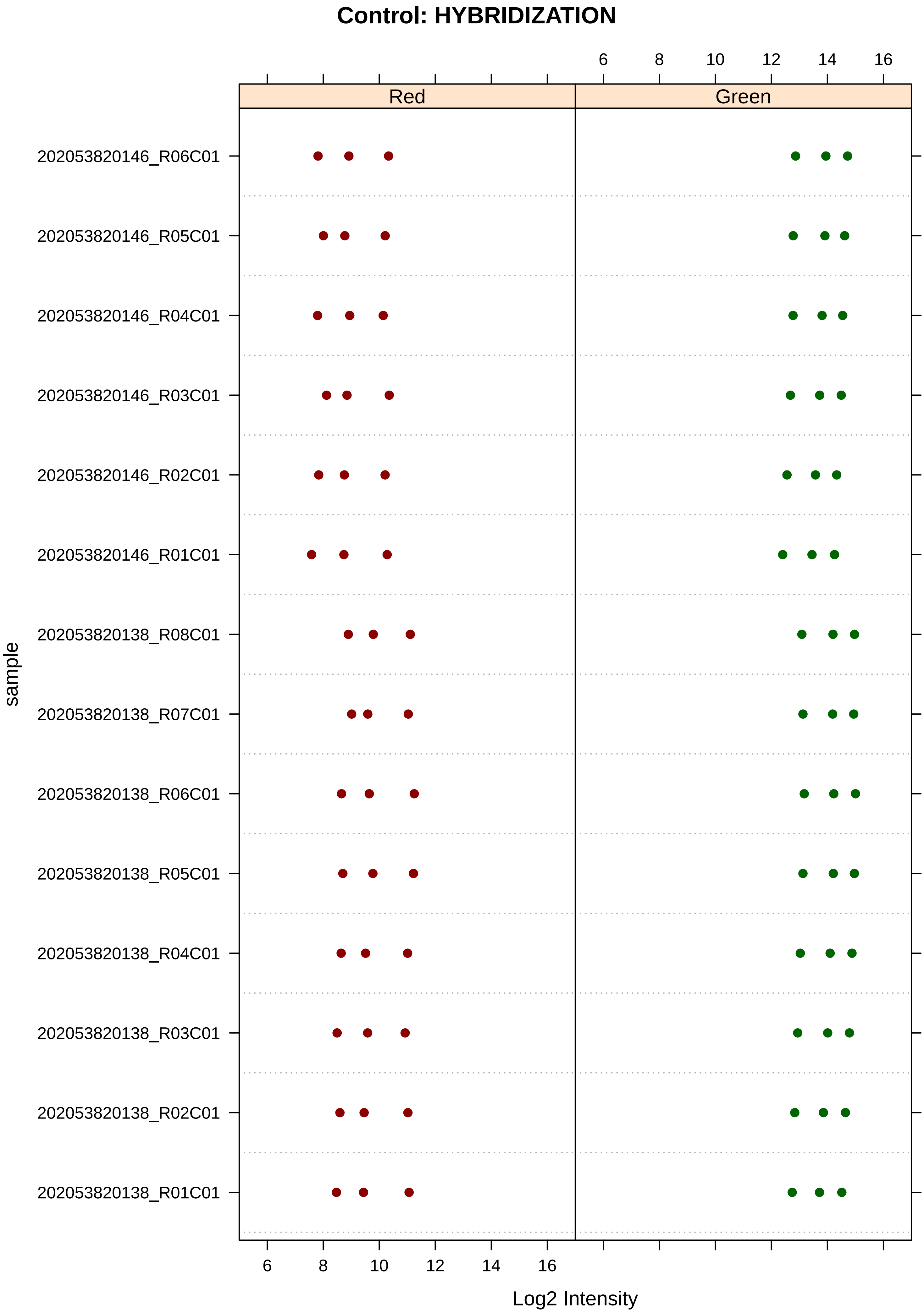

Control: HYBRIDIZATION
Red
Green
sample
202053820146_R06C01
202053820146_R05C01
202053820146_R04C01
202053820146_R03C01
202053820146_R02C01
202053820146_R01C01
202053820138_R08C01
202053820138_R07C01
202053820138_R06C01
202053820138_R05C01
202053820138_R04C01
202053820138_R03C01
202053820138_R02C01
202053820138_R01C01
6 8 10 12 14 16
Log2 Intensity

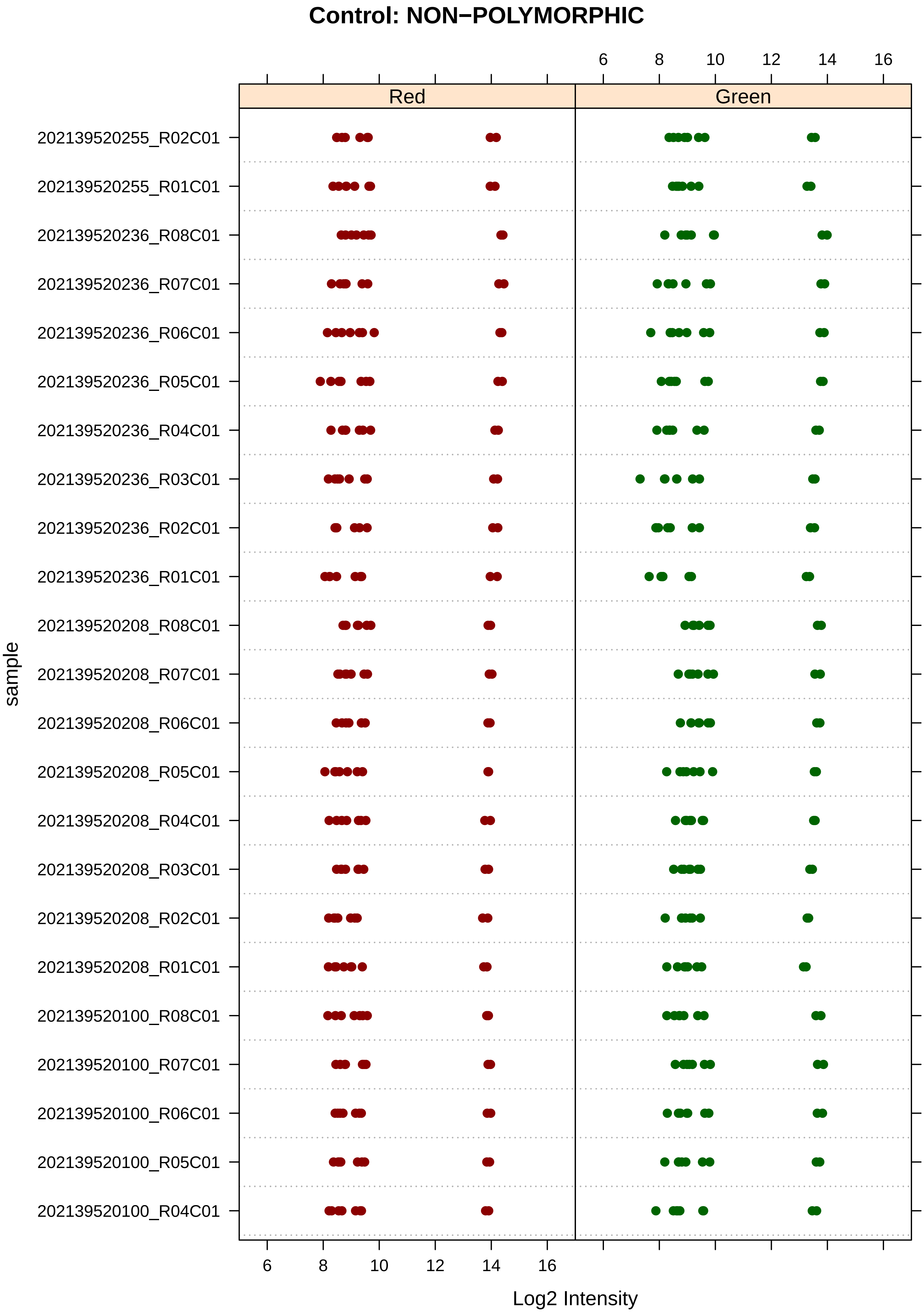

Control: NON−POLYMORPHIC
Red
Green
sample
202139520255_R02C01
202139520255_R01C01
202139520236_R08C01
202139520236_R07C01
202139520236_R06C01
202139520236_R05C01
202139520236_R04C01
202139520236_R03C01
202139520236_R02C01
202139520236_R01C01
202139520208_R08C01
202139520208_R07C01
202139520208_R06C01
202139520208_R05C01
202139520208_R04C01
202139520208_R03C01
202139520208_R02C01
202139520208_R01C01
202139520100_R08C01
202139520100_R07C01
202139520100_R06C01
202139520100_R05C01
202139520100_R04C01
6 8 10 12 14 16
Log2 Intensity

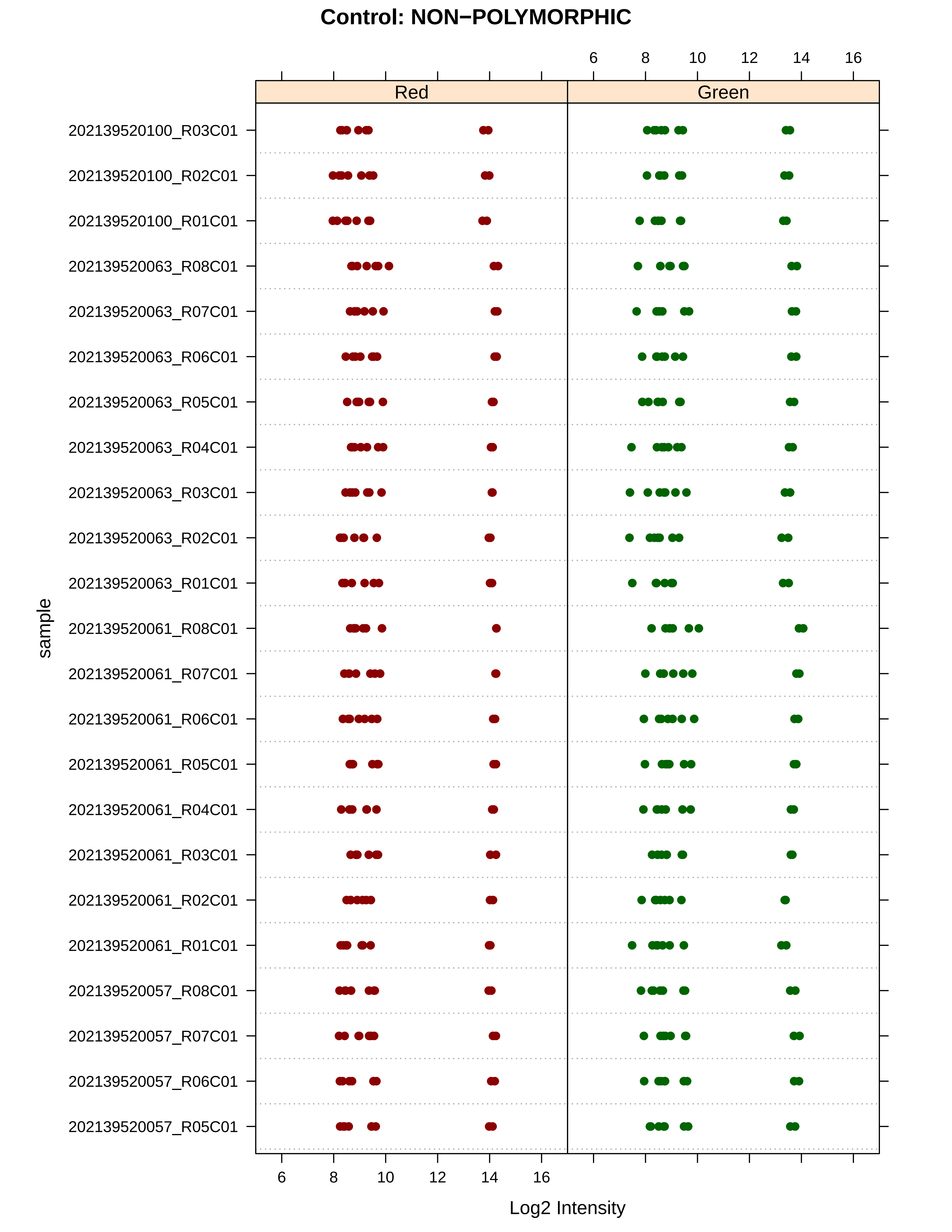

Control: NON−POLYMORPHIC
Red
Green
sample
Log2 Intensity
202139520100_R03C01
202139520100_R02C01
202139520100_R01C01
202139520063_R08C01
202139520063_R07C01
202139520063_R06C01
202139520063_R05C01
202139520063_R04C01
202139520063_R03C01
202139520063_R02C01
202139520063_R01C01
202139520061_R08C01
202139520061_R07C01
202139520061_R06C01
202139520061_R05C01
202139520061_R04C01
202139520061_R03C01
202139520061_R02C01
202139520061_R01C01
202139520057_R08C01
202139520057_R07C01
202139520057_R06C01
202139520057_R05C01
6
8
10
12
14
16

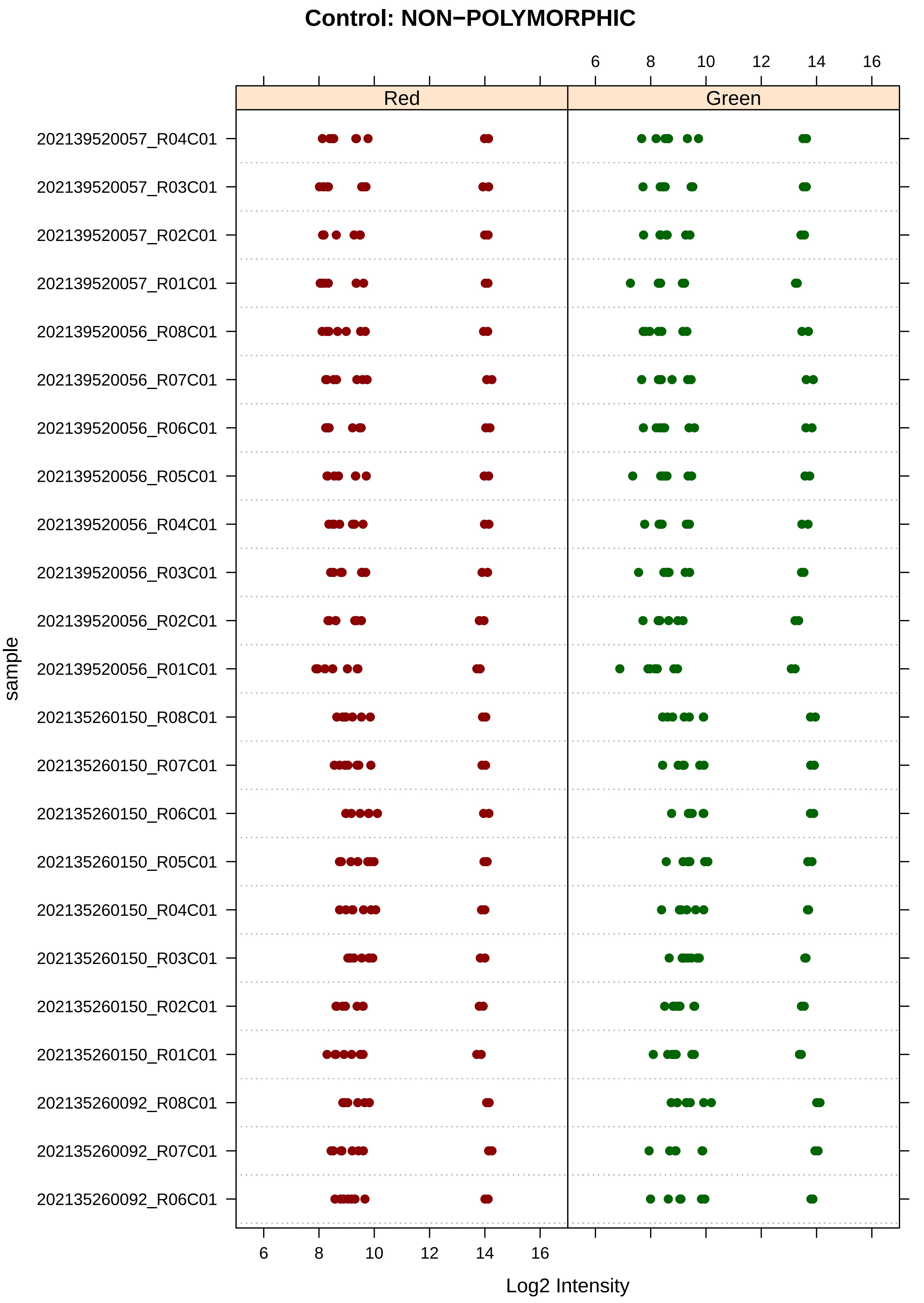

Control: NON−POLYMORPHIC
Red
Green
sample
Log2 Intensity
202139520057_R04C01
202139520057_R03C01
202139520057_R02C01
202139520057_R01C01
202139520056_R08C01
202139520056_R07C01
202139520056_R06C01
202139520056_R05C01
202139520056_R04C01
202139520056_R03C01
202139520056_R02C01
202139520056_R01C01
202135260150_R08C01
202135260150_R07C01
202135260150_R06C01
202135260150_R05C01
202135260150_R04C01
202135260150_R03C01
202135260150_R02C01
202135260150_R01C01
202135260092_R08C01
202135260092_R07C01
202135260092_R06C01
6
8
10
12
14
16

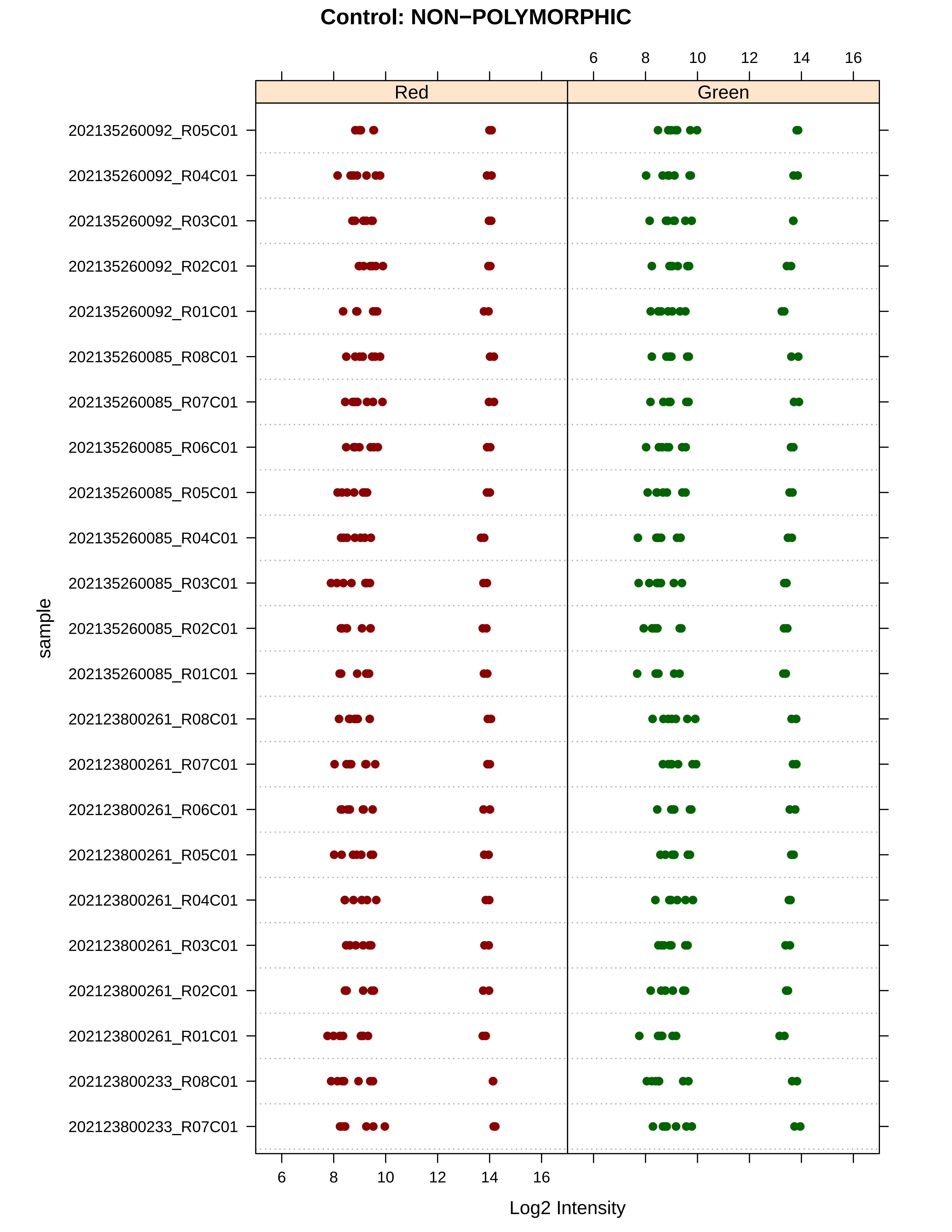
Control: NON−POLYMORPHIC
Red
Green
sample
Log2 Intensity
6
8
10
12
14
16
202135260092_R05C01
202135260092_R04C01
202135260092_R03C01
202135260092_R02C01
202135260092_R01C01
202135260085_R08C01
202135260085_R07C01
202135260085_R06C01
202135260085_R05C01
202135260085_R04C01
202135260085_R03C01
202135260085_R02C01
202135260085_R01C01
202123800261_R08C01
202123800261_R07C01
202123800261_R06C01
202123800261_R05C01
202123800261_R04C01
202123800261_R03C01
202123800261_R02C01
202123800261_R01C01
202123800233_R08C01
202123800233_R07C01

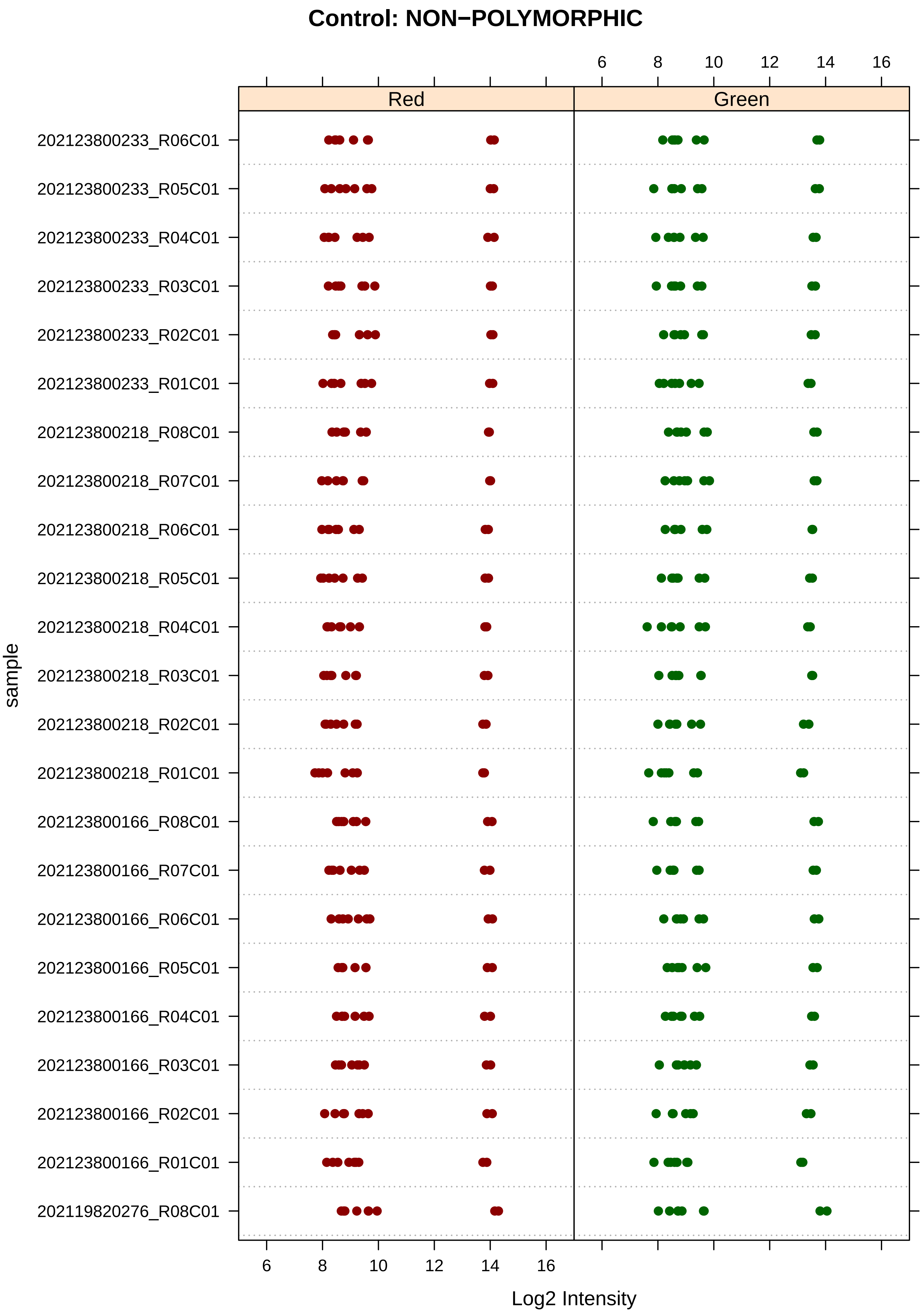

Control: NON−POLYMORPHIC
Red
Green
sample
Log2 Intensity
6
8
10
12
14
16
202123800233_R06C01
202123800233_R05C01
202123800233_R04C01
202123800233_R03C01
202123800233_R02C01
202123800233_R01C01
202123800218_R08C01
202123800218_R07C01
202123800218_R06C01
202123800218_R05C01
202123800218_R04C01
202123800218_R03C01
202123800218_R02C01
202123800218_R01C01
202123800166_R08C01
202123800166_R07C01
202123800166_R06C01
202123800166_R05C01
202123800166_R04C01
202123800166_R03C01
202123800166_R02C01
202123800166_R01C01
202119820276_R08C01

Control: NON−POLYMORPHIC

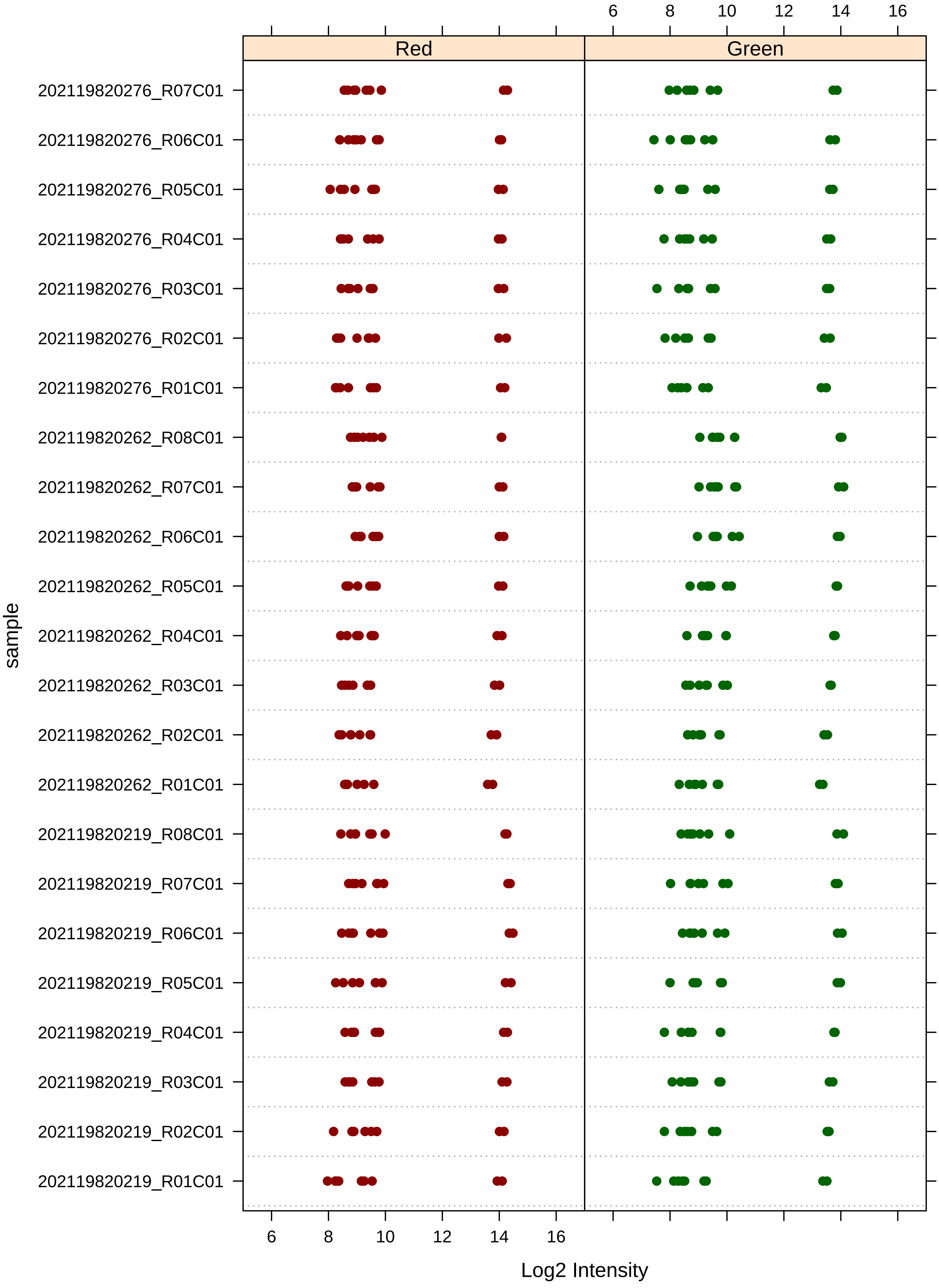

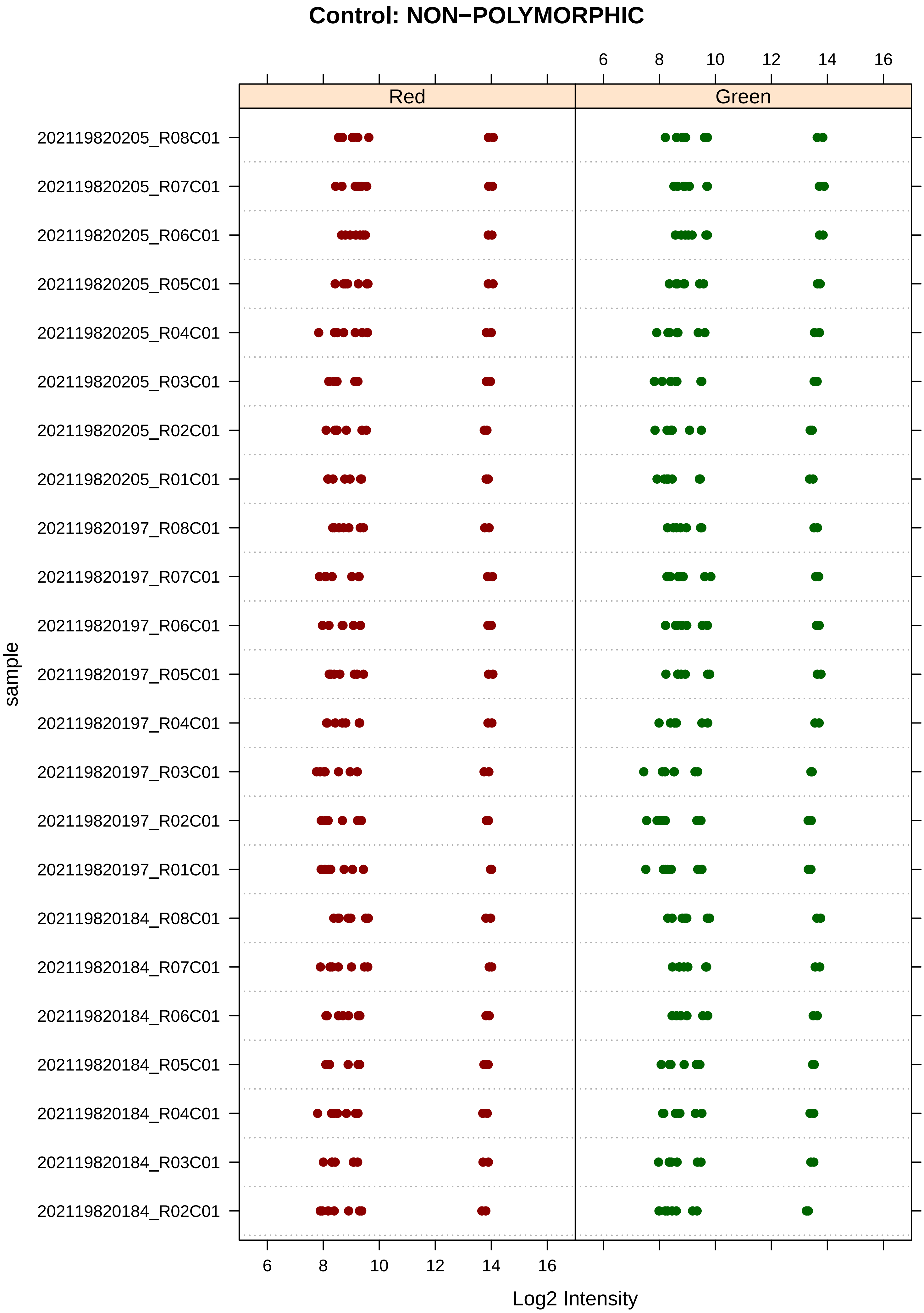

Control: NON−POLYMORPHIC
Red
Green
sample
Log2 Intensity
6
8
10
12
14
16
202119820205_R08C01
202119820205_R07C01
202119820205_R06C01
202119820205_R05C01
202119820205_R04C01
202119820205_R03C01
202119820205_R02C01
202119820205_R01C01
202119820197_R08C01
202119820197_R07C01
202119820197_R06C01
202119820197_R05C01
202119820197_R04C01
202119820197_R03C01
202119820197_R02C01
202119820197_R01C01
202119820184_R08C01
202119820184_R07C01
202119820184_R06C01
202119820184_R05C01
202119820184_R04C01
202119820184_R03C01
202119820184_R02C01

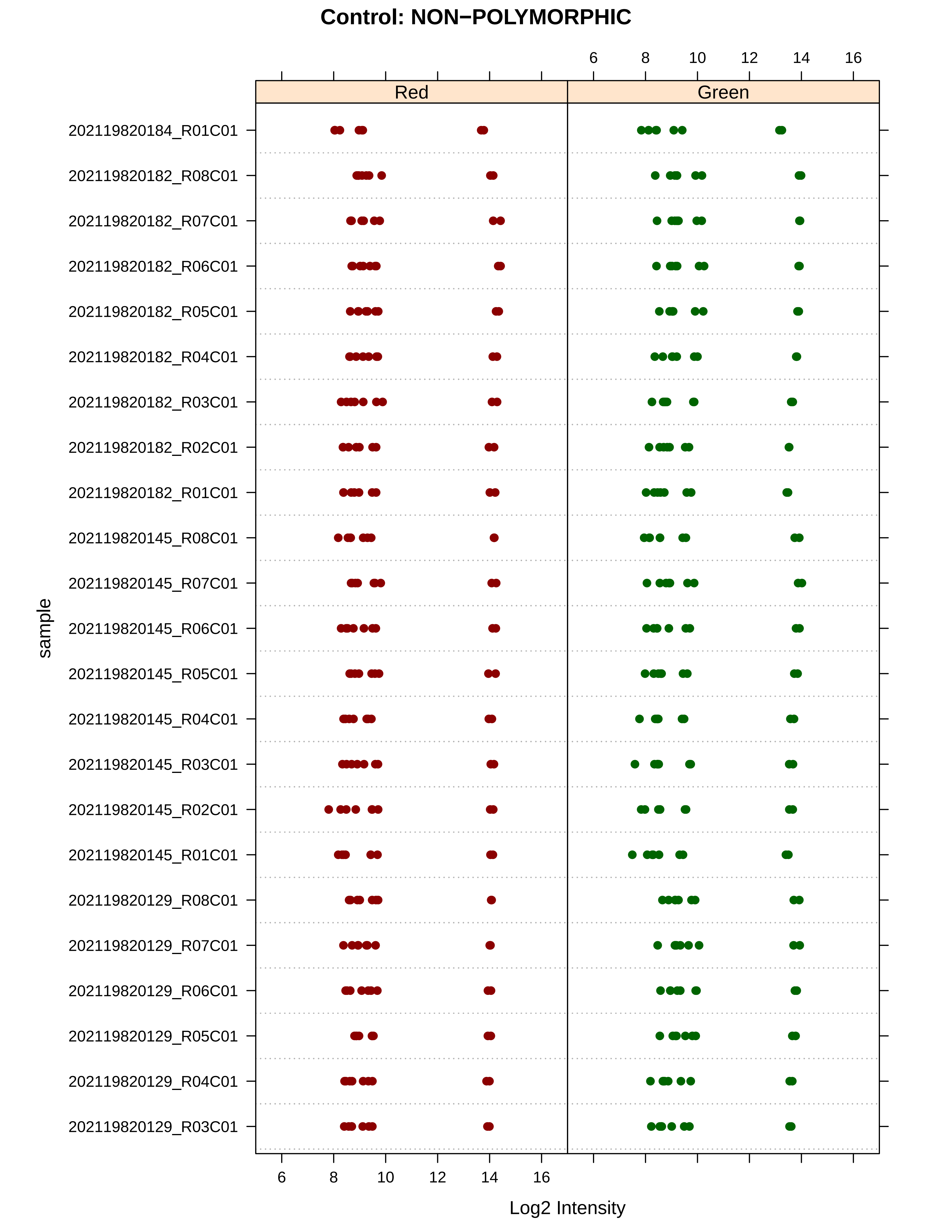

Control: NON−POLYMORPHIC
Red
Green
202119820184_R01C01
202119820182_R08C01
202119820182_R07C01
202119820182_R06C01
202119820182_R05C01
202119820182_R04C01
202119820182_R03C01
202119820182_R02C01
202119820182_R01C01
202119820145_R08C01
202119820145_R07C01
202119820145_R06C01
202119820145_R05C01
202119820145_R04C01
202119820145_R03C01
202119820145_R02C01
202119820145_R01C01
202119820129_R08C01
202119820129_R07C01
202119820129_R06C01
202119820129_R05C01
202119820129_R04C01
202119820129_R03C01
sample
6
8
10
12
14
16
Log2 Intensity

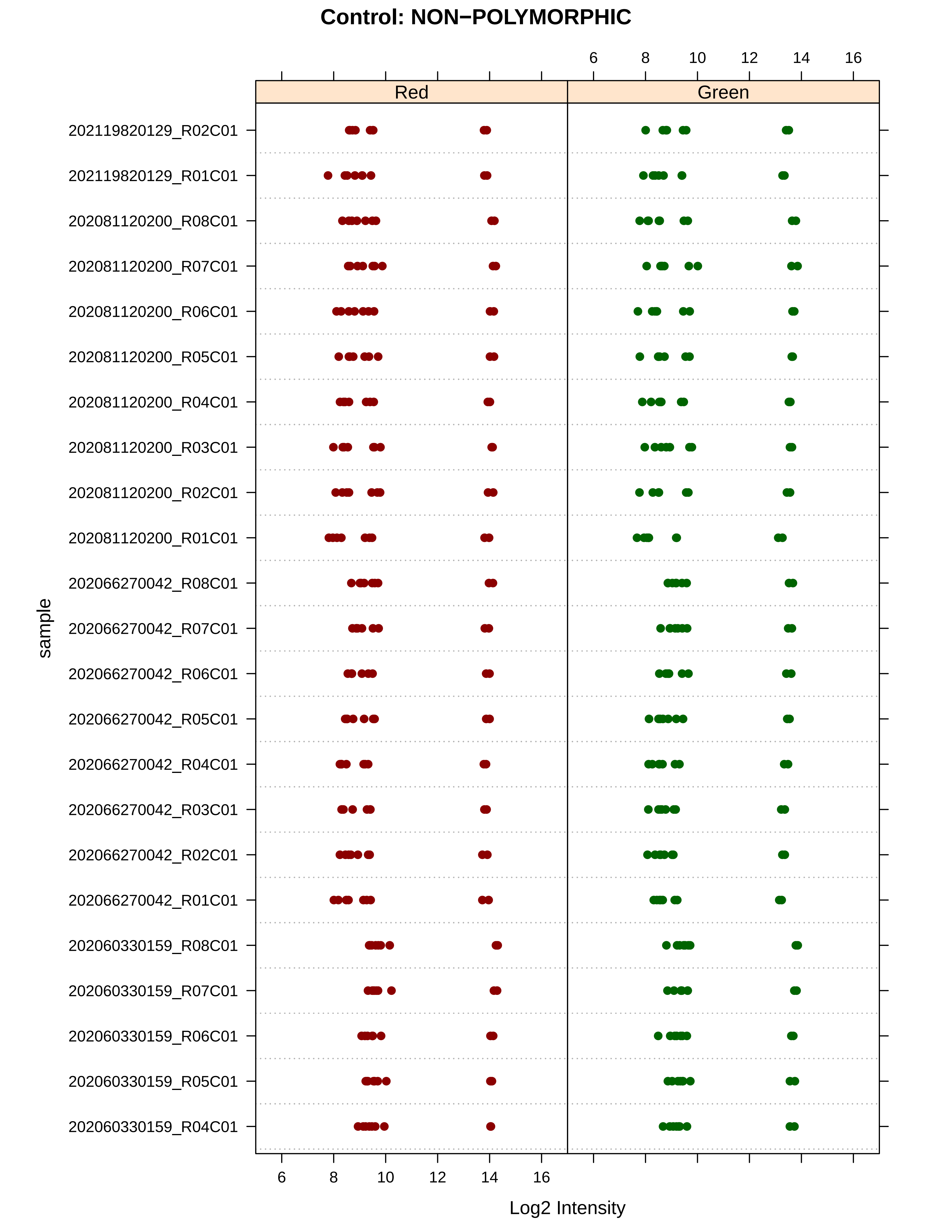

Control: NON−POLYMORPHIC
Red
Green
sample
202119820129_R02C01
202119820129_R01C01
202081120200_R08C01
202081120200_R07C01
202081120200_R06C01
202081120200_R05C01
202081120200_R04C01
202081120200_R03C01
202081120200_R02C01
202081120200_R01C01
202066270042_R08C01
202066270042_R07C01
202066270042_R06C01
202066270042_R05C01
202066270042_R04C01
202066270042_R03C01
202066270042_R02C01
202066270042_R01C01
202060330159_R08C01
202060330159_R07C01
202060330159_R06C01
202060330159_R05C01
202060330159_R04C01
6
8
10
12
14
16
Log2 Intensity

Control: NON−POLYMORPHIC

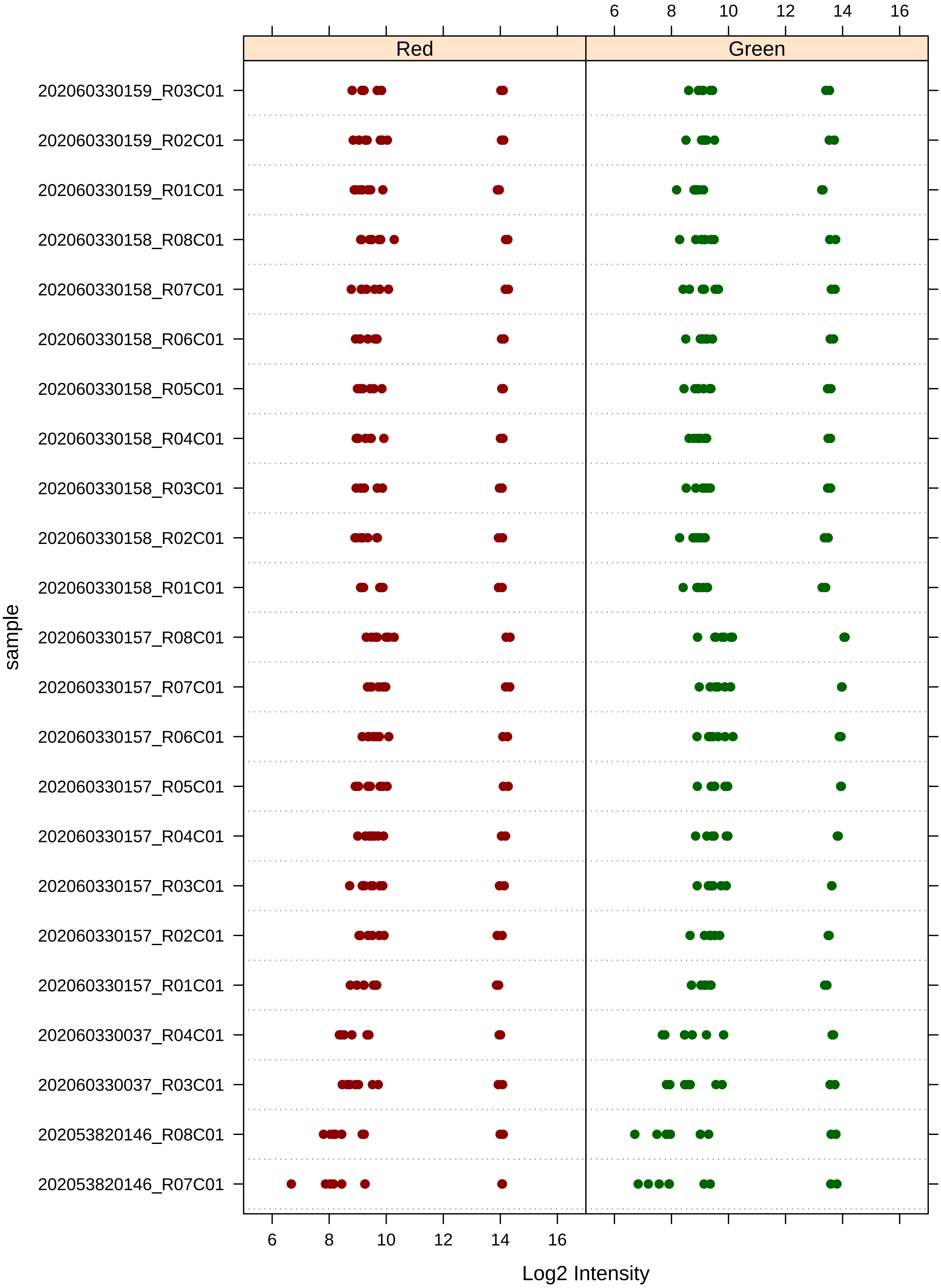

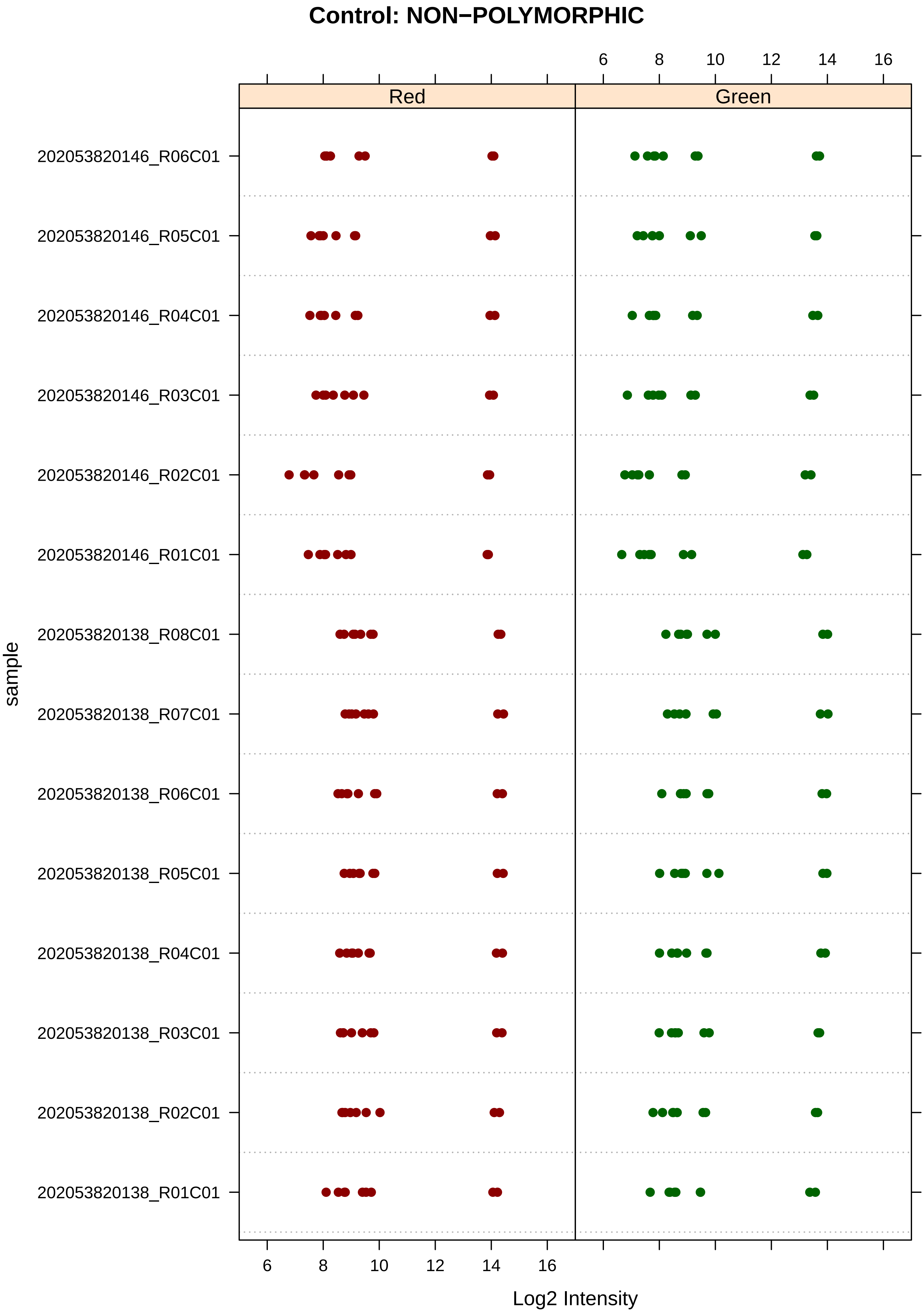
Control: NON−POLYMORPHIC
Red
Green
sample
Log2 Intensity
202053820146_R06C01
202053820146_R05C01
202053820146_R04C01
202053820146_R03C01
202053820146_R02C01
202053820146_R01C01
202053820138_R08C01
202053820138_R07C01
202053820138_R06C01
202053820138_R05C01
202053820138_R04C01
202053820138_R03C01
202053820138_R02C01
202053820138_R01C01
6
8
10
12
14
16

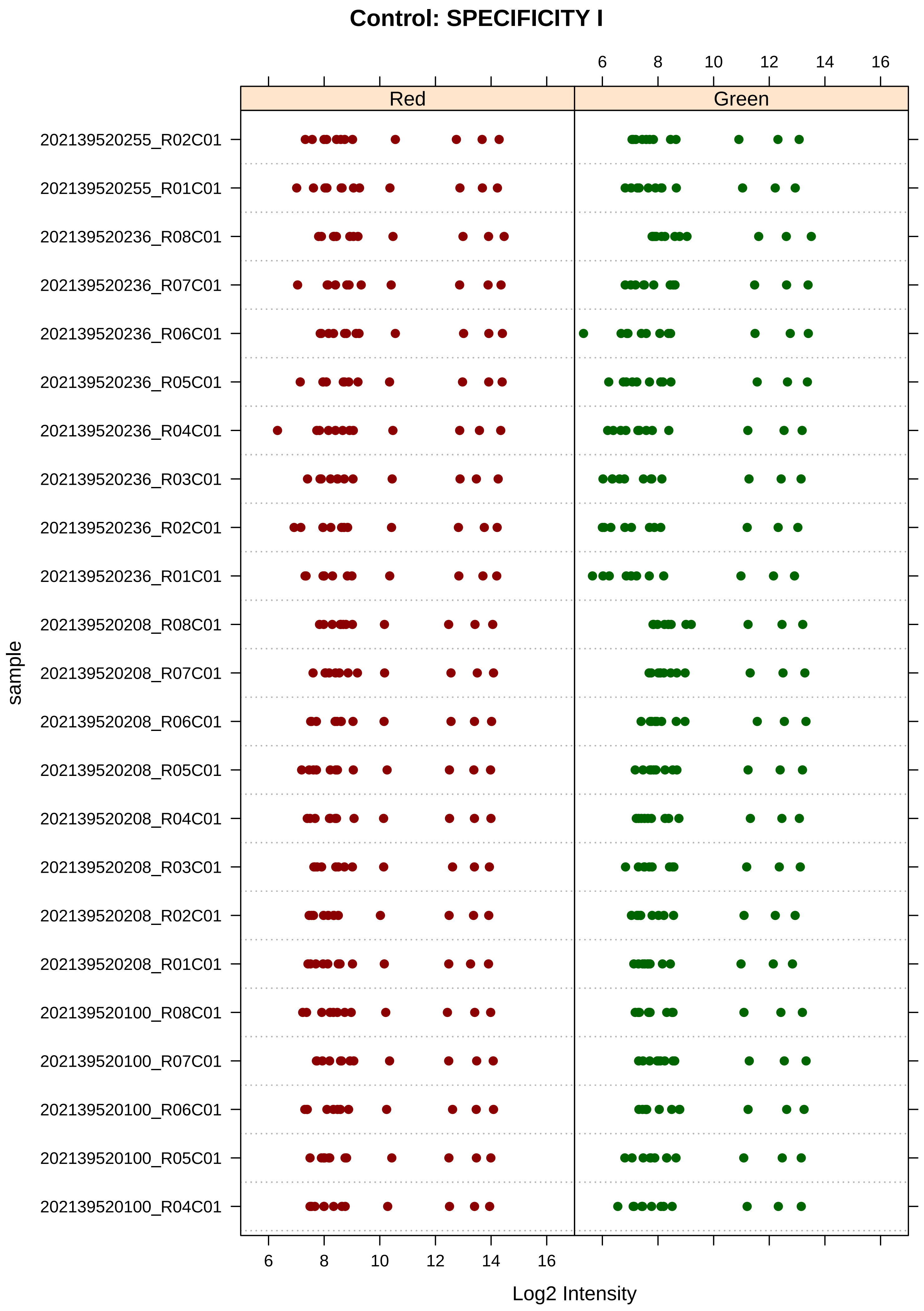

Control: SPECIFICITY I
Red
Green
sample
Log2 Intensity
202139520255_R02C01
202139520255_R01C01
202139520236_R08C01
202139520236_R07C01
202139520236_R06C01
202139520236_R05C01
202139520236_R04C01
202139520236_R03C01
202139520236_R02C01
202139520236_R01C01
202139520208_R08C01
202139520208_R07C01
202139520208_R06C01
202139520208_R05C01
202139520208_R04C01
202139520208_R03C01
202139520208_R02C01
202139520208_R01C01
202139520100_R08C01
202139520100_R07C01
202139520100_R06C01
202139520100_R05C01
202139520100_R04C01
6
8
10
12
14
16

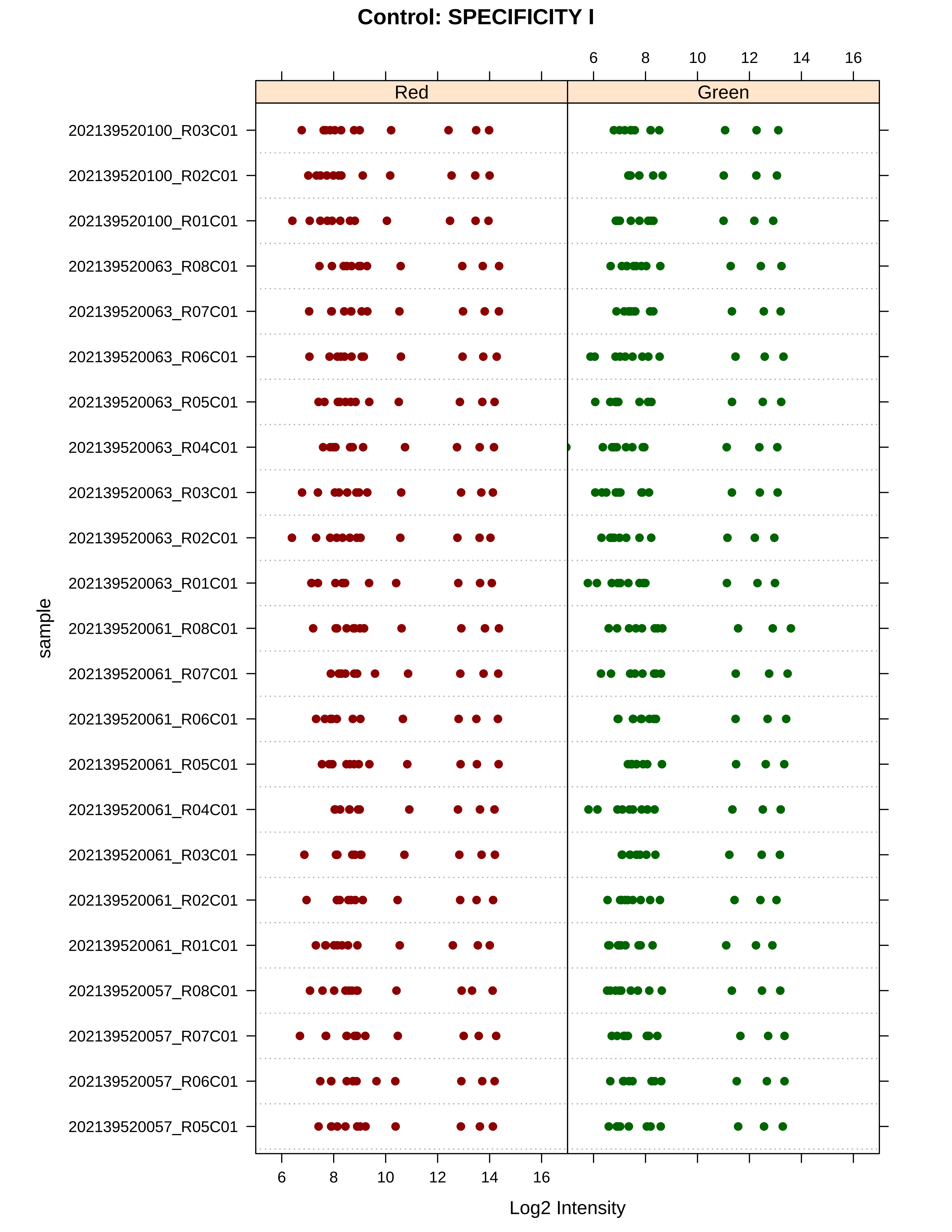

Control: SPECIFICITY I
Red
Green
sample
Log2 Intensity
202139520100_R03C01
202139520100_R02C01
202139520100_R01C01
202139520063_R08C01
202139520063_R07C01
202139520063_R06C01
202139520063_R05C01
202139520063_R04C01
202139520063_R03C01
202139520063_R02C01
202139520063_R01C01
202139520061_R08C01
202139520061_R07C01
202139520061_R06C01
202139520061_R05C01
202139520061_R04C01
202139520061_R03C01
202139520061_R02C01
202139520061_R01C01
202139520057_R08C01
202139520057_R07C01
202139520057_R06C01
202139520057_R05C01
6
8
10
12
14
16

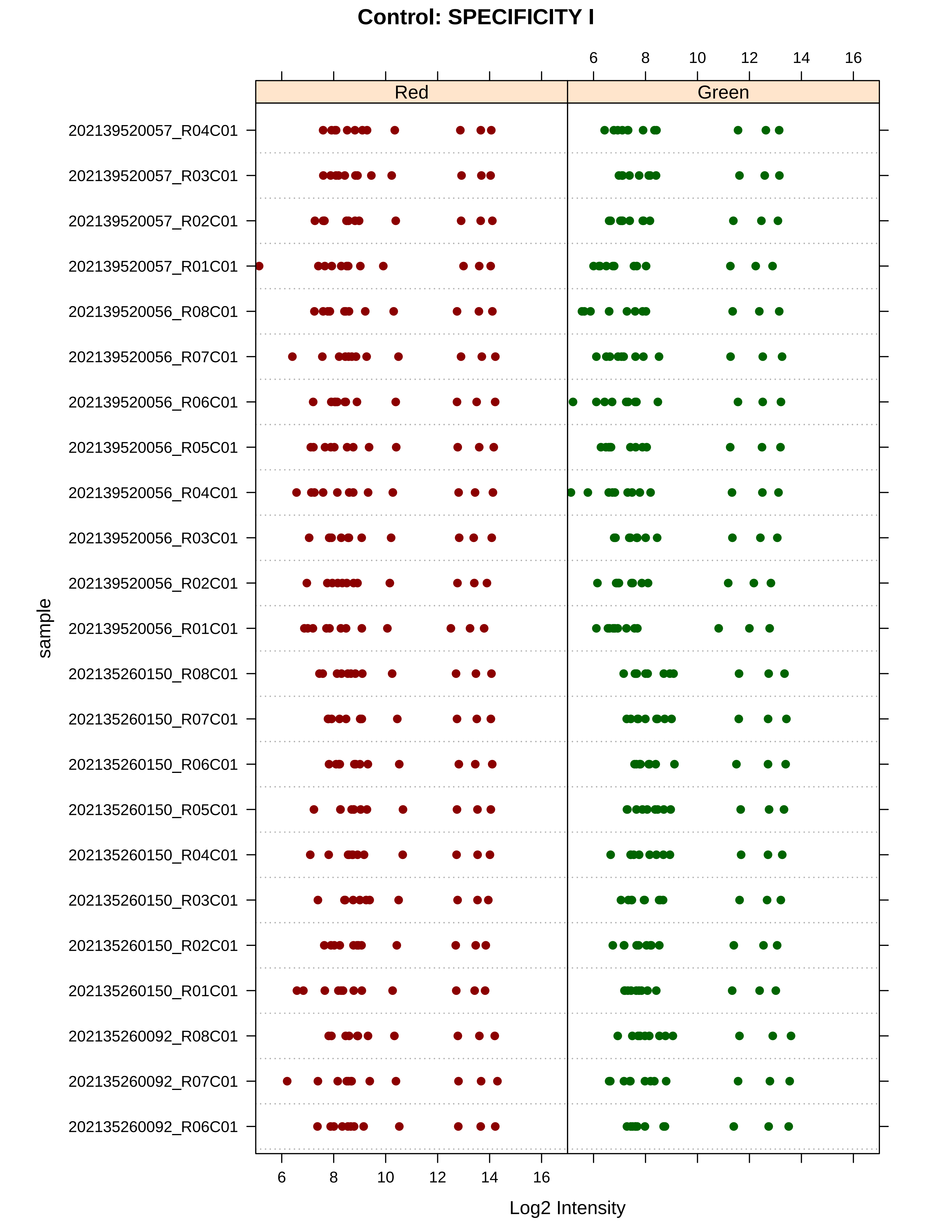

Control: SPECIFICITY I
Red
Green
sample
202139520057_R04C01
202139520057_R03C01
202139520057_R02C01
202139520057_R01C01
202139520056_R08C01
202139520056_R07C01
202139520056_R06C01
202139520056_R05C01
202139520056_R04C01
202139520056_R03C01
202139520056_R02C01
202139520056_R01C01
202135260150_R08C01
202135260150_R07C01
202135260150_R06C01
202135260150_R05C01
202135260150_R04C01
202135260150_R03C01
202135260150_R02C01
202135260150_R01C01
202135260092_R08C01
202135260092_R07C01
202135260092_R06C01
6
8
10
12
14
16
Log2 Intensity

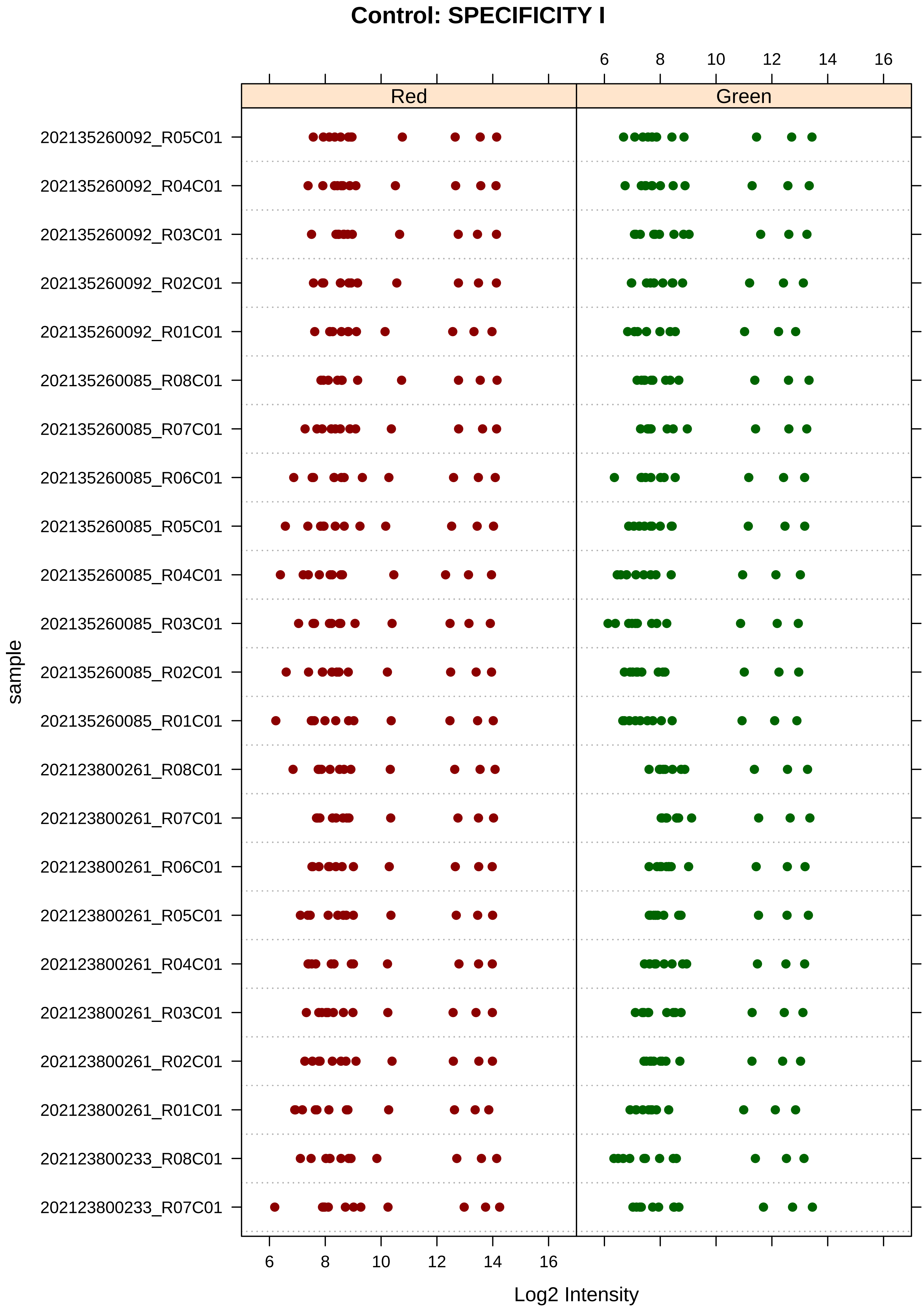

Control: SPECIFICITY I
Red
Green
sample
Log2 Intensity
6
8
10
12
14
16
202135260092_R05C01
202135260092_R04C01
202135260092_R03C01
202135260092_R02C01
202135260092_R01C01
202135260085_R08C01
202135260085_R07C01
202135260085_R06C01
202135260085_R05C01
202135260085_R04C01
202135260085_R03C01
202135260085_R02C01
202135260085_R01C01
202123800261_R08C01
202123800261_R07C01
202123800261_R06C01
202123800261_R05C01
202123800261_R04C01
202123800261_R03C01
202123800261_R02C01
202123800261_R01C01
202123800233_R08C01
202123800233_R07C01

Control: SPECIFICITY I

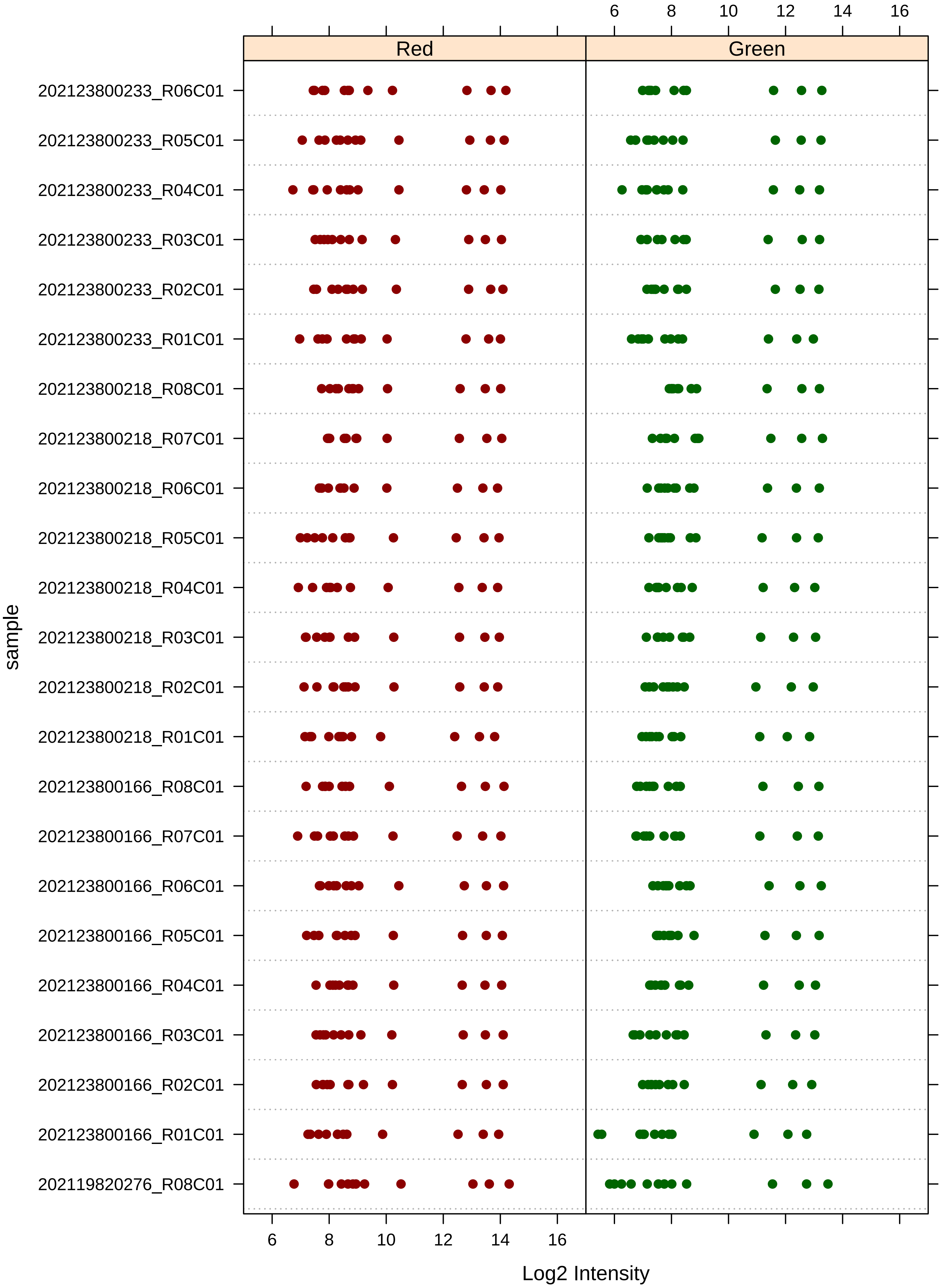

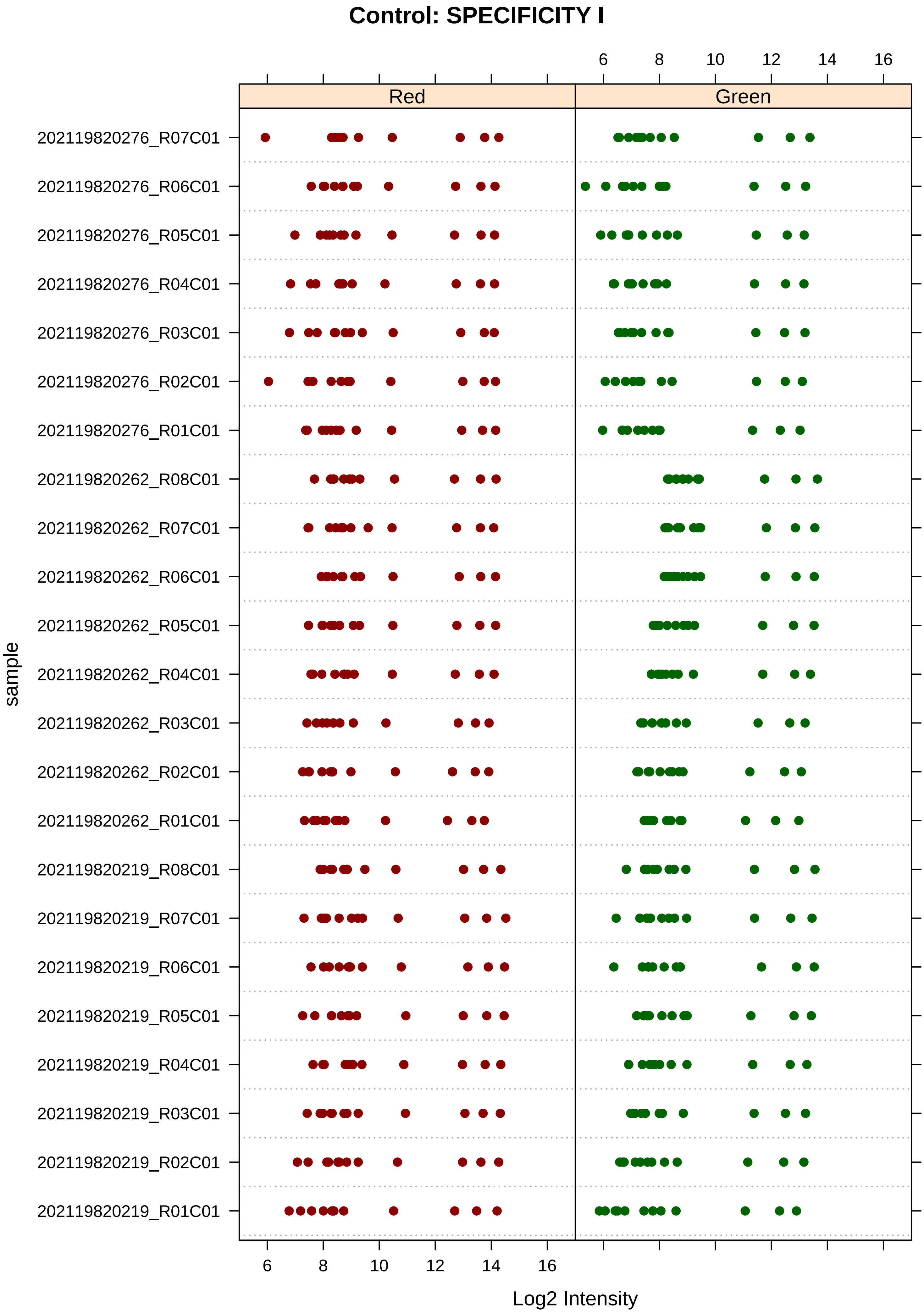

Control: SPECIFICITY I
Red
Green
sample
Log2 Intensity
202119820276_R07C01
202119820276_R06C01
202119820276_R05C01
202119820276_R04C01
202119820276_R03C01
202119820276_R02C01
202119820276_R01C01
202119820262_R08C01
202119820262_R07C01
202119820262_R06C01
202119820262_R05C01
202119820262_R04C01
202119820262_R03C01
202119820262_R02C01
202119820262_R01C01
202119820219_R08C01
202119820219_R07C01
202119820219_R06C01
202119820219_R05C01
202119820219_R04C01
202119820219_R03C01
202119820219_R02C01
202119820219_R01C01
6 8 10 12 14 16

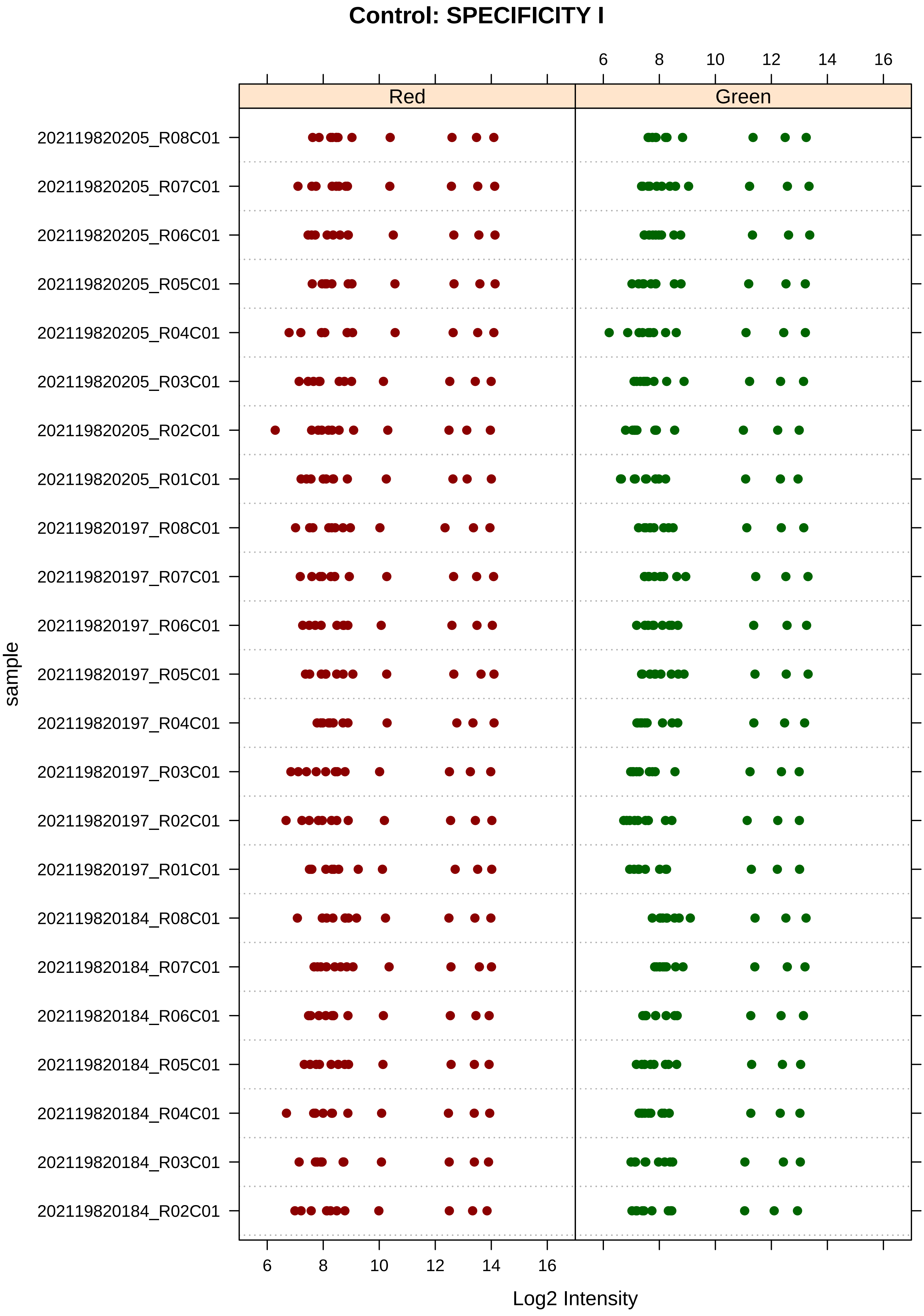
Control: SPECIFICITY I
Red
Green
sample
Log2 Intensity
202119820205_R08C01
202119820205_R07C01
202119820205_R06C01
202119820205_R05C01
202119820205_R04C01
202119820205_R03C01
202119820205_R02C01
202119820205_R01C01
202119820197_R08C01
202119820197_R07C01
202119820197_R06C01
202119820197_R05C01
202119820197_R04C01
202119820197_R03C01
202119820197_R02C01
202119820197_R01C01
202119820184_R08C01
202119820184_R07C01
202119820184_R06C01
202119820184_R05C01
202119820184_R04C01
202119820184_R03C01
202119820184_R02C01
6
8
10
12
14
16

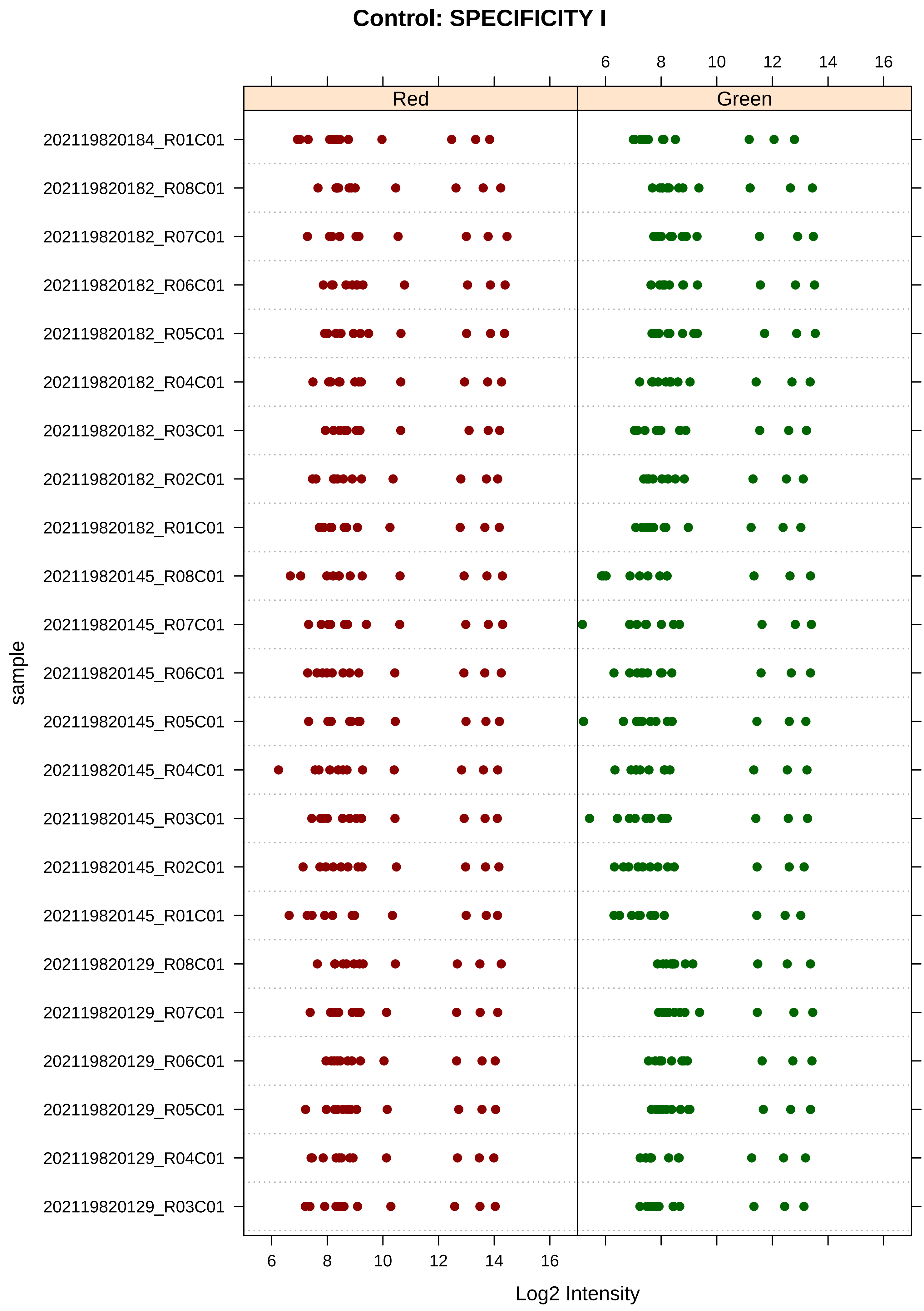

Control: SPECIFICITY I
Red
Green
sample
202119820184_R01C01
202119820182_R08C01
202119820182_R07C01
202119820182_R06C01
202119820182_R05C01
202119820182_R04C01
202119820182_R03C01
202119820182_R02C01
202119820182_R01C01
202119820145_R08C01
202119820145_R07C01
202119820145_R06C01
202119820145_R05C01
202119820145_R04C01
202119820145_R03C01
202119820145_R02C01
202119820145_R01C01
202119820129_R08C01
202119820129_R07C01
202119820129_R06C01
202119820129_R05C01
202119820129_R04C01
202119820129_R03C01
6
8
10
12
14
16
Log2 Intensity

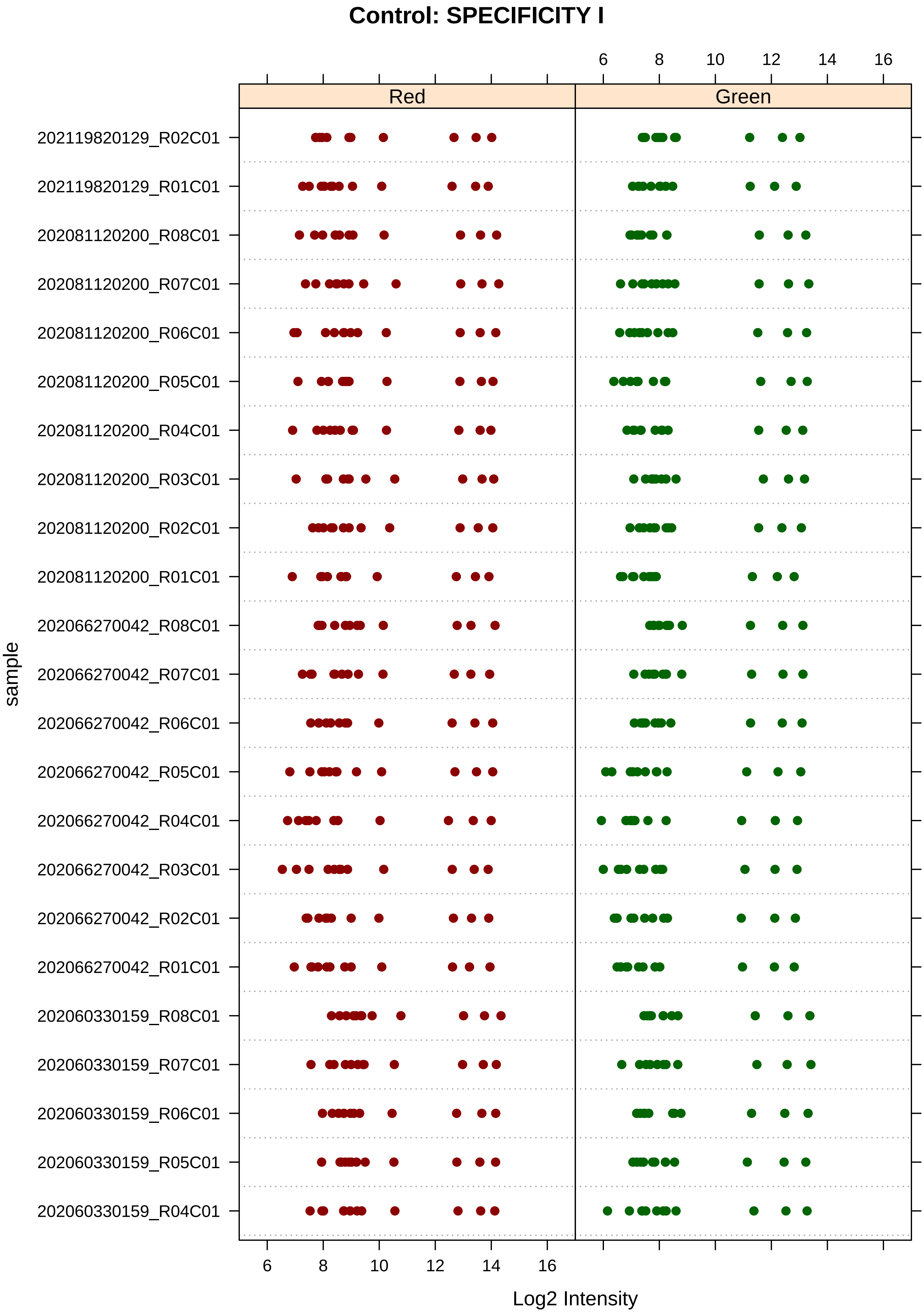
Control: SPECIFICITY I
Red
Green
sample
Log2 Intensity
202119820129_R02C01
202119820129_R01C01
202081120200_R08C01
202081120200_R07C01
202081120200_R06C01
202081120200_R05C01
202081120200_R04C01
202081120200_R03C01
202081120200_R02C01
202081120200_R01C01
202066270042_R08C01
202066270042_R07C01
202066270042_R06C01
202066270042_R05C01
202066270042_R04C01
202066270042_R03C01
202066270042_R02C01
202066270042_R01C01
202060330159_R08C01
202060330159_R07C01
202060330159_R06C01
202060330159_R05C01
202060330159_R04C01
6
8
10
12
14
16

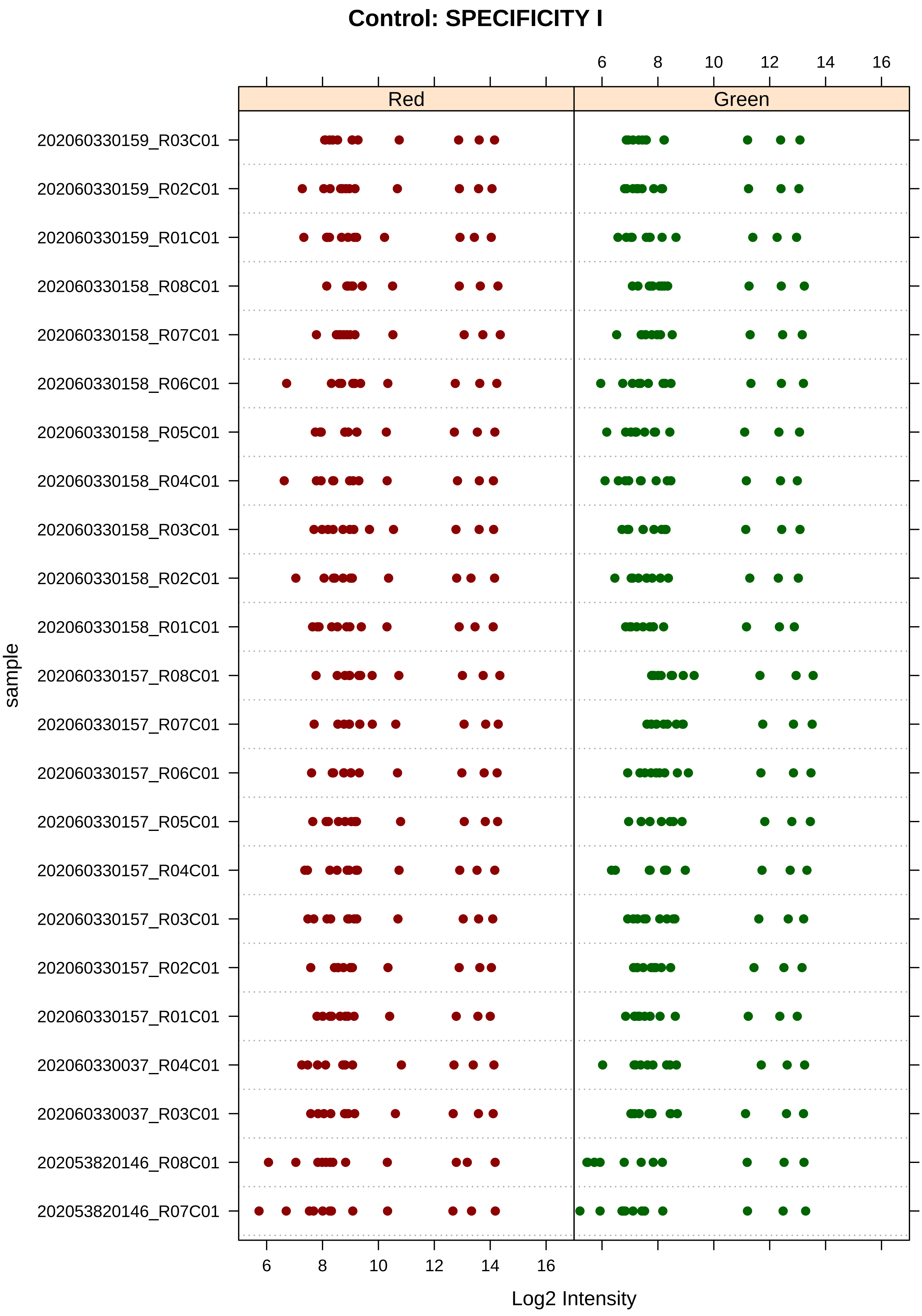

Control: SPECIFICITY I
Red
Green
sample
202060330159_R03C01
202060330159_R02C01
202060330159_R01C01
202060330158_R08C01
202060330158_R07C01
202060330158_R06C01
202060330158_R05C01
202060330158_R04C01
202060330158_R03C01
202060330158_R02C01
202060330158_R01C01
202060330157_R08C01
202060330157_R07C01
202060330157_R06C01
202060330157_R05C01
202060330157_R04C01
202060330157_R03C01
202060330157_R02C01
202060330157_R01C01
202060330037_R04C01
202060330037_R03C01
202053820146_R08C01
202053820146_R07C01
6
8
10
12
14
16
Log2 Intensity

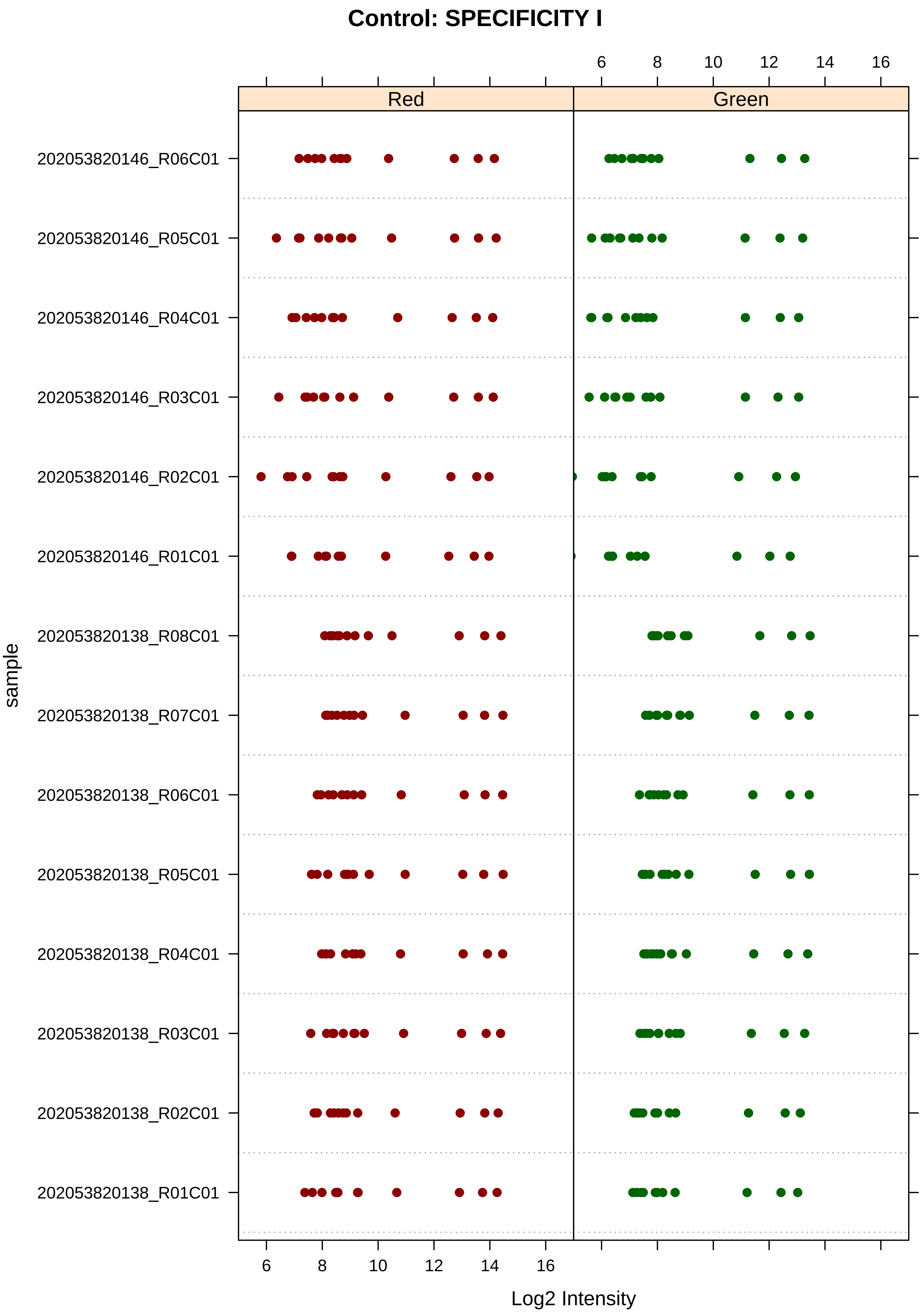

Control: SPECIFICITY I
Red
Green
sample
Log2 Intensity
202053820146_R06C01
202053820146_R05C01
202053820146_R04C01
202053820146_R03C01
202053820146_R02C01
202053820146_R01C01
202053820138_R08C01
202053820138_R07C01
202053820138_R06C01
202053820138_R05C01
202053820138_R04C01
202053820138_R03C01
202053820138_R02C01
202053820138_R01C01
6
8
10
12
14
16

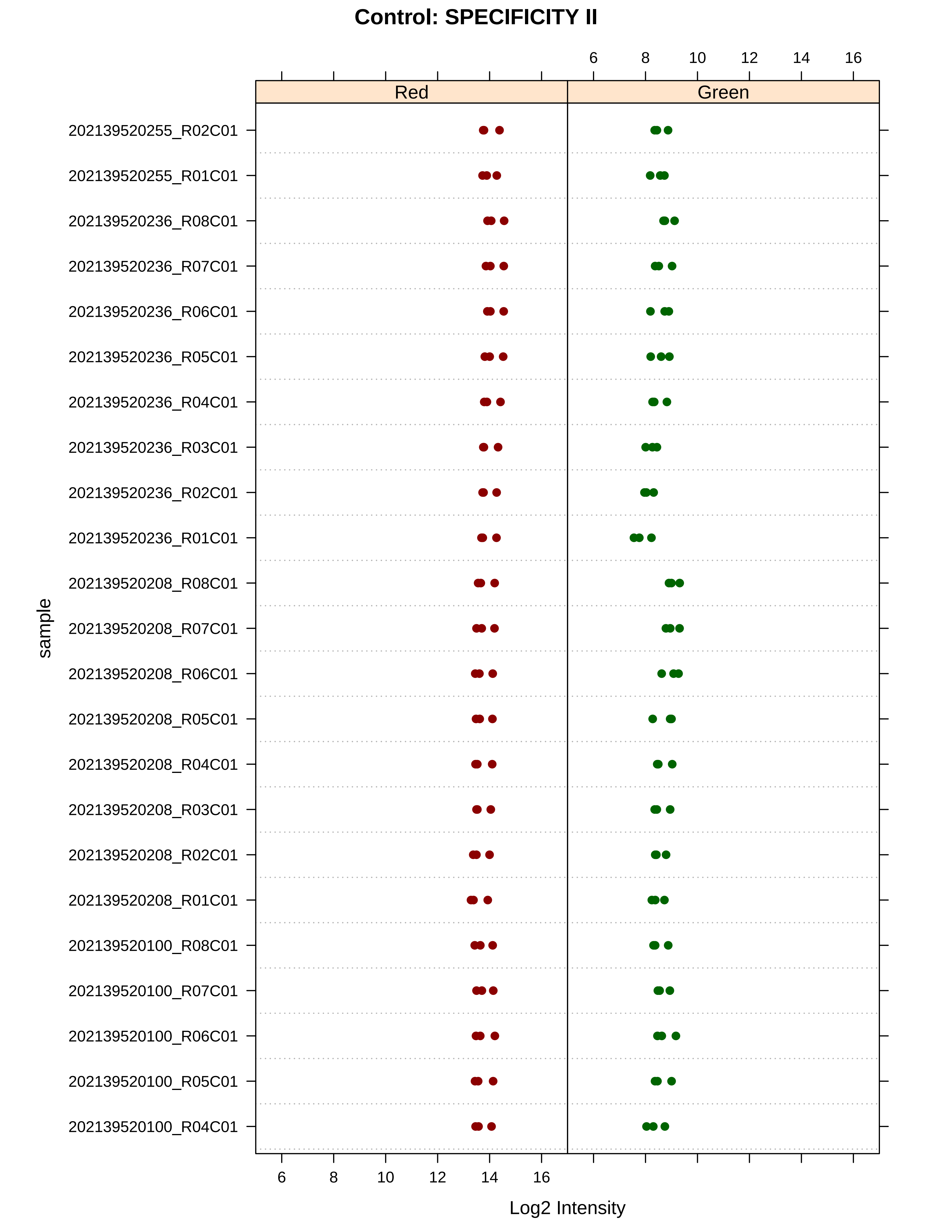

Control: SPECIFICITY II
Red
Green
sample
202139520255_R02C01
202139520255_R01C01
202139520236_R08C01
202139520236_R07C01
202139520236_R06C01
202139520236_R05C01
202139520236_R04C01
202139520236_R03C01
202139520236_R02C01
202139520236_R01C01
202139520208_R08C01
202139520208_R07C01
202139520208_R06C01
202139520208_R05C01
202139520208_R04C01
202139520208_R03C01
202139520208_R02C01
202139520208_R01C01
202139520100_R08C01
202139520100_R07C01
202139520100_R06C01
202139520100_R05C01
202139520100_R04C01
6
8
10
12
14
16
Log2 Intensity

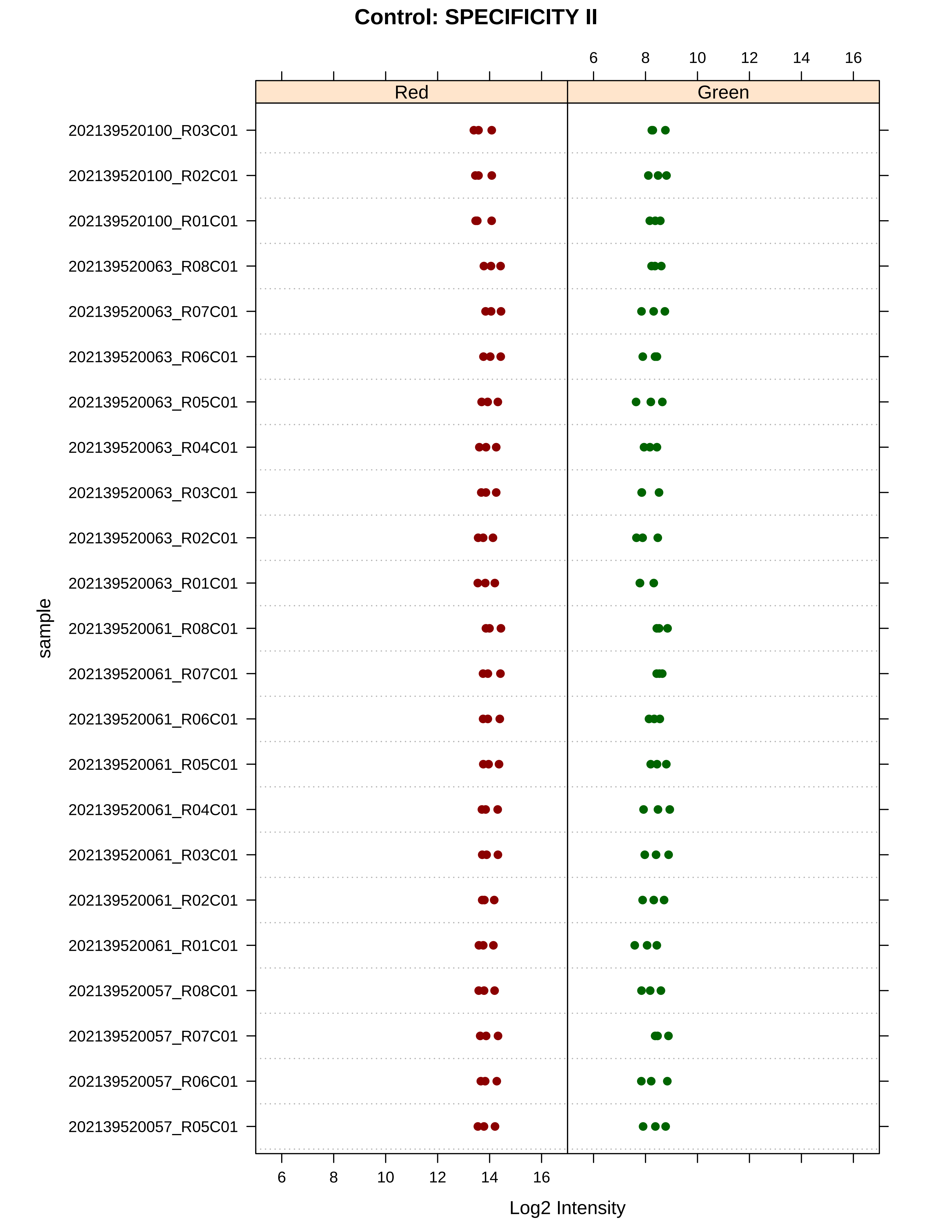
Control: SPECIFICITY II
Red
Green
sample
Log2 Intensity
6
8
10
12
14
16
202139520100_R03C01
202139520100_R02C01
202139520100_R01C01
202139520063_R08C01
202139520063_R07C01
202139520063_R06C01
202139520063_R05C01
202139520063_R04C01
202139520063_R03C01
202139520063_R02C01
202139520063_R01C01
202139520061_R08C01
202139520061_R07C01
202139520061_R06C01
202139520061_R05C01
202139520061_R04C01
202139520061_R03C01
202139520061_R02C01
202139520061_R01C01
202139520057_R08C01
202139520057_R07C01
202139520057_R06C01
202139520057_R05C01

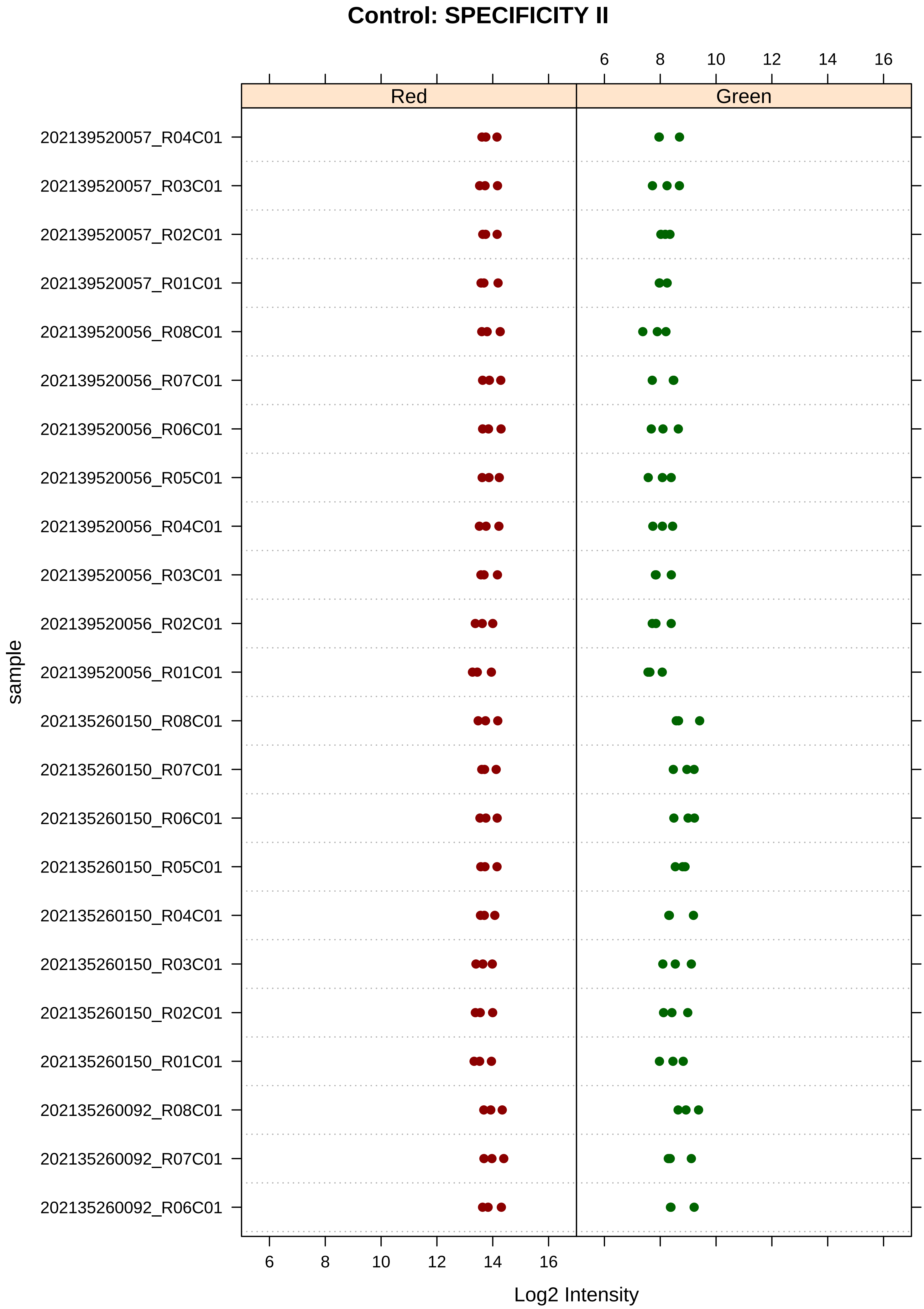

Control: SPECIFICITY II
Red
Green
sample
Log2 Intensity
6
8
10
12
14
16
202139520057_R04C01
202139520057_R03C01
202139520057_R02C01
202139520057_R01C01
202139520056_R08C01
202139520056_R07C01
202139520056_R06C01
202139520056_R05C01
202139520056_R04C01
202139520056_R03C01
202139520056_R02C01
202139520056_R01C01
202135260150_R08C01
202135260150_R07C01
202135260150_R06C01
202135260150_R05C01
202135260150_R04C01
202135260150_R03C01
202135260150_R02C01
202135260150_R01C01
202135260092_R08C01
202135260092_R07C01
202135260092_R06C01

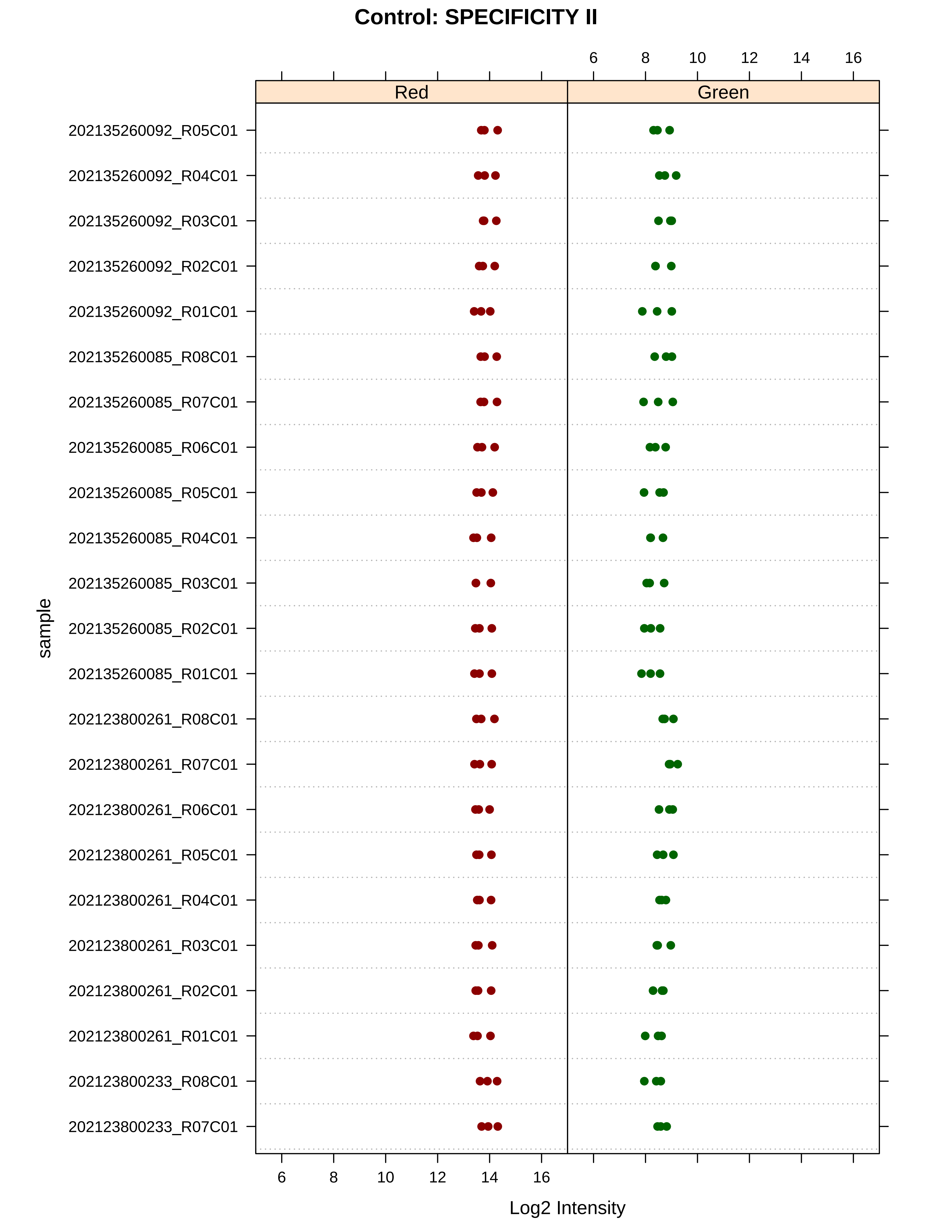

Control: SPECIFICITY II
Red
Green
sample
202135260092_R05C01
202135260092_R04C01
202135260092_R03C01
202135260092_R02C01
202135260092_R01C01
202135260085_R08C01
202135260085_R07C01
202135260085_R06C01
202135260085_R05C01
202135260085_R04C01
202135260085_R03C01
202135260085_R02C01
202135260085_R01C01
202123800261_R08C01
202123800261_R07C01
202123800261_R06C01
202123800261_R05C01
202123800261_R04C01
202123800261_R03C01
202123800261_R02C01
202123800261_R01C01
202123800233_R08C01
202123800233_R07C01
6 8 10 12 14 16
Log2 Intensity

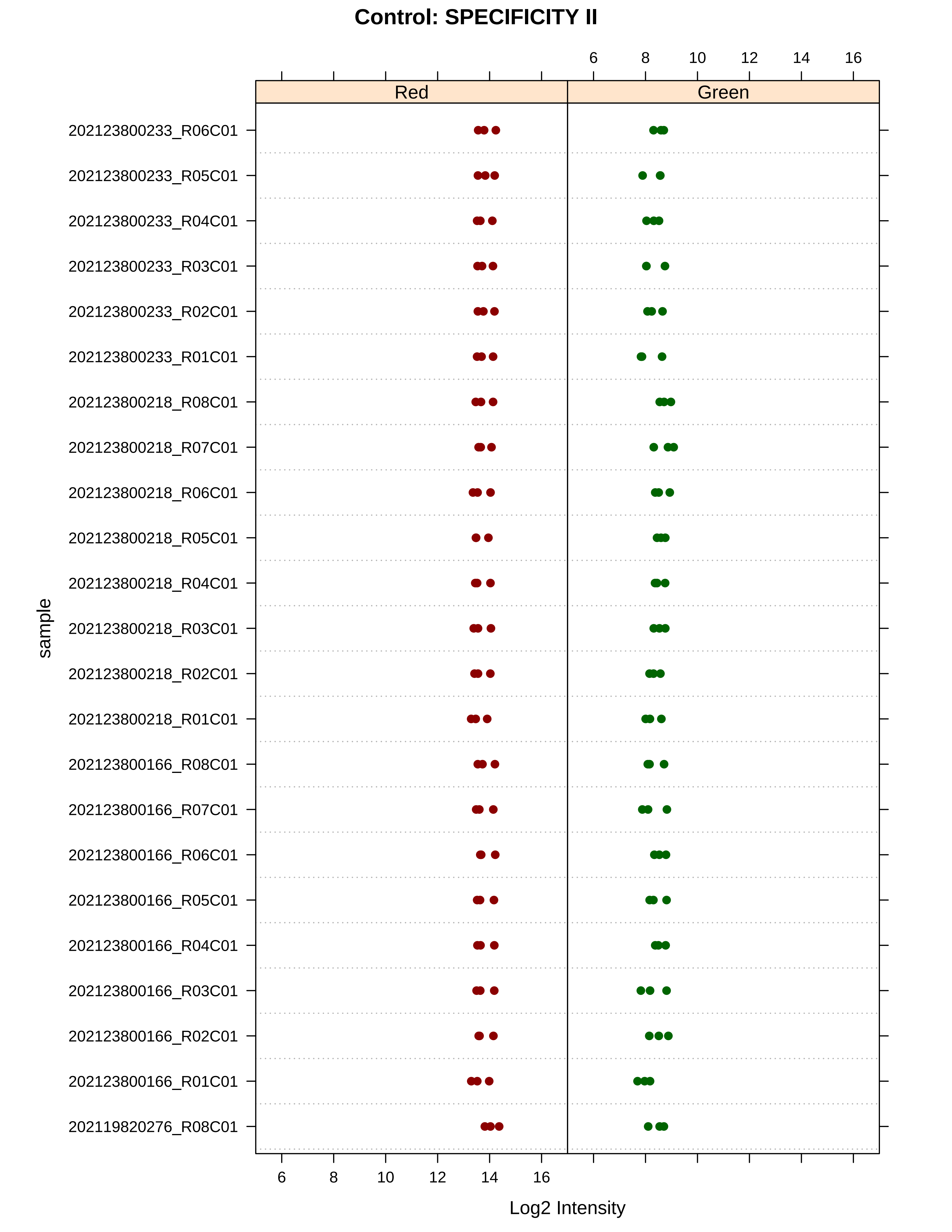

Control: SPECIFICITY II
Red
Green
sample
Log2 Intensity
6
8
10
12
14
16
202123800233_R06C01
202123800233_R05C01
202123800233_R04C01
202123800233_R03C01
202123800233_R02C01
202123800233_R01C01
202123800218_R08C01
202123800218_R07C01
202123800218_R06C01
202123800218_R05C01
202123800218_R04C01
202123800218_R03C01
202123800218_R02C01
202123800218_R01C01
202123800166_R08C01
202123800166_R07C01
202123800166_R06C01
202123800166_R05C01
202123800166_R04C01
202123800166_R03C01
202123800166_R02C01
202123800166_R01C01
202119820276_R08C01

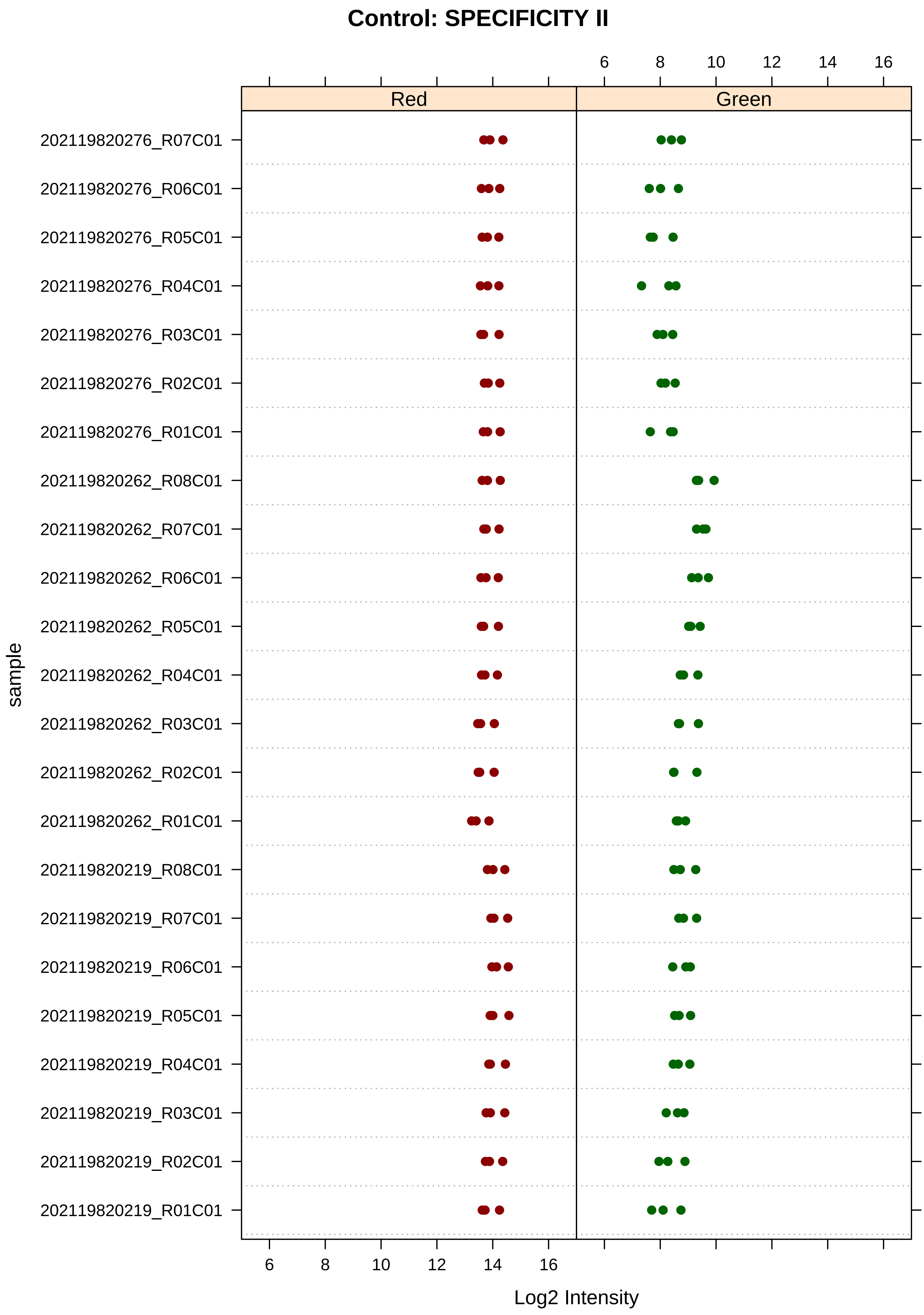
Control: SPECIFICITY II
Red
Green
sample
Log2 Intensity
202119820276_R07C01
202119820276_R06C01
202119820276_R05C01
202119820276_R04C01
202119820276_R03C01
202119820276_R02C01
202119820276_R01C01
202119820262_R08C01
202119820262_R07C01
202119820262_R06C01
202119820262_R05C01
202119820262_R04C01
202119820262_R03C01
202119820262_R02C01
202119820262_R01C01
202119820219_R08C01
202119820219_R07C01
202119820219_R06C01
202119820219_R05C01
202119820219_R04C01
202119820219_R03C01
202119820219_R02C01
202119820219_R01C01
6
8
10
12
14
16

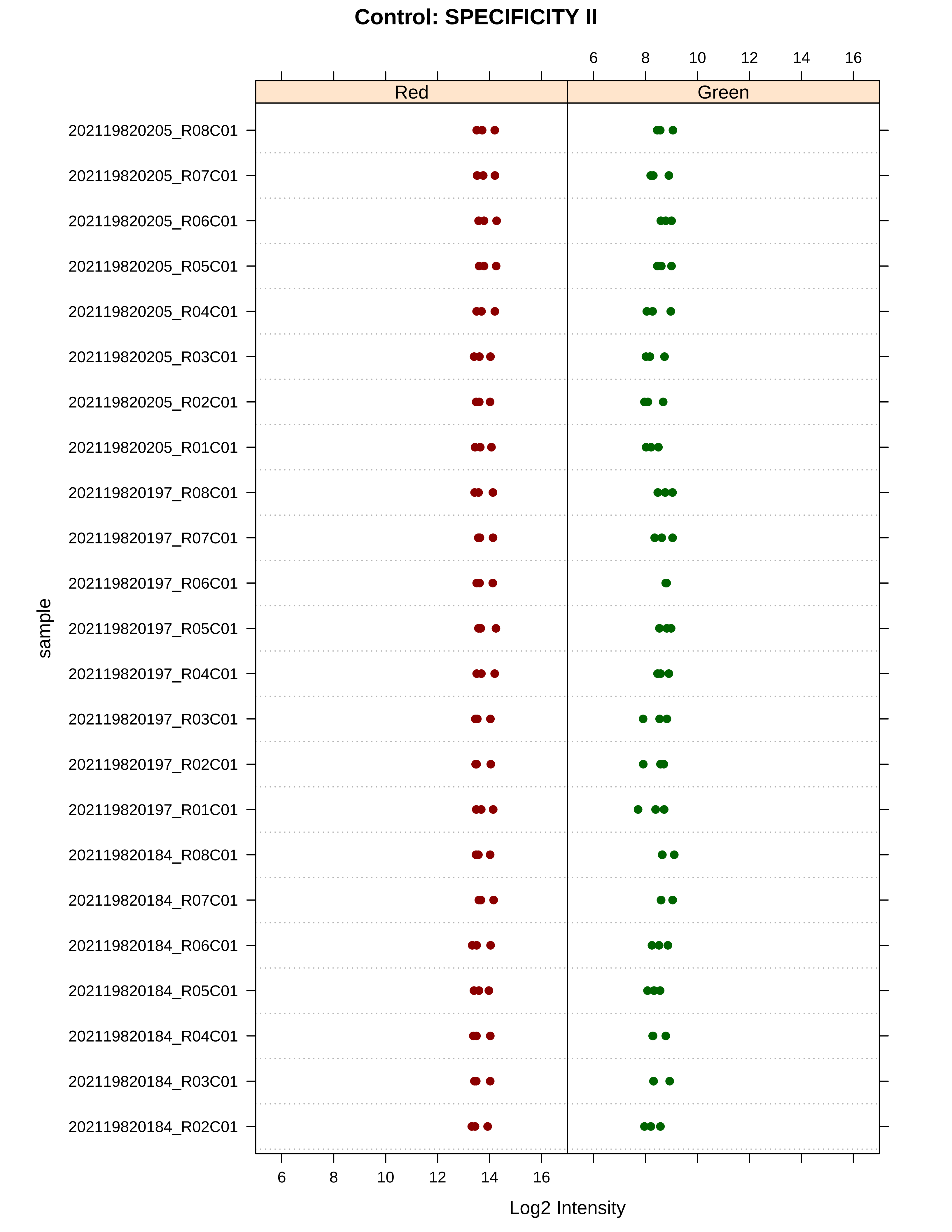

Control: SPECIFICITY II
Red
Green
sample
Log2 Intensity
202119820205_R08C01
202119820205_R07C01
202119820205_R06C01
202119820205_R05C01
202119820205_R04C01
202119820205_R03C01
202119820205_R02C01
202119820205_R01C01
202119820197_R08C01
202119820197_R07C01
202119820197_R06C01
202119820197_R05C01
202119820197_R04C01
202119820197_R03C01
202119820197_R02C01
202119820197_R01C01
202119820184_R08C01
202119820184_R07C01
202119820184_R06C01
202119820184_R05C01
202119820184_R04C01
202119820184_R03C01
202119820184_R02C01
6 8 10 12 14 16

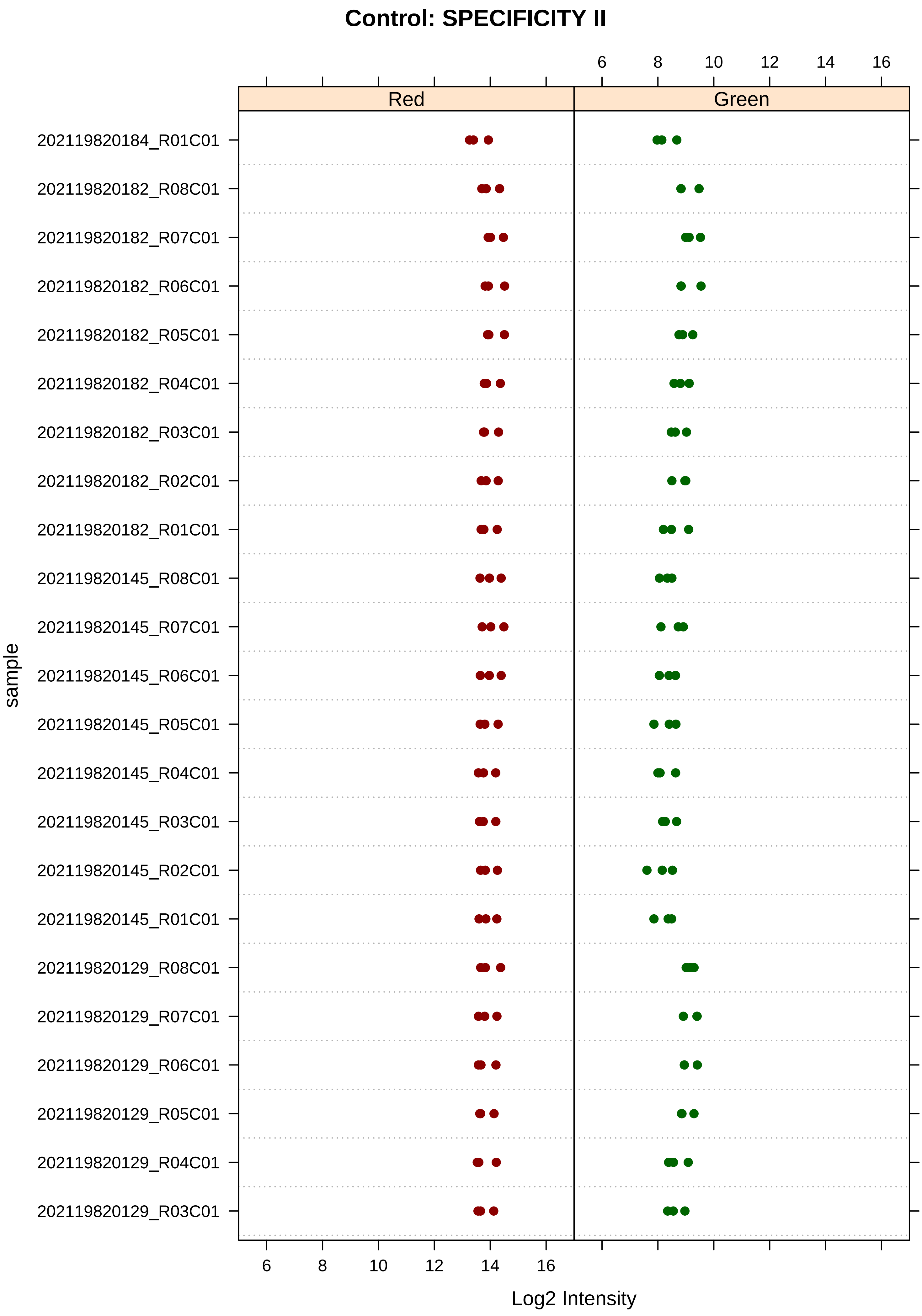

Control: SPECIFICITY II
Red
Green
sample
Log2 Intensity
202119820184_R01C01
202119820182_R08C01
202119820182_R07C01
202119820182_R06C01
202119820182_R05C01
202119820182_R04C01
202119820182_R03C01
202119820182_R02C01
202119820182_R01C01
202119820145_R08C01
202119820145_R07C01
202119820145_R06C01
202119820145_R05C01
202119820145_R04C01
202119820145_R03C01
202119820145_R02C01
202119820145_R01C01
202119820129_R08C01
202119820129_R07C01
202119820129_R06C01
202119820129_R05C01
202119820129_R04C01
202119820129_R03C01
6
8
10
12
14
16

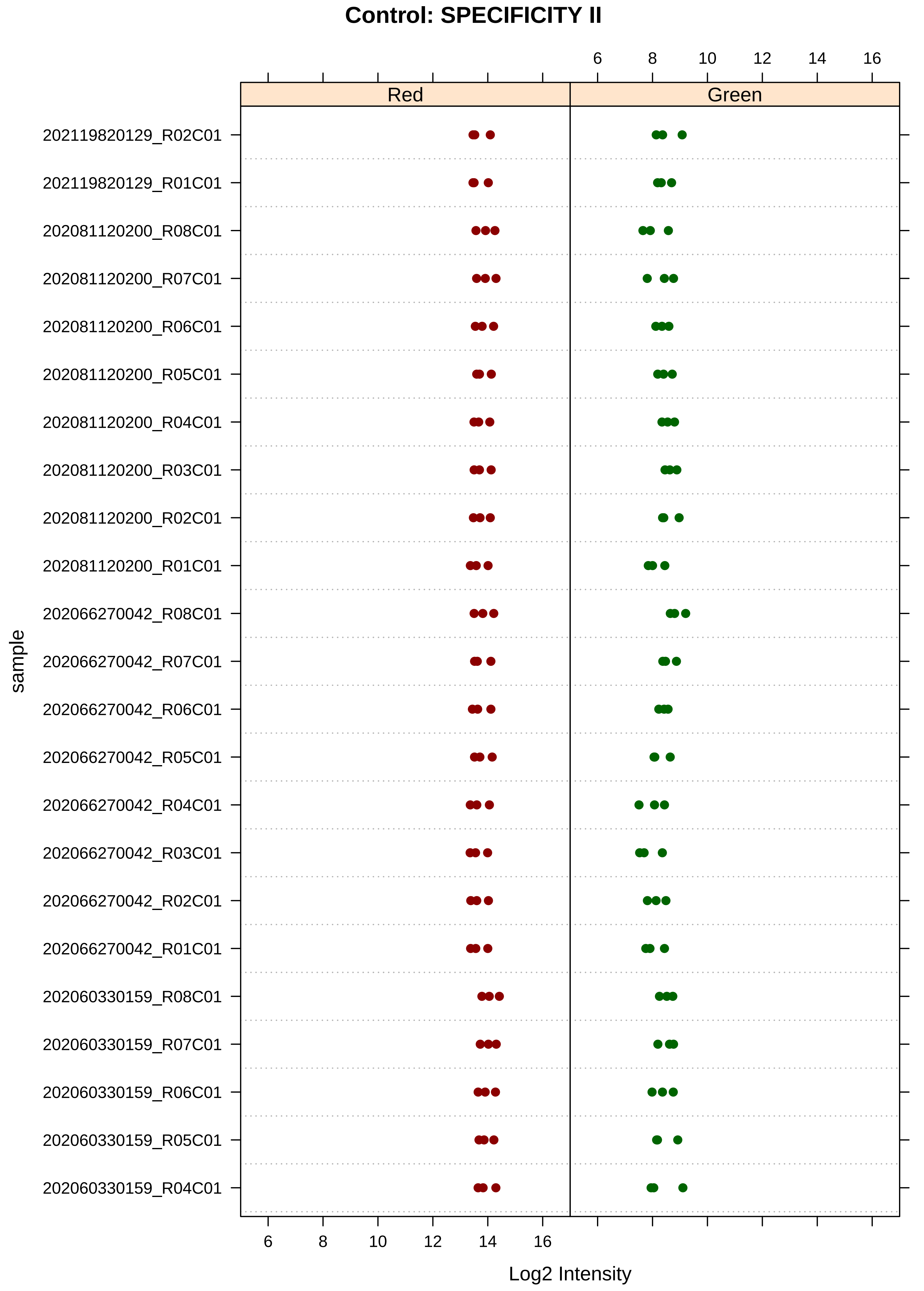

Control: SPECIFICITY II
Red
Green
sample
Log2 Intensity
6
8
10
12
14
16
202119820129_R02C01
202119820129_R01C01
202081120200_R08C01
202081120200_R07C01
202081120200_R06C01
202081120200_R05C01
202081120200_R04C01
202081120200_R03C01
202081120200_R02C01
202081120200_R01C01
202066270042_R08C01
202066270042_R07C01
202066270042_R06C01
202066270042_R05C01
202066270042_R04C01
202066270042_R03C01
202066270042_R02C01
202066270042_R01C01
202060330159_R08C01
202060330159_R07C01
202060330159_R06C01
202060330159_R05C01
202060330159_R04C01

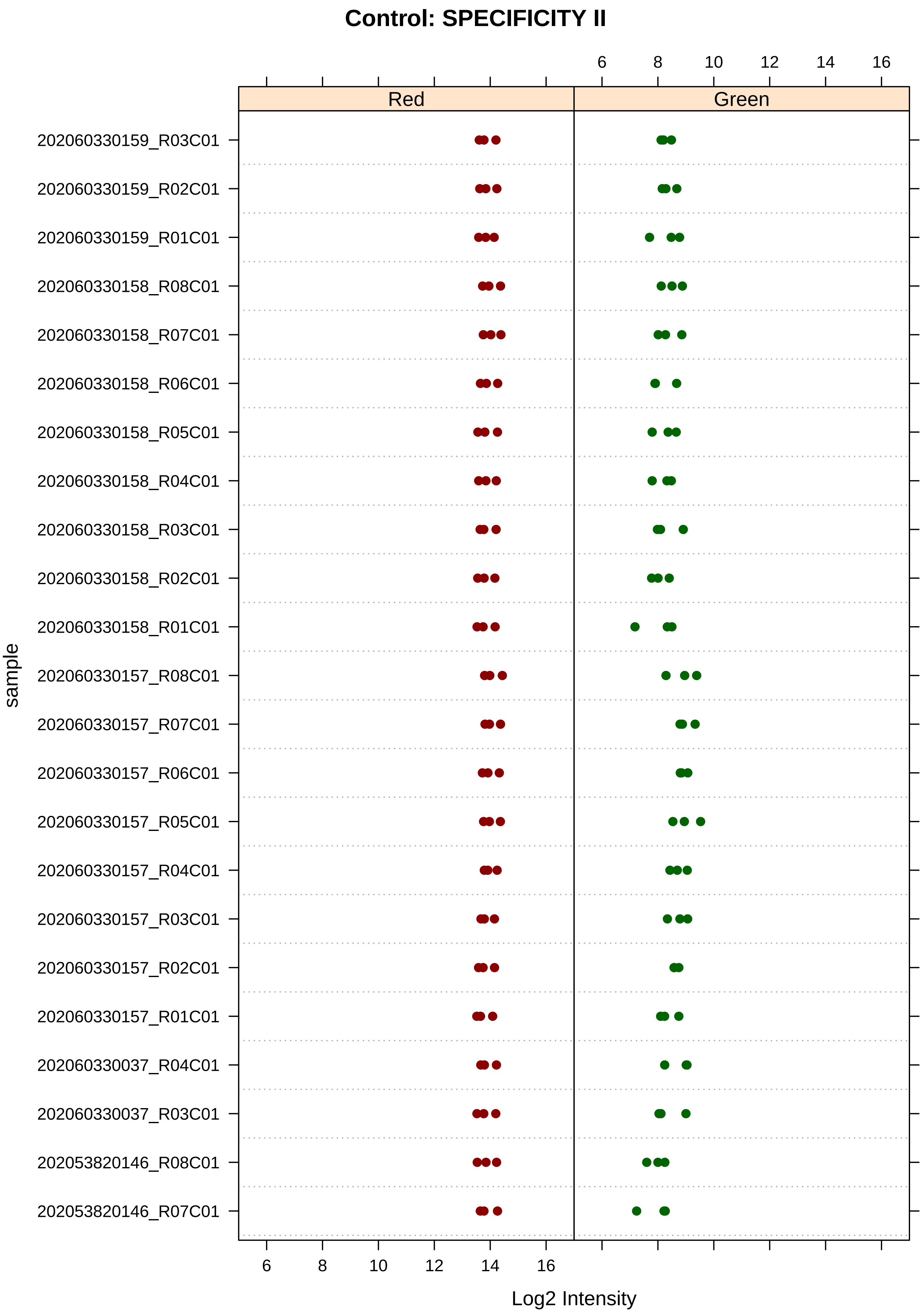
Control: SPECIFICITY II
Red
Green
202060330159_R03C01
202060330159_R02C01
202060330159_R01C01
202060330158_R08C01
202060330158_R07C01
202060330158_R06C01
202060330158_R05C01
202060330158_R04C01
202060330158_R03C01
202060330158_R02C01
202060330158_R01C01
202060330157_R08C01
202060330157_R07C01
202060330157_R06C01
202060330157_R05C01
202060330157_R04C01
202060330157_R03C01
202060330157_R02C01
202060330157_R01C01
202060330037_R04C01
202060330037_R03C01
202053820146_R08C01
202053820146_R07C01
sample
6
8
10
12
14
16
Log2 Intensity

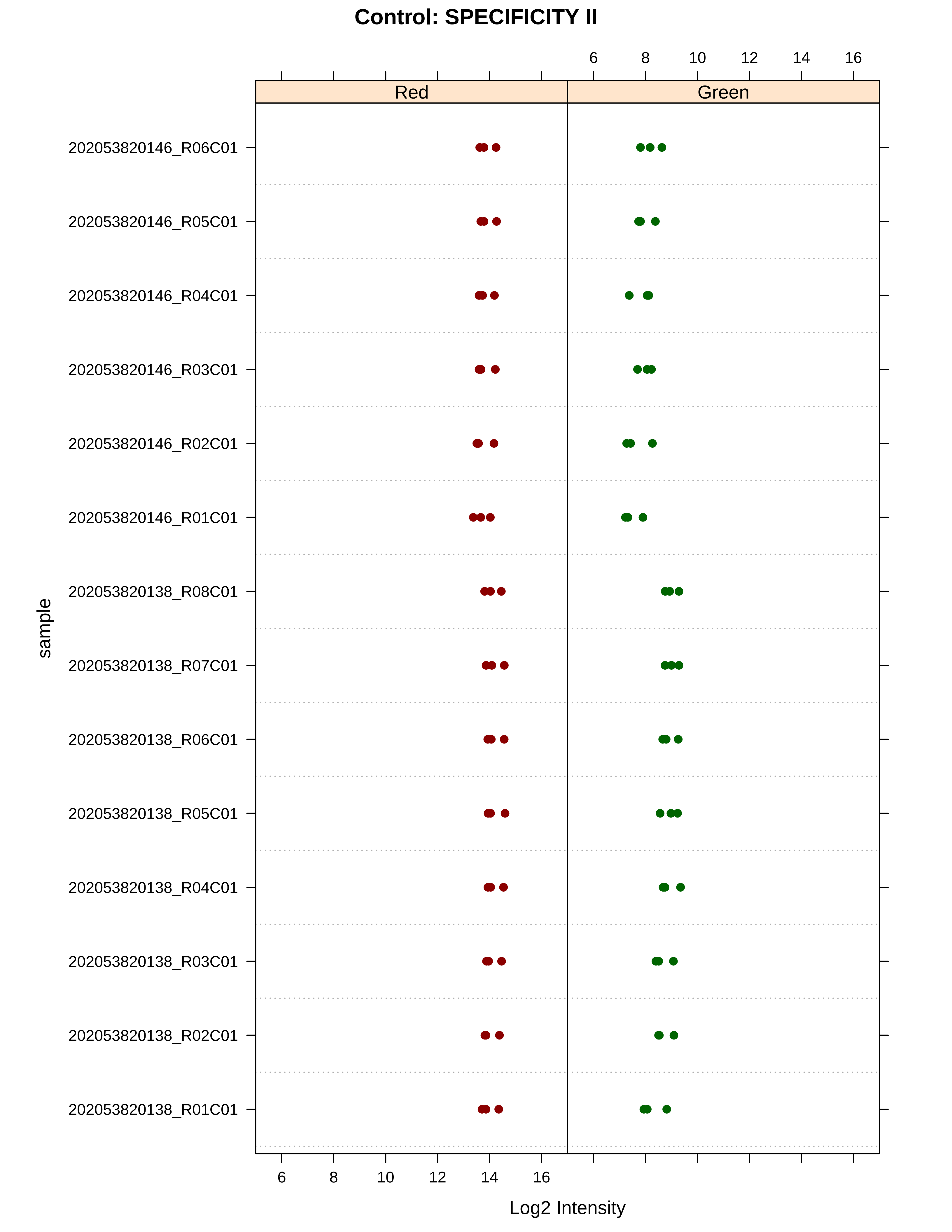

Control: SPECIFICITY II
Red
Green
sample
Log2 Intensity
202053820146_R06C01
202053820146_R05C01
202053820146_R04C01
202053820146_R03C01
202053820146_R02C01
202053820146_R01C01
202053820138_R08C01
202053820138_R07C01
202053820138_R06C01
202053820138_R05C01
202053820138_R04C01
202053820138_R03C01
202053820138_R02C01
202053820138_R01C01
6
8
10
12
14
16

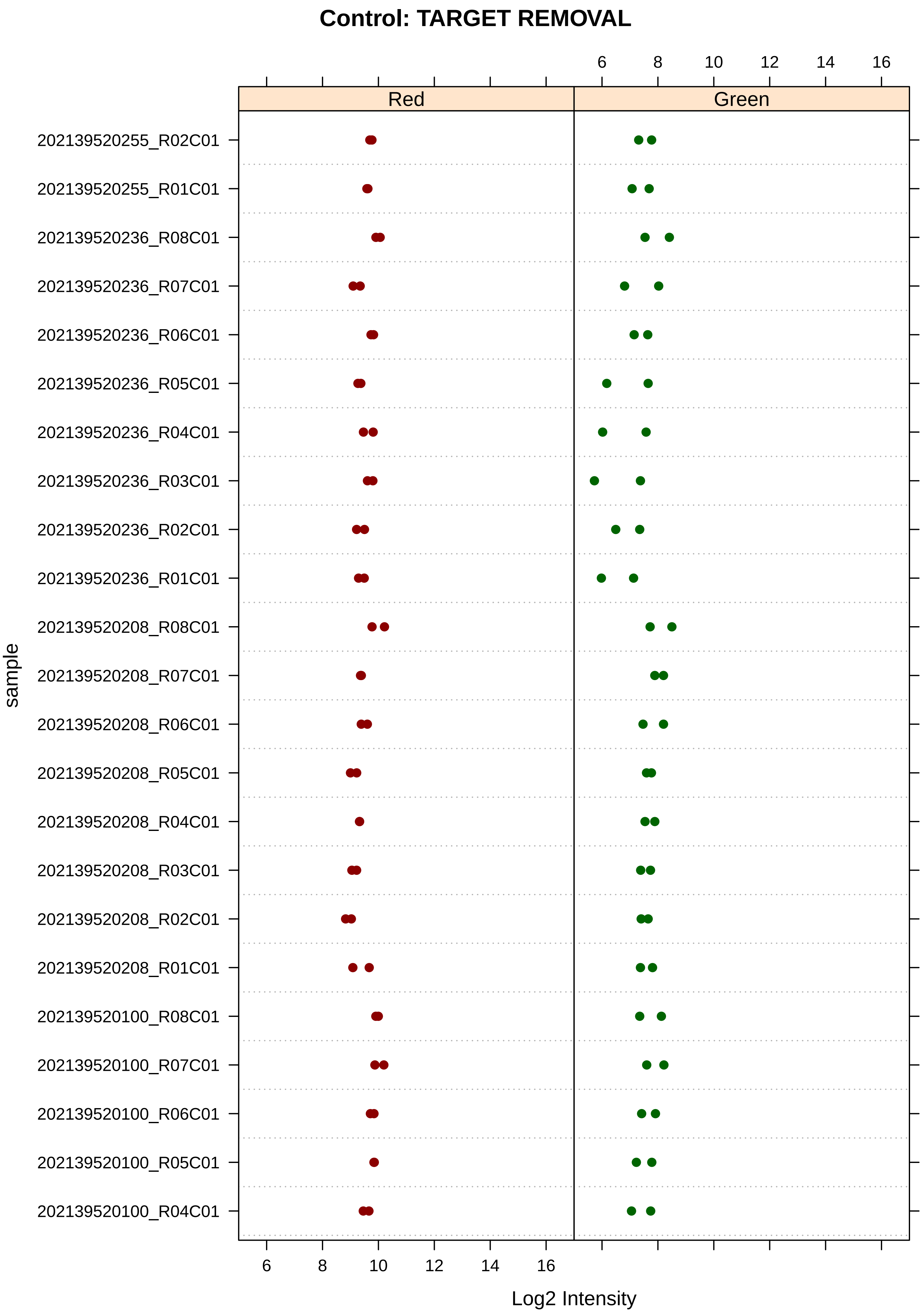
Control: TARGET REMOVAL
Red
Green
sample
Log2 Intensity
202139520255_R02C01
202139520255_R01C01
202139520236_R08C01
202139520236_R07C01
202139520236_R06C01
202139520236_R05C01
202139520236_R04C01
202139520236_R03C01
202139520236_R02C01
202139520236_R01C01
202139520208_R08C01
202139520208_R07C01
202139520208_R06C01
202139520208_R05C01
202139520208_R04C01
202139520208_R03C01
202139520208_R02C01
202139520208_R01C01
202139520100_R08C01
202139520100_R07C01
202139520100_R06C01
202139520100_R05C01
202139520100_R04C01
6
8
10
12
14
16

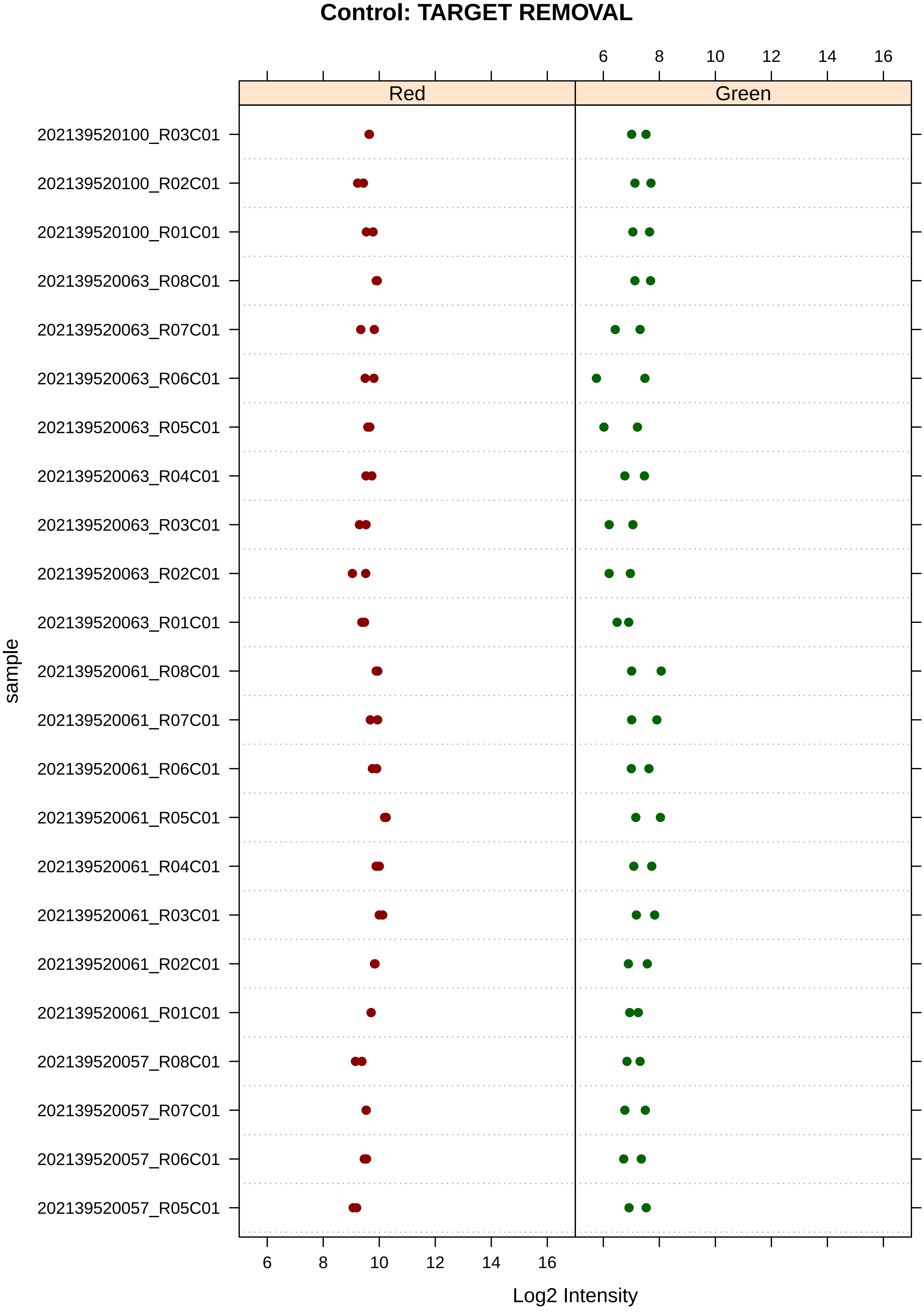
Control: TARGET REMOVAL
Red
Green
sample
Log2 Intensity
202139520100_R03C01
202139520100_R02C01
202139520100_R01C01
202139520063_R08C01
202139520063_R07C01
202139520063_R06C01
202139520063_R05C01
202139520063_R04C01
202139520063_R03C01
202139520063_R02C01
202139520063_R01C01
202139520061_R08C01
202139520061_R07C01
202139520061_R06C01
202139520061_R05C01
202139520061_R04C01
202139520061_R03C01
202139520061_R02C01
202139520061_R01C01
202139520057_R08C01
202139520057_R07C01
202139520057_R06C01
202139520057_R05C01
6
8
10
12
14
16

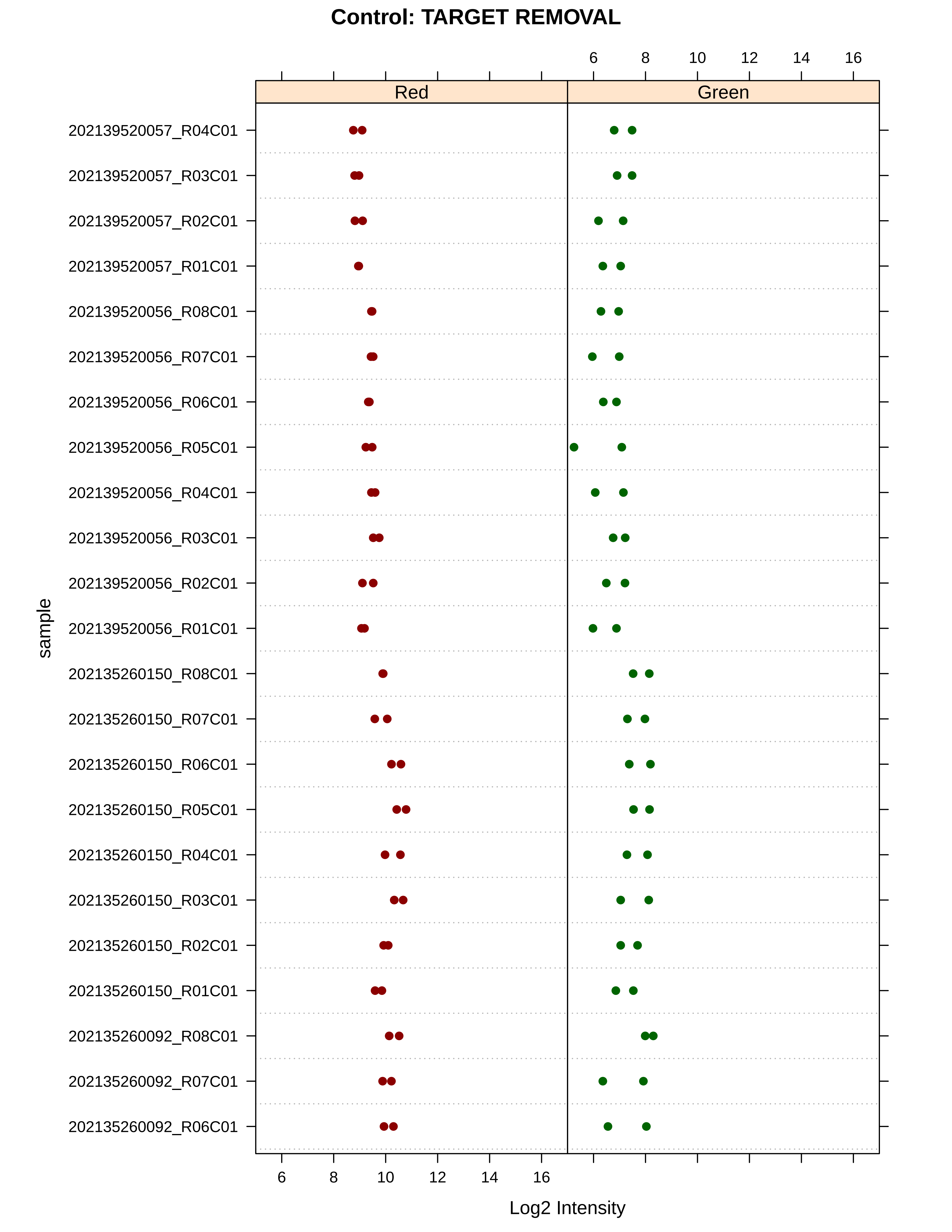
Control: TARGET REMOVAL
Red
Green
sample
Log2 Intensity
202139520057_R04C01
202139520057_R03C01
202139520057_R02C01
202139520057_R01C01
202139520056_R08C01
202139520056_R07C01
202139520056_R06C01
202139520056_R05C01
202139520056_R04C01
202139520056_R03C01
202139520056_R02C01
202139520056_R01C01
202135260150_R08C01
202135260150_R07C01
202135260150_R06C01
202135260150_R05C01
202135260150_R04C01
202135260150_R03C01
202135260150_R02C01
202135260150_R01C01
202135260092_R08C01
202135260092_R07C01
202135260092_R06C01
6
8
10
12
14
16

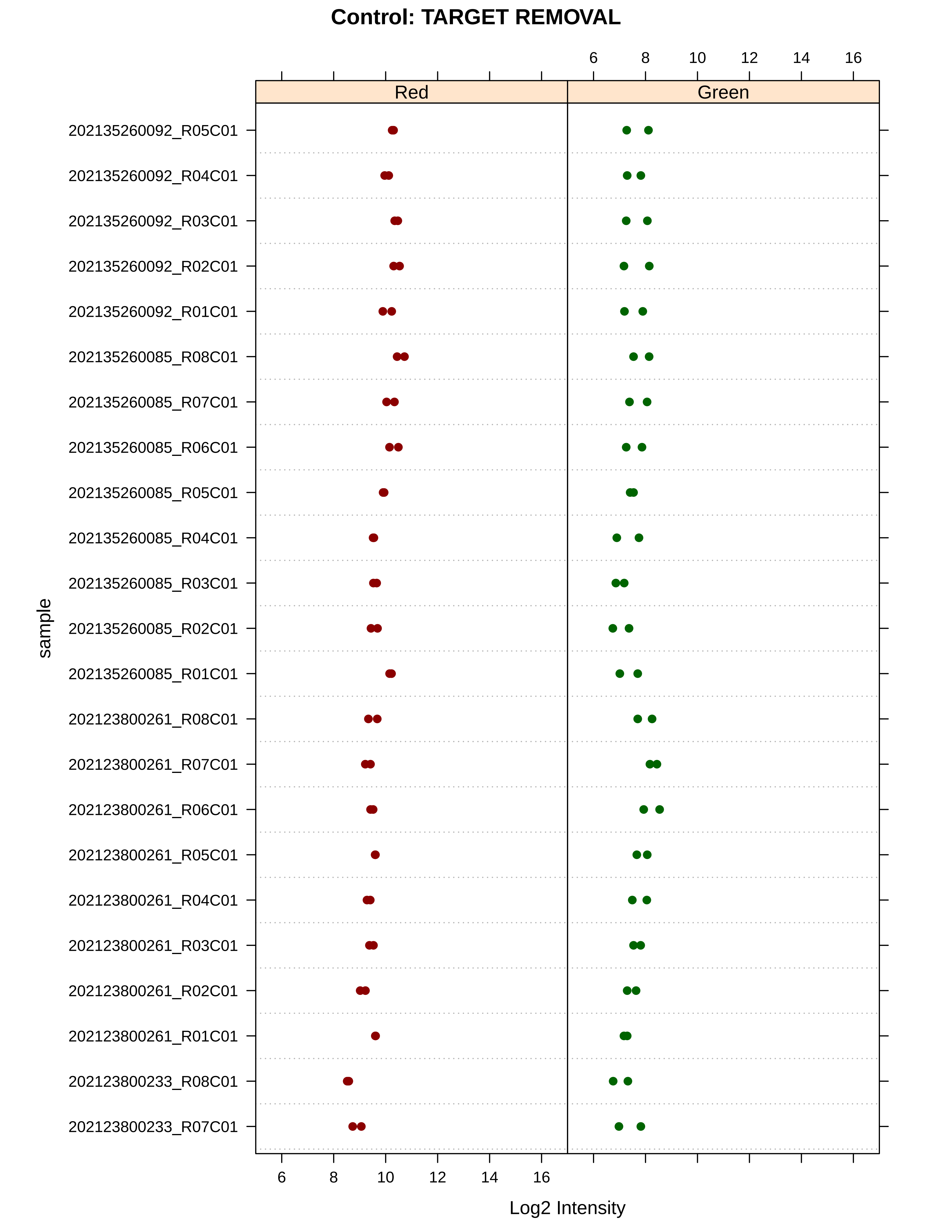

Control: TARGET REMOVAL
Red
Green
sample
Log2 Intensity
6
8
10
12
14
16
202135260092_R05C01
202135260092_R04C01
202135260092_R03C01
202135260092_R02C01
202135260092_R01C01
202135260085_R08C01
202135260085_R07C01
202135260085_R06C01
202135260085_R05C01
202135260085_R04C01
202135260085_R03C01
202135260085_R02C01
202135260085_R01C01
202123800261_R08C01
202123800261_R07C01
202123800261_R06C01
202123800261_R05C01
202123800261_R04C01
202123800261_R03C01
202123800261_R02C01
202123800261_R01C01
202123800233_R08C01
202123800233_R07C01

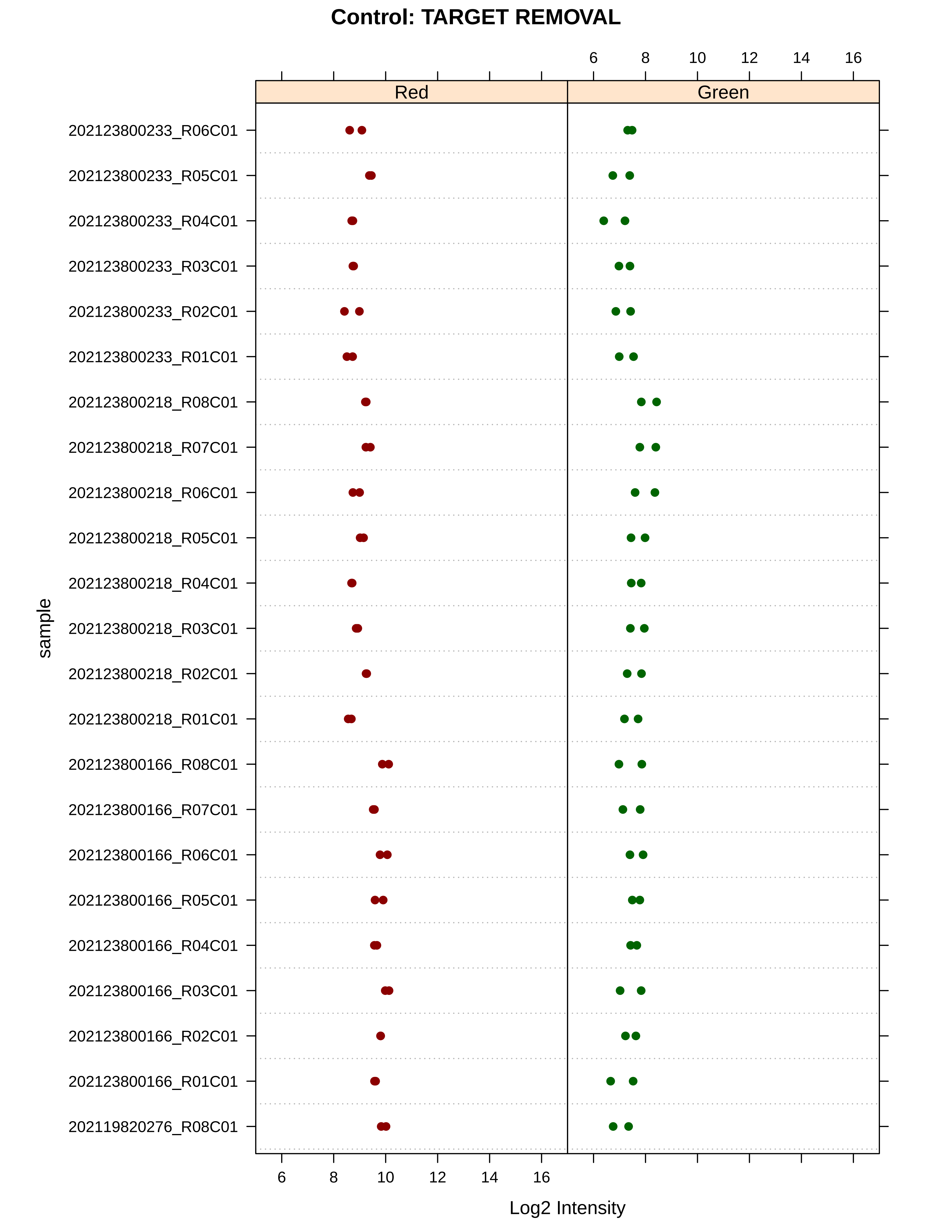

Control: TARGET REMOVAL
Red
Green
sample
202123800233_R06C01
202123800233_R05C01
202123800233_R04C01
202123800233_R03C01
202123800233_R02C01
202123800233_R01C01
202123800218_R08C01
202123800218_R07C01
202123800218_R06C01
202123800218_R05C01
202123800218_R04C01
202123800218_R03C01
202123800218_R02C01
202123800218_R01C01
202123800166_R08C01
202123800166_R07C01
202123800166_R06C01
202123800166_R05C01
202123800166_R04C01
202123800166_R03C01
202123800166_R02C01
202123800166_R01C01
202119820276_R08C01
6 8 10 12 14 16
Log2 Intensity

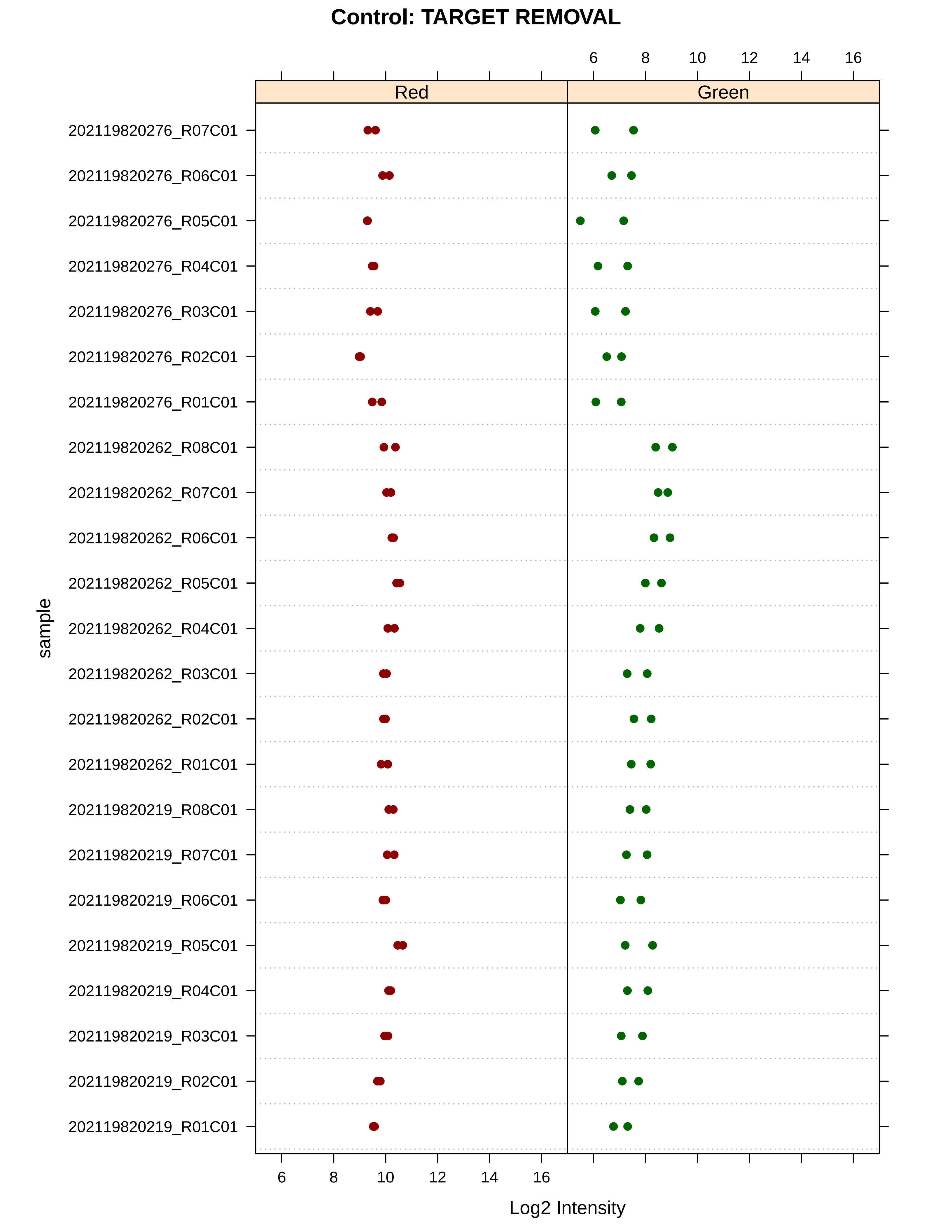
Control: TARGET REMOVAL
Red
Green
sample
Log2 Intensity
202119820276_R07C01
202119820276_R06C01
202119820276_R05C01
202119820276_R04C01
202119820276_R03C01
202119820276_R02C01
202119820276_R01C01
202119820262_R08C01
202119820262_R07C01
202119820262_R06C01
202119820262_R05C01
202119820262_R04C01
202119820262_R03C01
202119820262_R02C01
202119820262_R01C01
202119820219_R08C01
202119820219_R07C01
202119820219_R06C01
202119820219_R05C01
202119820219_R04C01
202119820219_R03C01
202119820219_R02C01
202119820219_R01C01
6
8
10
12
14
16

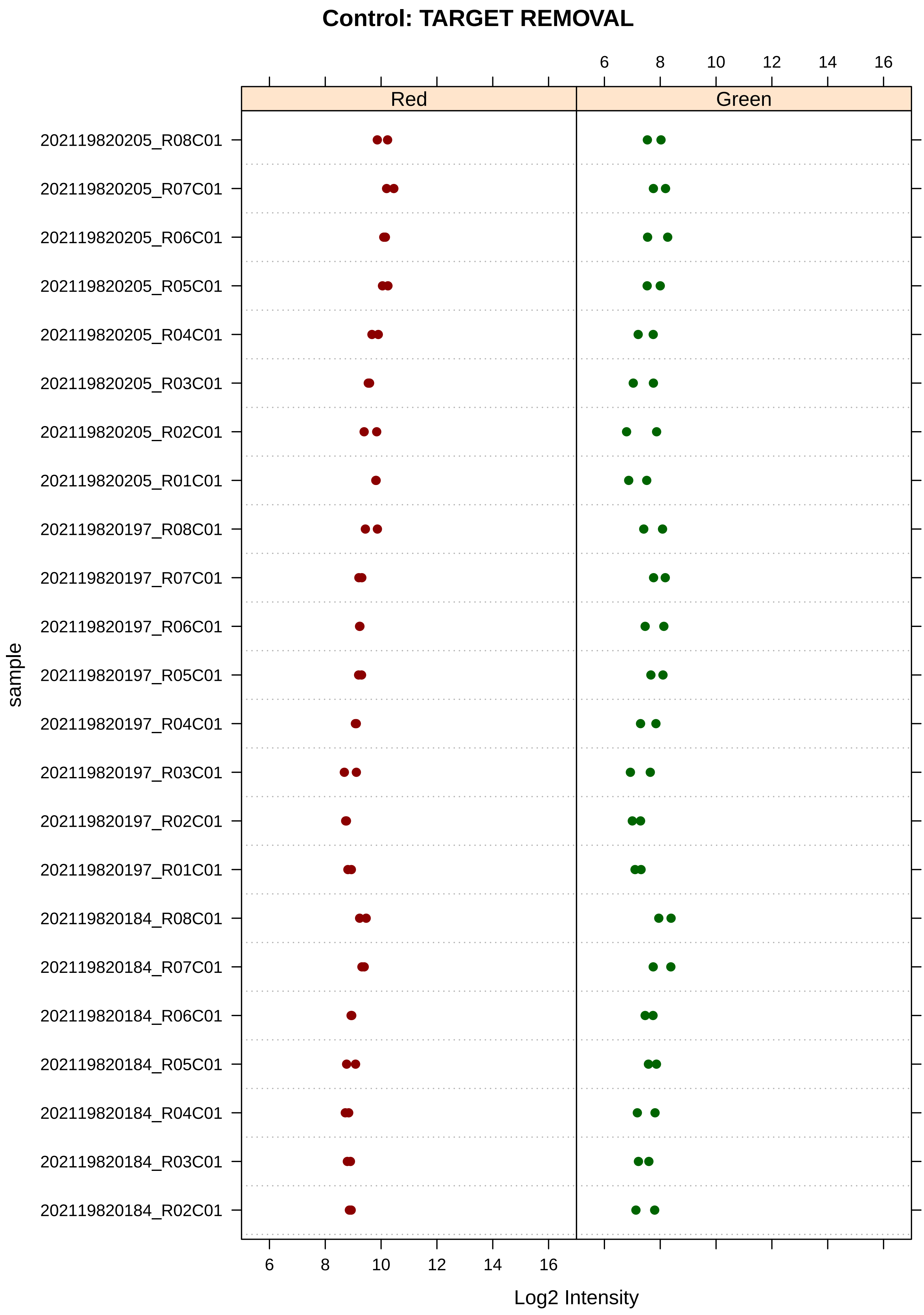
Control: TARGET REMOVAL
Red
Green
sample
Log2 Intensity
6
8
10
12
14
16
202119820205_R08C01
202119820205_R07C01
202119820205_R06C01
202119820205_R05C01
202119820205_R04C01
202119820205_R03C01
202119820205_R02C01
202119820205_R01C01
202119820197_R08C01
202119820197_R07C01
202119820197_R06C01
202119820197_R05C01
202119820197_R04C01
202119820197_R03C01
202119820197_R02C01
202119820197_R01C01
202119820184_R08C01
202119820184_R07C01
202119820184_R06C01
202119820184_R05C01
202119820184_R04C01
202119820184_R03C01
202119820184_R02C01

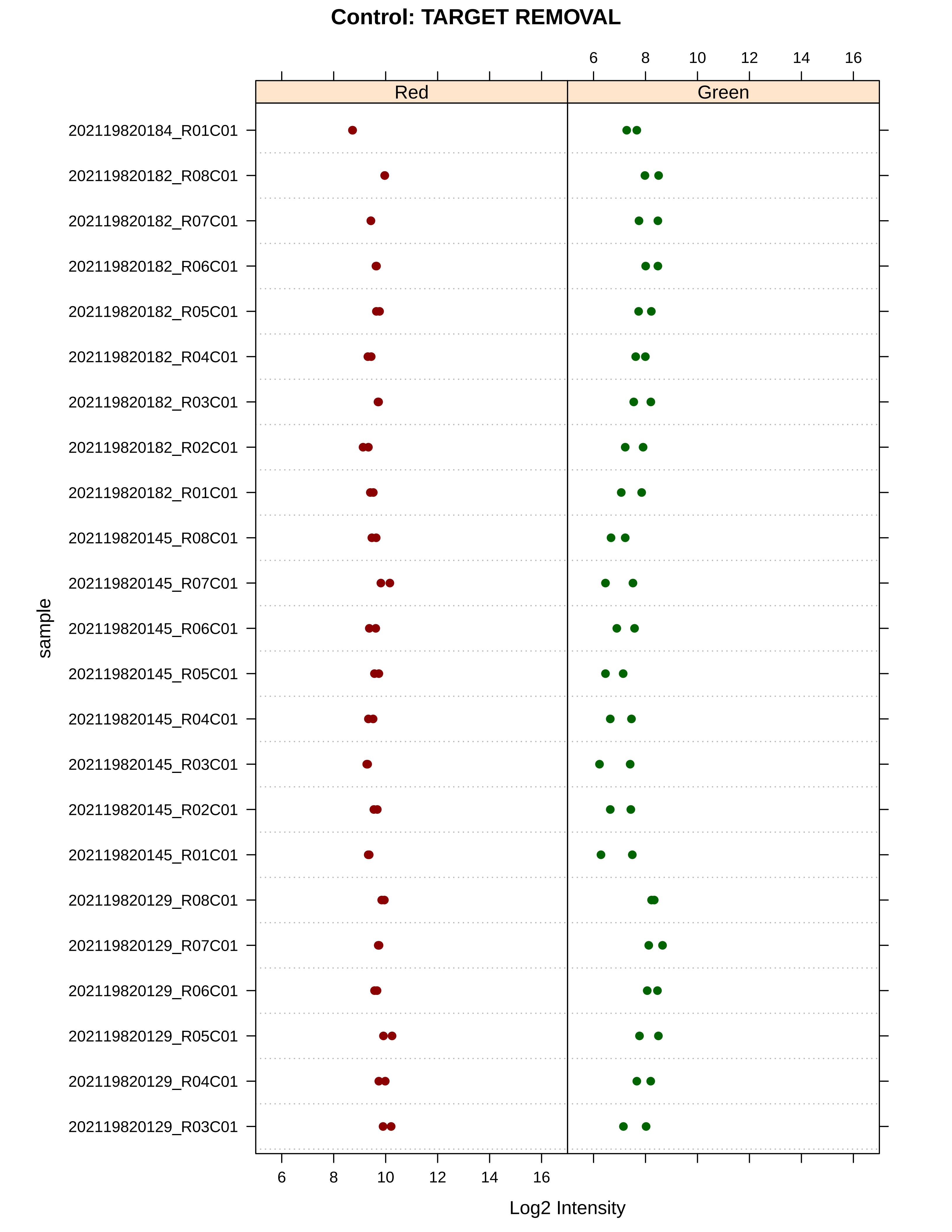

Control: TARGET REMOVAL
Red
Green
sample
202119820184_R01C01
202119820182_R08C01
202119820182_R07C01
202119820182_R06C01
202119820182_R05C01
202119820182_R04C01
202119820182_R03C01
202119820182_R02C01
202119820182_R01C01
202119820145_R08C01
202119820145_R07C01
202119820145_R06C01
202119820145_R05C01
202119820145_R04C01
202119820145_R03C01
202119820145_R02C01
202119820145_R01C01
202119820129_R08C01
202119820129_R07C01
202119820129_R06C01
202119820129_R05C01
202119820129_R04C01
202119820129_R03C01
6 8 10 12 14 16
Log2 Intensity

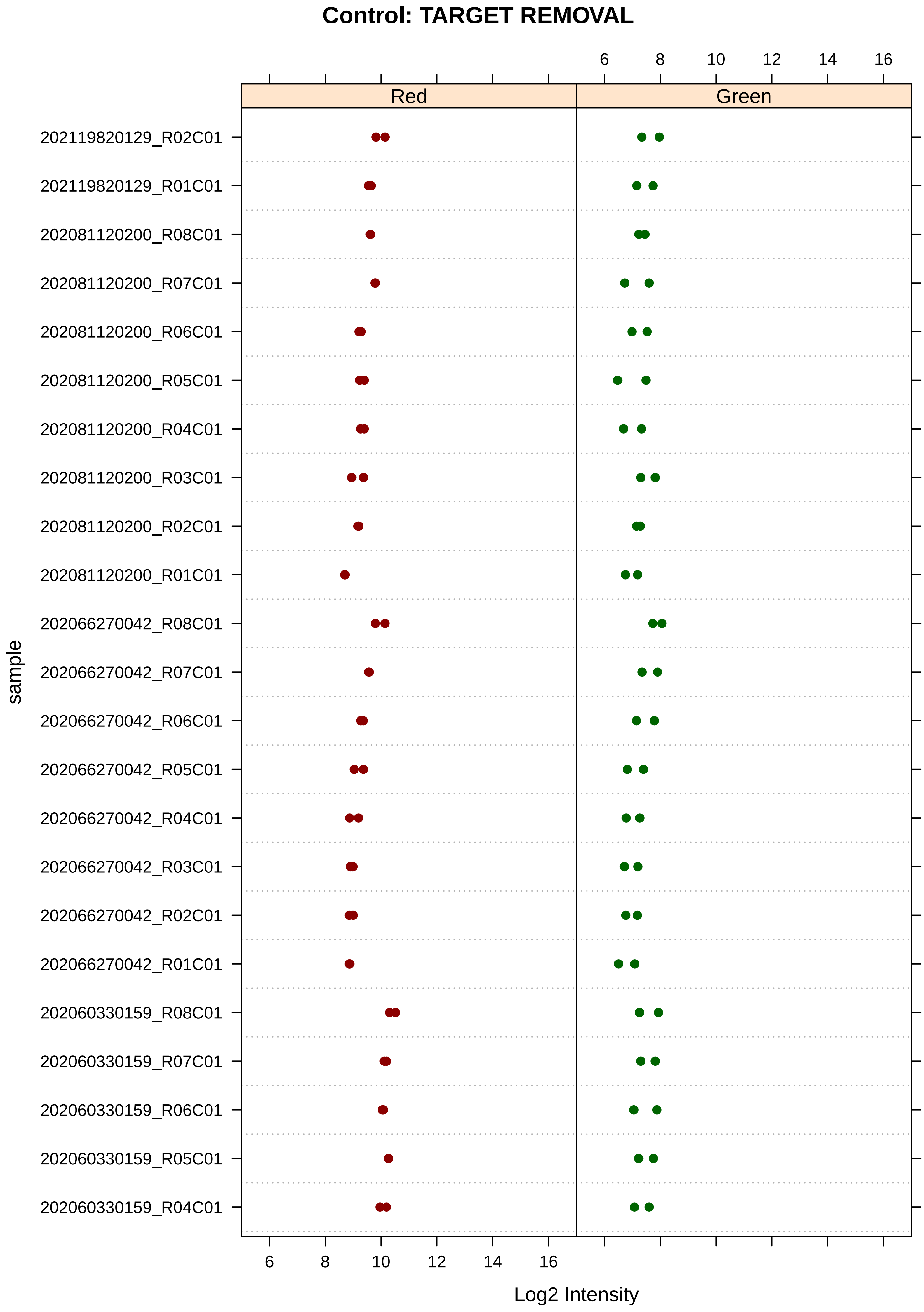
Control: TARGET REMOVAL
Red
Green
sample
Log2 Intensity
6
8
10
12
14
16
202119820129_R02C01
202119820129_R01C01
202081120200_R08C01
202081120200_R07C01
202081120200_R06C01
202081120200_R05C01
202081120200_R04C01
202081120200_R03C01
202081120200_R02C01
202081120200_R01C01
202066270042_R08C01
202066270042_R07C01
202066270042_R06C01
202066270042_R05C01
202066270042_R04C01
202066270042_R03C01
202066270042_R02C01
202066270042_R01C01
202060330159_R08C01
202060330159_R07C01
202060330159_R06C01
202060330159_R05C01
202060330159_R04C01

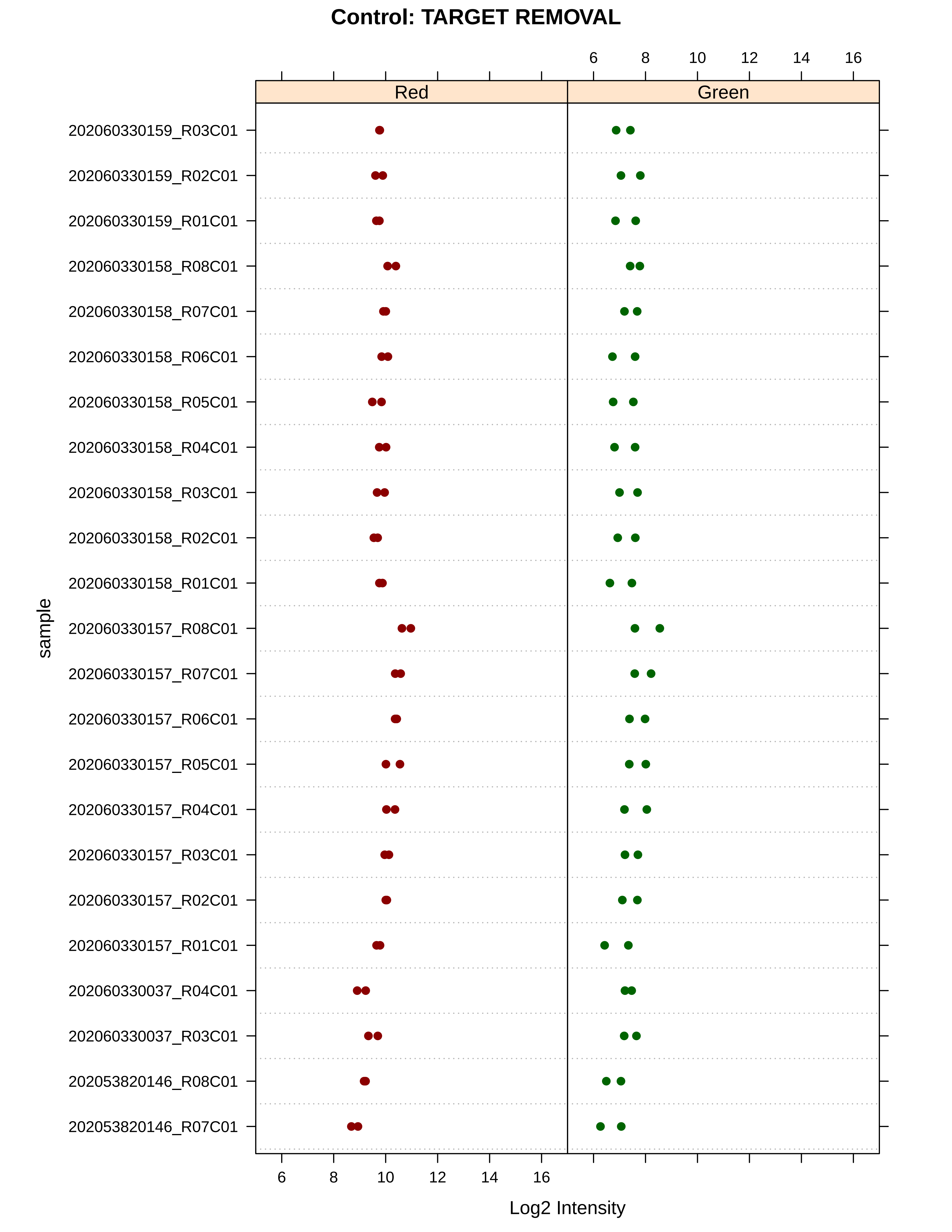

Control: TARGET REMOVAL
Red
Green
sample
Log2 Intensity
202060330159_R03C01
202060330159_R02C01
202060330159_R01C01
202060330158_R08C01
202060330158_R07C01
202060330158_R06C01
202060330158_R05C01
202060330158_R04C01
202060330158_R03C01
202060330158_R02C01
202060330158_R01C01
202060330157_R08C01
202060330157_R07C01
202060330157_R06C01
202060330157_R05C01
202060330157_R04C01
202060330157_R03C01
202060330157_R02C01
202060330157_R01C01
202060330037_R04C01
202060330037_R03C01
202053820146_R08C01
202053820146_R07C01
6
8
10
12
14
16

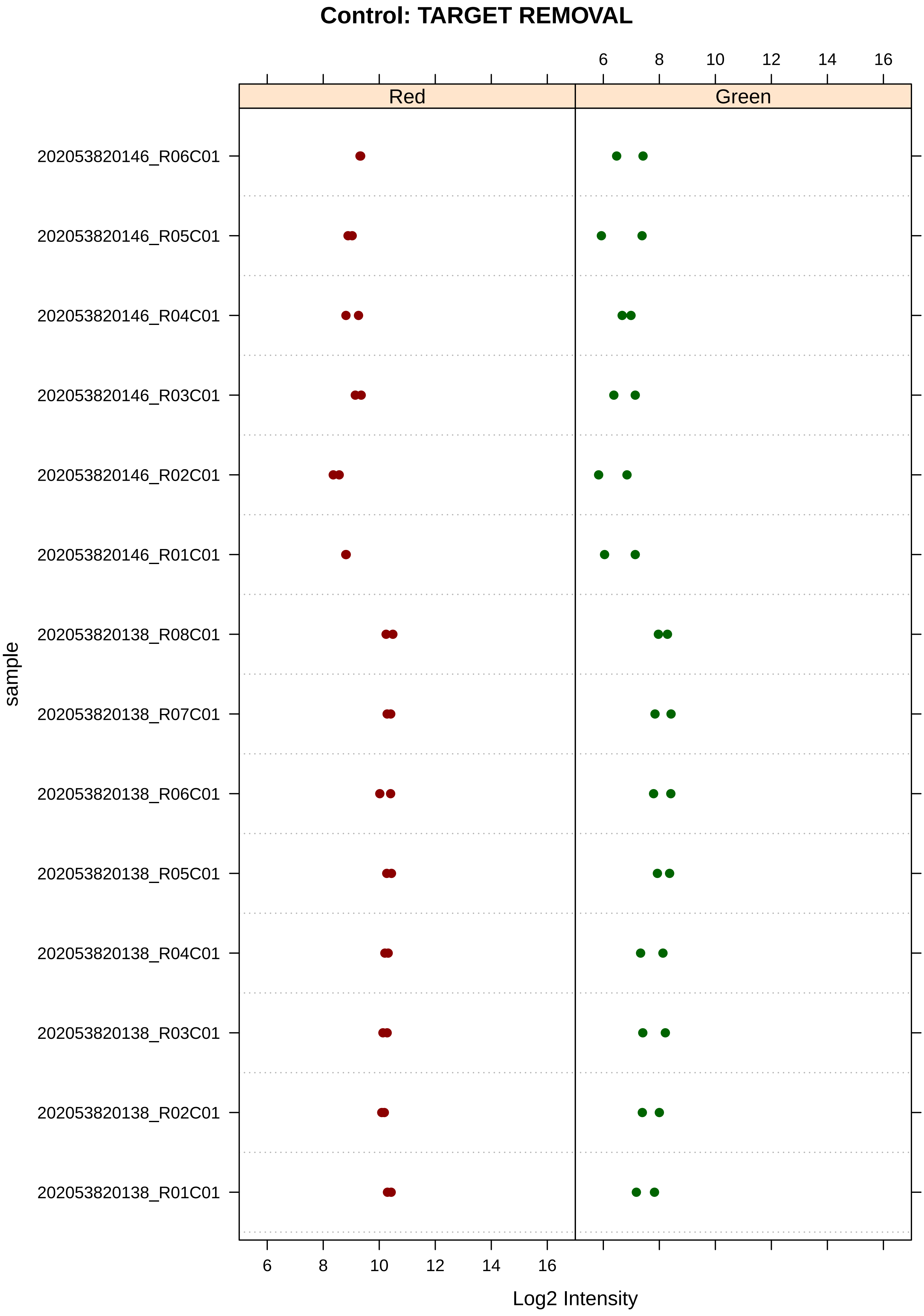

Control: TARGET REMOVAL
Red
Green
sample
202053820146_R06C01
202053820146_R05C01
202053820146_R04C01
202053820146_R03C01
202053820146_R02C01
202053820146_R01C01
202053820138_R08C01
202053820138_R07C01
202053820138_R06C01
202053820138_R05C01
202053820138_R04C01
202053820138_R03C01
202053820138_R02C01
202053820138_R01C01
6
8
10
12
14
16
Log2 Intensity
